# Recent Advances in Enantioselective Pd-Catalyzed Allylic Substitution: From Design to Applications

**DOI:** 10.1021/acs.chemrev.0c00736

**Published:** 2021-03-19

**Authors:** Oscar Pàmies, Jèssica Margalef, Santiago Cañellas, Jinju James, Eric Judge, Patrick J. Guiry, Christina Moberg, Jan-E. Bäckvall, Andreas Pfaltz, Miquel A. Pericàs, Montserrat Diéguez

**Affiliations:** † Universitat Rovira i Virgili, Departament de Química Física i Inorgànica, C/Marcel·lí Domingo, 1, 43007 Tarragona, Spain; ‡ Discovery Sciences, Janssen Research and Development, Janssen-Cilag, S.A. Jarama 75A, 45007, Toledo, Spain; § Centre for Synthesis and Chemical Biology, School of Chemistry, University College Dublin, Belfield, Dublin 4, Ireland; ∥ KTH Royal Institute of Technology, Department of Chemistry, Organic Chemistry, SE 100 44 Stockholm, Sweden; ⊥ Department of Organic Chemistry, Arrhenius Laboratory, Stockholm University, SE 106 91 Stockholm, Sweden; # Department of Chemistry, University of Basel. St. Johanns-Ring 19, 4056 Basel, Switzerland; ¶ Institute of Chemical Research of Catalonia (ICIQ), The Barcelona Institute of Science and Technology, Av. Països Catalans 16, 43007 Tarragona, Spain; □ Departament de Química Inorgànica i Orgànica, Universitat de Barcelona. 08028 Barcelona, Spain

## Abstract

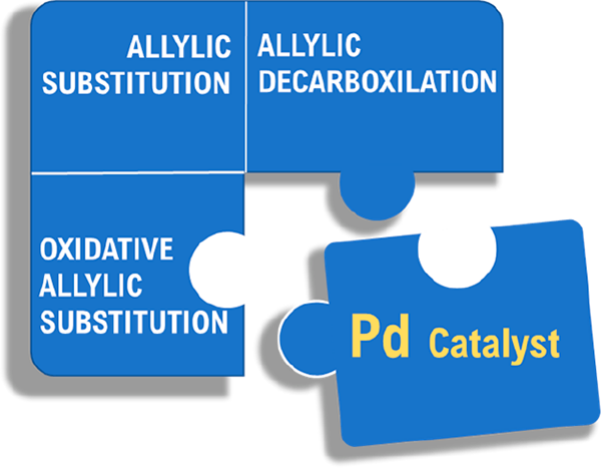

This Review compiles the evolution, mechanistic understanding, and more recent advances in enantioselective Pd-catalyzed allylic substitution and decarboxylative and oxidative allylic substitutions. For each reaction, the catalytic data, as well as examples of their application to the synthesis of more complex molecules, are collected. Sections in which we discuss key mechanistic aspects for high selectivity and a comparison with other metals (with advantages and disadvantages) are also included. For Pd-catalyzed asymmetric allylic substitution, the catalytic data are grouped according to the type of nucleophile employed. Because of the prominent position of the use of stabilized carbon nucleophiles and heteronucleophiles, many chiral ligands have been developed. To better compare the results, they are presented grouped by ligand types. Pd-catalyzed asymmetric decarboxylative reactions are mainly promoted by PHOX or Trost ligands, which justifies organizing this section in chronological order. For asymmetric oxidative allylic substitution the results are grouped according to the type of nucleophile used.

## 1 Introduction

Sustainable production is one of the important challenges facing our society. The efficient use of energy and raw materials and the reduction of waste are requirements for industrial growth. Leading companies are persistently looking for improvements to increase their competitive advantages. This has been especially noteworthy in the production of enantiopure compounds, which play a key role in many technologically and biologically relevant applications. The production of such compounds is growing and industry is searching for better synthetic procedures that are more selective, straightforward, less costly and environmentally friendly. In achieving these goals, asymmetric catalysis plays an essential role.^1−3^

Among the catalytic reactions leading to chiral products, enantioselective Pd-catalyzed allylic substitution and decarboxylative and oxidative allylic substitutions are unique in two respects. First, the enantioselectivity can be induced in several ways. Second, many types of bonds, such as C–C, C–N, and C–O bonds, can be formed with the same catalyst, and the resulting products can be further transformed by taking advantage of the alkene functionality. Other advantages are the high functional group tolerance and mild reaction conditions typically employed. In the past decade impressive results have been obtained in the development of highly efficient catalytic systems by exploring new generations of ligands, catalysts and reaction conditions. Great achievements have also been made in the development of new strategies including chiral counteranion methodology, synergistic dual Pd/PTC (chiral phase-transfer catalysts), synergistic dual Pd/organocatalysis, and synergistic dual bimetallic catalysis.^4−7^ Catalyst design relies increasingly on structural information, and computational studies (thanks to the advances in computational power and methods) are increasingly being used, moving away from the costly trial-and-error based discovery. Remarkable efforts have also been made to enlarge the scope of substrates and nucleophiles, thereby increasing the possibilities for applications to the synthesis of more complex organic molecules. Novel tandem reactions have been developed, such as allylic substitution and ring-closing metathesis or Pauson–Khand reactions, which have been efficiently applied in the preparation of chiral (poly)carbo- and heterocyclic compounds.

**Figure 1:**
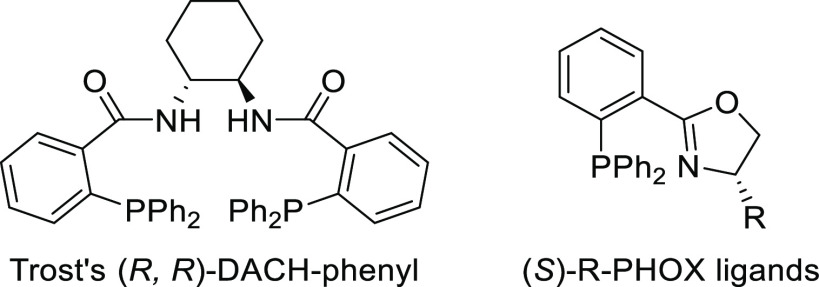
Trost’s diphosphine ligands and phosphine-oxazoline PHOX ligands.

Despite the extensive research dedicated to the field, the existing general reviews are very old (e.g., the latest covering allylic alkylation is a *Chem. Rev.* article by Trost from 2003).^8^ There are more recent reviews (most of them cover the advances made until 2011–2012), but these reviews are mostly microreviews or book chapters that mainly cover narrow specific areas (e.g., one type of nucleophile, substrate or one type of ligand or only describe mechanistic aspects, ...) or they only cover one of the three reactions that are discussed in this review.^9−41^ A comprehensive review that discusses the latest advances in mechanistic studies, catalytic results and applications for the three reactions, is important for the development of future research. This Review covers the literature from 2008, and we compile, for each reaction, the catalytic data, as well as examples of their application to the synthesis of more complex molecules. We also include sections in which we discuss key mechanistic aspects for high selectivity and a comparison with other metals (with advantages and disadvantages). For Pd-catalyzed asymmetric allylic substitution, we have grouped the catalytic data according to the type of nucleophile employed. Because of the prominent position of the use of stabilized carbon nucleophiles and heteronucleophiles, many chiral ligands have been developed. To better compare the results, we will present them grouped by ligand types. Pd-catalyzed asymmetric decarboxylative reactions are mainly promoted by Trost ligands or PHOX ligands (Figure 1), which justifies organizing this section in chronological order. For asymmetric oxidative allylic substitution the results are grouped according to the type of nucleophile used.

## 2 Asymmetric Allylic Substitution

### 2.1 Substrate Types

The catalyst performance depends fundamentally on the nature of the substrate. For instance, a Pd-catalyst with Trost’s ligands is well suited for unhindered disubstituted substrates (both linear and cyclic), while the PHOX-based Pd-catalysts work well with hindered disubstituted substrates (Figure 1).^42−45^ In this respect, research on Pd-catalyzed allylic substitution has been widely directed toward reducing the substrate dependency of the catalyst. In the past decade, some catalytic systems with heterodonor ligands use the same ligand to alkylate disubstituted hindered and unhindered substrates. Substantial progress has also been made to enlarge the scope of substrates and nucleophiles, thereby increasing the possibilities for applications to the synthesis of more complex organic molecules.^46−49^

Most substrates belong to the group of so-called activated allylic substrates that contain a readily reacting leaving group, with acetate and carbonates being the most common. These substrates produce stoichiometric amounts of waste, which has a significant environmental and economic impact. For this reason, unactivated allylic substrates (such as allylic alcohols, allylic ethers, vinyl epoxides, allylic amines, ...) have become more popular^26^ although they require additives (e.g., Lewis acids, Brønsted acids, ...) to activate them under the reaction conditions used. Among the unactivated allylic substrates, allylic alcohols are the most popular because they are easily available and usually require only catalytic amounts or substoichiometric quantities of the additive. This contrasts with the higher stability of allylic ethers and amines that require the presence of the activating additive in stoichiometric amounts.

#### 2.1.1 1,3-Disubstituted Substrates with Identical Substituents at the Allylic Termini

**Figure 2:**
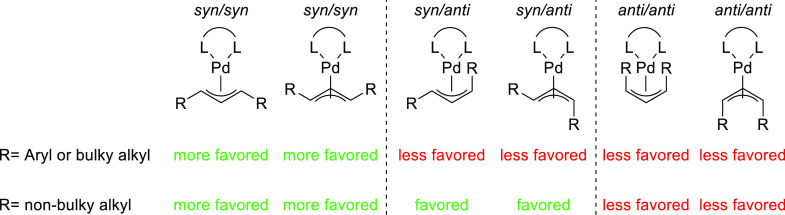
Possible formed Pd η^3^-allyl intermediates in symmetrical 1,3-disubstituted linear substrates depending on the nature of the allyl substitutents.

1,3-Disubstituted allyl esters with identical substituents at C1 and C3, which give rise to symmetrical allyl intermediates, are the most common substrates in Pd-catalyzed allylic substitution. Among them, linear 1,3-diarylallyl esters are most popular, with *rac*-1,3-diphenylallyl acetate often serving as a model substrate (Figure 2, R = Ph). This substrate class has the advantage that compared with unsymmetrically substrates there are no regioselectivity issues. In addition, it is easier to achieve high enantioselectivity because *syn*/*syn* isomers are energetically strongly favored over the *syn*/*anti* and *anti*/*anti* isomers (Figure 2). The less favorable *syn*/*anti* and *anti*/*anti* isomers are generated in large amounts only for catalytic systems that are sterically congested around the allylic system, which disfavors the formation of *syn*/*syn* isomers. PHOX-type ligands have been considered the ligands of choice for these type of substrates.

The enantioselectivity for linear 1,3-dialkylallyl substrates is more difficult to control than for the corresponding diaryl derivatives, especially for those bearing less sterically demanding alkyl groups like *rac*-1,3-dimethyl-3-acetoxyprop-1-ene (Figure 2, R = Me). For such substrates the isomers arising from the *syn*/*anti* disposition of the alkyl groups must also be considered as undesired intermediates in which the nucleophile can attack the allylic system (Figure 2). For these less sterically hindered substrates, the Trost type ligands have proved to be optimal.

For cyclic substrates only the *anti*/*anti* geometry is possible. Since there are only small hydrogen substituents at the terminal allylic carbon atoms to guide enantiodiscrimination, the enantioselectivity is more difficult to control than for linear substrates as the catalyst must generate a more precisely confined chiral pocket. For this substrate class Trost’s ligands have played a predominant role.

#### 2.1.2 1,3-Disubstituted Substrates with Nonidentical Substituents at the Allylic Termini

**Scheme 1:**
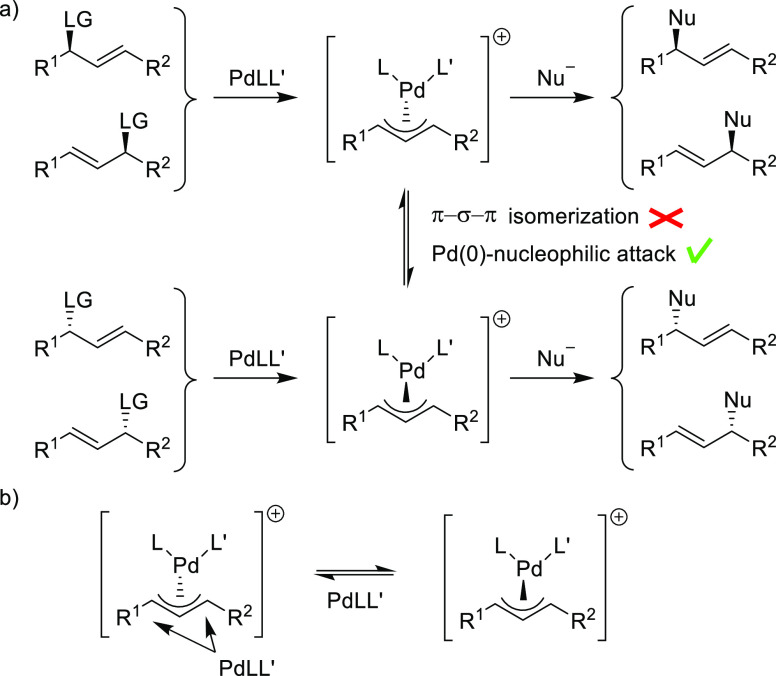
(a) Pd-Catalyzed Allylic Substitution of Unsymmetrically 1,3-Disubstituted Substrates and (b) Epimerization of Pd-Allyl Complexes via Pd(0)-Catalyzed Allyl Exchange

Racemic 1,3-disubstituted substrates with different substituents at C1 and C3 are a challenging class of substrates because of the additional problem of regiocontrol and because two isomeric allyl intermediates are formed, which cannot interconvert via π–σ–π isomerization (which merely results in *syn–anti* isomerization) (Scheme 1a). Interconversion can, in principle, occur by a process in which a Pd(0) complex acts as a nucleophile and replaces the Pd(II) complex bound to the allyl system by back-side attack with inversion of configuration (Scheme 1b).^50,51^ In general this so-called Pd(0)-catalyzed allyl exchange is not observed and, consequently, a mixture of two enantioenriched regioisomers is obtained. In this case, the catalyst only influences the regioselectivity, while the product configuration is determined by the configuration of the substrate as the overall reaction proceeds with retention of configuration. Thus, conversion of a racemic substrate to a single enantioenriched product is not possible, as described above for substrates having two identical R substituents. However, it has been found in some cases that one of the possible products can be obtained regio- and enantioselectively by kinetic resolution. On the other hand, a few successful examples have been reported in which a dynamic kinetic resolution takes place through rapid interconversion of the two allyl intermediates, converting both substrate enantiomers preferentially to a single enantioenriched product.^52−55^

#### 2.1.3 Monosubstituted Substrates

**Figure 3:**
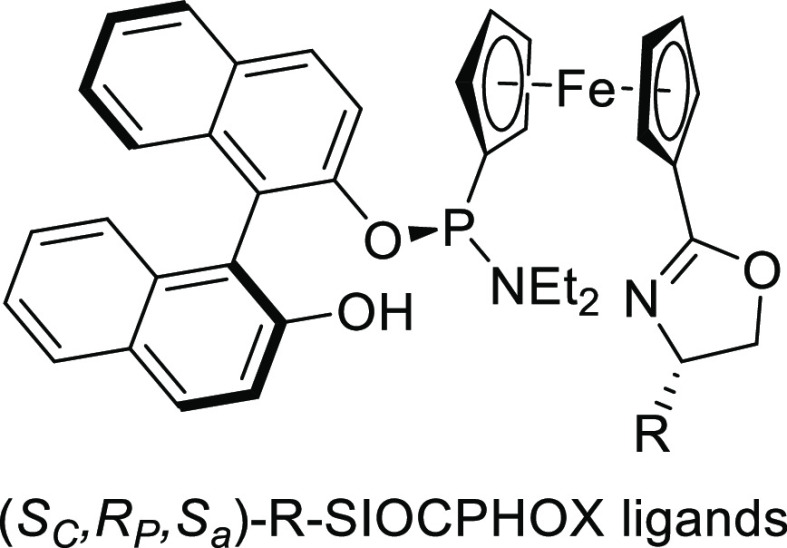
Ferrocene-binol-based P-oxazoline (*S*_C_,*R*_P_,*S*_a_)-R-SIOCPHOX Ligands.

Monosubstituted substrates pose the additional challenge that two regioisomers, the α- and the γ-products, can be obtained, so regioselectivity must be controlled. Most of the Pd-catalysts favor the formation of linear isomers which, unless a prochiral nucleophile is used, leads to undesired achiral products. Although specific ligands have been reported that favor the formation of branched product, their scope is still limited compared to catalysts based on Ir and Mo (for stabilized carbon nucleophiles) and Cu (for nonstabilized carbon nucleophiles). Specifically Ir-complexes have become the catalysts of choice for this class of substrates.^56^ For Pd-catalysts the ferrocene-binol based P-oxazoline SIOCPHOX ligands represent the state of the art for this substrate type providing high regio- and enantioselectivities with several type of nucleophiles (Figure 3).^57^

#### 2.1.4 Trisubstituted Substrates

This is another challenging substrate class. To simplify the overall picture, most of the substrates have two identical geminal substituents (e.g., *rac*-1,1-diphenyl-1-hepten-3-yl acetate) so the regioselectivity can be controlled by steric constraints favoring nucleophilic attack at the less substituted allylic carbon terminus. The enantioselectivity is controlled by the chiral Pd-catalyst and PHOX-type ligands have played a dominant role for these substrates.^10^

#### 2.1.5 *Meso*-Substrates with Two Enantiotopic Leaving Groups

**Scheme 2:**
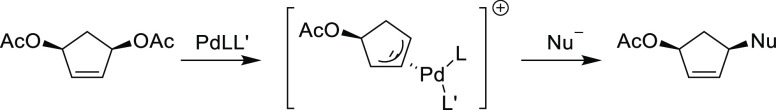
Pd-Catalyzed Allylic Desymmetrization of *meso*-Cyclopent-4-ene-1,3-diyl Diacetate

Substrates of this type have also been extensively studied.^10^ Special attention has been paid to *meso*-cycloalkenediol derivatives (e.g., *meso*-cyclopent-4-ene-1,3-diyl diacetate) because they lead to important chiral synthons for the synthesis of biologically active compounds. They can be desymmetrized using a chiral catalyst by regioselective displacement of one the leaving groups by the nucleophile. Nucleophilic attack then takes place at the less hindered allylic terminus, resulting in the replacement of one of the leaving groups by the nucleophile with overall retention of configuration (Scheme 2). It should be mentioned that the desymmetrized products can be subjected to a second allylic substitution, which increases the diversity of products that are accessible by this approach.

### 2.2 Malonates, Related Stabilized C-Nucleophiles, and O-, S-, N-, and P-Nucleophiles

Stabilized carbon nucleophiles, such as carbanions derived from 1,3-dicarbonyl compounds, maintain a prominent position in enantioselective Pd-catalyzed allylic substitutions. Apart from malonates and related stabilized C-nucleophiles including various functionalized malonates, β-diketones, 2-cyanoacetates, pyrroles, nitromethane, etc., N- and O-nucleophiles and to a lesser extent P- and S-nucleophiles have been used. Among the reactions studied the alkylation of *rac*-1,3-diphenylallyl acetate using malonates, and especially dimethyl malonate as the nucleophile, continues to serve, together with the Rh-catalyzed asymmetric hydrogenation of dehydroamino acid derivatives, as benchmark reactions to evaluate the potential of new ligands in asymmetric catalysis. Accordingly, since 2008, a vast number of papers on the development of new ligands for the alkylation of this benchmark substrate with malonate derivatives were published. Ligand design covered a wide array of structures ranging from monodentate P-donor ligands to homo- and heterodonor bidentate ligands. In this section alone, more than one hundred new ligand families have been developed and applied with success. Although bidentate ligands continue to maintain a privileged position, some monodentate ligands such as the TADDOL-based phosphoramidites and binaphthol-based phosphoramidites (the so-called Feringa type ligands) have provided outstanding results on more challenging and synthetically interesting substrates or/and nucleophiles (section 2.2.1). An important part of the research has also been directed to reduce the substrate dependency. Thus, some P,P′, P,N, and P,S-ligand families (sections 2.2.5, 2.2.6.2, and 2.2.8, respectively) use the same ligand to successfully alkylate disubstituted hindered and unhindered substrates and even monosubstituted substrates. On the other hand, from a synthetic point of view, many recent studies were also devoted to more valuable and more challenging substrates and/or nucleophiles using well-established ligand scaffolds or slight modifications of them (e.g., Trost’s and PHOX type ligands; sections 2.2.2.1, and 2.2.6.1, respectively). In this respect, some noteworthy studies have also been published on the use of well-known diphosphine ligands, such as BINAP-type, BIPHEP, and SegPhos (section 2.2.2.2). Thus, many types of C-nucleophiles including various functionalized malonates, β-diketones, 2-cyanoacetates, pyrroles, etc., N- and O-nucleophiles, and to a lesser extent P- and S-nucleophiles, have been studied with success. In the following sections, we compile the catalytic data reported grouped by ligand types. To compare the results from each group of ligands, we first discuss the data reported for newly designed ligands, which have been mostly applied in the allylic alkylation of 1,3-disubstituted linear substrates with identical substituents at C1 and C3 using malonates as nucleophiles. Subsequently, we summarize the results obtained with other nucleophiles and substrates. In the Bidentate Homodonor P,P-Ligands (section 2.2.2) and Bidentate Heterodonor P,N(sp^2^)-Ligands (section 2.2.6) subsections, the application of Trost diphosphine ligands and phosphine-oxazoline PHOX type ligands have been included.

#### 2.2.1 Monodentate P-Donor Ligands

**Scheme 3:**
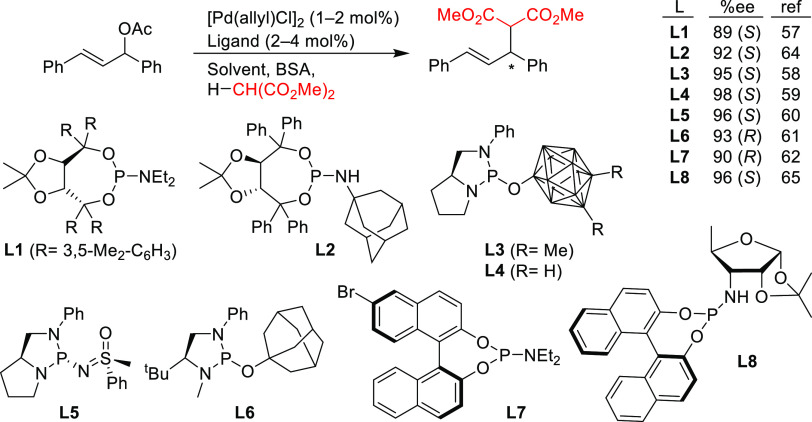
Representative Examples of Monodentate P-Donor Ligands Applied in the Pd-Catalyzed AAA of *rac*-1,3-Diphenylallyl Acetate Using Dimethyl Malonate as Nucleophile

Among the monodentate P-donor ligands developed since 2008 for Pd-catalyzed asymmetric allylic alkylation (AAA), diamidophosphites^58−62^ and phosphoramidites^63−66^ were found to provide higher enantioselectivities than their phosphite,^67,68^ phosphinite,^69^ and phosphine^70−72^ counterparts. Scheme 3 collects the most representative families of monodentate P-donor ligands evaluated in the allylic alkylation of *rac*-1,3-diphenylallyl acetate as substrate with dimethyl malonate as nucleophile. Two of them are TADDOL-based phosphoramidite ligands (**L1**–**L2**), four are P*-chiral diazophospholidine-based ligands (**L3**–**L6**), one is a binaphthyl-based ligand (**L7**) and one is a furanoside-based ligand (**L8**). Enantioselectivities of up to 98% *ee* were obtained (see Scheme 3). With monodentate ligands **L2**,^65^**L4**,^60^ and **L5**(61) the same levels of enantioselectivity were also achieved with pyrrolidine and sodium *para*-toluene sulfinate as nucleophiles. Bauer’s group also studied other substrates using Pd–**L8** as catalyst.^66^ Although only low enantioselectivities (up to 49% *ee*) were achieved in the Pd-allylic alkylation of less sterically hindered substrates (e.g., *rac*-1,3-dimethylallyl and cyclohexenyl carbonates), the Pd/**L8** catalyst yielded promising results for unsymmetrically substituted linear substrates like 4-phenylbut-3-en-2-yl acetate (with regio- and enantioselectivities up to 75% and 90% *ee*, respectively) and cinnamyl acetate (with regio- and enantioselectivities up to 93% and up to 79% *ee*, respectively).

**Scheme 4:**
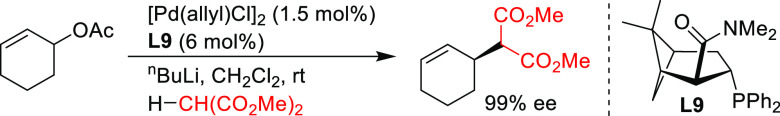
Pd-Catalyzed Allylic Alkylation of Cyclohexenyl Acetate Using Monodentate Phosphine **L9**

High *ee* values for unhindered cyclic substrates with monodentate ligands are unusual. An exception was found with a catalyst derived from the monophosphine ligand **L9** (*ee* values up to 99% in the allylic alkylation of cyclohexenyl acetate; Scheme 4). The mechanism of this catalyst system was investigated by a combination of advanced NMR spectroscopic methods and DFT calculations. Since the allyl intermediates are difficult to study by standard NMR spectroscopic methods (^3^*J* and NOE) because of the high conformational flexibility, additional information was acquired from residual dipolar couplings (RDC). Determination of the RDC data required orientation of the air- and moisture-sensitive intermediate in an anisotropic medium (high molecular-weight poly(α-benzyl-l-glutamate, PBLG, see section 2.4).^73^

Further work on monodentate P-donor ligands focused on their use in the allylic substitution of other synthetically interesting substrates or nucleophiles. Most studies were carried out on binaphthol-based phosphoramidites, demonstrating that modifications of the binol backbone, and especially of the amine part, were crucial for obtaining high enantioselectivity.

**Scheme 5:**
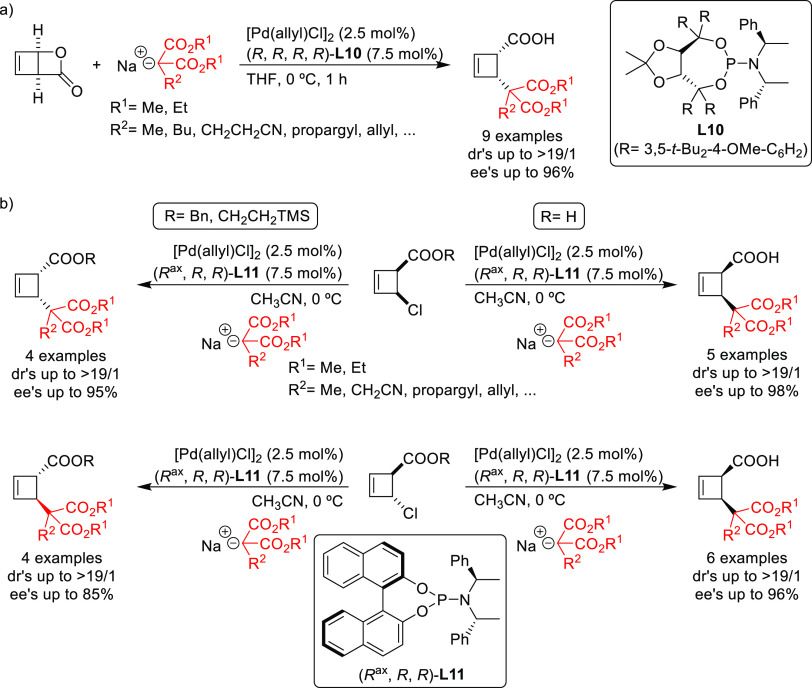
Deracemization of (a) *cis*-2-Oxabicyclo[2.2.0]hex-5-en-3-one and (b) *cis*- and *trans*-4-Chlorocyclobut-2-ene Carboxylic Acid Derivatives Using a Range of Malonates

In this respect, the group of Maulide reported some notable examples,^74,75^ showing that the TADDOL-based phosphoramidite ligand **L10** can efficiently control the deracemization of the strained lactone *cis*-2-oxabicyclo[2.2.0]hex-5-en-3-one with a range of malonates (Scheme 5a).^74^ The reactions were highly *cis*-selective providing the alkylated products in high diastereo- and enantioselectivities (up to >19/1 and 96% *ee*, respectively). Subsequently, they also identified a catalyst, the Pd/**L11** complex with a Feringa type ligand, that efficiently deracemizes *cis*- and *trans*-4-chlorocyclobut-2-ene carboxylic acid with malonates (Scheme 5b).^75^ These reactions were again highly *cis*-selective for both substrates providing the alkylated products in high diastereo- and enantioselectivities (up to >19/1 and 98% *ee*, respectively). Notably, the reactivity of carboxylic esters differs from that of the free carboxylic acids. Whereas the reaction of *cis*-4-chlorocyclobut-2-ene carboxylic esters proceeded with high *cis*-selectivity, the reaction of the *trans*-isomers led to *trans*-cyclobutenes (Scheme 5b). Interestingly, in all cases, the use of PHOX ligands instead of monophosphoramidites **L10** and **L11** led to the preferential formation of *trans*-isomers in high dr’s and *ee* values (see section 2.2.6.1.).

**Scheme 6:**
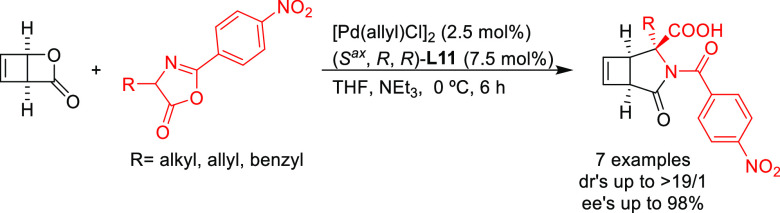
Deracemization of *cis*-2-Oxabicyclo[2.2.0]hex-5-en-3-one Using Azlactones

Maulide’s group further extended the nucleophile scope to include azlactones in the Pd-catalyzed allylic alkylation of the strained lactone, *cis*-2-oxabicyclo[2.2.0]hex-5-en-3-one, as substrate (Scheme 6).^74^ For this transformation, which involves the combination of two prochiral compounds, the monophosphoramidite (*S*^ax^,*R*,*R*)-**L11** provided excellent diastereo- and enantioselectivities (up to >19/1 and 98% *ee*, respectively).

**Scheme 7:**
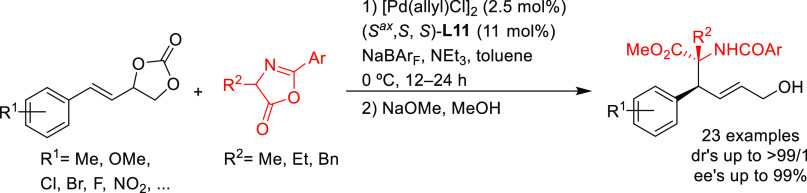
Pd-Catalyzed Allylic Alkylation of 4-Arylvinyl-1,3-dioxolan-2-ones with Azlactones

Later, Zhang and co-workers further explored azlactones in the Pd-catalyzed allylic alkylation of 4-arylvinyl-1,3-dioxolan-2-ones (Scheme 7).^76^ They found that Pd/(*S*^ax^,*S*,*S*)-**L11** provided the corresponding branched chiral α-amino acids with vicinal tertiary and quaternary stereocenters with excellent selectivities (dr’s up to >99/1 and *ee* values up to 99%).

**Scheme 8:**
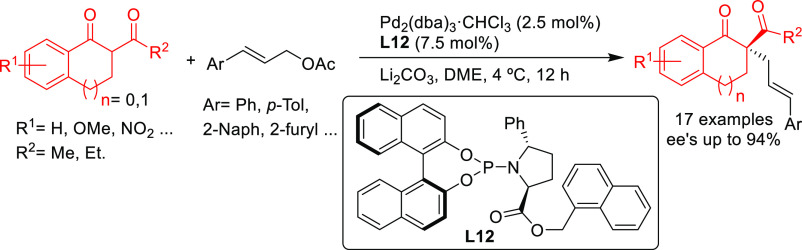
Pd-Catalyzed Allylic Alkylation of Cinnamyl Acetate Derivatives with 1,3-Diketones Using Pd/**L12** Catalytic System

In 2015, the Trost group developed a novel nonsymmetric binaphthol-based phosphoramidite ligand **L12** that was successfully applied in the allylic alkylation of a range of cinnamyl acetate derivatives with several 1,3-diketones (*ee* values up to 94%; Scheme 8).^77^

**Scheme 9:**
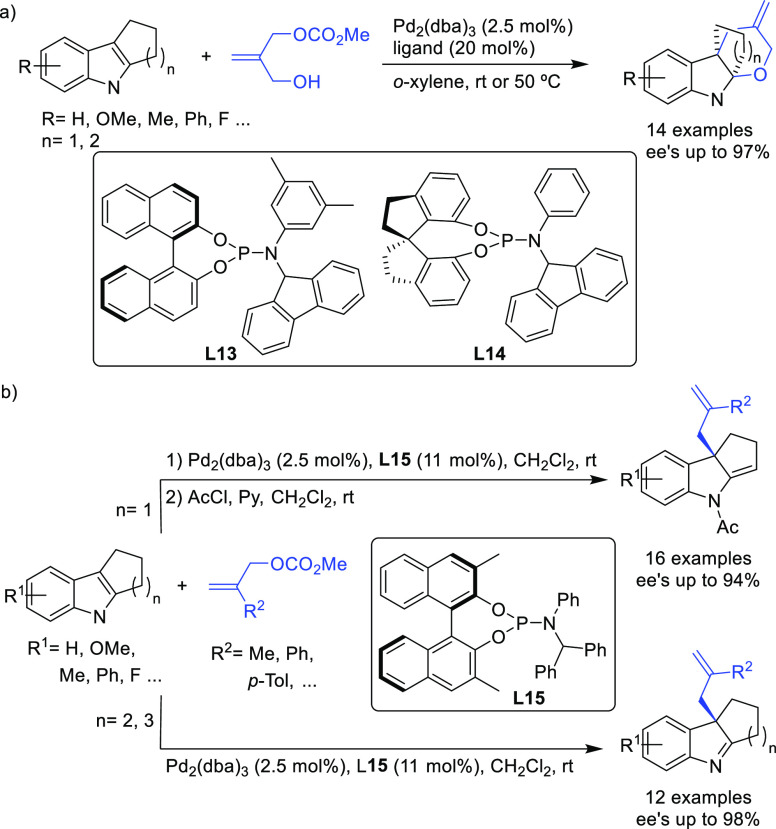
Pd-Catalyzed Allylic Dearomatization of Polycyclic Indoles with Allylic Carbonates

Monophosphoramidites have also been successfully used in the Pd-catalyzed allylic dearomatization of indoles.^78,79^ Different from ligands **L10**–**L12**, the chirality of the binaphthol or spiro backbone alone is sufficient to induce high enantioselectivities. A range of indoles with a fused cyclopentane or cyclohexane group were used as C-nucleophiles in the allylic alkylation of 2-(hydroxymethyl)allyl methyl carbonate (Scheme 9a).^78^ Because of the presence of a hydroxy group in the side chain of the substrate, the reaction proceeds in a cascade fashion providing bridged indolines with excellent enantioselectivities (up to 97% *ee*). Interestingly, the selection of the ligand depends on the size of the indole fused ring: whereas the binaphthol-based phosphoramidite ligand **L13** provides the best results for indoles with a fused cyclopentane ring, the cyclohexane-based indoles performed best with the phosphoramidite ligand **L14** with a spirocyclic backbone.

The same research group later developed a Pd-allylic dearomatization of indoles with several allylic carbonates bearing an alkyl or aryl substituent at the C2 position (Scheme 9b).^79^ In this case, the binaphthol-based monophosphoramidite **L15**, which differs from ligand **L13** with respect to the substituents of the exocyclic amine, played a key role in achieving excellent enantiocontrol (up to 98% *ee*). It should be mentioned that indolenines derived from indoles with a fused cyclopentane are not stable upon purification by chromatography. To avoid this problem, the indolenines were transformed to the stable enamine derivatives by a one pot acetylation and isomerization process.

**Scheme 10:**
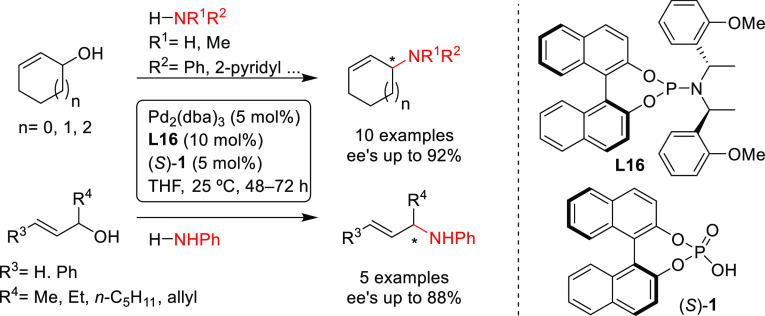
Pd-Catalyzed Allylic Amination of Unactivated Allylic Alcohols Using a Combination of Chiral Brønsted Acid ((*S*)-**1**) and Pd/**L16**-Catalytic System

Further notable examples on the use of binaphthol-based monophosphoramidite Pd-catalysts with nucleophiles other than carbon, such as N, O and S, have also been reported. In 2014, Beller and co-workers described the Pd-catalyzed allylic amination of nonactivated allylic alcohols for the synthesis of cyclic and acyclic allylic amines,^80^ using a combination of Pd_2_(dba)_3_, a binaphthol-based phosphoramidite (**L16**) and a Brønsted acid ((*S*)-**1**). Notably, cyclic and acyclic allylic alcohols were suitable for this transformation, affording the desired allylic amines in good-to-high enantioselectivities (up to 92% *ee*; Scheme 10).

**Scheme 11:**
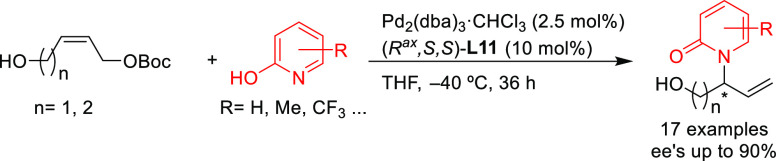
Synthesis of N-Substituted 2-Pyridones via Allylic Amination Using Pd/(*R*^ax^,*S*,*S*)-**L11** Catalytic System

Zhang’s group reported the allylic amination of hydroxy-containing allylic carbonates with 2-pyridones using Pd/(*R*^ax^,*S*,*S*)-**L11** as catalyst (Scheme 11).^81^ In this way *N*-substituted 2-pyridones are accessible with complete regioselectivity and high enantioselectivities (up to 90% *ee*).

**Scheme 12:**
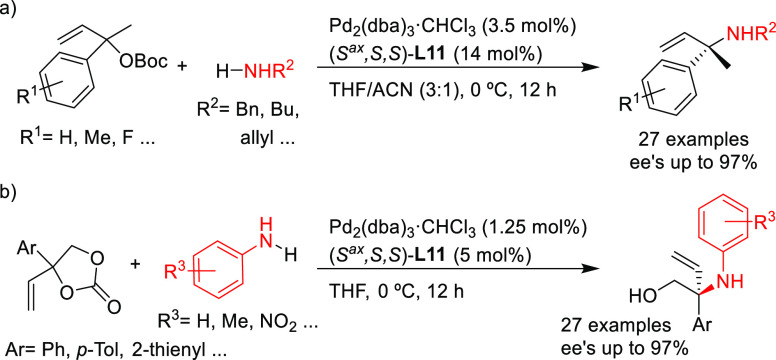
Pd-Catalyzed Allylic Amination of (a) α,α-Disubstituted Allylic Carbonates and (b) Vinyl Cyclic Carbonates Using Pd/(*S*^ax^*,S*,*S*)-**L11** Catalytic System

Another remarkable example of Pd-catalyzed allylic amination is its use for the synthesis of α,α-disubstituted *N*-alkyl/aryl allyl amines (Scheme 12a).^82^ With the appropriate monophosphoramidite ligand (*S*^ax^*,S*,S)-**L11**, Kleij’s group achieved high regio- and enantioselectivities (up to 66/1, up to 97% *ee*, respectively) in the amination of a broad selection of α,α-disubstituted allylic carbonates with a wide range of primary alkyl amines. Notably, the reaction also worked well with anilines, which are less reactive, providing the corresponding α,α-disubstituted *N*-aryl allyl amines. The authors also demonstrated the synthetic potential of the resulting products by transforming them into enantioenriched amides, epoxides, allylic nitrones and functionalized aziridines.

The same research group also used the Pd/(*S*^ax^*,S*,S)-**L11** catalyst in the highly enantioselective amination of vinyl cyclic carbonates with a range of anilines for the synthesis of chiral α,α-disubstituted allylic *N*-aryl amines (*ee* values up to 97%; Scheme 12b).^83^ Again, the allylation products could be transformed into a variety of compounds such as chiral ethers, oxazolidinones, diamines and carbamates.

**Scheme 13:**
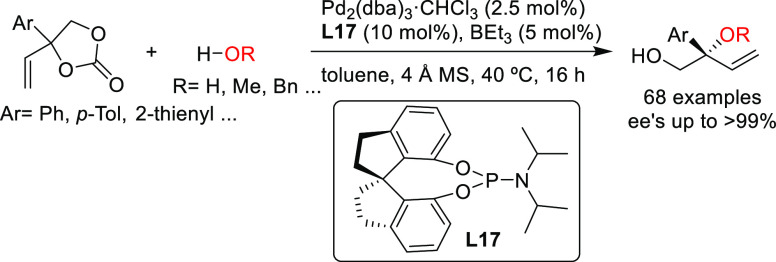
Pd-Catalyzed Allylic Substitution of Vinylethylene Carbonates with Water and Alcohols Using Pd/**L17** Catalyst

Starting from the same substrate class (aryl-substituted vinylethylene carbonates), the group of Zhang described the highly regio-and enantioselective allylic substitution with water and several alcohols via cooperative B/Pd catalysis, (up to >99% *ee*; Scheme 13) to afford tertiary alcohols and ethers.^84^ The catalytic system, formed in situ by mixing the Pd/**L17** complex and triethyl borane, is a boronate complex, which stabilizes the zwitterionic Pd η^3^-allyl intermediate. More recently, the same authors expanded their work to diols, which could be converted into mono- and bisetherified polyglycol derivatives with complete regioselectivity and excellent enantio- and diastereoselectivities (up to >99% *ee* and up to >20/1 dr).^85^

**Scheme 14:**
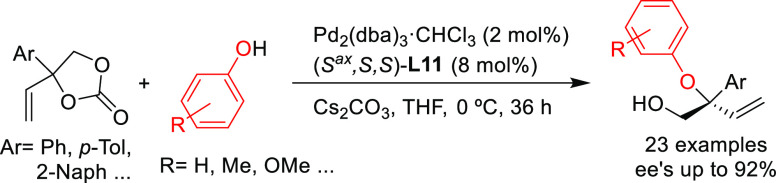
Pd-Catalyzed Allylic Substitution of Vinylethylene Carbonates with Phenols Using Pd/(*S*^ax^,*S*,*S*)-**L11** Catalyst

Kleij’s group developed a similar strategy in which the zwitterionic Pd η^3^-allyl intermediate is stabilized by a metal instead of boron.^86^ They obtained a range of tertiary allylic aryl ethers with high enantioselectivities (up to 92% *ee*) using Pd-(*S*^ax^,*S*,*S*)-**L11** as catalyst (Scheme 14).

**Scheme 15:**
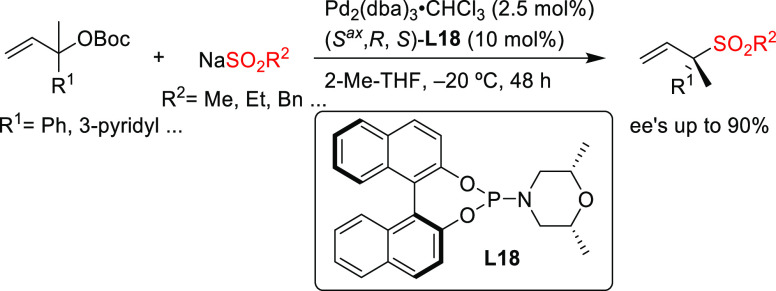
Regio- and Enantioselective Synthesis of Chiral α,α-Disubstituted Allylic Sulfones

More recently, Kleij’s group also developed an efficient method for the synthesis of α,α-disubstituted allylic sulfones from a range of allyl carbonates and sodium sulfinates using Pd/**L18** as catalyst (Scheme 15).^87^ The development of the new phosphoramidite ligand **L18** proved to be crucial in achieving both high regio- and enantiocontrol. This ligand optimization study illustrated the delicate balance between the location of the steric impediment and its influence on the reaction outcome. In addition, the authors demonstrated the utility of their method by synthesizing the sesquiterpene (−)-agelasidine A (see section 2.5).

**Scheme 16:**
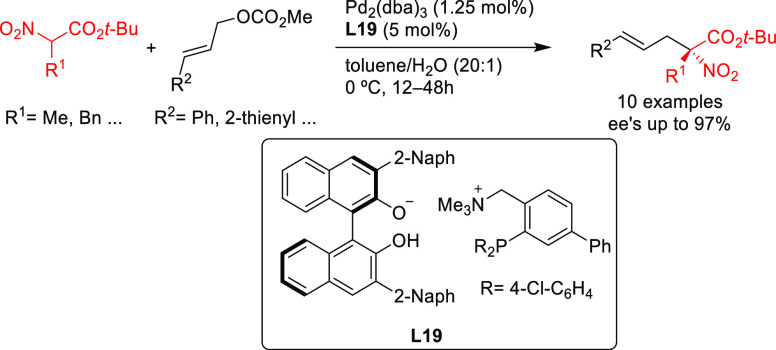
Pd-Catalyzed Allylic Alkylation of Cinnamyl-Type Carbonates with α-Nitrocarboxylates

Finally, an interesting new design concept for monodentate ligands design was introduced by Ooi and co-workers. They developed an achiral cationic ammonium-phosphine hybrid ligand paired with a chiral binaphtholate anion.^88^ They found that the Pd-catalyst derived from the binaphtholate-based ligand **L19** provided high enantioselectivities in the allylic alkylation of challenging cinnamyl-type carbonates with α-nitrocarboxylates (up to 97% *ee*; Scheme 16).

**Scheme 17:**
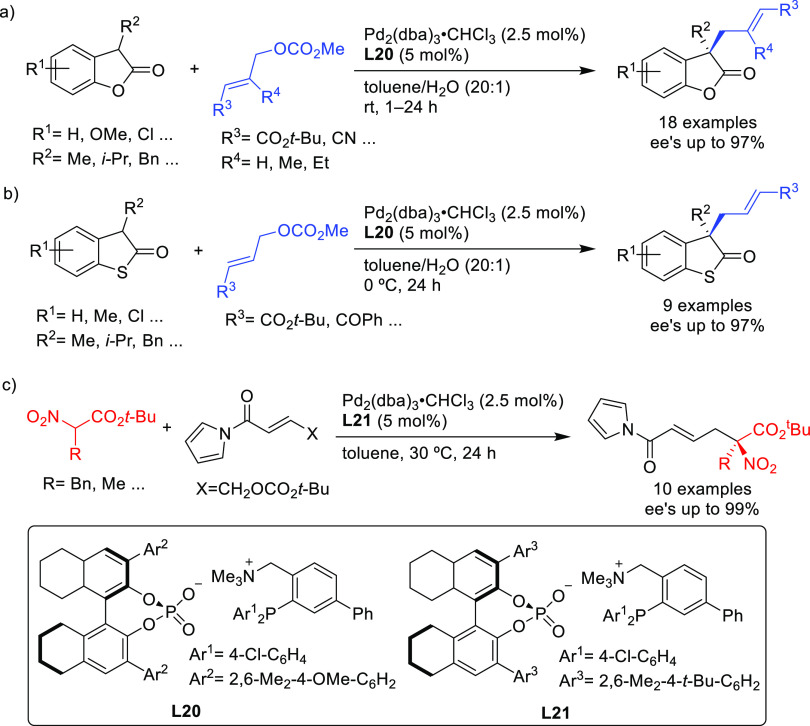
Representative Pd-Catalyzed Allylation Using Ammonium-Phosphine Hybrid Ligand Paired with a Chiral Phosphate Anion

Subsequently, this type of ligand was further modified by replacing the binaphtholate by a binaphthol-based phosphate anion.^89−92^ A highly enantioselective allylation of α-substituted benzofuran-2(3*H*)-ones with functionalized allylic carbonates was achieved using Pd/**L20** as catalyst (up to 97% *ee*; Scheme 17a).^89,90^ This approach was further extended to the allylation of α-substituted benzothiophenones (*ee* values up to 97% using Pd/**L20** as catalyst; Scheme 17b)^91^ and of α-nitrocarboxylates (*ee* values up to 99% using Pd/**L21** as catalyst; Scheme 17c).^92^

#### 2.2.2 Bidentate Homodonor P,P-Ligands

##### 2.2.2.1 Applications of Trost Diphosphine Ligands

**Scheme 18:**
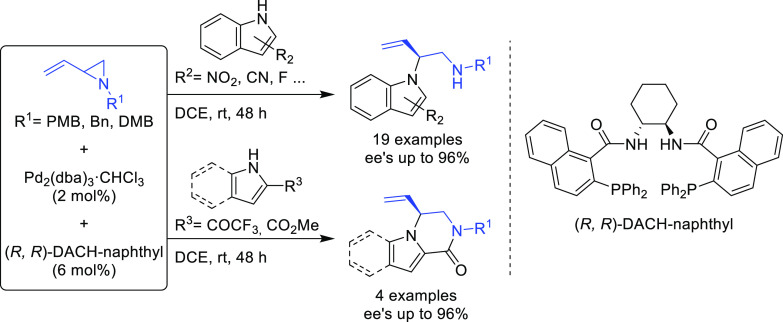
Pd-Catalyzed DYKAT of Vinyl Aziridines Using (*R,R*)-DACH-naphthyl Trost Ligand

Important new applications of Pd-catalyzed allylic substitution of Trost diphosphine ligands and some specific variations of them have been reported by the Trost group.^8,15,18,24^ A notable example is the Pd-catalyzed dynamic kinetic asymmetric transformation (DYKAT) of vinyl aziridines, with both substituted 1*H*-pyrroles and 1*H*-indoles, to obtain exclusively the *N*-alkylated branched products in high yields and enantioselectivities (*ee* values up to 96%; Scheme 18).^93^ No electron-withdrawing groups on the vinyl aziridine and electron-withdrawing groups on the *N*-heterocycle were needed for the reaction to work. Moreover, many types of functional groups are tolerated in the *N*-heterocyclic nucleophile. This methodology was also applied to the synthesis of pharmaceuticals and biologically active natural products, such as longamide B, longamide B methyl ester, hanishin, agesamides A and B, and cyclooroidin.

**Scheme 19:**
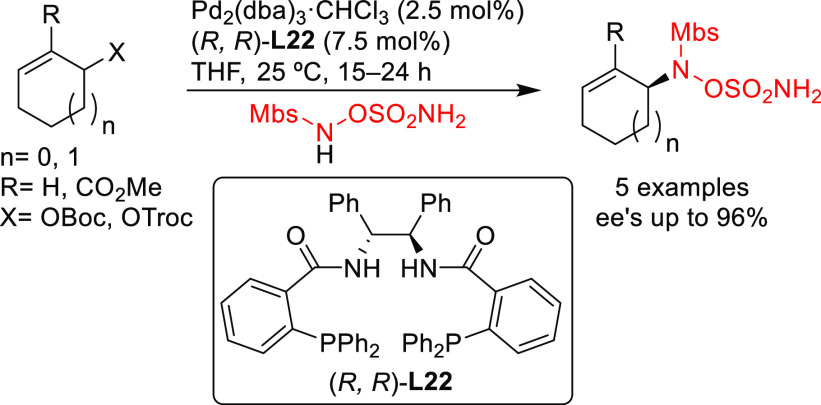
Pd-Catalyzed Allylic Amination of Cyclic Allylic Carbonates with 4-Methoxy-*N*-(sulfamoyloxy)benzenesulfonamide Using Pd/(*R,R*)-**L22** as Catalyst

Trost and co-workers also reported a modification of their ligand with an (*R*,*R*)-1,2-diphenylethane 1,2-diamine bridge fragment. Ligand (*R*,*R*)-**L22** was successfully used in the Pd-catalyzed amination of 5- and 6-membered ring allylic carbonates, with 4-methoxy-*N*-(sulfamoyloxy)benzenesulfonamide as nucleophile (*ee* values up to 96%; Scheme 19).^94^ The asymmetric desymmetrization of *meso*-di-*tert*-butyl cyclohex-2-ene-1,4-diyl bis(carbonate) also provided the monosubstituted product in high yield and with enantioselectivities of up to 95% *ee*. Acyclic substrates (methyl pent-3-en-2-yl carbonate and butadiene monoepoxide) were also coupled efficiently with MbsNHOSO_2_NH_2_ (95% *ee* and 94% *ee*, respectively) [Mbs= 4-methoxy-benzenesulfonamide].

**Scheme 20:**
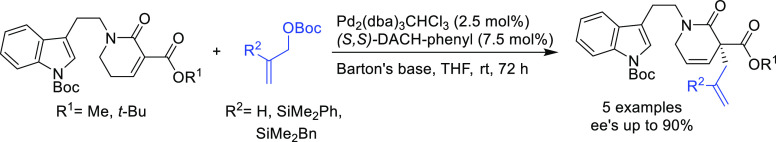
Pd-Catalyzed Allylic Alkylation with Indole-Containing *N*-Alkyl Lactams

A further application of the Pd/(*R,R*)-DACH-phenyl catalyst is shown in Scheme 20. Using indoles with a pendant lactam ring at the 3-position as nucleophiles, monoterpene indole alkaloids are accessible with high enantioselectivity.^95^

**Scheme 21:**
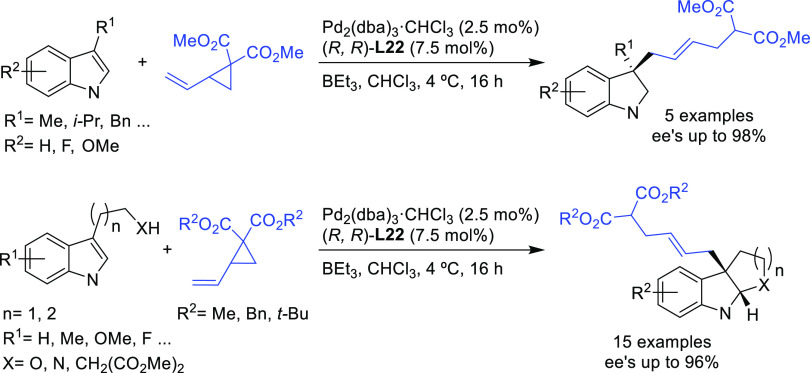
Pd-Catalyzed Allylic Alkylation of 3-Substituted Indoles and Tryptophan Derivatives with Vinylcyclopropanes Using Pd/(*R*,*R*)-**L22** Catalytic System

In a recent related study, Trost’s group reported the allylic alkylation of vinylcyclopropanes with 3-substituted indoles and tryptophan derivatives using the modified Trost ligand (*R*,*R*)-**L22**.^96^ A broad range of 3,3-disubstituted indolenines and indolines were synthesized with excellent enantioselectivies (*ee* values up to 98%; Scheme 21).

**Scheme 22:**
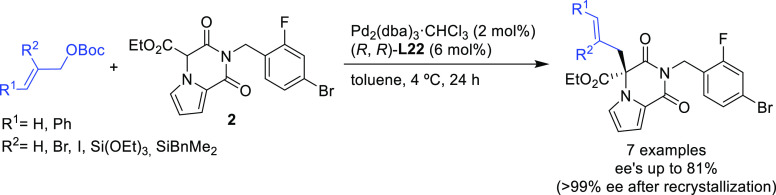
Pd-Catalyzed Allylic Alkylation of Allyl *tert*-Butyl Carbonates with Amidomalonate **2**

Another interesting example using the modified (*R*,*R*)-**L22** Trost ligand is the allylic alkylation of allyl *tert*-butyl carbonates with amidomalonate **2** (Scheme 22).^97^ This strategy was used to synthesize (−)-ranirestat, an aldolase reductase inhibitor (see section 2.5).

**Scheme 23:**
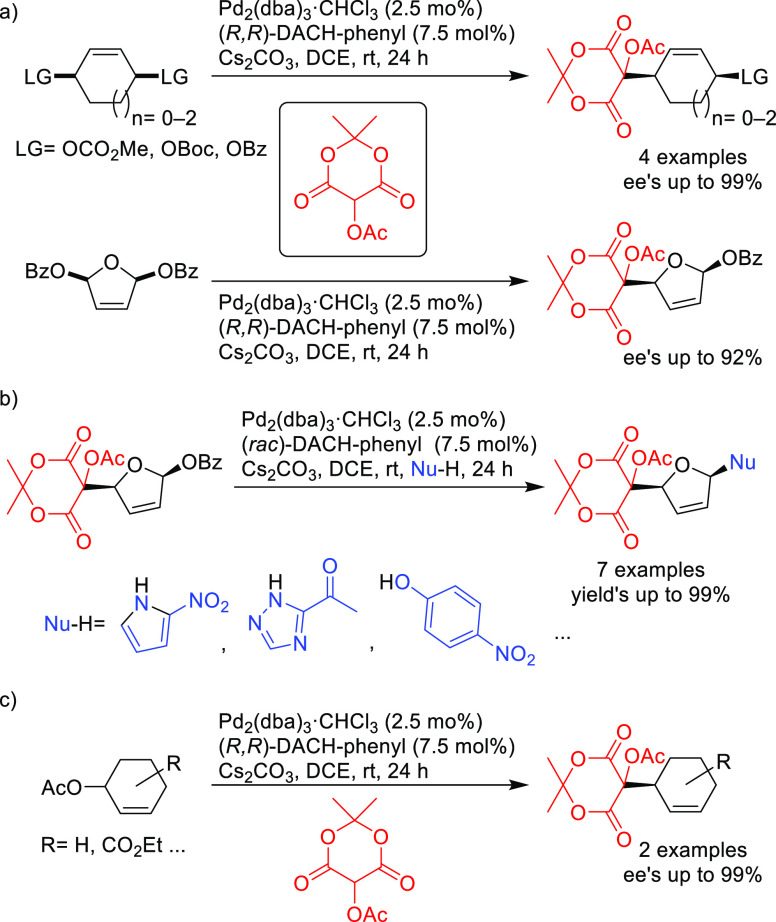
Pd-Catalyzed Allylic Substitution of (a) *meso*- and Racemic Cyclic Substrates with Acetoxy Meldrum’s Acid Derivatives, (b) Subsequent Allylic Substitution with N- and O-Nucleophiles, and (c) Cyclohexenyl Acetates with Acetoxy Meldrum’s Acid Derivatives

Trost’s group also demonstrated that acetoxy Meldrum’s acid can be used as a versatile acyl anion equivalent in the Pd-catalyzed allylic alkylation of *meso*- and racemic cyclic substrates.^98^ Thus, 5- to 7-membered ring *meso*-substrates were desymmetrized with high enantioselectivity using the Pd/(*R*,*R*)-DACH-phenyl catalyst (*ee* values up to 99%; Scheme 23a). The resulting compounds were then converted to a variety of products by a second allylic substitution with several N- and O-nucleophiles using Pd/(*rac*)-DACH-phenylas catalyst (Scheme 23b). Excellent enantioselectivities (*ee* values up to 99%) were also achieved in the allylic alkylation of some cyclohexenyl acetates (Scheme 23c).

**Scheme 24:**
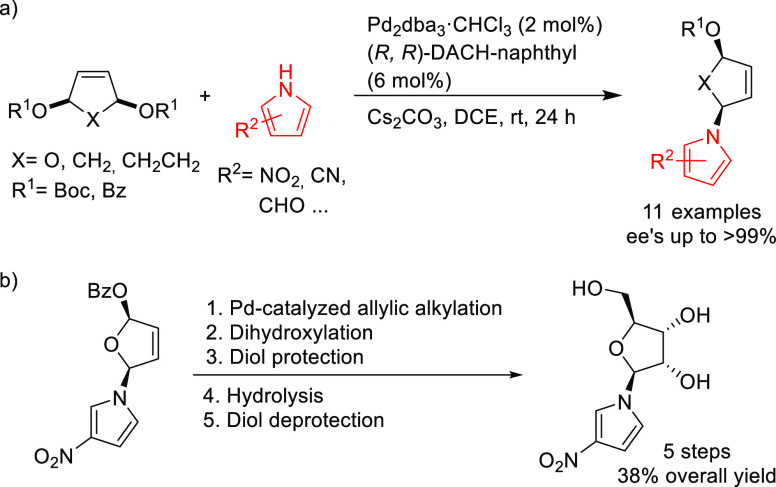
Pd-Catalyzed Desymmetrization of 5- and 6-Membered Ring Cyclic *meso*-Substrates with Electron-Deficient Pyrroles

This approach was also successfully extended to the desymmetrization of 5- and 6-membered ring cyclic *meso*-substrates with electron-deficient pyrroles using the (*R,R*)-DACH-naphthyl Trost ligand. The products were obtained with perfect regio- and diastereoselectivity and excellent enantioselectivities (up to >99% *ee*; Scheme 24a).^99^ This strategy was employed for the synthesis of a pyrrole-substituted ribonucleoside analogue in five steps and 38% overall yield from the primary allylation (Scheme 24b).

**Scheme 25:**
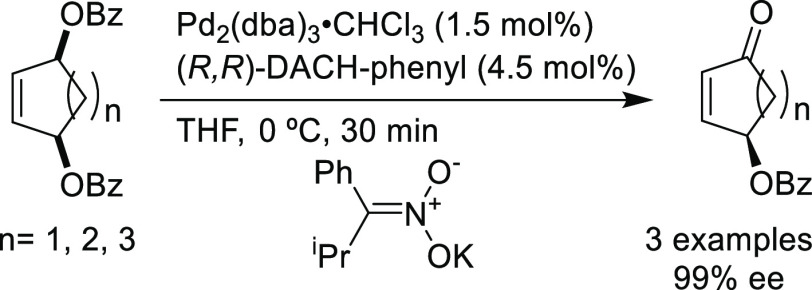
Pd-Catalyzed Oxidative Desymmetrization of Cyclic *meso*-Dibenzoates Using Pd/(*R,R*)-DACH-phenyl as Catalyst

The Pd/(*R,R*)-DACH-phenyl complex also proved to be an efficient catalyst for the oxidative desymmetrization of cyclic *meso*-dibenzoates (Scheme 25).^100^ The resulting chiral cycloalkenones served as building blocks for the synthesis of epoxyquinoid natural products.

**Scheme 26:**
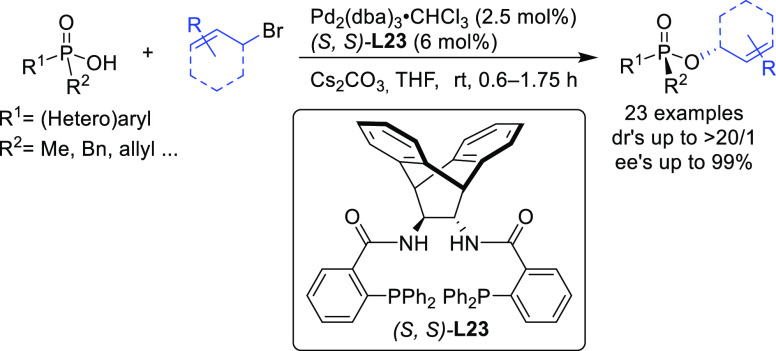
Desymmetrization of Phosphinic Acids via Pd-Catalyzed Allylic Substitution

Trost’s group has also reported an efficient desymmetrization of phosphinic acids with an interesting modification of the Trost ligand, the (*S,S*)-diaminoethanoanthracene-based ligand **L23** (Scheme 26).^101^ The Pd/(*S,S*)-**L23** catalyst was able to discriminate between the two enantiotopic oxygen atoms providing a novel synthetic path to *P*-chiral phosphinates with high diastereo- and enantioselectivities (Scheme 26).

**Scheme 27:**
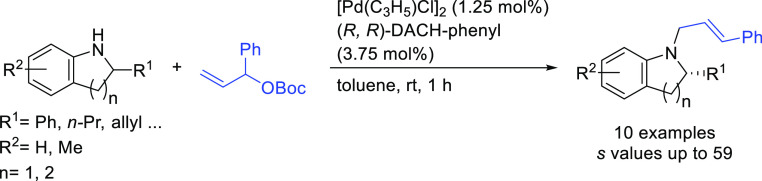
Pd-Catalyzed Kinetic Resolution of Indolines with Pd/(*R,R*)-DACH-phenyl as Catalyst

Notable applications of Trost-type ligands were also reported by other research groups. Scheme 27 highlights the work of Hou and co-workers from 2009 on the kinetic resolution of racemic indolines via the Pd-catalyzed allylic amination of the *tert*-butyl(1-phenylallyl) carbonate (Scheme 27). Using the Pd/(*R,R*)-DACH-phenyl catalyst, enantioenriched indolines and allylic indolines were produced in moderate-to-high enantiomeric excesses (36–94% *ee* for indolines and 51–92% *ee* for allylic indolines; *s* values up to 59).^102^

**Scheme 28:**
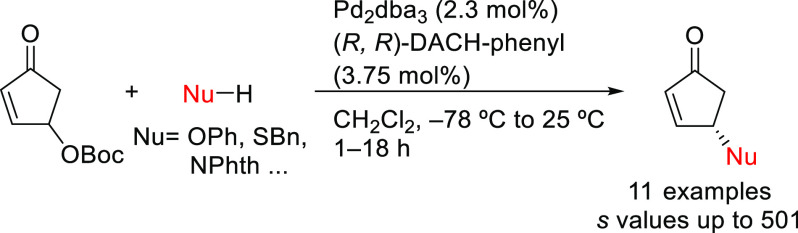
Pd-Catalyzed Kinetic Resolution *O*-Boc Protected 4-Hydroxycyclopentenone with Pd/(*R,R*)-DACH-phenyl as Catalyst

In 2013, Reiser and co-workers employed the Pd/(*R,R*)-DACH-phenyl catalyst for the kinetic resolution of *O*-Boc protected 4-hydroxycyclopentenone, a versatile intermediate that can be readily accessed from furfuryl alcohol in two steps, with a range of N-, O-, and S-nucleophiles (Scheme 28).^103^ This protocol gave rise to enantioenriched cyclopentenones with moderate-to-excellent selectivity factors (*s* values up to 501). By this approach, a key intermediate for the synthesis of the enantiomer of the antiviral and antitumor drug noraristeromycin was prepared.

**Scheme 29:**
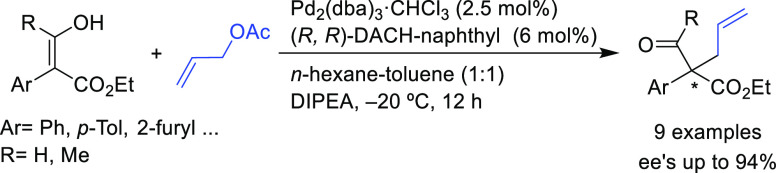
Pd-Catalyzed Allylation of Hydroxyacrylates Using Pd/(*R*,*R*)-DACH-naphthyl as Catalyst

Hossain’s group successfully employed hydroxyacrylates as nucleophiles instead of the commonly used ketoesters.^104,105^ The reaction yielded a range of enantioenriched α-aryl quaternary carbonyl compounds in high *ee* values (up to 94%; Scheme 29) using the Pd/(*R*,*R*)-DACH-naphthyl catalyst. The same group also developed an intramolecular version using the corresponding allyl enol ethers to yield α-aryl quaternary aldehydes in *ee* values of up to 90%.^106^

**Scheme 30:**
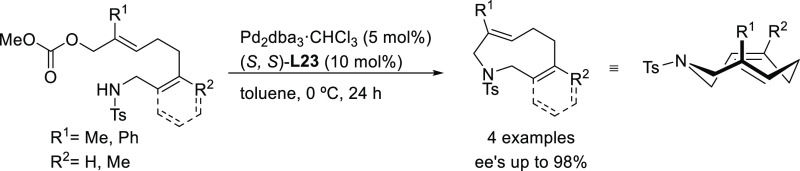
Synthesis of 9-Membered Cyclic Amides Bearing Planar Chirality via Pd-Catalyzed Asymmetric Allylic Cyclization Using Pd/(*S*,*S*)-**L23** as Catalyst

Tomooka and co-workers described the enantioselective synthesis of nine-membered cyclic amides with planar chirality via Pd-catalyzed allylic cyclization of achiral allylic carbonates using the diaminoethanoanthracene-based Pd/(*S*,*S*)-**L23** catalyst (Scheme 30).^107^ The reaction proceeded in moderate-to-good yields, and generally excellent enantioselectivities with substituted allylic carbonates (R^1^ ≠ H; *ee* values up to 98%) whereas unsubstituted derivatives (R^1^ = H) gave lower *ee* values of up to 66%.

**Scheme 31:**
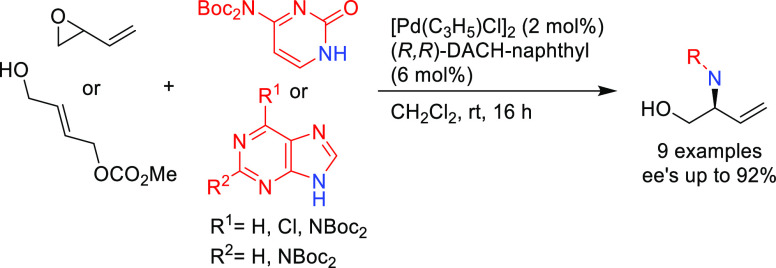
Pd-Catalyzed Allylic Amination of 2-Vinyloxirane or 4-Hydroxybut-2-en-1-yl Methyl Carbonate with Pyrimidinic and Purinic Bases

In 2015, Díaz, Castillón and co-workers demonstrated that the Pd/(*R*,*R*)-DACH-naphthyl complex is an efficient catalyst for the allylic amination of the 2-vinyloxirane and the 4-hydroxybut-2-en-1-yl methyl carbonate with pyrimidinic and purinic bases (*ee* values up to 92%; Scheme 31).^108^ The resulting amines were converted to a range of acyclic nucleoside phosphonates.

**Scheme 32:**
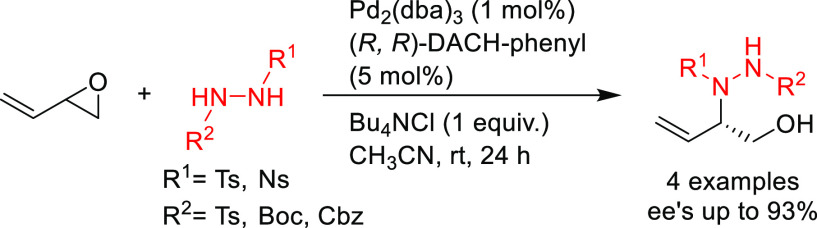
Pd-Catalyzed Allylic Amination of 2-Vinyloxirane with 1,2-Disubstituted Hydrazines

Similarly, Shipman and co-workers described the asymmetric allylic amination of 2-vinyloxirane with several 1,3-disubstituted hydrazines, providing allylic hydroxy-hydrazines in high enantioselectivities (up to 93% *ee*) with the Pd/(*R*,*R*)-DACH-naphthyl catalyst (Scheme 32).^109^ These products were demonstrated to be versatile precursors for synthetically useful transformations such as the cyclization to diazetidines or the conversion of the alkene into an amine.

**Scheme 33:**
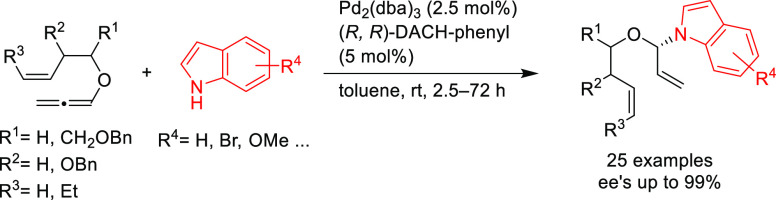
Pd-Catalyzed *N*-Selective Addition Reaction of Indoles to Alkoxyallenes

In 2018, Rhee and co-workers developed the asymmetric addition of a range of indoles to alkoxyallenes that proceeds through Pd η^3^-allyl intermediates with Pd/(*R*,*R*)-DACH-phenyl as catalyst (Scheme 33).^110^ This method is fully regioselective and gives rise to enantioenriched dienes (*ee* values up to 99%). The potential of this reaction was demonstrated with the highly efficient synthesis of chiral *N*-glycosylindoles via ring-closing metathesis of the dienes.

**Scheme 34:**
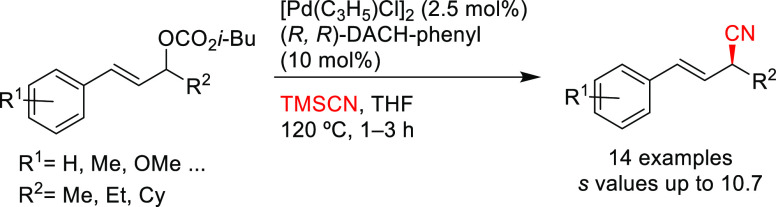
Pd-Catalyzed Kinetic Resolution of Allyl Carbonates with Trimethylsilyl Cyanide

Hou’s group also disclosed a kinetic resolution of unsymmetrical acyclic allyl carbonates with trimethylsilyl cyanide using Pd/(*R*,*R*)-DACH-phenyl as catalyst(*s* values up to 10.7; Scheme 34).^111^

**Scheme 35:**
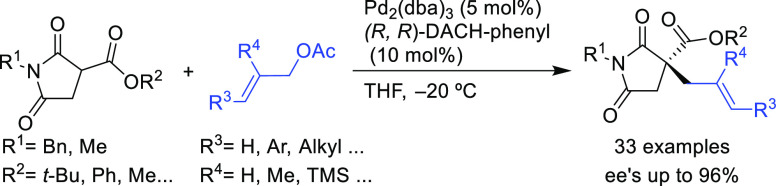
Synthesis of α-Quaternary Succinimides via Pd-Catalyzed Allylic Alkylation

Cossy’s group successfully employed the Trost catalyst (Pd/(*R*,*R*)-DACH-phenyl) in the allylation of succinimide derivatives (Scheme 35; *ee* values up to 96%).^112^ This reaction gives access to a variety of α-quaternary succinimides, motifs which are present in many natural products and pharmaceuticals.

**Scheme 36:**
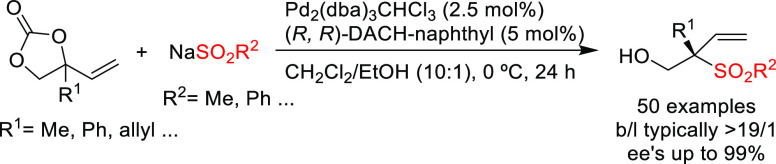
Pd-Catalyzed Allylic Sulfonylation of Vinyl Cyclic Carbonates Using Pd/(*R,R*)-DACH-naphthyl as Catalyst

Khan’s and Zhao’s group reported another example of the use of Trost-type ligands, in this case (*R,R*)-DACH-naphthyl, in the Pd-catalyzed allylic sulfonylation of vinyl cyclic carbonates with sodium sulfinates (Scheme 36).^113^ A broad range of sulfone-containing compounds bearing a tetrasubstituted carbon stereocenter was synthesized with excellent regioselectivities favoring the branched isomer and high *ee* values.

**Scheme 37:**
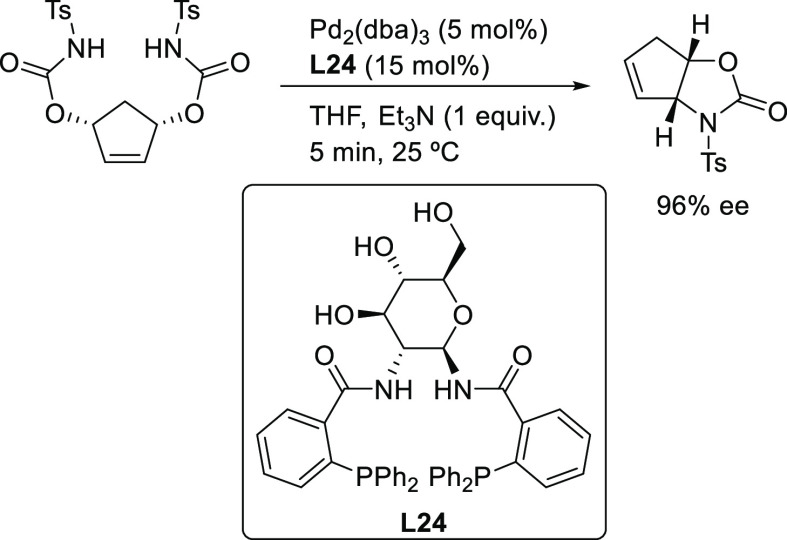
Pd-Catalyzed Desymmetrization of *meso*-Cyclopent-4-ene-1,3-diyl Bis(Tosylcarbamate) Using Pd/**L24** as Catalyst

Finally, a remarkable modification of the Trost ligand was reported by Ruffo and co-workers with the diphosphine ligand **L24**, in which the 1,2-diaminocyclohexane backbone was replaced by a β-1,2-d-glucodiamine (Scheme 37).^114^ The Pd/**L24** catalyst was successfully used in the desymmetrization of *meso*-cyclopent-4-ene-1,3-diyl bis(tosylcarbamate) through an intramolecular allylic substitution, affording the (3*R*, 6*S*)-3-tosyl-3,3a,6,6a-tetrahydro-2H-cyclopenta[d]oxazol-2-one in quantitative yield and very high enantiomeric excess (96% *ee*) in short reaction times (5 min). Notably, the catalyst could be recycled using bmpyBF_4_ as solvent.

##### 2.2.2.2 Application of Other Diphosphine Ligands

**Scheme 38:**
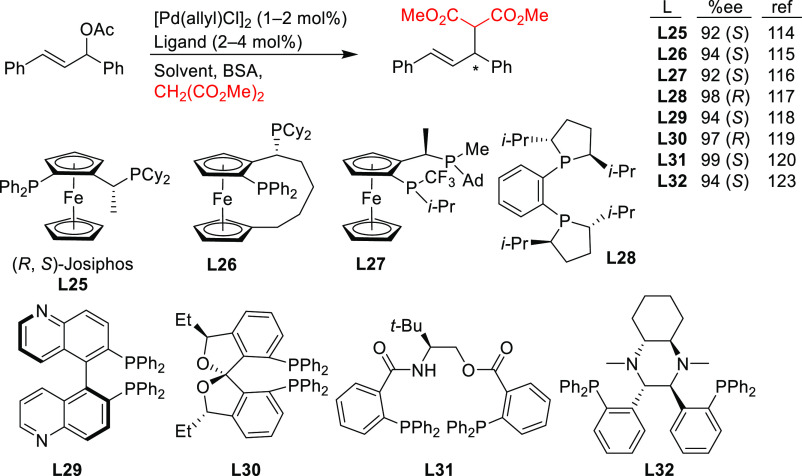
Representative Examples of Diphosphine Ligands Applied in the Pd-Catalyzed AAA of *rac*-1,3-Diphenylallyl Acetate Using Dimethyl Malonate as Nucleophile

Since 2008 other diphosphines as well were used, although only a few of them provided high enantioselectivities. Scheme 38 collects the most representative ligand families evaluated in the allylic alkylation of the model substrate *rac*-1,3-diphenylallyl acetate with dimethyl malonate as nucleophile. In particular, Josiphos-type^115−117^ and DuPHOS-type^118^ diphosphines (**L25**–**L28**) provided *ee* values of up to 98%. Similarly high levels of enantioselectivity (up to 97% *ee*) were also obtained with chiral biquinolyl-^119^ and spiroketal-based^120^ diphosphine ligands **L29** and **L30** (Scheme 38).

**Scheme 39:**
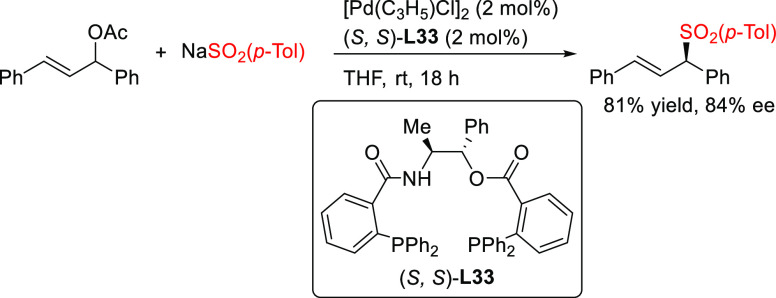
Pd-Catalyzed Allylic Sulfonylation of the *rac*-1,3-Diphenylallyl Acetate with Sodium *p*-Toluenesulfinate Using Pd/(*S*,*S*)-**L33** Catalyst

As previously mentioned, the Trost type ligands are not well suited for the alkylation of hindered substrates, such as *rac*-1,3-diphenylallyl acetate. To overcome this problem, the group of Hitchcock replaced one of the amido groups in the Trost ligand by an ester group. As a result the *tert*-leucinol-derived diphosphine **L31** provided excellent *ee* values (up to 99%) in the allylic alkylation of *rac*-1,3-diphenylallyl acetate (Scheme 38).^121,122^ The performance of ligand **L31** was rationalized by the Lloyd-Jones/Norrby model in which the nucleophilic attack is assisted through a hydrogen bond with the amido group of the ligand (see section 2.4).^122^ As a further modification, β-(*o*-diphenylphosphino)benzoyloxy (*o*-diphenylphosphino)benzamide (*S*,*S*)-**L33** was reported, which was used in the allylic sulfonylation of *rac*-1,3-diphenylallyl acetate with sodium *p*-toluenesulfinate (Scheme 39) and the allylic alkylation of the same substrate with dimethyl malonate (*ee* values up to 84%).^123^

**Scheme 40:**
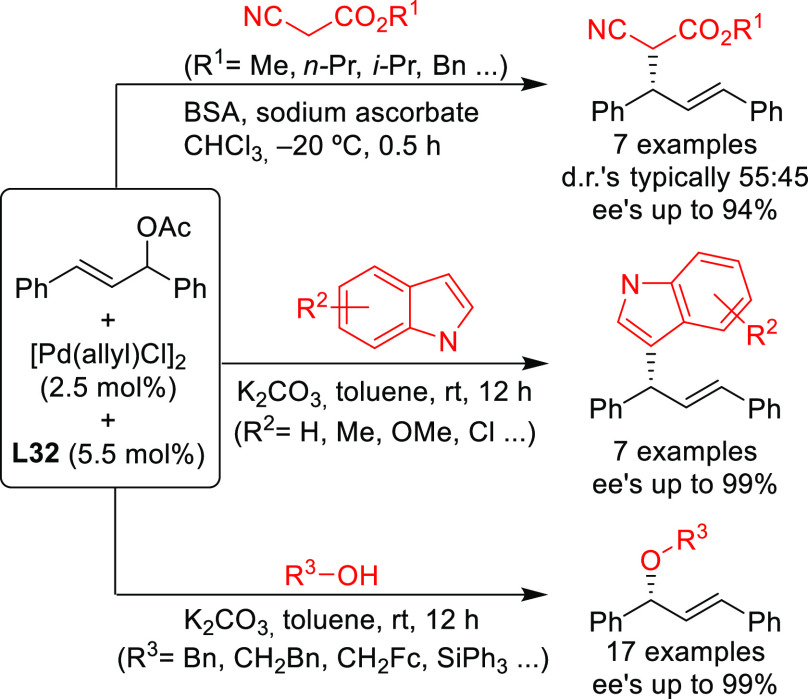
Pd-Catalyzed Asymmetric Allylic Substitution of 1,3-Diphenylallyl Acetate Using a Range of (a) 2-Cyanoesters, (b) Indoles, and (c) Alkylic Alcohols Using Pd/**L32** as Catalyst

On the basis of a similar design, Xu and co-workers developed a *trans*-1,2-diaminocycloxane-derived diphosphine ligand (Fei-Phos ligand **L32**), which gave high enantioselectivities in the allylic alkylation of *rac*-1,3-diphenylallyl acetate with several malonates (*ee* values up to 94%; Scheme 38).^124,125^ The Fei-Phos ligand was also successfully employed in allylic substitutions with a variety of other C-, O- and N-nucleophiles. For example, high enantioselectivities were obtained in the alkylation of 1,3-diphenylallyl acetate with C-nucleophiles such as 2-cyanoacetates and indoles (Scheme 40; *ee* values up to 94% for 2-cyanoacetates and up to 99% for indoles).^124^ Similarly high *ee* values were achieved with alkyl alcohols and silanols (Scheme 40).^125^ Amines were also used as nucleophiles but high enantioselectivities were only achieved with anilines (up to 86%), while alkyl amines were either unreactive or provided low *ee* values.^124^ The authors proposed that the key for the enantioselectivity induced by the Pd/**L32** catalyst is a hydrogen bond between the nucleophile and the amino group of the ligand that directs nucleophilic attack.^126^

**Scheme 41:**
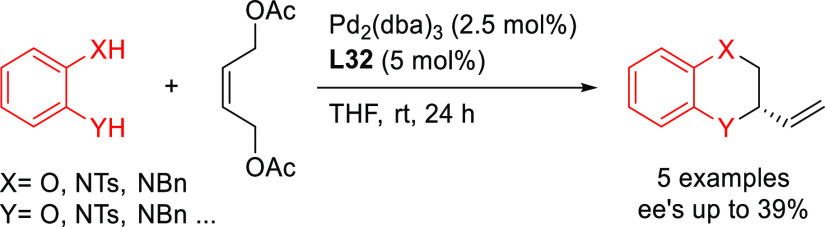
Synthesis of 2-Vinyl-2,4-dihydro-benzo[1,4]dioxin, Oxazine, and Diazine Compounds

Recently, the same group studied another application of the Fei-Phos ligand, although with less success. They reported the synthesis of 2-vinyl-2,4-dihydro-benzo[1,4]dioxin, oxazine and diazine products through a tandem Pd-catalyzed allylic substitution of (*Z*)-but-2-ene-1,4-diacetate with 1,2-bifunctional nucleophiles and subsequent cyclization.^127^ However, the enantioselectivities of the five reactions investigated were low (*ee* values up to 39%; Scheme 41).

**Scheme 42:**
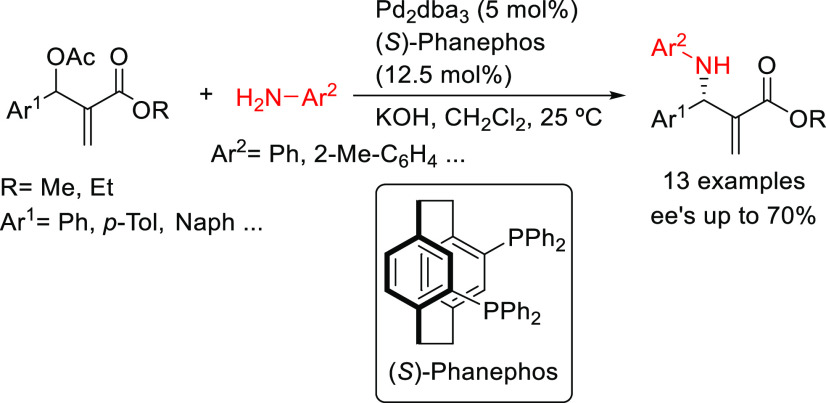
Pd-Catalyzed Allylic Amination of Acetylated Morita–Baylis–Hillman Products Using Pd/(*S*)-Phanephos as Catalyst

Some noteworthy studies have been published on the use different diphosphine ligands for substrates other than the model *rac*-1,3-diphenylallyl acetate. One example is the application of the Walphos ligand in the allylic alkylation of cyclohexenyl acetate with dimethyl malonate (*ee* values up to 98%).^128^ Another example is the Pd-catalyzed allylic amination of acetylated Morita–Baylis–Hillman products with a range of aromatic amines (Scheme 42).^129^ The use of Pd/(*S*)-Phanephos as catalyst yielded the corresponding unsaturated amino-esters in moderate enantioselectivities (*ee* values up to 70%) and good regioselectivities in favor of the desired branched product (typically >15:1).

**Scheme 43:**
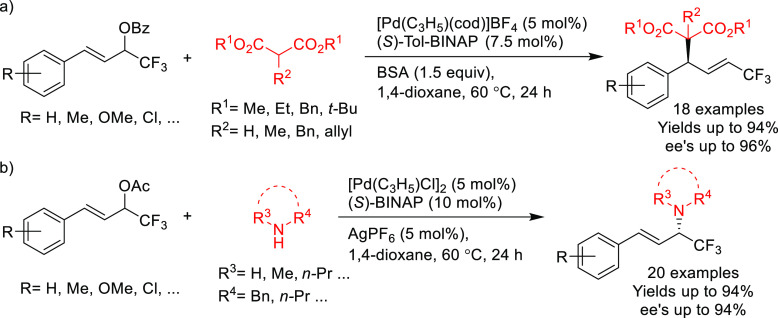
Pd-Catalyzed Asymmetric Allylic Substitution of Unsymmetrical 1,3-Disubstituted Allylic Substrates with (a) Malonates and (b) Amines via DYKAT

Other examples involve BINAP-type ligands for the allylic substitution of unsymmetrical 1,3-disubstituted allylic systems via dynamic kinetic asymmetric transformations (DYKAT). A range of 1,1,1-trifluoro-4-arylbut-3-en-2-yl benzoates was efficiently deracemized with a variety of malonates using the [Pd(C_3_H_5_)(cod)]BF_4_/(*S*)-Tol-BINAP catalytic system (Scheme 43a).^54^ Similarly, (*S*)-BINAP was used in the Pd-catalyzed allylic amination of 1,1,1-trifluoro-4-arylbut-3-en-2-yl acetates via DYKAT (Scheme 43b).^53^ The use of Pd/(*S*)-BINAP/AgPF_6_ as catalyst led to the corresponding amines in high yields, high regioselectivities in favor of the α-product and high enantioselectivities (up to 94% *ee*). The use of a silver cocatalyst proved to be key for the α-selectivity of this process, and its removal switched the regioselectivity toward the γ-isomer with good selectivity (92:8, γ:α).

**Scheme 44:**
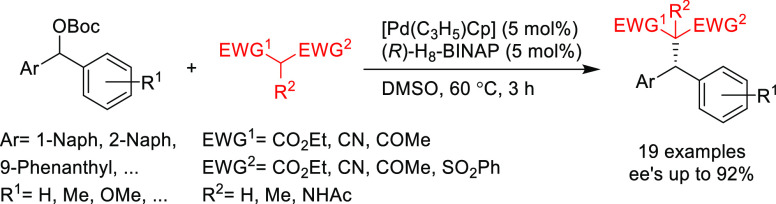
Pd-Catalyzed Benzylic Alkylation of Diarylmethyl Carbonates via DYKAT

Hirano and Miura and co-workers developed an asymmetric benzylic alkylation via DYKAT with the Pd/(*R*)-H_8_-BINAP catalyst without the use of an external base.^130^ A range of racemic diarylmethyl carbonates was converted to chiral products containing a chiral benzylic stereocenter using different C-nucleophiles, such as malonates, 1,3-diketones, malononitrile, β-ketoesters, 2-cyanoesters, and β-sulfonylesters (Scheme 44). Moreover, with the addition of carbonate bases, the Pd/(*R*)-H_8_-BINAP system was also able to achieve an effective DYKAT of the corresponding pivalates.

**Scheme 45:**
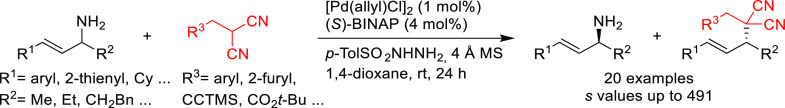
Pd-Catalyzed KR of Unsymmetrical Disubstituted Primary Allylic Amines with Malononitriles Using Pd/(*S*)-BINAP

Another example of the use of BINAP-type ligands is the kinetic resolution of unsymmetrically disubstituted primary allylic amines via Pd-catalyzed allylic alkylation of malononitriles (Scheme 45).^131^ The reaction enabled the asymmetric synthesis of α-branched allyl-substituted malononitriles with high selectivity (*s* factors up to 491). The reaction is accelerated in the presence of mesityl sulfonyl hydrazide.

**Scheme 46:**
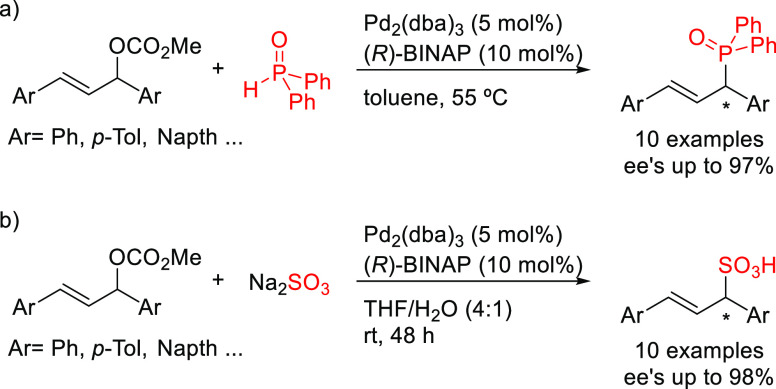
Pd-Catalyzed Allylic Substitution of 1,3-Diaryl-Substituted Allylic Carbonates with (a) Diphenylphosphine Oxide and (b) Sodium Sulfite Using Pd/(*R*)-BINAP as Catalyst

Another notable example of the application of BINAP was reported by Zhao and co-workers studying the Pd-catalyzed allylic substitution of 1,3-diaryl-substituted allylic carbonates with diphenylphosphine oxide as P-nucleophile (Scheme 46a).^132^ The desired diphenylphosphine oxides were obtained in good-to-high enantioselectivities (*ee* values up to 97%). The absolute configuration of the products was not determined. Unfortunately, other P-nucleophiles, such as diisopropyl phosphonate, failed in this reaction. Moreover, the use of monosubstituted linear allylic carbonates exclusively led to achiral linear allylic phosphonates in good yields. The same group subsequently reported the use of Pd/(*R*)-BINAP as catalyst in the allylic substitution of 1,3-diaryl-substituted allylic acetates with sodium sulfite as a sulfur-based nucleophile (Scheme 46b).^133^ A range of allylic sulfonic acids was synthesized with high enantioselectivities (*ee* values up to 98%). The absolute configuration of the products was not determined. In this transformation, the use of water as a cosolvent was a key factor for achieving the desired reactivity.

**Scheme 47:**
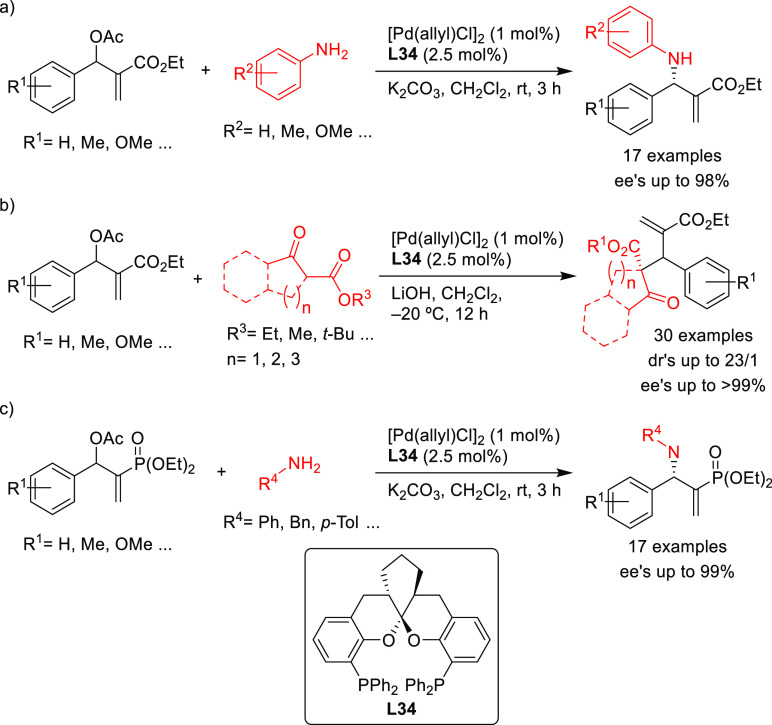
Pd-Catalyzed Allylic Substitution of Morita–Baylis–Hillman Adducts Using Several C- and N-Nucleophiles with the Pd/**L34** Catalytic System

Three applications of a new class of spiroketal-based diphosphine **L34** have also been reported (Scheme 47). Liu, Wang, Ding, and co-workers successfully applied **L34** in the Pd-catalyzed allylic amination of Morita–Baylis–Hillman adducts with a range of anilines (*ee* values up to 98%; Scheme 47a).^134,135^ The resultant optically active β-arylamino acid esters were transformed into the corresponding β-lactam derivatives. The scope of the reaction was subsequently extended to β-ketoesters and β-amidoesters as nucleophiles (Scheme 47b; *ee* values up to >99% and dr’s up to 23/1).^136^ The same group also reported the use of Pd/**L34** catalyst for the asymmetric allylic amination of 2-diethylphosphonate-substituted allylic acetates with primary amines, affording a series of β-aminophosphonates bearing an α-methylene functionality in excellent regio- and enantioselectivities (Scheme 47c; *ee* values up to 99%).^137^

**Scheme 48:**
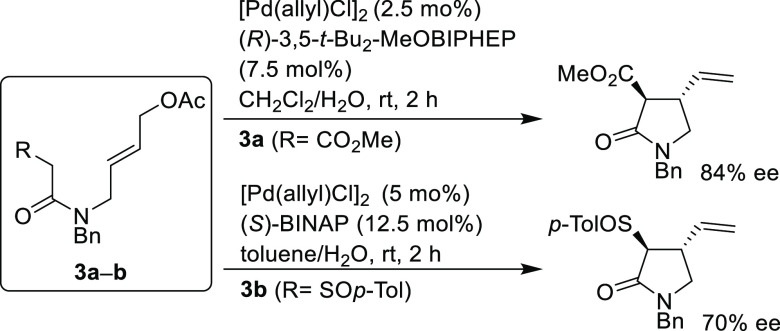
Synthesis of Enantioenriched Disubstituted γ-Lactams Using Pd/(*R*)-3,5-*t*-Bu_2_-MeOBIPHEP and Pd/(*S*)-BINAP

Using either BINAP or a BIPHEP type ligands, Poli’s group developed the intramolecular allylic alkylation of β-amidoester **3a** and β-sulfinylamide **3b** to yield enantioenriched disubstituted γ-lactams with *ee* values of up to 84% and 70%, respectively (Scheme 48).^138,139^

**Scheme 49:**
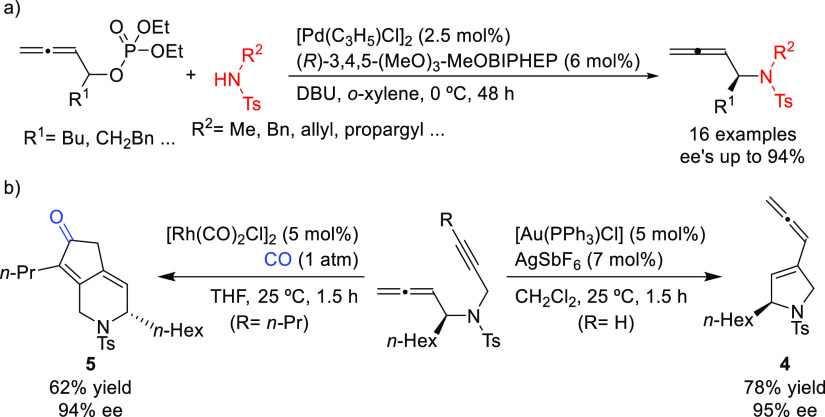
(a) Pd-Catalyzed Allylic Amination of Allenyl Phosphates Using Pd/(*R*)-3,4,5-(MeO)_3_-MeOBIPHEP as Catalyst and (b) Synthesis of Chiral 2,5-Dihydropyrrole Derivative **4** and the Bicyclic Ketone **5**

Ma and co-workers developed a highly enantioselective Pd-catalyzed allylic amination of allenyl phosphates, producing 2,3-allenyl amines. The BIPHEP derivative, ((*R*)-3,4,5-(MeO)_3_-MeOBIPHEP),proved to be a key ligand for this transformation, providing 2,3-allenyl amines with high enantioselectivities (*ee* values up to 94%; Scheme 49a) using DBU as base.^140^ One of the products bearing a propargylic substituent was converted to the enantioenriched 2,5-dihydropyrrole derivative **4** and the bicyclic ketone **5** by cyclization (Scheme 49b).

**Scheme 50:**
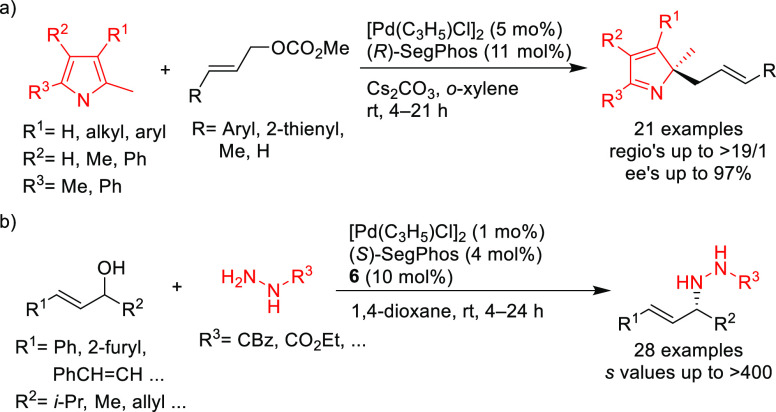
Pd-Catalyzed Allylic Substitution of (a) Pyrroles with Monosubstituted Allylic Carbonates and (b) Unactivated Allylic Alcohols with Monosubstituted Hydrazines Using Pd/SegPhos Catalysts

SegPhos-type ligands are another class of versatile diphosphines with several applications in Pd-catalyzed allylic substitutions of substrates other than the model *rac*-1,3-diphenylallyl acetate. A range of pyrroles was efficiently dearomatized using monosubstituted allylic carbonates with Pd/(*R*)-SegPhos catalysts (Scheme 50a).^141,142^ The reaction proceeded smoothly with good-to-excellent regioselectivities (up to >19/1 in favor of the linear product) and high enantioselectivities (*ee* values up to 97%). Another application of SegPhos ligand is the kinetic resolution of unactivated allylic alcohols with monosubstituted hydrazines via the Pd-catalyzed allylic amination reported by Tian and co-workers in 2016. A range of chiral allylic alcohols and allylic hydrazines was accessed in excellent selectivity values (*s* values up to >400) using the Pd/(*S*)-SegPhos catalyst and 2,5-dichlorobenzenesulfonohydrazide **6** as additive (Scheme 50b).^143^

**Scheme 51:**
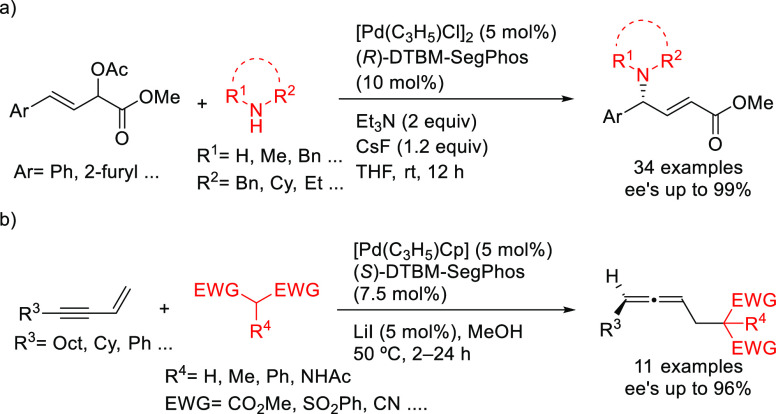
Synthesis of (a) α,β-Unsaturated-γ-amino Esters and (b) Axially Chiral 1,3-Disubstituted Allenes Using Pd/(*S*)-DTBM-SegPhos as Catalyst

A variation of SegPhos ligand was used by Zhang, Liu, and co-workers. (*R*)-DTBM-SegPhos proved to be an efficient ligand in the Pd-catalyzed allylic amination of 4-substituted 2-acetoxybut-3-enoates with primary and secondary amines. The method gave rise to a range of chiral α,β-unsaturated γ-amino esters with excellent enantioselectivities (*ee* values up to 99%; Scheme 51a).^144^ More recently, Tsukamoto’s group developed a Pd/LiI cocatalyzed reaction leading to axially chiral 1,3-disubstituted allenes from conjugated enynes.^145^ Good-to-high enantioselectivities (up to 96% *ee*) were achieved using Pd/(*S*)-DTBM-SegPhos with malonates, bis(sulfonyl)methane derivatives, acetylacetone and malononitrile (Scheme 51b).

**Scheme 52:**
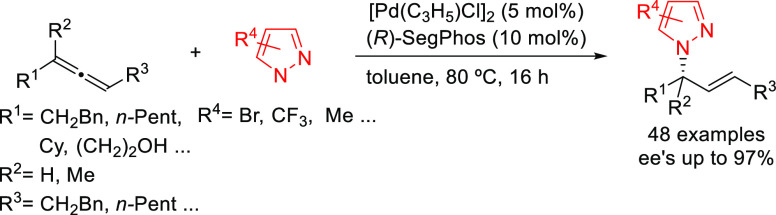
Synthesis of Allylated Pyrazoles via DKR of Allenes Using Pd/(*R*)-SegPhos as Catalyst

A further example of the use of a Pd/(*R*)-SegPhos catalyst was described by Breit’s group with the dynamic kinetic resolution of racemic allenes with pyrazoles as nucleophiles (Scheme 52).^146^ Many allylated pyrazoles that are of importance in medicinal chemistry were prepared with high enantioselectivities.

##### 2.2.2.3 Applications of Diphosphinite, Diphosphoramidite, Diphosphite, and Bisdiamidophosphite Ligands

**Scheme 53:**
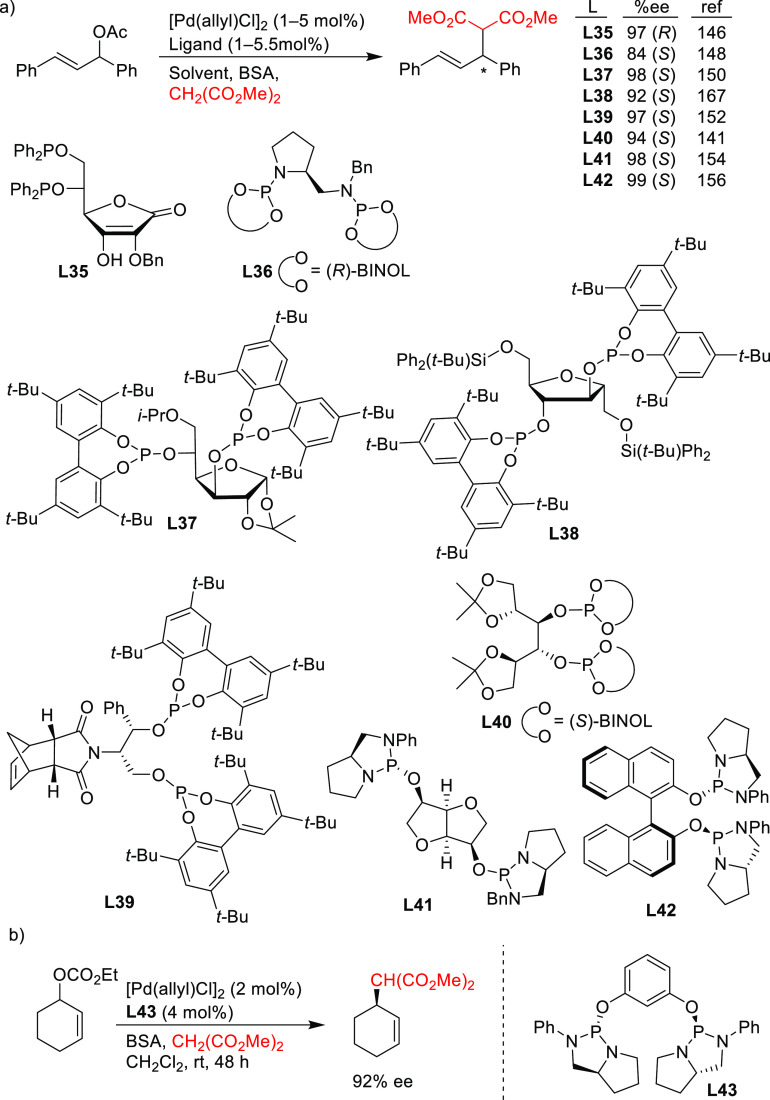
Representative Examples of P,P-Ligands Other than Diphosphines Applied in the Pd-Catalyzed AAA of (a) *rac*-1,3-Diphenylallyl Acetate and (b) Cyclohex-2-enyl Ethyl Carbonate Using Dimethyl Malonate as Nucleophile

A range of phosphinites,^147,148^ diphosphoramidites,^149^ diphosphites,^150−154^ and bisdiamidophosphites^155−164^ were also applied as ligands. High enantioselectivities were mainly achieved in the alkylation of 1,3-diphenylallyl acetate using malonates (Scheme 53a). Scheme 53 collects the most representative ligand families and their application in the allylic alkylation of the benchmark linear substrate and one example of successful application in the reaction of a cyclic substrate, using dimethyl malonate as nucleophile. Most of these ligands are diphosphites and bisdiamidophosphites, confirming the conclusions from earlier work in 2005 that demonstrated the versatility of diphosphites with biaryl groups, which have proven to be highly efficient ligands for allylation with both hindered and unhindered linear and cyclic substrates due to the flexibility of the biaryl phosphite groups that can adapt the chiral pocket to the steric demands of the substrates.^16,165,166^ Furthermore, the acceptor capacity of the phosphite groups also leads to an increase of activity (TOF’s up to >22 000 h^–1^).^167^

Among the examples collected in Scheme 53 (ligands **L35**–**L43**), it is noteworthy that the diphosphite ligand **L37** not only provided high enantioselectivities, but also allowed kinetic resolution of *rac*-1,3-diphenylallyl acetate under optimized conditions (*s* value of 122). This ligand and derivatives thereof have also been used in the Pd-catalyzed allylic alkylation of the more challenging 1-phenyl-3-acetoxyprop-1-ene, but although *ee* values of up to 83% were achieved, the regioselectivity in favor of the desired branched isomer was low.^151^ Interestingly, also the furanoside diphosphite ligand **L38**, related to **L37**, was successfully employed in the Pd-catalyzed allylic substitution with dimethyl malonate (92%) and benzyl amine using neat ionic liquids (91% *ee*). Ionic liquids allowed the Pd-catalyst to be recycled 10 times in the asymmetric allylic amination as a benchmark reaction. The catalyst achieved similar levels of enantioselectivity over the 10 runs and similar levels of conversion over the first 9 runs.^168^ The Pd/**L38** complex was also tested in the Pd-catalyzed allylic phosphination of the benchmark substrate 1,3-diphenylallyl acetate with diphenylphosphine in a neat ionic liquid. Although the catalytic activity was very high (full conversion in 6 h), the enantioselectivity was very low (13% *ee*). The authors attributed the low asymmetric induction to the competition between the P-nucleophile, the product of the allylic phosphination and the diphosphite ligand **L38** as coordinating species. Diphosphite ligand **L40**, was also applied in the Pd-catalyzed allylic alkylation of 4-phenylbut-3-en-2-yl acetate providing high regioselectivities (up to 93%) but low enantioselectivity.^152^

Gavrilov and co-workers have shown the benefit of using bisdiamidophosphites with diazaphospholididene rings, providing enantioselectivities of >90% *ee* in the allylic alkylation of 1,3-diphenylallyl acetate with dimethyl malonate regardless of the ligand backbone used.^155−160,162−164^ Among them, ligands **L41** and **L42** are highlighted for the excellent *ee* values induced in reactions with primary and secondary alkyl amines and sodium *para*-toluenesulfinate.^155^ However, *ee* values clearly decreased with less hindered substrates like cyclohexenyl carbonates or esters.^157,158^ In contrast, the resorcinol-based P-stereogenic bisdiamidophosphite **L43** provided 92% *ee* in the allylic alkylation of cyclohex-2-enyl ethyl carbonate with dimethyl malonate (Scheme 53b).^169^

**Scheme 54:**
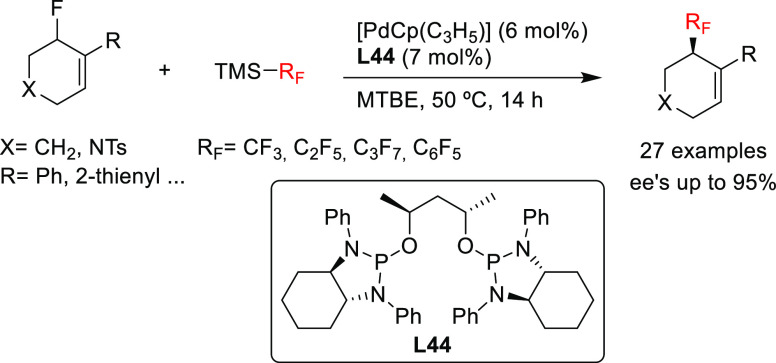
Synthesis of α-Substituted Fluoroalkylated Carbo- and Heterocycles Using Pd/**L44** as Catalyst

A recent notable application by Trost and co-workers involves the use of bisdiamidophosphite ligand **L44** (Scheme 54) in the asymmetric allylic fluoroalkylation of α-substituted cyclic allyl fluorides.^170^ A range of fluoroalkylated cyclic compounds were obtained with excellent enantioselectivities (up to 95% *ee*). The unique role of allyl fluorides suggests a synergistic interplay of the fluoride leaving group and the pronucleophile in ionization and nucleophilic activation. In addition, mechanistic studies indicate an overall retention of configuration, which is in line with a double inversion mechanism.^171^

Prochiral β-ketoesters such as 2-oxocyclohexanecarboxylate have also been extensively used as nucleophiles in the alkylation of monosubstituted substrates, such as cinnamyl acetate, providing high regioselectivities in favor of the linear products. However, *ee* values were only moderate (up to 72% *ee*) using diphosphite ligands (e.g., **L37**)^151^ and bisdiamidophosphite ligands.^159,160,163^

#### 2.2.3 Bidentate Homodonor Biscarbene Ligands

Biscarbene ligands have been rarely used because of the low-to-moderate enantioselectivities (*ee* values up to 81%) reported for the benchmark Pd-catalyzed allylic alkylation of *rac*-1,3-diphenylallyl acetate with dimethyl malonate.^172,173^

#### 2.2.4 Bidentate Homodonor *N*,*N*-Ligands

**Scheme 55:**
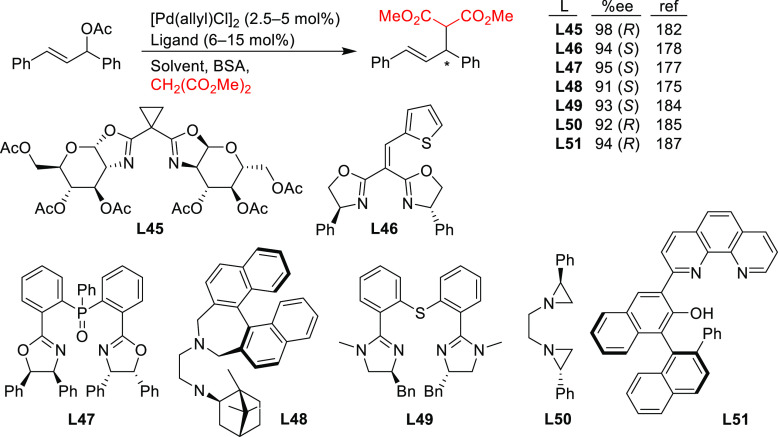
Representative Examples of Bidentate Homodonor *N*,*N*-Ligands Applied in the Pd-Catalyzed AAA of *rac*-1,3-diphenylallyl Acetate Using Dimethyl Malonate as Nucleophile

This field has been dominated by bisoxazoline ligands,^174−183^ albeit other *N*,*N*-ligands (e.g., bisamines, bisimidazolines, diaziridines, phenantroline, etc.)^184−188^ have also provided high enantioselectivities. Essentially all of them, however, suffer from low reaction rates and limited substrate scope. In addition, high enantioselectivities were generally limited to reactions of 1,3-diarylallyl acetates with several C-nucleophiles such as malonates, acetylacetone and malononitrile. Scheme 55 collects those ligand families that have provided high enantioselectivities in the allylic alkylation of 1,3-diphenylallyl acetate using dimethyl malonate as nucleophile.

**Scheme 56:**
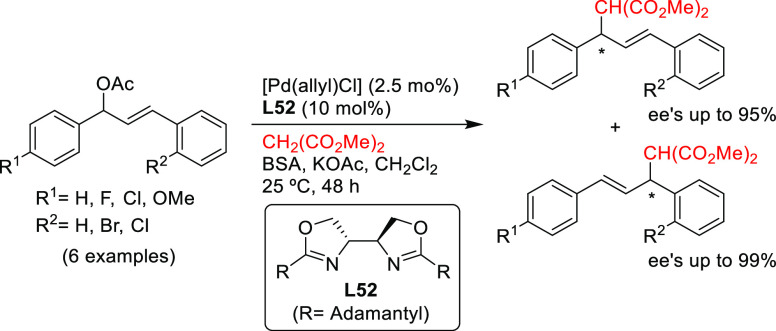
Kinetic Resolution of Unsymmetrical Allylic Acetates Using Pd/**L52** Catalytic System

The only example of the use of *N*,*N*-ligands in the alkylation of substrates other than the benchmark reaction was reported by Kesavan and co-workers. They used the Pd/**L52** bioxazoline complexas catalyst in the kinetic resolution of unsymmetrically substituted 1,3-diaryl allyl acetates with high enantioselectivities (Scheme 56).^181^

#### 2.2.5 Bidentate Heterodonor P,P′-Ligands

**Figure 4:**
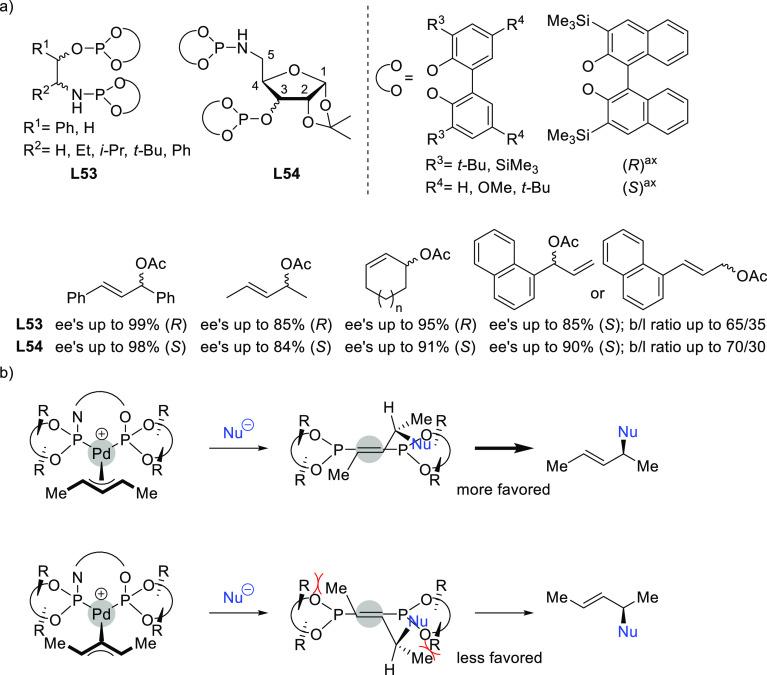
(a) Enantioselectivities achieved in allylic alkylation of some di- and monosubstituted hindered and unhindered substrates with dimethyl malonate as nucleophile using Pd/**L53** and Pd/**L54** as catalysts. (b) Schematic representation of how the steric interaction upon the attack of the nucleophile affects the outcome of the reaction.

Several types of heterodonor P,P′-ligands were evaluated in Pd-catalyzed allylations, most of them heterodonor phosphine-containing ligands (e.g., phosphine-phosphoramidite, phosphine-diaminophosphine oxide and phosphine-phosphite),^189−195^ although phosphite-phosphoramidite ligands generally performed better in terms of enantioselectivitiy and substrate scope.^196−198^ Of the latter group, we highlight two families, ligands of type **L53** derived from 1,2-amino alcohols^196^ and **L54** with a furanoside backbone.^197^ All of them share the advantage of a modular structure and short syntheses from readily available starting materials and are also air stable. They were successfully applied in the allylic substitution of mono- and disubstituted hindered substrates, unhindered cyclic substrates and unhindered linear substrates with dimethyl malonate and benzylamine (Figure 4a). For ligands **L53**, the enantioselectivity is mostly controlled by the chirality of the biaryl phosphite/phosphoramidite groups. Fine-tuning by variation of the substituents and configuration of the ligand backbone allows the adjustment of the chiral pocket for a specific substrate. In contrast, for ligands **L54** chirality at the ligand backbone has a major impact. For instance, the configuration at C3 strongly influences the size of the chiral pocket. Ligands with (*R*)-configuration at C3 generate a small chiral pocket and are well suited for reactions with unhindered substrates while those with (*S*)-configuration have a larger chiral pocket and induce better enantioselectivities with hindered substrates. Subtle variations at the ligand backbone and at the biaryl phosphite/phosphoramidite moieties allows one to maximize the enantioselectivity for each substrate type. Interestingly, ligands **L53** and **L54** provided higher *ee* values than their diphosphite analogues, which reaffirmed the importance of introducing electronic differentiation of the two coordinating atoms in the ligand design. NMR spectroscopic studies of Pd η^3^-allyl intermediates, which contain 1,3-diphenyl, 1,3-dimethyl or cyclohexenyl allyl groups, helped to understand the effect of the ligand parameters on catalytic performance. In the allylic alkylation of linear hindered substrates, it was found that in ligands **L53** the substituents at the carbon atoms of the amino alcohol backbone and in the *para*-position of the biaryl moieties fixed the configuration of the biaryl moieties. This prevented the formation of mixtures of *syn*/*syn* and *syn*/*anti* isomers and was a key factor for obtaining high enantioselectivities. On the other hand, for unhindered substrates it was found that the steric interaction upon attack of the nucleophile was the key factor to control enantioselectivity, favoring the nucleophilic attack to one specific *syn*/*syn* isomer (*endo* or *exo*), the one leading to a reduction of steric strain (Figure 4b). It is known that nucleophilic substitution of the Pd-1,3-allyl cationic complex to form the Pd-olefin complex must be accompanied by rotation (see section 2.4). Model studies showed that the substituents at the carbon atoms of the amino alcohol backbone control the conformation of the seven-membered chelate favoring the attack of the nucleophile to one of the *syn*/*syn* isomers (*endo* or *exo*), the one that reduces the steric strain during the rotation. With ligands **L54**, for enantioselectivities to be high, the configuration at C3, the position of the phosphoramidite group (at either C5 or C3 of the furanoside backbone) and the configurations of the biaryl moieties needed to be properly combined to either enhance electronic differentiation between the most electrophilic terminal allylic carbon atoms of the isomers formed or favor formation of the isomer that reacts the fastest with the nucleophile. For both families it was found that nucleophilic attack preferentially occurs at the allylic terminal carbon atom *trans* to the phosphoramidite.

#### 2.2.6 Bidentate Heterodonor P,N(sp^2^)-Ligands

##### 2.2.6.1 Application of PHOX Phosphine-Oxazoline Ligands and Previously Reported Modifications

**Scheme 57:**
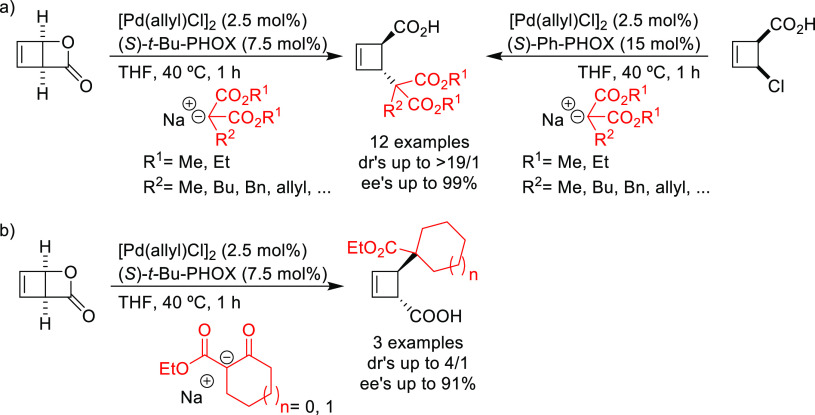
Deracemization of (a) *cis*-2-Oxabicyclo[2.2.0]hex-5-en-3-one and *cis*-4-Chlorocyclobut-2-ene Carboxylic Acid Derivatives Using a Range of Malonates and (b) *cis*-2-Oxabicyclo[2.2.0]hex-5-en-3-one Using Ketoesters

After 2008, new applications of the original PHOX ligands or their previously reported modifications were reported. Here, we highlight the work of Maulide’s group on the effective deracemization of the strained lactone *cis*-2-oxabicyclo[2.2.0]hex-5-en-3-one and the *trans*-4-chlorocyclobut-2-ene carboxylic acid using malonates with *t*-Bu-PHOX and Ph-PHOX ligands, respectively (Scheme 57a).^74,75,199^ In contrast to monophosphoramidite ligands, that led to *cis*-alkylated products (Scheme 5), the reaction with PHOX ligands was highly *trans*-selective providing the alkylated products with high diastereo- and enantioselectivities (up to >19/1 dr and up to 98% *ee*). The *t*-Bu-PHOX ligand also performed well with several ketoesters, leading to the formation of *trans*-disubstituted cyclobutenes with an additional sterogenic center (Scheme 57b). The *ee* values achieved were again very good (up to 91% *ee*) although the diastereoselectivity was not fully controlled (up to 4/1 dr).

**Scheme 58:**
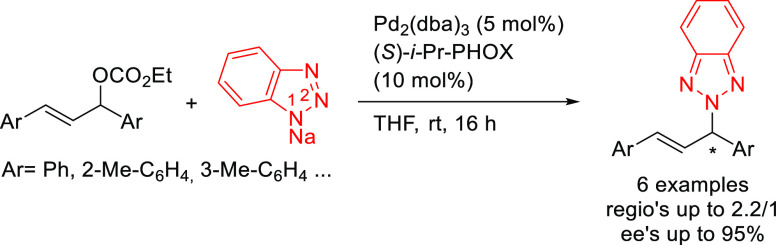
Pd-Catalyzed Allylic Amination of 1,3-Diaryl Allyl Carbonates with Sodium Benzotriazolide Using Pd/(*S*)-*i*-Pr-PHOX as Catalyst

In 2011, Zhao’s group reported the first use of sodium benzotriazolide as a nitrogen-based nucleophile in the allylic amination of ethyl 1,3-diaryl allyl carbonates with Pd/(*S*)-*i-*Pr-PHOX as catalyst. This reaction has added difficulty caused by the presence of two nucleophilic nitrogen atoms (N1 and N2), which can lead to two regioisomers. Both isomers were formed in moderate-to-high enantioselectivities (*ee* values up to 95% for the N2 isomer), although the regioselectivities were typically low (up to 2.2/1; Scheme 58).^200^

**Scheme 59:**
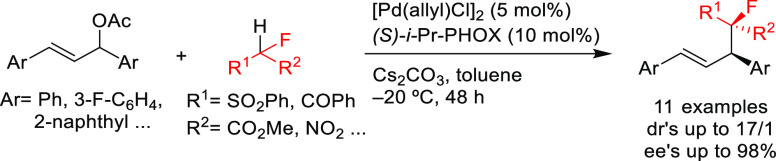
Pd-Catalyzed AAA of 1,3-Diaryl-Substituted Allylic Substrates with Monofluorinated Methylene Derivatives Using Pd/(*S*)-*i*-Pr-PHOX as Catalyst

More recently, the same group also showed that the Pd/(*S*)-*i*-Pr-PHOX catalyst can be used for the diastero- and enantioselective Pd-catalyzed allylic alkylation of 1,3-diaryl-substituted allylic substrates with monofluorinated methylene derivatives such as methyl 2-fluoro-2-(phenylsulfonyl)acetate (Scheme 59).^201^ This reaction provides access to fluorinated allylic compounds with two stereogenic centers with high *ee* values (up to 98%) and moderate-to-high diastereoselectivities (up to 17/1). The dr values were affected by the steric demands of the substrate and the substituted fluorinated methylene derivatives. The utility of this transformation was demonstrated with the synthesis of (*S*,*S*,*S*)-3,4-dihydro-2-*H*-pyrrole-1-oxide with 95% *ee* and >20/1 dr.

**Scheme 60:**
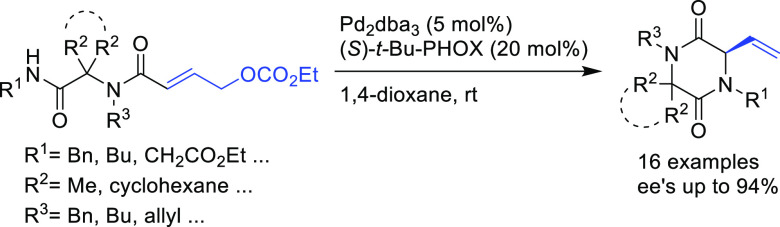
Synthesis of Chiral Spiro-Diketopiperazines via Intramolecular Pd/(*S*)-^t^Bu-PHOX Catalyzed Allylation

Another recent application was reported by Ruijter’s group who found that the Pd/(*S*)-*t*-Bu-PHOX catalyst efficiently catalyzed the intramolecular allylic amination of Ugi adducts (Scheme 60).^202^ A range of spiro-diketopiperazines, which are important building blocks for drug synthesis, were obtained in high yields and enantioselectivities (up to 94% *ee*).

**Scheme 61:**
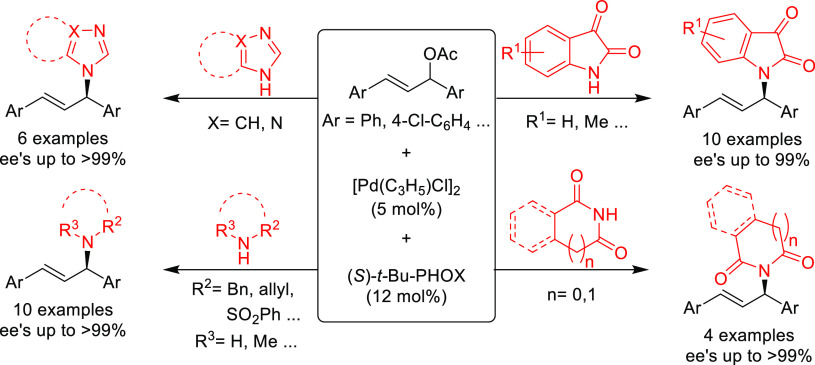
Pd-Catalyzed Allylic Amination Using Isatins, Sulfonamides, Imides, Amines, and Several N-Heterocyclic Nucleophiles Reactions carried out using CHCl_3_ as solvent at 25 °C for 48 h.

In 2020 Wolf’s group reported the use of the Pd/(*S*)-*t*-Bu-PHOX catalyst for the allylic amination of several 1,3-diaryl allyl acetates with a range of isatins, sulfonamides, imides, amines and several *N*-heterocycles (Scheme 61).^203^

**Scheme 62:**
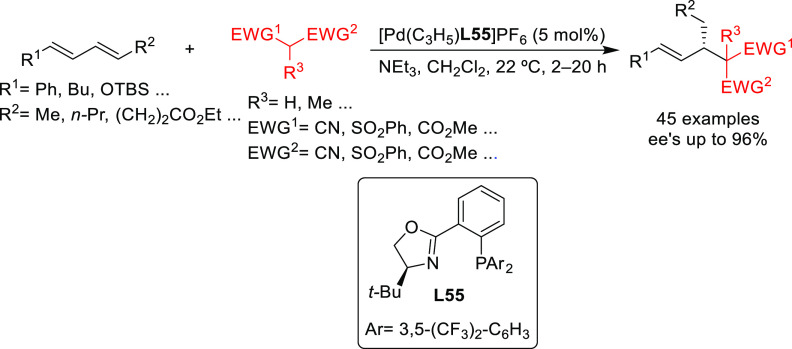
Pd-Catalyzed Allylic Alkylation of Acyclic 1,3-Dienes with a Range of C-Nucleophiles Using Pd/**L55** as Catalyst

Among the new applications of known variants of PHOX ligands we highlight the work by Malcolmson’s group on the allylic alkylation of acyclic 1,3-dienes with C-nucleophiles, such as Meldrum’s acid derivatives, β-diketones, and malononitriles using the electron-deficient PHOX derivative **L55** and triethylamine as base (Scheme 62).^204,205^ An excess of triethylamine was needed to form the monoalkylated product selectively. Many aryl- and alkyl-substituted dienes were efficiently alkylated with a range of β-dicarbonyl compounds as nucleophiles to yield allyl compounds with a stereogenic center at the carbonyl β-position. Subsequently, the authors found that a noncoordinating BAr_F_ counterion and the addition of NEt_3_·HBAr_F_ as a Brønsted acid cocatalyst improved the reaction.^205^

**Scheme 63:**
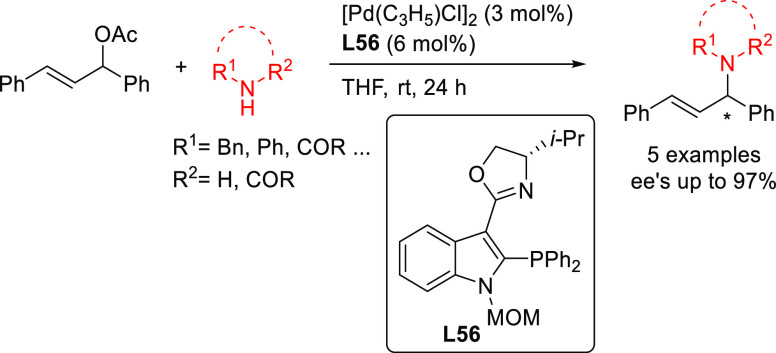
Pd-Catalyzed Allylic Amination of 1,3-Diphenylallyl Acetate with Amines Using Pd/**L56** as Catalyst

Franzén and co-workers successfully applied the phosphine-oxazoline ligand **L56**, possessing an indole instead of a phenyl backbone, in the Pd-catalyzed allylic amination of *rac*-1,3-diphenylallyl acetate with a range of amines (*ee* values up to 97%; Scheme 63).^206^^207^

**Scheme 64:**
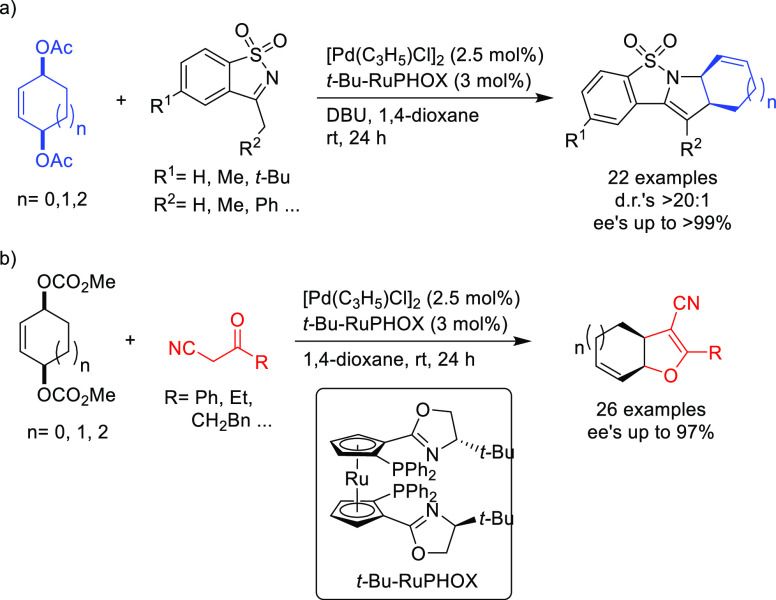
Construction of Enantioenriched (a) Fused Azabicycles and (b) Bicyclic Dihydrofurans via Pd/RuPHOX-Catalyzed Allylic Desymmetrization Processes

Among several new applications of RuPHOX ligands, the synthesis of chiral fused azabicycles by an allylic substitution cascade was reported, involving an initial desymmetrization of cyclic *meso*-diacetates by allylic alkylation, followed by an allylic amination using cyclic *N*-sulfonylimines as both C- and N-nucleophiles (Scheme 64a).^208^ The initial alkylation is the enantioselectivity-determining step in this transformation, which allows the synthesis of azabicycles in excellent diastereo- and enantioselectivities (dr’s > 20/1 and up to >99% *ee*). The same group later applied the *t*-Bu-RuPHOX ligand in the related reaction of *meso*-dicarbonates with 3-oxo-nitriles (Scheme 64b).^209^ The resulting chiral bicyclic dihydrofurans were obtained in high yields and *ee* values (up to 97%).

**Scheme 65:**
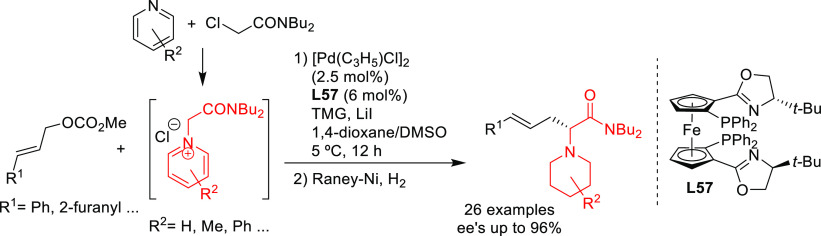
Synthesis of Chiral Piperidine-Containing Amino Acid Derivatives via a One-Pot Pd-Catalyzed Allylic Substitution/Hydrogenation Sequence

A ferrocene analog of RuPHOX (ligand **L57**) was applied in a one-pot Pd-catalyzed allylic substitution/hydrogenation sequence with several cinnamyl-type methyl carbonates and in situ formed α-(pyridine-1-yl)-acetamides as nucleophiles. Chiral piperidine-containing amino acid derivatives were obtained with high yields and enantioselectivities (up to 96% *ee*; Scheme 65).^210^

**Scheme 66:**
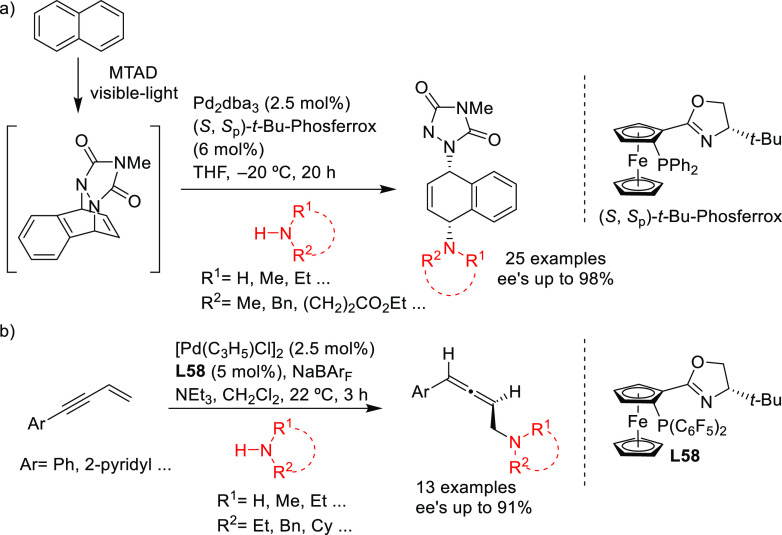
Preparation of Chiral (a) *syn*-1,4-Diaminated Products Derived from Naphthalene and (b) Aminomethyl-Substituted Allenes Using Phosferrox Ligands

Phosferrox ligands, in which the phenyl group of the PHOX ligand had been replaced by a ferrocene moiety, recently found new applications in the Pd-catalyzed AAA. For instance, Sarlah’s group developed a one pot protocol for the dearomative *syn*-1,4-diamination of naphthalene that involves a visible-light mediated [4+2]-photocycloaddition followed by a Pd-catalyzed allylic amination (Scheme 66a).^211^ A variety of amines were employed in this formal desymmetrization of naphthalene, leading to *syn*-1,4-diaminated products with high enantioselectivities (up to 98% *ee*) using the (*S*,*S*_*p*_)-*t*-Bu-Phosferrox ligand. Another interesting example is the work of Malcolmson’s group on the synthesis of chiral aminomethyl-substituted allenes in high *ee* values (up to 91% *ee*) using the (*S*,*S*_*p*_)-*t*-Bu-Phosferrox derivative **L58** bearing an electron-poor bis(perfluorophenyl)phosphine group (Scheme 66b).^212^

**Scheme 67:**
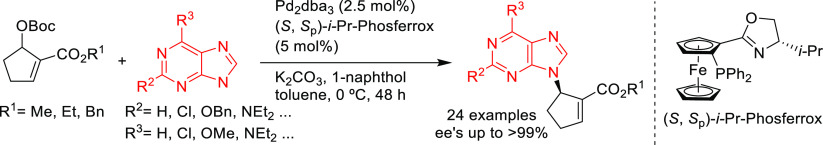
Synthesis of Chiral Carbocyclic Nucleosides via Pd/(*S*,*S*_*p*_)-*i*-Pr-Phosferrox-Catalyzed Allylic Amination

Guo’s group reported the use of the (*S*,*S*_*p*_)-*i*-Pr-Phosferrox ligand for the synthesis of chiral carbocyclic nucleosides via Pd-catalyzed allylic amination of alicyclic Morita–Baylis–Hillman adducts with purines (Scheme 67).^213^ The reaction proceeded with excellent N9/N7-selectivities (>19/1) and excellent enantioselectivities (up to >99% *ee*).

**Scheme 68:**
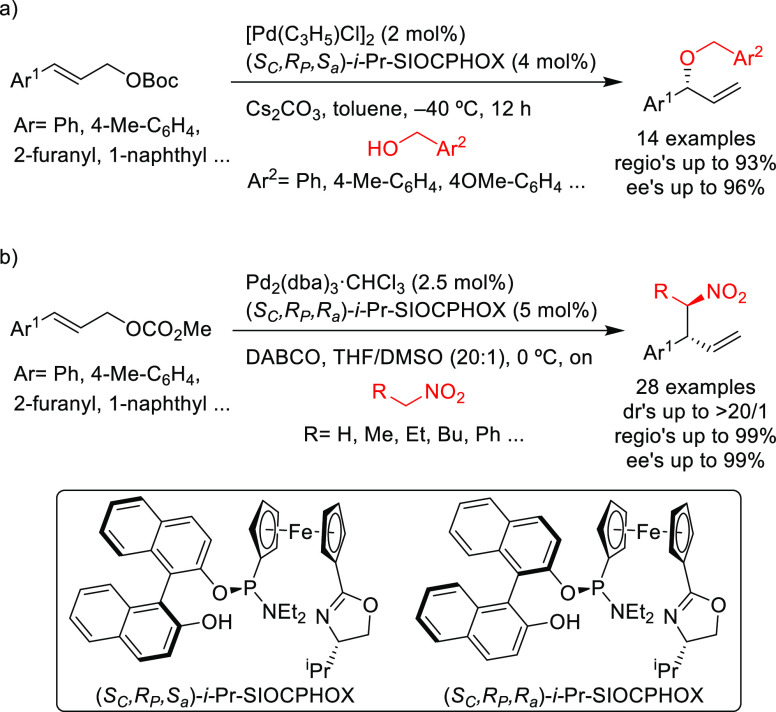
Pd-Catalyzed AAA of a Range of Cinnamyl-Type Carbonates with (a) Benzyl Alcohols and (b) Nitroalkanes Using Pd/SIOCPHOX as Catalyst

Hou’s group continued taking advantage of the ferrocene-binol-based P-oxazoline (SIOCPHOX) ligands, expanding the nucleophile scope for the Pd-catalyzed allylic alkylation of monosubstituted substrates. Currently Hou’s ligand represents the state of the art for this substrate type using both C- and N-nucleophiles.^57^ This work was further expanded to the asymmetric etherification of monosubstituted allylic substrates with benzyl alcohols. High regio- and enantioselectivities were achieved using Pd/(*S*_*c*_,*R*_*p*_,*S*_*a*_)-*i*-Pr-SIOCPHOX as catalyst (Scheme 68a).^214^ Neither the introduction of a *p*-nitro group on the benzyl alcohol nor the use of secondary and tertiary alcohols were tolerated in this reaction.

Nitromethane^215^ and other nitroalkanes^216^ were also used as nucleophiles with this catalyst system (Scheme 68b) with excellent regio- and enantioselectivities. In reactions with nitroalkanes other than nitromethane, two adjacent stereogenic centers were formed with high diastereoselectivity (dr values up to >20/1). The SIOCPHOX ligand screening indicated that the central chirality on the phosphorus atom controls the configuration of the alkylated product. Results also showed that there is a cooperative effect between the different chirality elements that results in a matched combination for the (*S*_*c*_,*R*_*p*_,*R*_*a*_)-*i*-Pr-SIOCPHOX ligand. The usefulness of these transformations was demonstrated with the synthesis of important building blocks and drugs, such as (*R*)-rolipram and (*R*)-baclofem. The former is an anti-inflammatory agent and antidepressant, while the latter is an antispasmotic agent (see section 2.5).

##### 2.2.6.2 Application of New P-Oxazolines and Other P,N(sp^2^)-Ligands

**Figure 5:**
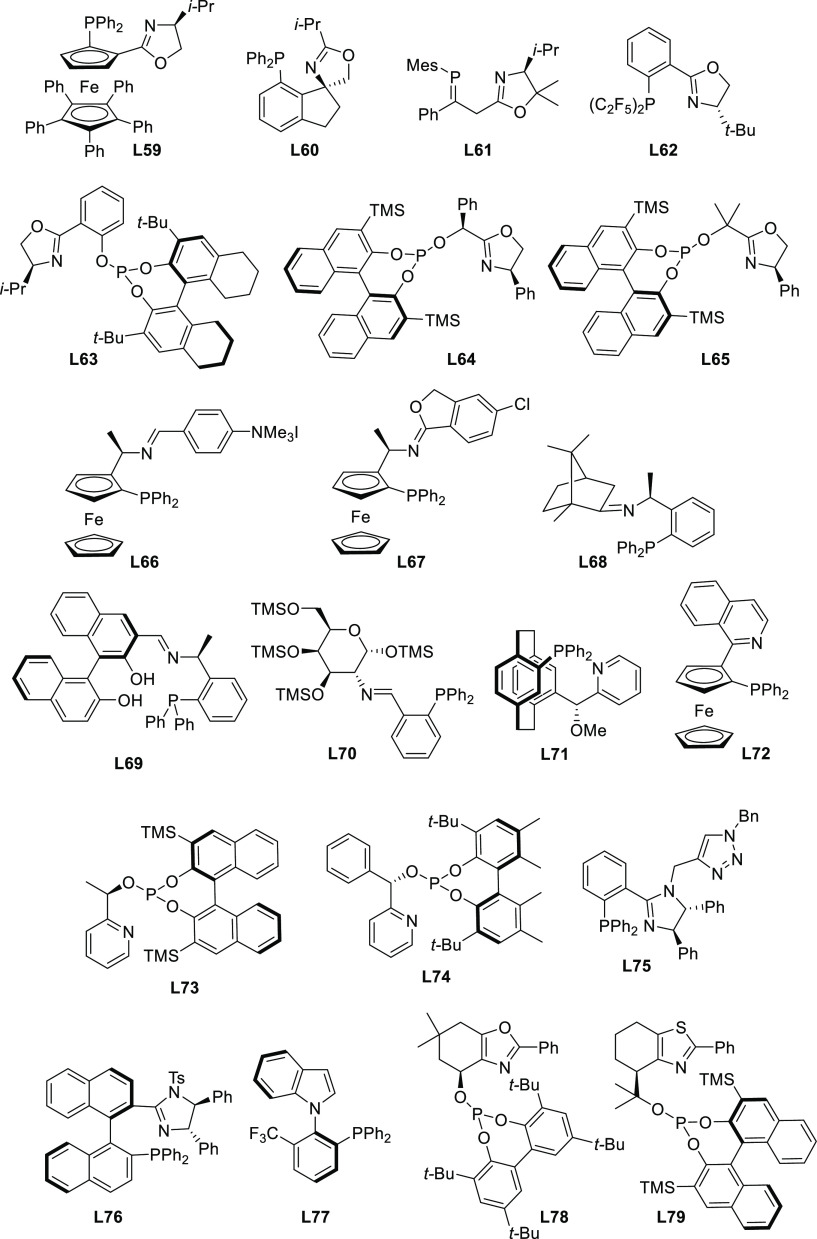
Representative examples of new heterodonor P,N(sp^2^)-ligands applied in Pd-catalyzed allylic substitutions.

The interest in this kind of ligand for Pd-catalyzed allylic substitution continues to be spurred by the early success of the Pd-PHOX catalytic system. Albeit the field is still dominated by P-oxazoline ligands,^74,75,217−231^ other ligands, such as P-iminos,^232−240^ P-pyridine/quinolines,^241−248^ and others,^249−257^ are increasingly studied. Figure 5 collects the most successful classes of new P-oxazoline ligands and other new P,N(sp^2^)-ligands.

**Scheme 69:**
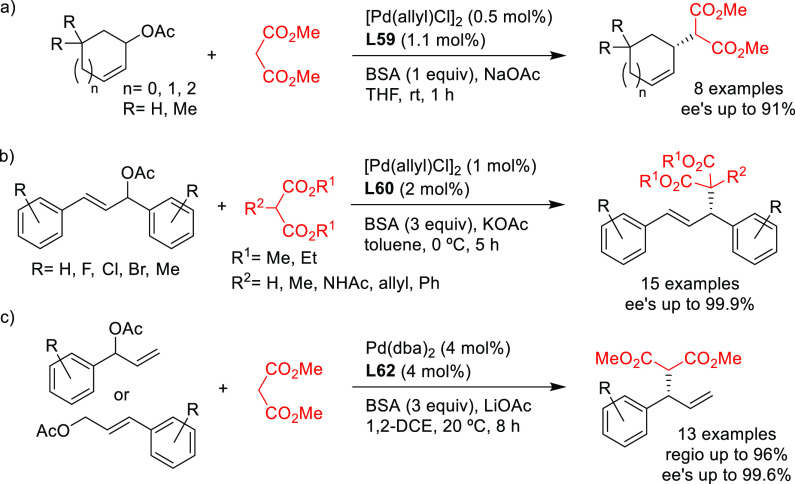
Pd-Catalyzed Allylic Alkylation Using Malonates as Nucleophiles of (a) Cyclic, (b) Linear 1,3-Arylated, and (c) Monosubstituted Substrates with Pd/**L59**, Pd/**L60**, and Pd/**L62** as Catalyst, Respectively

Most of the research on P-oxazolines was focused on novel phosphine-oxazoline ligands by either modifying the ligand backbone or the electronic properties of the phosphine group. Two of the most successful ligand backbone modifications are represented by the bulky pentaphenylferrocenylphosphine-oxazoline ligand **L59**(220) and the highly rigid ligand **L60**(222) with a spirocyclic backbone. Ligand **L59** was specifically designed to improve the enantioselectivity achieved with PHOX ligands in the Pd-catalyzed allylic substitution of cyclic substrates with dimethyl malonate (Scheme 69a). Notably, high enantioselectivities (*ee* values up to 91%) were obtained even with the more challenging cyclopentenyl acetate substrate. Ligand **L60**, on the other hand, showed excellent catalytic performance (*ee* values up to 99.9%) in the allylic alkylation of 1,3-diarylallyl acetates with diverse malonates, some of them with a substituent in the α-position (Scheme 69b),^222^ as well as with indoles and alkyl alcohols.^223^ Two notable modifications of the electronic properties of the phosphine group were introduced by Gates and Shen. The Pd/**L61** catalyst induced high enantioselectivities (up to 92% *ee*) in the reaction of *rac*-1,3-diphenylallyl acetate with a range of malonates.^218^ Shen’s group prepared perfluoroalkylated derivatives of the PHOX ligand inspired by the previous discovery that the introduction of a π-acceptor P-group increases the regioselectivity toward the branched product in the alkylation of monosubstituted substrates.^47,219^ As a result, a range of arylated monosubstituted substrates could be successfully alkylated with **L62** (b/l ratio up to 96/4 and *ee* values up to 99%; Scheme 69c). Somewhat inferior *ee* values were observed in reactions with branched substrates, probably as a result of a memory effect (vide infra).

**Scheme 70:**
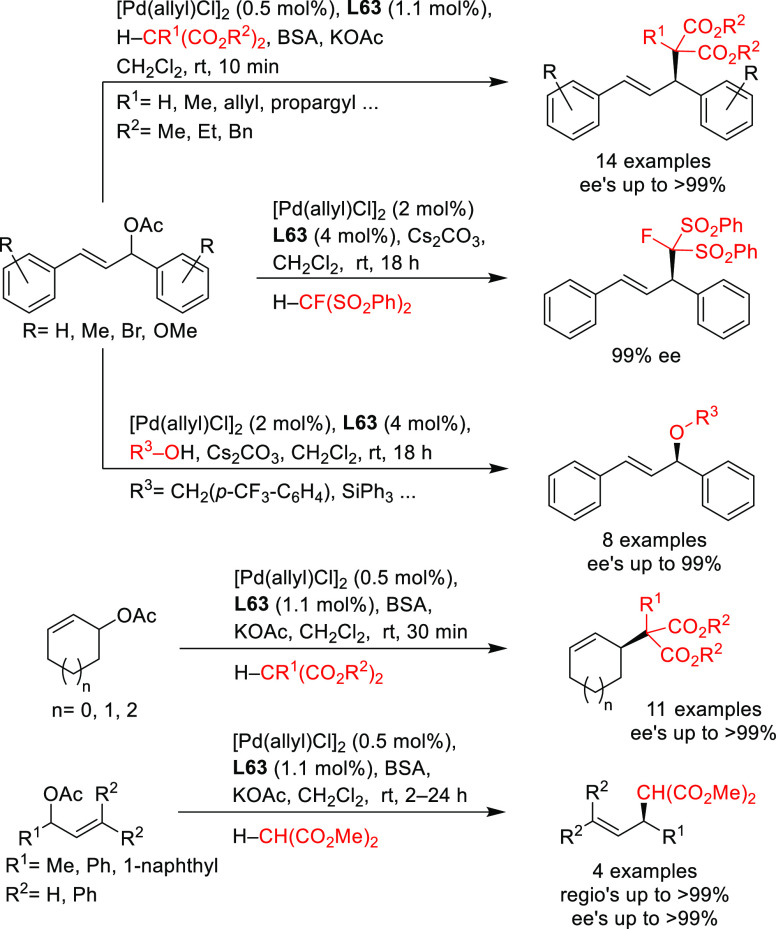
Pd-Catalyzed Allylic Substitution of Several Substrate Types with C-, N-, and O-Nucleophiles Using Pd/**L63** as Catalyst

Another fruitful design concept was the replacement of the phosphine group in the PHOX ligands by biaryl phosphite groups. In this respect, the use of the air stable ligand **L63** afforded high enantioselectivities (*ee* values up to >99%) in the allylic substitution of hindered linear substrates and unhindered linear and cyclic substrates (Scheme 70).^229^ Mechanistic studies confirmed that its large substrate scope is a result of the flexibility of the biaryl phosphite group that allows the size of the chiral pocket to adapt to the steric demands of the substrates (see section 2.4). A range of 1,3-diarylallyl acetates and cyclic allylic substrates of different ring sizes with several malonates, including examples with different substituents at the α-position, were also successfully alkylated.^229^ High *ee* values were also achieved using 1-fluorobis(phenylsulfonyl)methane, a fluoromethide equivalent,^258^ and some O-nucleophiles such as electron-poor benzylic alcohols and silanols. The Pd/**L63** catalyst also provided high regio- and enantioselectivities for a range of mono- and trisubstituted substrates.

**Scheme 71:**
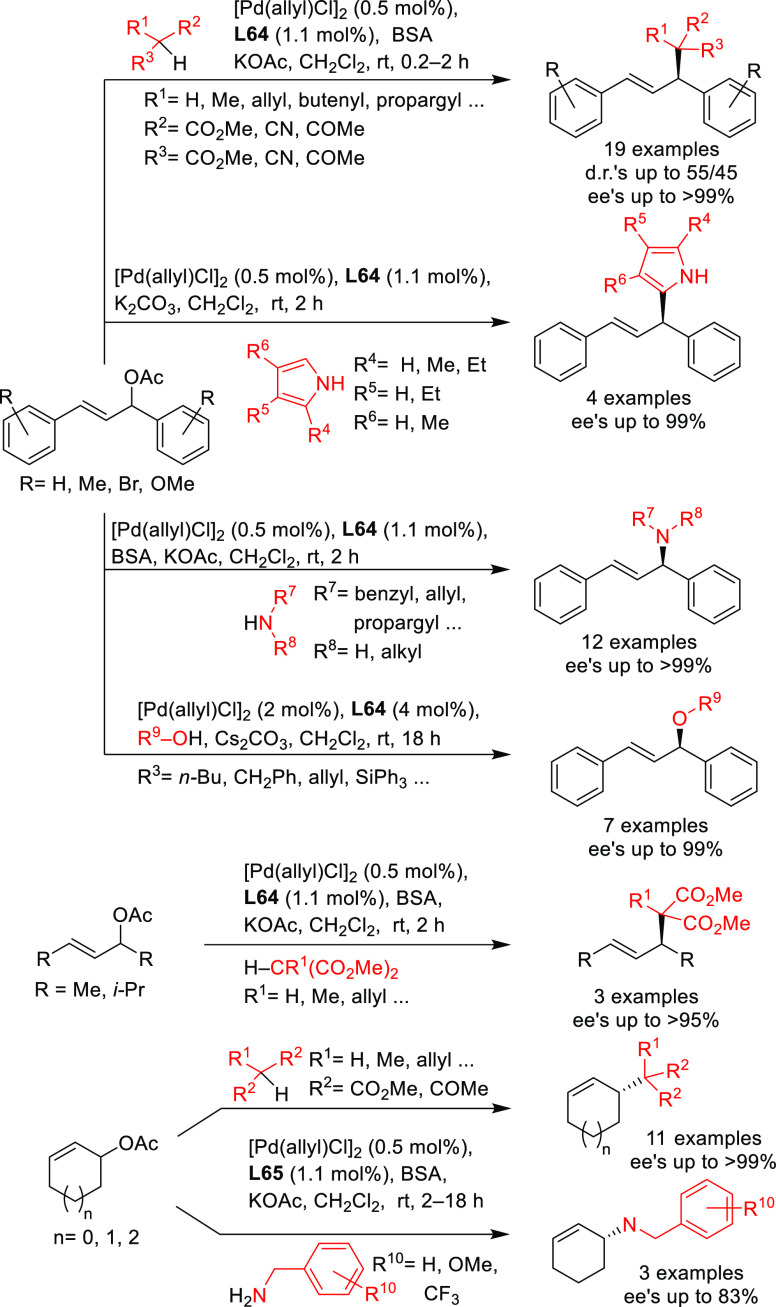
Pd-Catalyzed Allylic Substitution of Several Symmetrical Disubstituted Substrates with a Range of Nucleophiles Using Pd/**L64** and Pd/**L65** as Catalysts

**Scheme 72:**
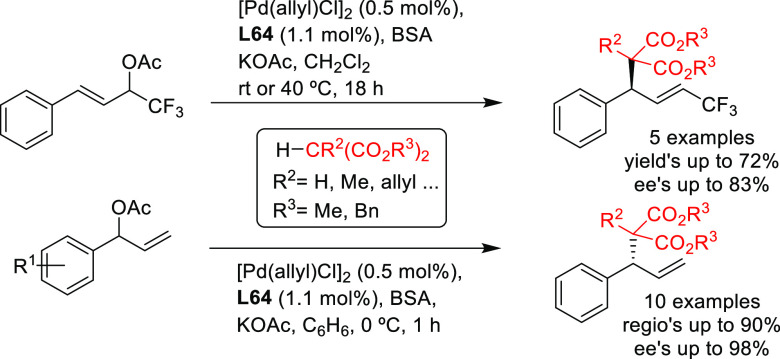
Pd-Catalyzed Allylic Alkylation of Several Unsymmetrical Substrates with a Range of Malonates Using Pd/**L64** as Catalyst

With the aim to further improve the catalyst structure with air-stable and readily available ligands, the *o*-phenylene tether in ligand **L63** was replaced by an alkyl backbone chain. Compared to Pd/**L63**, the Pd/**L64** catalyst provided higher activities (TOF up to 8000 h^–1^) and excellent oenantiocontrol in a wider range of mono- and symmetrically disubstituted substrates (*ee* values up to >99%, 74 examples in total; Schemes 71 and 72).^230^ High enantioselectivities were achieved in a wide range of symmetrically disubstituted linear allylic acetates, containing alkyl or aryl substituents, with many C-nucleophiles including α-substituted malonates, malononitrile, diketones, 2-cyanoacetates and pyrroles (Scheme 71). The Pd/**L64** catalyst also showed excellent enantioselectivities with various primary and secondary amines, containing either alkyl or aryl groups, using benzylic, allylic and alkylic alcohols as well as silanols (Scheme 71). With ligand **L65**, a modification of ligand **L64** with different substituents at the alkyl backbone chain, *ee* values could be improved to up to >99% in the allylic alkylation of cyclic substrates (Scheme 71).

Moreover the Pd/**L64** catalyst is one of the few catalytic systems that can deracemize unsymmetrically disubstituted substrates, such as 1,1,1-trifluoro-4-phenylbut-3-en-2-yl acetate, via a dynamic kinetic asymmetric transformation with a range of malonates (yield’s up to 72% and *ee* values up to 80%; Scheme 72). This family of catalyst precursors was also applied in the Pd-catalyzed allylic alkylation of 1-arylallyl acetates with malonates (regioselectivities up to 90% and *ee* values up to 98%; Scheme 72). However, the regioselectivities in favor of the branched product diminished when using α-substituted malonates (e.g., regioselectivities dropped from 83% using dimethyl malonate to 60% using dimethyl 2-methylmalonate).^230^

The replacement of the phosphine moiety in the PHOX ligand by a diamidophosphite moiety was also studied, although the *ee* values achieved were lower than those obtained with the phosphite analogues (e.g., *ee* values up 96% in the allylic alkylation of 1,3-diphenyllallyl acetate with dimethyl malonate).^231^

**Scheme 73:**
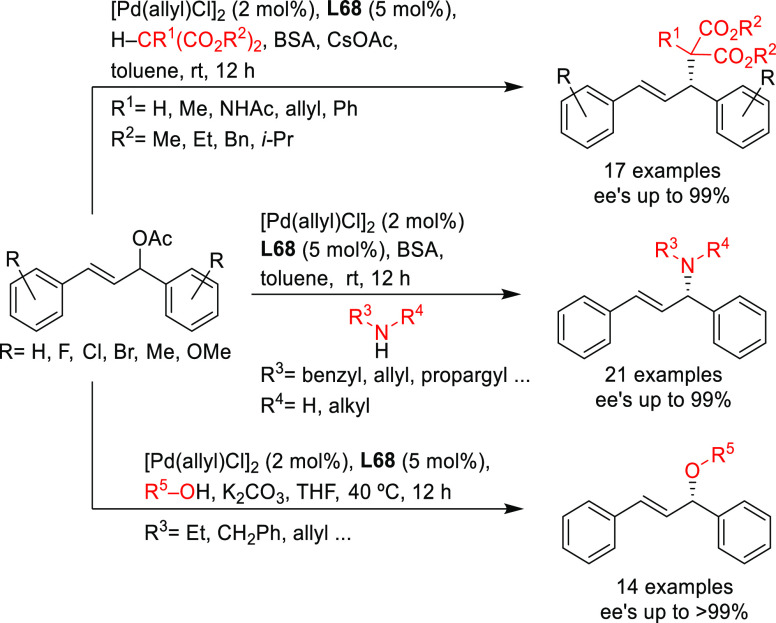
Pd-Catalyzed Allylic Substitution of a Range of *rac*-1,3-Diarylallyl Acetates with Pd/**L68** as Catalyst

A review of the P-imino ligands applied in this transformation reveals that derivatives of chiral-1-(2-phosphino)ferrocenylethylamine are particularly well suited. For instance, ligand **L66** provided high enantioselectivities (up to 95% *ee*) in the allylic alkylation of *rac*-1,3-diphenylallyl acetate with a range of malonates.^232^ Subsequently, Van der Eycken’s group further improved the ligand by introducing a ketamine group (ligand **L67**) achieving *ee* values of up to 99% in the alkylation of *rac*-1,3-diphenylallyl acetate with malonates and enantioselectivities of up to 90% *ee* in the alkylation of cyclic substrates with dimethyl malonate.^233^ Later Xu’s group demonstrated that the ferrocenyl moiety is not essential to achieve high enantioselectivities.^239^ They developed a D-camphor-based phosphine-imino ligand **L68** inducing excellent enantioselectivities (up to 99% *ee*) in the allylic substitution of 1,3-diarylallyl acetates with several malonates, amines and nonaromatic alcohols (Scheme 73).

**Scheme 74:**
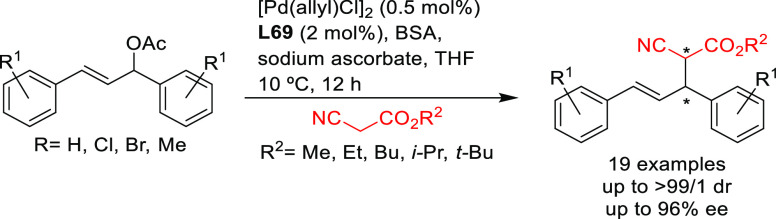
Pd-Catalyzed Allylic Alkylation of *rac*-1,3-Diarylallyl Acetates with 2-Cyanoacetates Using Pd/**L69** as Catalyst

The same group modified ligand **L68** by replacing the camphor group by a chiral 1,1′-bi-2-naphthyl moiety (ligand **69**). The Pd/**L69** catalyst proved to be highly efficient in the allylic alkylation of several 1,3-diarylallyl acetates with unsubstituted 2-cyanoacetates producing chiral monosubstituted 2-cyanoacetates with two adjacent stereogenic centers with high diastereo- and enantioselectivities (up to >99/1 dr and up to 96% *ee*; Scheme 74).^259^

Excellent enantioselectivities were also induced by phosphine-imino ligands with a chiral imine group.^236,240^ For instance, enantioselectivities of up to >99% *ee* were described with the phosphine-imino ligand **L70** derived from d-glucosamine.^240^

Pyridine/quinoline-based heterodonor P,N-ligands have also been used extensively.^241−248^ For example, Jiang’s group successfully applied the [2,2]-paracyclophane-derived phosphine-quinoline ligand **L71** in the allylic alkylation of *rac*-1,3-diphenylallyl acetate with dimethyl malonate (*ee* values up to 99%).^241^ Similar enantioselectivities were disclosed with the ferrocene-based phosphine-quinoline ligand **L72** with planar quirality.^248^

**Scheme 75:**
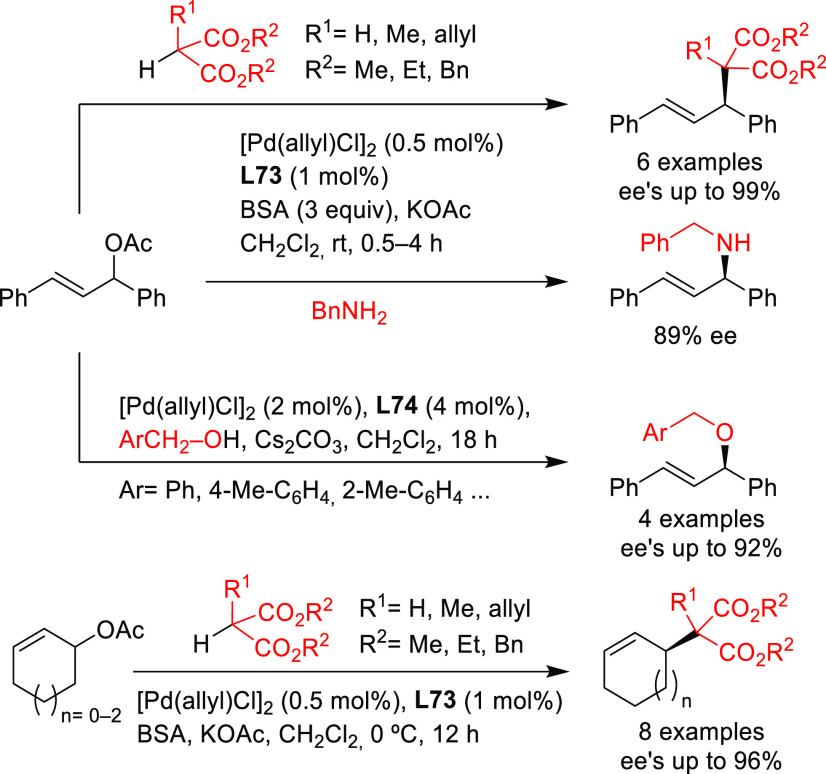
Pd-Catalyzed Allylic Substitution of Disubstituted Linear and Cyclic Substrates with Several Nucleophile Types Using Phosphite-Pyridine Ligands **L73** and **L74**

Again, the introduction of a biaryl phosphite moiety proved to be a valid approach to expand the substrate scope. In this respect, the phosphite-pyridine ligands **L73** and **L74** provided excellent enantioselectivities for several disubstituted linear and cyclic substrates with a wide range of malonates as well as N- and O-nucleophiles, such as benzyl amine and benzyl alcohols (Scheme 75). High enantioselectivities were also achieved for unsymmetrically trisubstituted substrates after slightly modifying the pyridyl and phosphite groups (*ee* values up to >99%).^246^

**Scheme 76:**
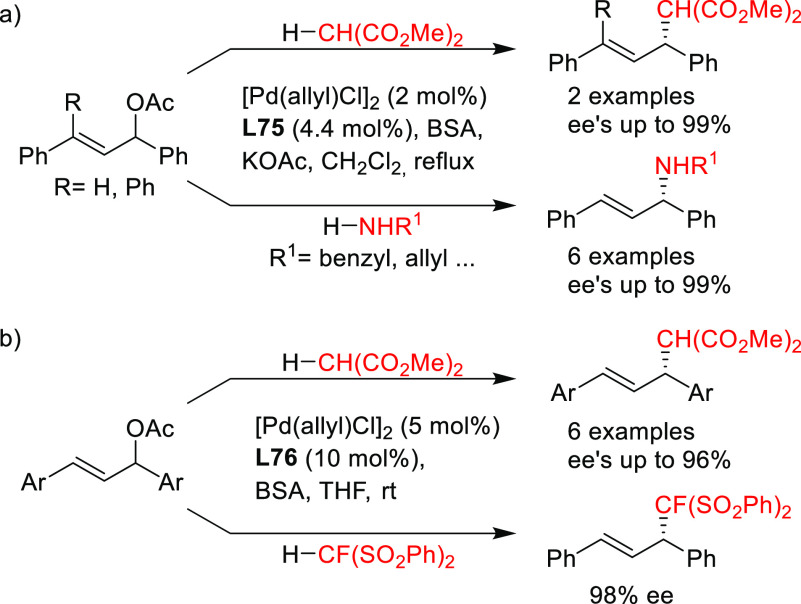
Pd-Catalyzed Allylic Substitution of Di- and Trisubstituted Linear Substrates Using (a) Pd/**L75** and (b) Pd/**L76** as Catalysts

Many research groups have designed novel heterodonor P,N-ligands with other N-sp^2^ donor groups ranging from labile imidazolines to robust indoles, pyrazoles, imidazoles, and oxazoles among others.^249−257^ The most promising results have been achieved with phosphine-imidazoline/indole and phosphite-oxazole/thiazole ligands. For example, the phosphine-imidazoline ligand **L75** containing a remote triazole substituent proved to be more effective than the parent ligand without the triazole group in the allylic substitution of di- and triaryl-substituted linear substrates with dimethyl malonate and amines as nucleophiles (*ee* values up to 99%; Scheme 76a).^256^ The group of Shi and co-workers showed that the binaphthyl-based phosphine-imidazoline ligand **L76** provided high *ee* values (up to 97%) in the allylic substitution of 1,3-diarylallyl acetates with dimethyl malonate and 1-fluoro-bis(phenylsulfonyl)methane (Scheme 76b).^257^ Indole-derived ligand **L77** developed in Mino’s group showed excellent enantioselectivities (up to 99% *ee*) in the allylic alkylation of *rac*-1,3-diphenylallyl acetate with dimethyl- and diethylmalonates.^254^ Phosphite-oxazole ligand **L78** also provided high *ee* values (up to 95%) in the allylic substitution of di- and triaryl-substituted linear substrates using both dimethyl malonate and benzylamine as nucleophiles. To increase the enantioselectivities for the more demanding unhindered di- and monosubstituted substrates, a more rigid thiazoline analog (ligand **L79**) was required (e.g., *ee* values up to 92% with *rac*-1,3-dimethylallyl acetate and regioselectivities up to 80% and *ee* values up to 92% in the alkylation of 1-(1-naphthyl)allyl acetate).^255^

#### 2.2.7 Bidentate Heterodonor P,N(sp^3^)-Ligands

**Scheme 77:**
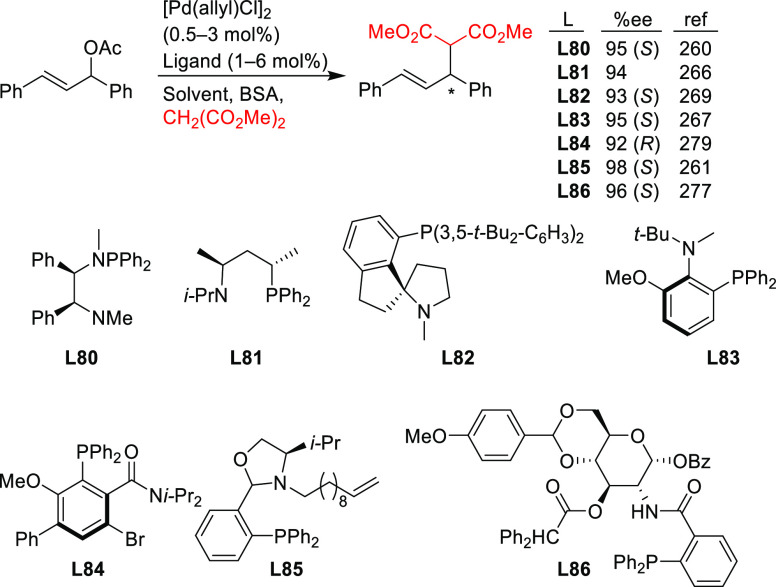
Representative Examples of Bidentate Heterodonor Phosphine-N(sp^3^) Ligands Applied in the Pd-Catalyzed Allylic Alkylation of *rac*-1,3-Diphenylallyl Acetate with Dimethyl Malonate as Nucleophile

The quest for more stable and inexpensive ligands spurred the interest for bidentate heterodonor P,N(sp^3^)-ligands. In most examples either amines^260−275^ or amides^276−281^ were used as N-donor groups and most studies focused on reactions of the benchmark linear substrate with dimethyl malonate as nucleophile (see Scheme 77). The low selectivity of this class of ligand has been attributed to the low stereoselective coordination of the N-sp^3^ group, which leads to the formation of diastereoisomeric mixtures of catalytic species. This has been overcome by three main strategies. One relies on the appropriate tuning of the ligand backbone and the amine substituent. For instance, Petit’s group developed a simple N-phosphine-amino ligand **L80** that provided high *ee* values (up to 95%) in the allylic alkylation of *rac*-1,3-diphenylallyl acetate with dimethyl malonate (Scheme 77).^261^ Similar enantioselectivities were achieved with phosphine-amino ligand **L81** in the allylation of 1,3-diarylallyl acetates (Scheme 77).^267^ More recently, the phosphine-amino ligand **82**, with a spiro[Indane-1,2′-pyrrolidine] backbone, was reported to provide high enantioselectivities in the alkylation of *rac*-1,3-diphenylallyl acetate with dimethyl malonate (Scheme 77), alkyl alcohols, and amines (*ee* values up to 97%).^270^ Mino’s group described another example of backbone control with the atropoisomeric 1-diphenylphosphino-2-amino ligand **L83** inducing *ee* values of up to 95% in the allylic alkylation of *rac*-1,3-diphenylallyl acetate (Scheme 77).^268,282^ Later on, Miller’s group extended Mino’s design to amides (ligand **L84**; *ee* values up to 92%, Scheme 77).^280^

**Scheme 78:**
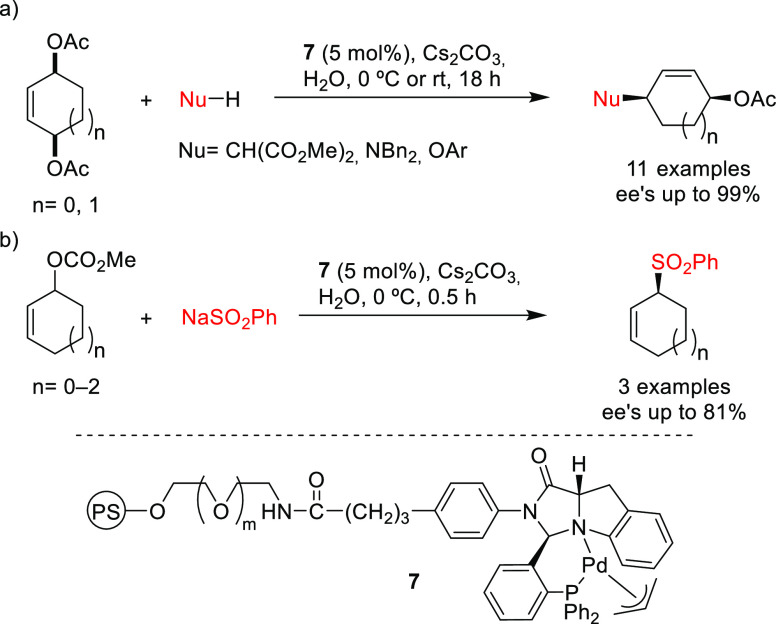
Pd-Catalyzed (a) Desymmetrization of *meso*-1,4-Acetoxy Cyclic Allylic Substrates and (b) Sulfonylation of Cycloalkenyl Carbonates Using Amphiphilic Palladium Complex **7**

A second strategy to favor stereoselective coordination of the N-group relies on introducing chiral substituents at the N-group. High enantioselectivities were achieved in the allylic alkylation of *rac*-1,3-diphenylallyl acetate with dimethyl malonate using chiral oxazolidines^262,266^ and chiral amines from the chiral pool^276,278^ (e.g., ligands **L85**–**L86**, Scheme 77). A remarkable example of an immobilized catalyst of this type is the amphiphilic polystyrene-poly(ethylene glycol) resin-supported chiral imidazoindolephosphine-palladium complex **7**,^283−288^ which was used in the desymmetrization of *meso*-1,4-acetoxy cyclic allylic substrates with various nucleophiles. The reaction proceeded in water under heterogeneous conditions to give the corresponding 1-acetoxy-4-substituted cycloalkenes with enantioselectivities up to 99% *ee* (Scheme 78a).^289^ The catalytic performance of this supported complex was also evaluated in the allylic sulfonylation of cycloalkenyl carbonates, using water as a solvent and sodium phenylsulfinate as a nucleophile. The enantiomeric purity of the corresponding cycloalkenyl sulfones significantly decreased as the reaction time increased (from 71% in 1 h to 10% *ee* in 12 h). This was attributed to the formation of Pd η^3^-allyl intermediates from the chiral allyl sulfones, leading to a partial racemization of the desired product. Decreasing the reaction temperature to 0 °C and the reaction time to 30 min increased selectivity (81% *ee*, Scheme 78b).^290^

**Scheme 79:**
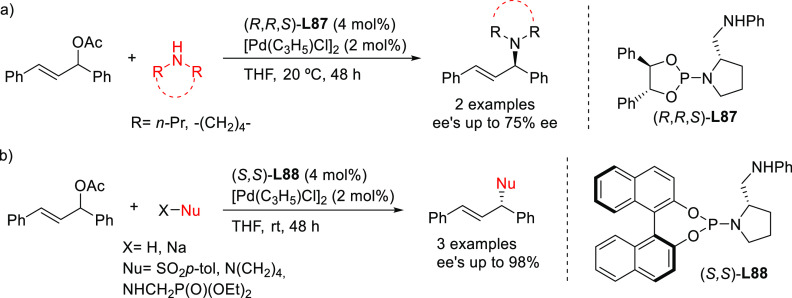
Pd-Catalyzed Allylic Amination of 1,3-Diphenylallyl Acetate Using (a) Pd/(*R*,*R*,*S*)-**L87** and (b) (*S,S*)-**L88** as Catalysts

A third strategy to control the stereoselective coordination of the N-sp^3^ group is based on matching the configurations of the chiral ligand backbone and at the chiral P-donor group. Following this strategy, Gavrilov, Rastorguev and co-workers applied the bidentate phosphoramidite-amine ligand (*R*,*R*,*S*)-**L87** in the allylic amination of *rac*-1,3-diphenylallyl acetate with enantioselectivities up to 75% *ee* (Scheme 79a).^291^ The results further indicated that there is a cooperative effect between the stereogenic centers of the benzoin and pyrrolidine moieties. Another example is ligand (*S,S*)-**L88**, a modification of **L87** in which the hydrobenzoin group has been replaced by a BINOL moiety. Ligand **L88** provided high enantiocontrol in the allylic substitution of *rac*-1,3-diphenylallyl acetate with *para*-toluenesulfinate, pyrrolidine and diethyl aminomethylphosphonate (*ee* values up to 98%; Scheme 79b).^274^

**Scheme 80:**
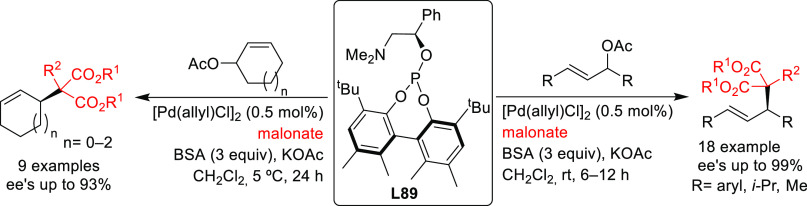
Pd-Catalyzed Allylic Substitution of Disubstituted Linear and Cyclic Substrates with Malonates Using Phosphite-Amine Ligand **L89** R^1^ = Me, Et, Bn; R^2^ = H, Me, allyl, propenyl, propargyl, ...

Other examples include several phosphite/phosphoramidite-amine ligands^271−275^ of which ligand **L89** provided high *ee* values for linear and cyclic disubstituted substrates with several α-substituted malonates (Scheme 80), as well as 1,3-diketones, benzylamines, and electron-poor benzylic alcohols.^273^

**Scheme 81:**
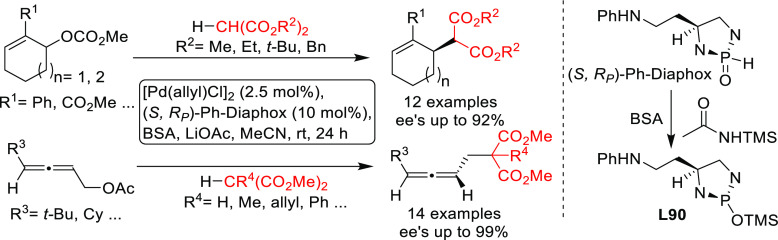
Pd-Catalyzed Allylic Alkylation of (a) 2-Substituted Cycloalkenyl Carbonates and (b) 2,3-Allenyl Acetates with Malonates Using Pd/(*S*,*R*_P_)-Ph-Diaphox Catalyst

Another interesting example is found in the work of Hamada’s group who developed the amine-diaminophosphine oxide (*S,R*_*P*_)-Ph-Diaphox, which is transformed in situ into the diamidophosphite-amine ligand **L90** with the N,O-bis(trimethylsilyl)acetamide (BSA) under the reaction conditions employed.^292,293^ The Pd/**L90** catalyst promoted the allylic alkylation of several 2-substituted cycloalkenyl carbonates with malonates with high enantioselectivities (up to 92% *ee*; Scheme 81a). Pd/**L90** also catalyzed the alkylation of 2,3-allenyl acetates with malonate nucleophiles to yield axially chiral allenes with excellent enantioselectivities (up to 99% *ee*; Scheme 81b).

**Scheme 82:**
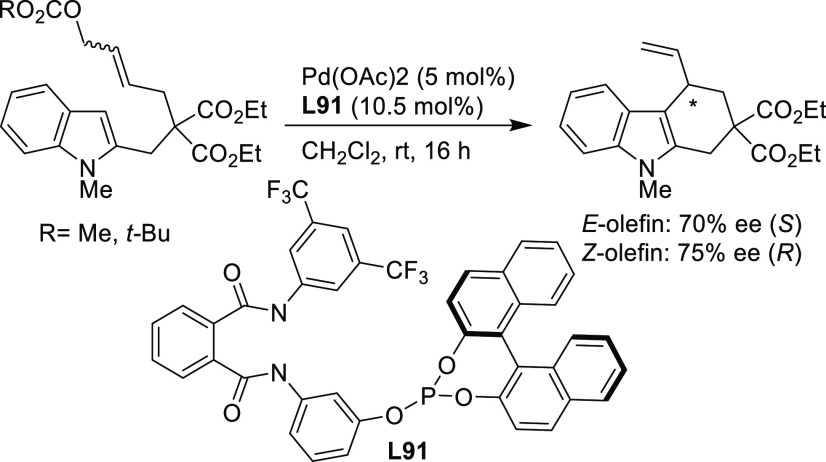
Synthesis of 4-Vinyltetrahydrocarbazole via Intramolecular Pd-Catalyzed Allylic Alkylation

Finally, an example of a ligand that contains a biaryl phosphite group as the only source of chirality is shown in Scheme 82. Pignataro and Gennari’s group demonstrated the utility of ligand **L91** in the intramolecular allylic alkylation to prepare 4-vinyltetrahydrocarbazole.^294^ A range of indole-containing allylic carbonates was cyclized in the presence of the Pd/**L91** catalytic system (*ee* values up to 75%; Scheme 82). Remarkably, the reaction was stereodivergent, so both enantiomers of the 4-vinyltetrahydrocarbazole were accessible by changing the geometry of the substrate double bond.

#### 2.2.8 Bidentate Heterodonor P,S-Ligands

**Figure 6:**
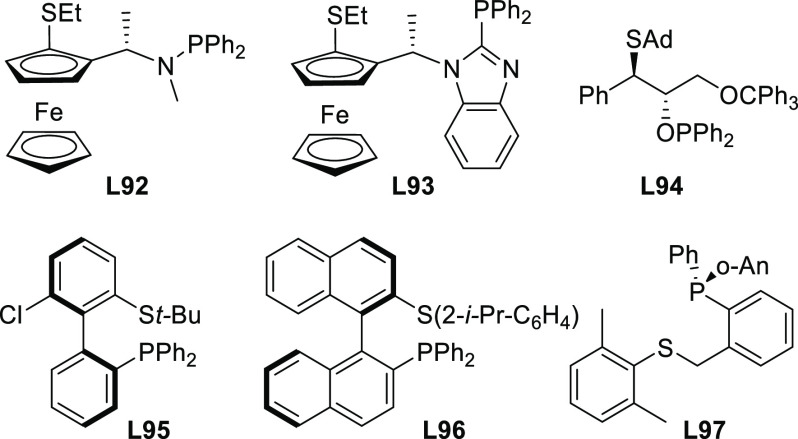
Representative bidentate heterodonor phosphine-thioether ligands **L92**–**L97**.

Research on P,S-ligands was inspired by the remarkable enantioselectivities achieved with Evans’ phosphinite-thioether ligands.^48^ This work encouraged the development of many P-thioether ligand libraries, although only a few of them provided high enantioselectivities and were applicable to diverse substrates.^295−306^ The unsatisfactory results were mainly explained by the fact that the sulfur thioether group becomes a stereogenic center upon coordination, which may lead to diastereomeric mixtures of active species resulting in low enantiocontrol. However, configuration at the coordinating sulfur atom can be controlled with the chirality at the ligand backbone as demonstrated by the development of ligands **L92**–**L97** (Figure 6).

**Scheme 83:**
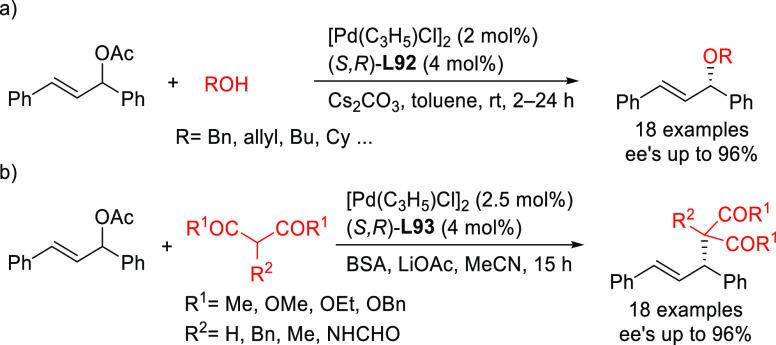
Pd-Catalyzed Allylic Substitution of 1,3-Diphenylallyl Acetate with (a) Aliphatic Alcohols and (b) Malonates Using Ferrocene-Based Phosphine-Amino Ligands **L92** and **L93**

Ligand **L92**, FerroNPS, developed by Chan’s group, is a ferrocene *N*-phosphine-thioether that has been successfully applied in the allylic substitution of *rac*-1,3-diphenlylallyl acetate with a number of less studied O-nucleophiles (*ee* values up to 95.5%; Scheme 83a).^307,308^ The related ligand **L93** proved to be effective in the enantioselective allylation of indoles,^309^ and in the alkylation of 1,3-diphenylallyl acetate and cyclic allylic substrates with a range of malonates (*ee* values up to 96% and 87%, respectively; Scheme 83b).^297^**L92** and **L93** are two of the many ferrocene-based ligands that were developed since Pregosin’s seminal work in 1996 on the use of ferrocene-based P-thioether ligands^310^ in Pd-catalyzed allylic alkylation.^306^ Although the problem of substrate and nucleophile scope in Pd-catalyzed allylic alkylation was not fully solved with ligands **L92** and **L93**, the promising results with substrates and nucleophiles other than 1,3-diphenylallyl acetate and malonate indicated considerable potential for *P*-thioether ligands.

**Scheme 84:**
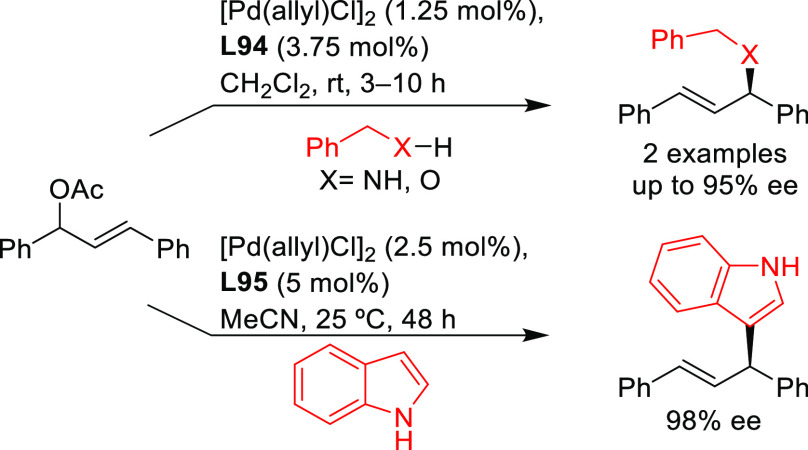
Pd-Catalyzed Allylic Substitution of 1,3-Diphenylallyl Acetates Using Benzyl Amine, Alcohol, and Indoles with Pd/**L94** and Pd/**L95** as Catalysts

Enantioselectivities of up to 99% were obtained with ligands **L94** and **L95**, that have a remarkably simple backbone, in the allylic substitution of hindered *rac*-1,3-diarylallyl acetates and trisubstituted substrates with dimethyl malonate, indoles, N-nucleophiles and O-nucleophiles (Scheme 84).^298,300^

**Scheme 85:**
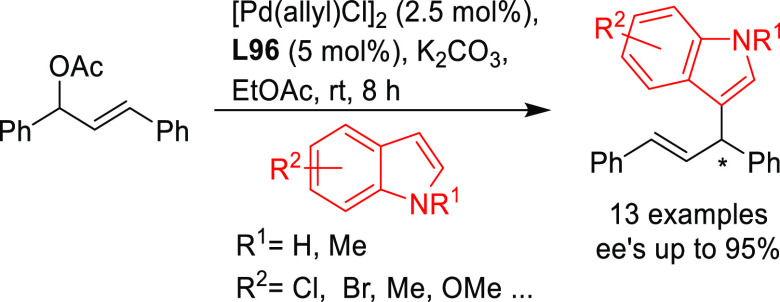
Pd-Catalyzed Allylic Substitution of 1,3-Diphenylallyl Acetate Using an Array of Indoles with Pd/**L96** as Catalyst

Despite extensive use of 1,1′-binaphthalene-based ligands in asymmetric catalysis, only the group of Hoshi, Hagiwara, and co-workers reported new applications of binaphthyl-based *P*-thioether ligands with a chirality axis as the unique stereogenic element.^311^ Pd/**L96** proved to be an efficient catalyst for the Pd-catalyzed allylic alkylation of 1,3-diphenylallyl acetate with indoles (Scheme 85). The presence of the sulfur substituent 2-*i*-Pr-C_6_H_4_ was crucial to achieve optimal enantioselectivity. The authors also demonstrated that this ligand compares well with MeO-MOP, BINAP, and DACH-Trost type ligands for this transformation.

Jugé’s group showed that a ligand with an achiral backbone and a stereogenic phosphine group (ligand **L97**) can provide the same levels of enantioselectivity as ligands **L93–L95** (Figure 6) in the allylic akylation of *rac*-1,3-diphenylallyl acetate using dimethyl malonate as nucleophile (*ee* values up to 96%).^301^

**Figure 7:**
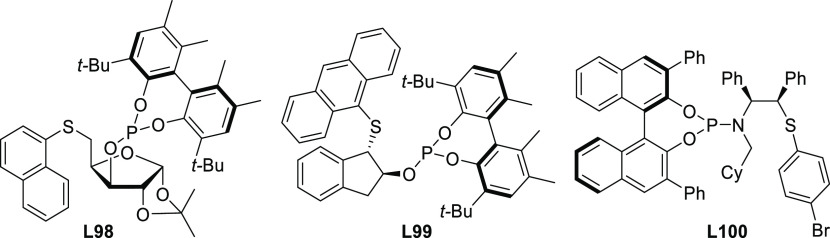
Thioether-phosphite/phosphoramidite ligands **L98–L100** applied in Pd-catalyzed allylic substitution reactions.

Efficient control of the configuration of the S-atom was also achieved by combining an appropriate ligand scaffold with a chiral biaryl phosphite^299,302−304^ or phosphoramidite^312,313^ group as illustrated by three of these families of ligands **L98**, **L99**, and **L100** (Figure 7).

**Scheme 86:**
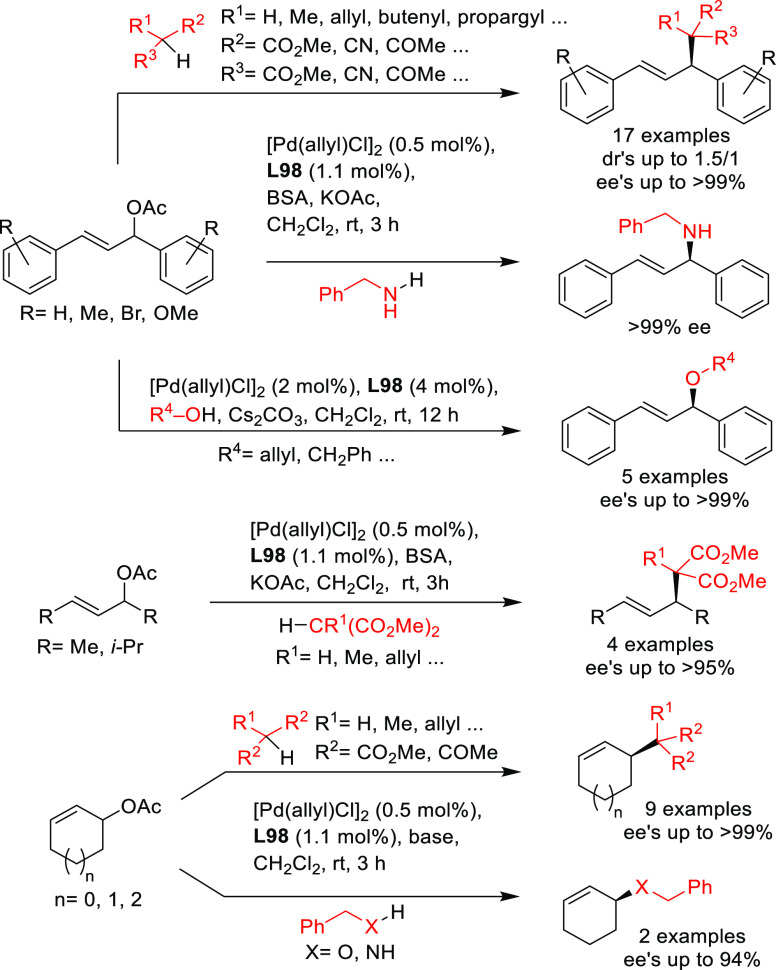
Pd-Catalyzed Allylic Substitution of Disubstituted Linear and Cyclic Substrates with C-, N-, and O-Nucleophiles Using Phosphite-Thioether Ligand **L98**

Furanoside-ligand **L98** was found to be optimal after screening many ligand parameters (configuration of C3, position of the thioether group at either C5 or C3 of the furanoside backbone, and substitution and configuration of the biaryl moiety and the thioether substituent).^299,302^ High enantioselectivities were obtained with hindered and unhindered disubstituted substrates (cyclic and linear), using C-nucleophiles, such as malonates, diketones, and cyanoesters, N-nucleophiles, and O-nucleophiles (with *ee* values up to >99%, Scheme 86). Of particular note are the excellent enantioselectivities of the etherification of linear and cyclic substrates, being the first examples of successful etherification of both substrate types. A mechanistic study of the Pd η^3^-allyl intermediates by NMR spectroscopy and DFT helped to understand the effect of the structural parameters of the ligand on catalytic performance (see section 2.4 for mechanistic details).

**Scheme 87:**
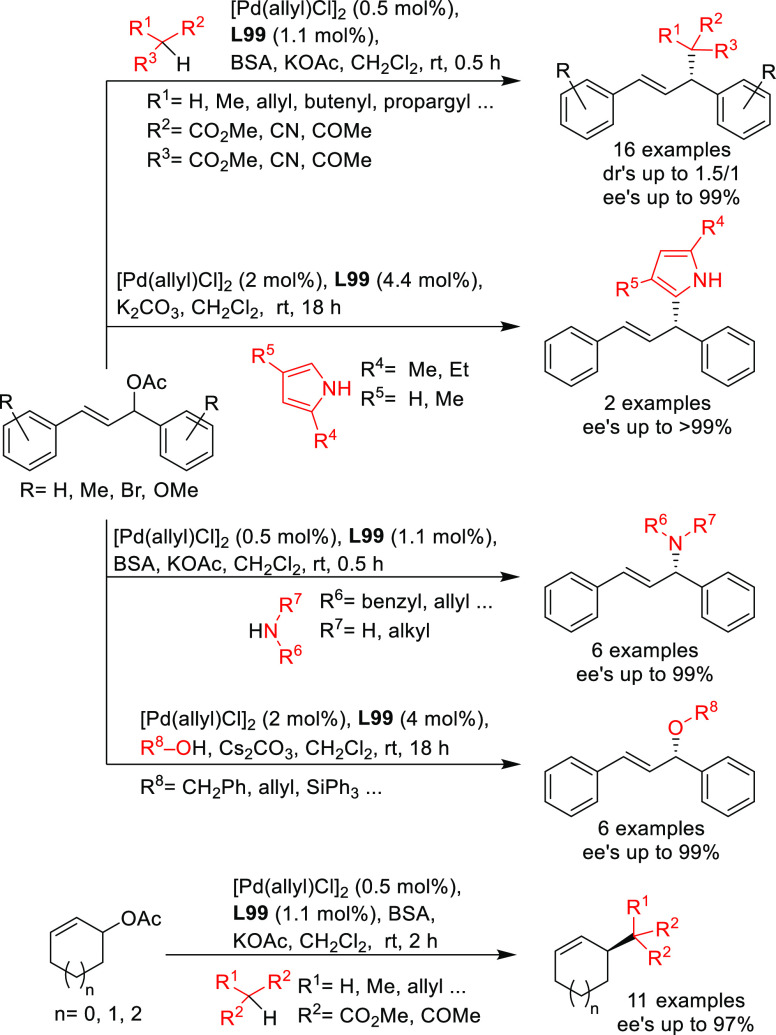
Pd-Catalyzed Allylic Substitution of Disubstituted Linear and Cyclic Substrates with C-, N-, and O-Nucleophiles Using Phosphite-Thioether Ligand **L99**

Later, it was found that the adaptable furanoside backbone in ligand **L98** was not needed to induce high *ee* values and that a simpler and modular indene backbone could be used (e.g., ligand **L99**).^303^ The modular structure of indene derivatives facilitated an iterative optimization of the ligand to adapt the size of the chiral cavity to a specific substrate type. In addition, the simple backbone simplified the NMR spectra, which facilitated studies of intermediates and accelerated DFT calculations. Conclusions from experimental data and DFT calculations led to the development of the anthracenethiol-derived phosphite-thioether ligand **L99** (Figure 7; see section 2.4 for mechanistic details) that provided excellent enantioselectivities for 40 compounds, including linear (un)hindered and cyclic substrates and a broader range of C-, N-, and O-nucleophiles, improving the scope over the Pd/**L99** catalyst (Scheme 87). A variety of allyl-, butenyl-, pentenyl-, and propargyl-substituted malonates reacted with 1,3-diarylallyl acetates to provide the substituted products in high yields with excellent enantioselectivities (up to 99% *ee*). Allylation of pyrroles, primary and secondary amines, and aliphatic alcohols also provided high enantioselectivities (*ee* values up to 99%).

**Scheme 88:**
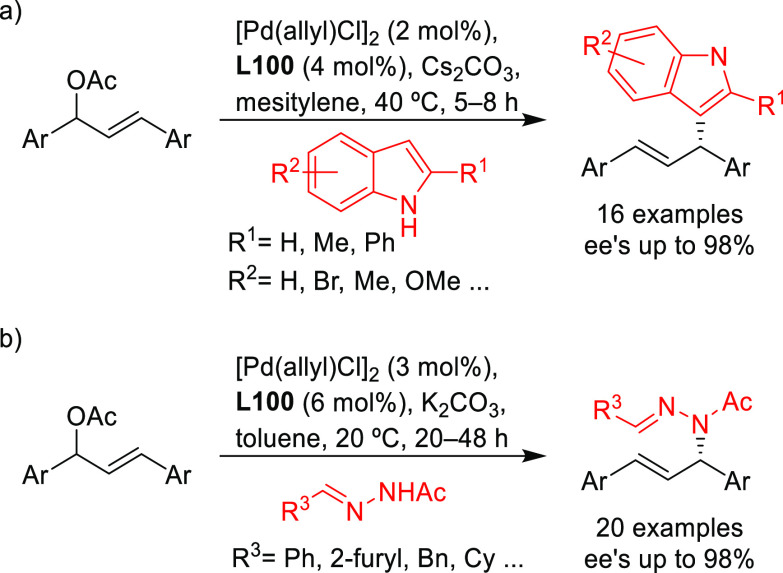
Pd-Catalyzed Allylic Substitution of 1,3-Diarylallyl Acetates with a Range of (a) Indoles and (b) Hydrazones Using Pd/**L100** as Catalyst

A third ligand family is represented by the novel phosphoramidite-thioether ligand **L100** that provided excellent enantioselectivities (up to 98% *ee*) in the allylic alkylation of 1,3-diarylallyl acetates with indoles and hydrazones (Scheme 88).^312,313^ High *ee* values (up to 98%) were also achieved in the allylic substitution of *rac*-1,3-diphenylallyl acetate with benzyl amine and benzyl alcohol as nucleophiles.^312^

**Figure 8:**
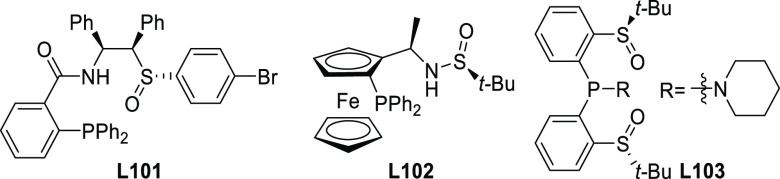
Representative phosphine-sulfoxide/sulfonamide ligands successfully applied in Pd-catalyzed allylic substitutions.

Another strategy to tackle the problem of controlling the configuration of the coordinated thioether group is based on the use of chiral sulfoxides or sulfonamides as structural elements instead of thioether groups (Figure 8).^52,314−320^

**Scheme 89:**
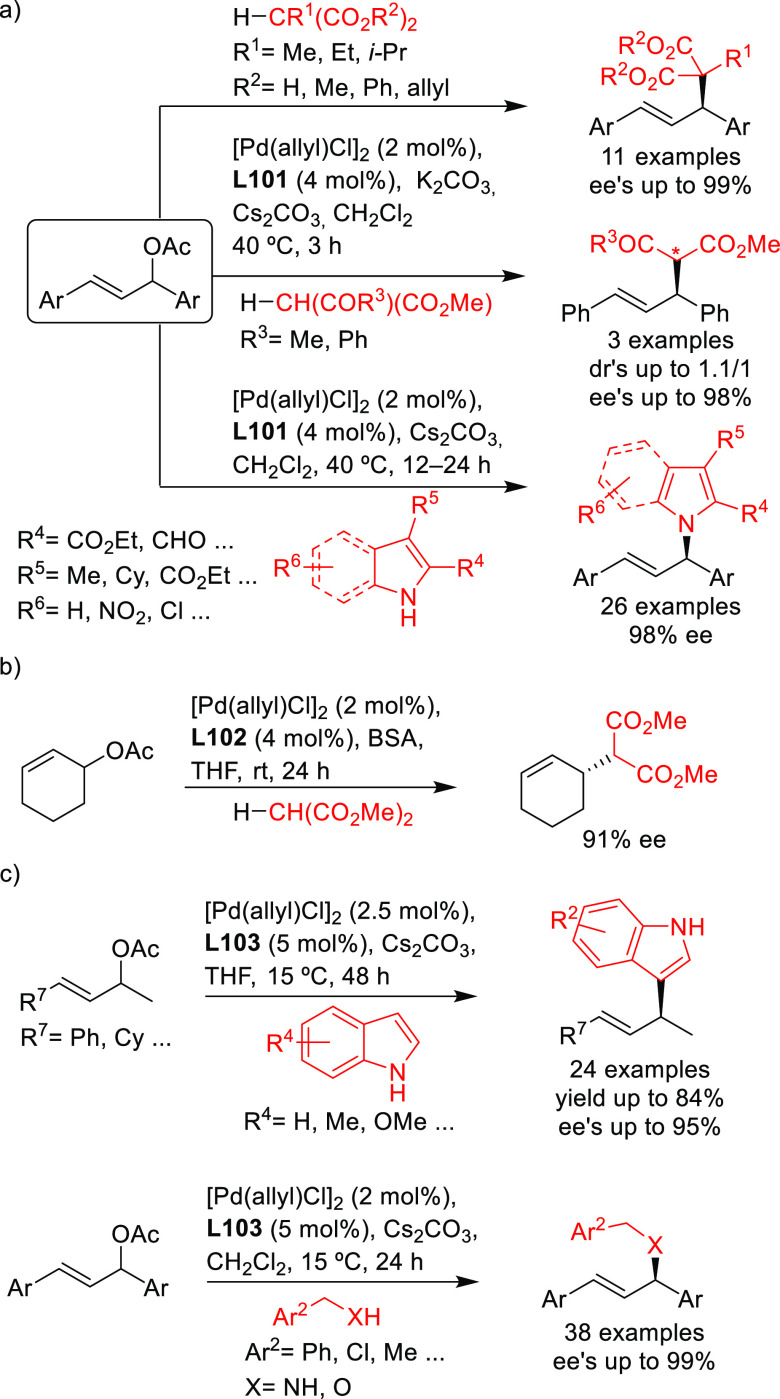
Representative Results for the Pd-Catalyzed AAA Using (a) Phosphine-Sulfoxide **L101**, (b) Phosphine-Sulfonamide **L102**, and (c) Bis(Sulfoxide)phosphine **L103** Ligands

As an example, the phosphine-sulfoxide ligand **L101** provided excellent enantiocontrol in the allylic alkylation of several 1,3-diarylallyl acetates with a range of malonates, including examples with different functionalities at the α-position, as well as ketoesters and amines (Scheme 89a).^315,318^ The ferrocene-based phosphine-sulfonamide ligand **L102** also induced *ee* values of up to 91% in the allylic alkylation of both 1,3-diarylallyl- and cyclohexenyl acetates (Scheme 89b).^316^ Another notable example is the bis(sulfoxide)phosphine ligand **L103** (Scheme 89c)^52^ that promoted the Pd-catalyzed dynamic kinetic resolution of racemic unsymmetrically 1,3-disubstituted allylic acetates with indoles. The unique stereocontrol of this catalytic system was explained by the presence of the two sulfoxide moieties, which play a distinct role in the reaction: one coordinates to Pd and the other acts as a hydrogen bond acceptor, directing nucleophilic attack of the indole by hydrogen bonding. Ligand **L103** was also successfully employed in the allylic amination and etherification of diaryl-substituted allylic acetates with benzylic amines and alcohols (up to 99% *ee*; Scheme 89c).^319^ The authors highlighted the bifunctional nature of the ligand displaying both Lewis and Brønsted basicity. Its function as a Brønsted base was supported by ^1^H NMR spectroscopic studies that showed a hydrogen bond interaction between the *tert*-butyl sulfinyl group and the amine/alcohol substrate.

#### 2.2.9 Bidentate Heterodonor P,Olefin-Ligands

**Figure 9:**
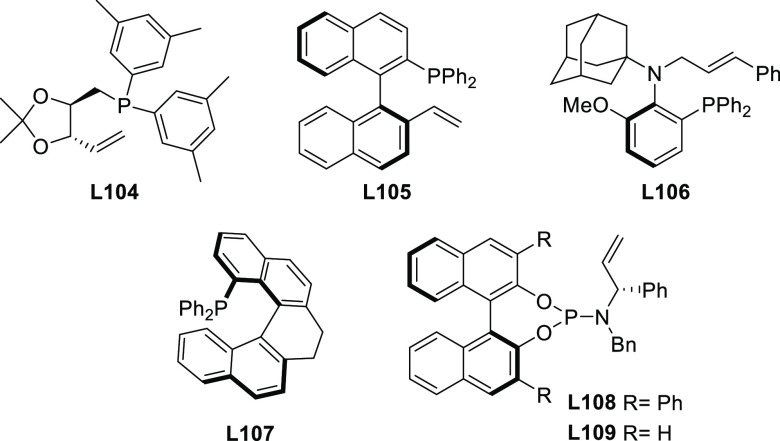
Representative P-alkene ligands successfully applied in Pd-catalyzed allylic substitutions.

Since the successful application of Hayashi’s norbornene-based phosphine-olefin ligands in Pd-catalyzed allylic substitution,^321^ heterodonor P,olefin-ligands have emerged as a promising ligand class for this transformation. The field has been dominated by phosphine-olefin ligands,^322−328^ although phosphinite-^329^ and phosphoramidite-olefin^330,331^ ligands have also been used (Figure 9).

**Scheme 90:**
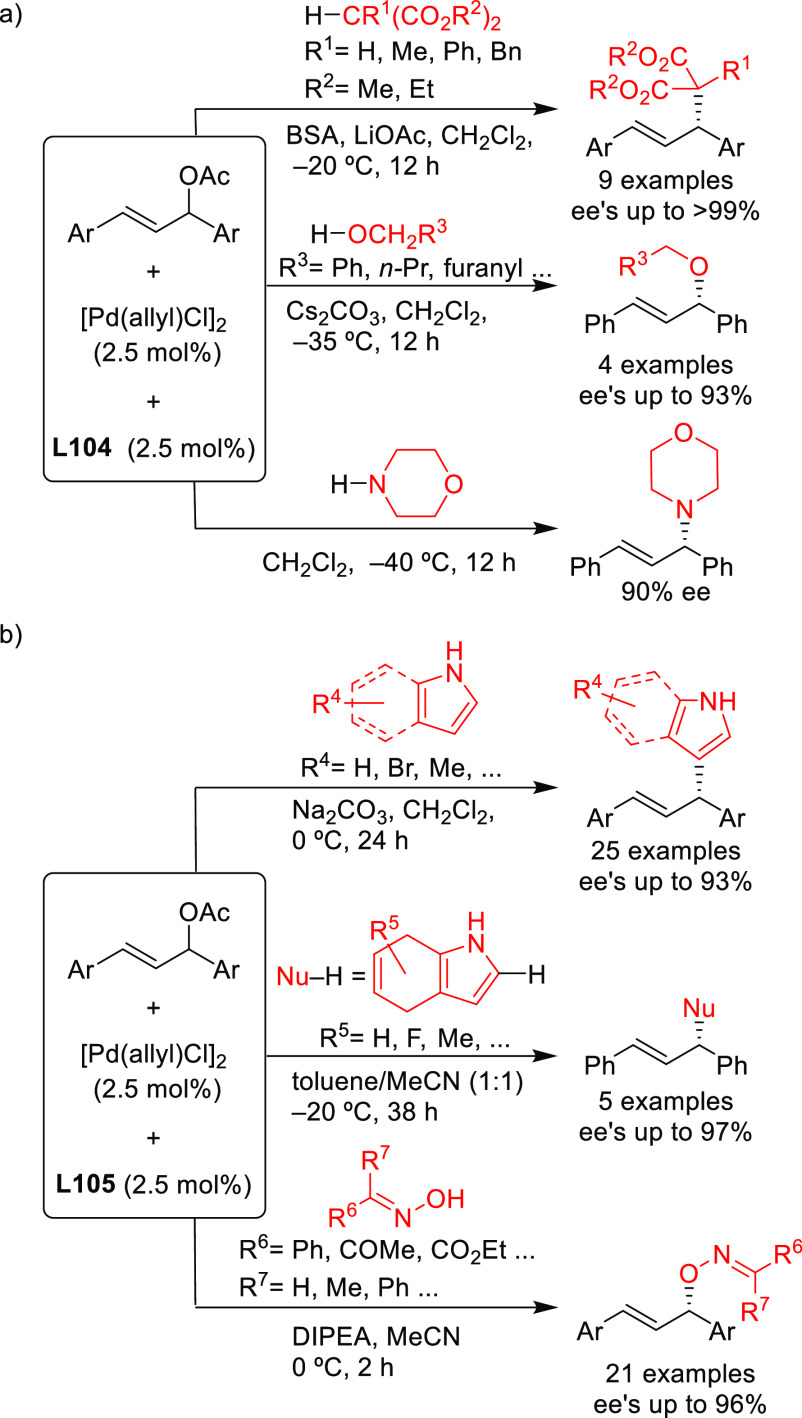
Representative Results for Pd-Catalyzed Allylic Substitution Using (a) Pd/**L104** and (b) Pd/**L105** Catalytic Systems

Du’s group successfully developed phosphine-olefin ligands **L104** and **L105** for the allylic substitution of *rac*-1,3-diarylallyl acetates (Scheme 90).^323,325−327^ Whereas the Pd/**L104** catalyst provided excellent enantioselectivities with a range of malonates, alkyl alcohols and morpholine (Scheme 90a),^323^ the Pd/**L105** catalyst induced very high *ee* values in reactions with indoles, pyrroles and 4,7-dihydroindoles as C-nucleophiles^325,326^ as well as oximes as O-nucleophiles^327^ (Scheme 90b).

**Scheme 91:**
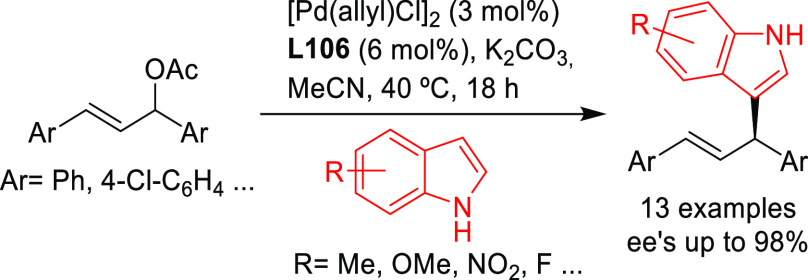
Pd-Catalyzed AAA of *rac*-1,3-Diarylallyl Acetates with Several Indoles Using the Pd/**L106** Catalytic System

Mino’s group modified the P,N(sp^3^)-ligand **L84** by introducing an olefinic donor group. One of these modified ligands, the *N*-1-adamantyl-*N*-cinnamylaniline derivative **L106** was found to induce high enantioselectivities in the allylic alkylation of *rac*-1,3-diarylallyl acetates with a range of indoles (Scheme 91).^328^

**Scheme 92:**
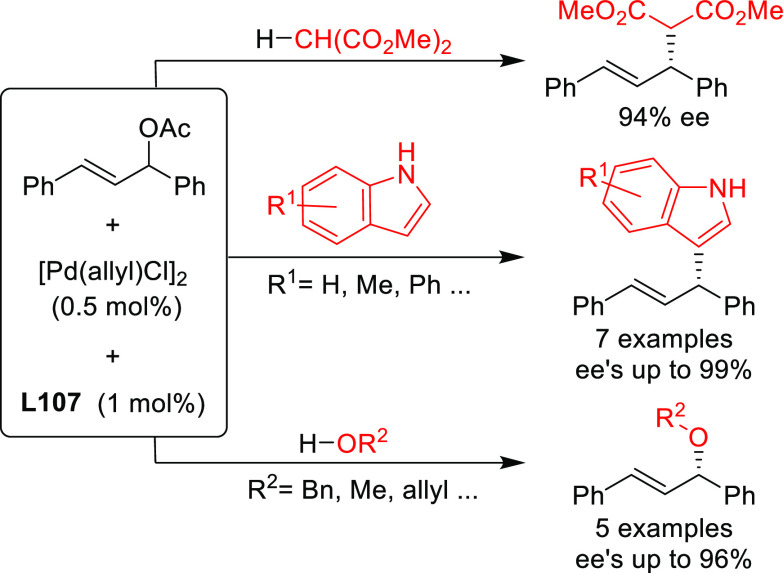
Pd-Catalyzed Allylic Substitution of *rac*-1,3-Diphenylallyl Acetate with a Range of Nucleophiles Using Pd/**L107** Catalyst Reactions carried out using Cs_2_CO_3_ as base and CH_2_Cl_2_ as solvent at rt.

Yamamoto and co-workers developed a helicene-derived phosphine-olefin ligand **L107**(324) that exerted efficient enantiocontrol (*ee* values up to >99%) in the allylic alkylation of *rac*-1,3-diphenylallyl acetate with dimethyl malonate, indoles and alkyl alcohols (Scheme 92).

**Scheme 93:**
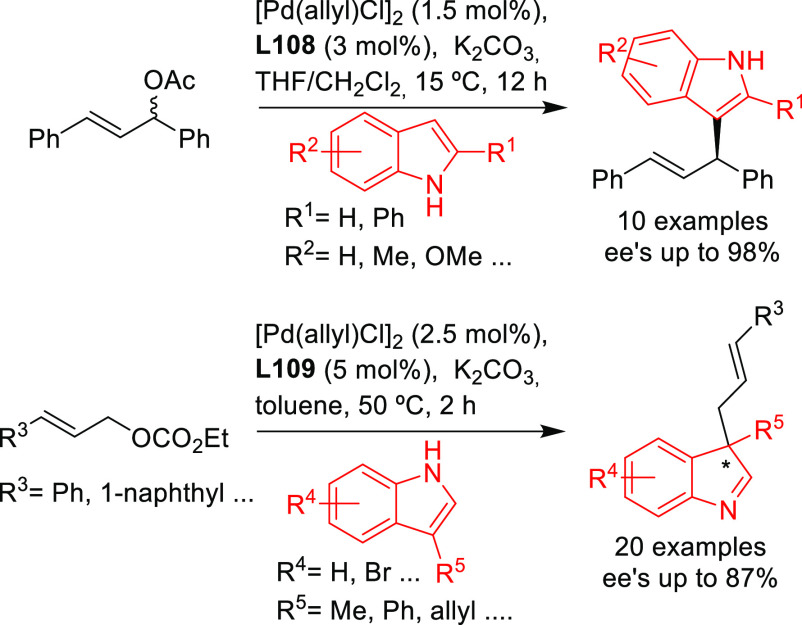
Representative Results for the Pd-Catalyzed Alkylation with Indoles Using Phosphoramidite-Olefin Ligands **L108** and **L109**

Du’s group reported the allylic alkylation of *rac*-1,3-diphenylallyl acetate and several cinnamyl type carbonates with 3-unsubstituted and 3-substituted indoles using Pd/phosphoramidite-olefin ligands **L108** and **L109** (Scheme 93).^330,331^ A variety of indolenines containing a tertiary or a quaternary carbon stereocenter were obtained in high yields with excellent enantioselectivitities (up to 98% *ee*; Scheme 93). They also showed that Pd/**L108** can be used in the allylic amination of *rac*-1,3-diphenylallyl acetate with a set of alkyl amines and hydroxylamine hydrochloride (*ee* values up to 95% *ee*).^330^

#### 2.2.10 Bidentate Heterodonor N,N′-Ligands

**Scheme 94:**
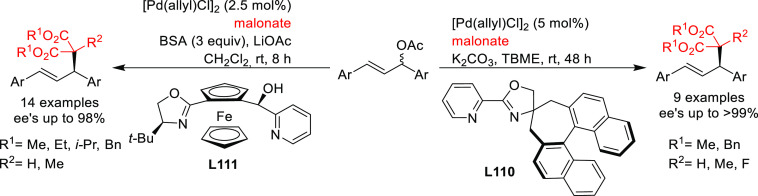
Pd-Catalyzed Allylic Alkylation of Disubstituted 1,3-Diarylallyl Acetates with Malonates Using Pyridine-Oxazoline Ligands **L110** and **L111**

A variety of heterodonor ligands with two different N-donor groups have been used, such as amine-imine, pyridine-amine, pyridine-imine ligands,^332−336^ and pyridine-oxazolines^337,338^ as a particularly effective ligand class. Scheme 94 highlights the high enantioselectivities obtained in the allylic alkylation of some 1,3-diarylallyl acetates with malonates using Pd/**L110**^337^ and Pd/**L111** as catalysts.^338^ However, higher catalyst loadings and/or longer reaction times were required in this case to achieve full conversion than with catalysts based on P-containing ligands. A further drawback is the poor conversion and enantioselectivity observed with other disubstituted and monosubstituted substrates.

#### 2.2.11 Bidentate Heterodonor N,S/Se-Ligands

**Scheme 95:**
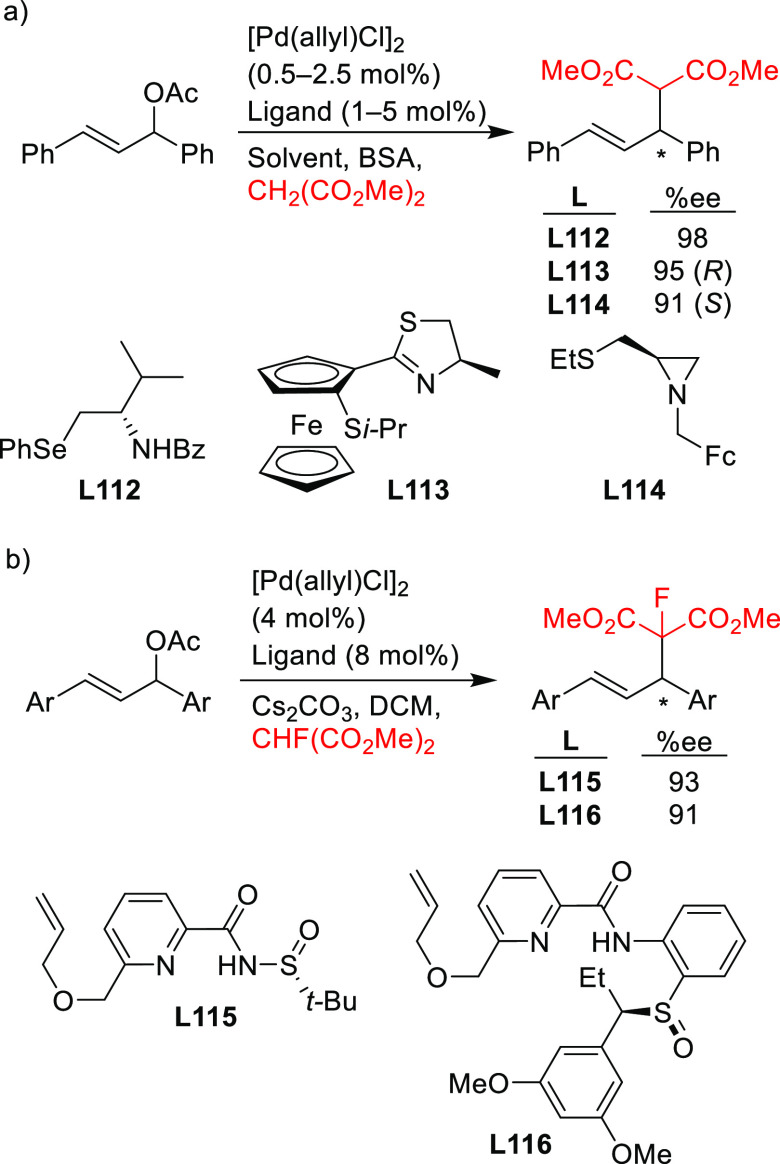
Representative Examples of Bidentate Heterodonor N,S/Se-Ligands Applied in the Allylic Alkylation of *rac*-1,3-Diarylallyl Acetates with (a) Dimethyl Malonate Using Ligands **L112**–**L114** and (b) Dimethyl 2-Fluoromalonate Using Ligands **L115** and **L116**

Heterodonor N,S/Se-ligands with a variety of donor group combinations (e.g., imine-thioether, oxazoline-sulfoxide, thiazoline-thioether) have also been studied.^339−351^ In most cases, they provided only moderate enantioselectivities. As a notable exception, up to 98% *ee* was obtained with the simple amine-selenoether ligand **L112** in the alkylation of *rac*-1,3-diphenylallyl acetate with dimethyl malonate (Scheme 95a).^340,341^ Similar enantioselectivities were achieved using the ferrocene-based thiazoline-thioether ligands **L113** and **L114** (Scheme 95a).^342,349^ X-ray and NMR spectroscopic studies of the π-allyl intermediates indicated that the sulfur atom was a stronger π-acceptor than the nitrogen atom. Furthermore, the pyridine-sulfonamide ligand **L115**(346) and pyridine-sulfoxide ligand **L116**(350) were successfully applied in the alkylation of *rac*-1,3-diarylallyl acetates with dimethyl 2-fluoromalonate (Scheme 95b). Pd/**L116** was also an effective catalyst for the reaction with ethyl 2-fluoro-3-oxobutanoate as nucleophile, producing the allylation products in high *ee* values (up to 98%), although diastereoselectivities were low (1.2:1 dr).

#### 2.2.12 Miscellaneous Ligands

**Scheme 96:**
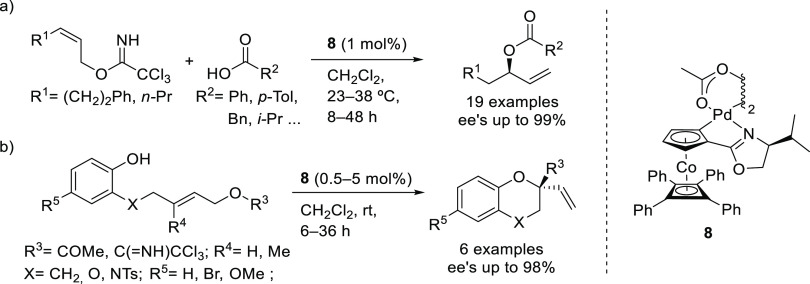
Synthesis of (a) Branched Allylic Esters and (b) 2-Vinyl Oxygen Heterocycles Using C,N-Palladacycle [(*R*_p_,*S*)-COP-OAc]_2_**8** as Catalyst

In 2010, Overman and co-workers studied the enantiopure C,N-palladacycle [(*R*_p_,*S*)-COP-OAc]_2_**8** as catalyst in the synthesis of branched allylic esters from (*Z*)-2-alkenyl trichloroacetimidates and carboxylic acids.^352^ The reaction led to the branched products with perfect regioselectivity (b/l ratios higher than 800) and high enantioselectivity (*ee* values up to 99%) with a variety of carboxylic acids (Scheme 96a). Remarkably, the authors reported a one-pot procedure for the in situ preparation of the trichloroimidate intermediate and its use in the enantioselective allylic substitution reaction from (*Z*)-2-alkene-1-ols. Subsequently, the same group developed a new type of air- and moisture-stable enantiopure C,N-palladacycles with an imidazoline-naphthalene backbone as catalysts for the same transformation but they provided lower enantioselectivities than palladacycle **8**.^353^ Interestingly, computational studies indicated that the alkene π-bond of the allylic imidate substrate is preferentially coordinated *cis* to the carbon ligand of the palladacycle with attack of an external nucleophile occurring from the least sterically hindered face in this quadrant (for mechanistic details see section 2.4). This suggests that introducing substituents in the vicinity of the carbon donor atom could be a good way to improve the *ee* values. Shortly after, the same group disclosed a further application of **8** as a catalyst for the enantioselective synthesis of 2-vinyl oxygen heterocycles through intramolecular allylic etherification of phenolic trichloroimidates and acetates (*ee* values up to 98%; Scheme 96b).^354^

**Scheme 97:**
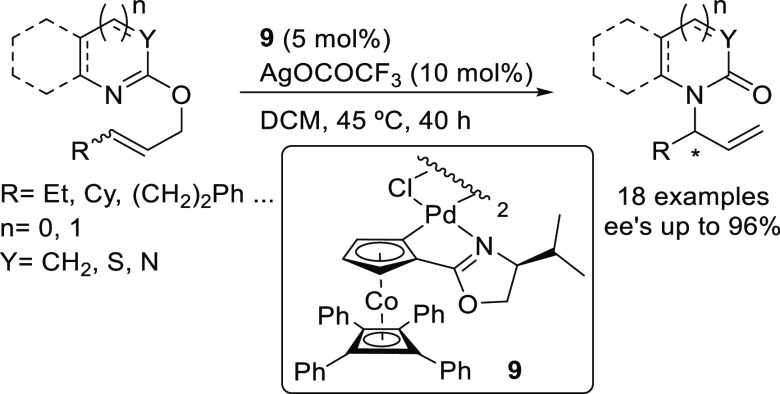
Synthesis of Enantioenriched Heterocycles via Formal [3,3]-Sigmatropic Rearrangement Using C,N-Palladacycle [(*R*_p_,*S*)-COP-Cl]_2_**9** as Catalyst

Batey and co-workers used the C,N-palladacycle [(*R*_p_,*S*)-COP-Cl]_2_**9** as a catalyst for the formal [3,3]-sigmatropic rearrangement of 2-allyloxypyridines and other heterocycles through Pd-catalyzed allylic amination using silver(I) trifluoroacetate as a cocatalyst.^355^ A range of enantioenriched heterocycles, such as allylic 2-pyridones, benzothiazolones, quinolinones, and isoquinolinones, were prepared in high enantiomeric excesses (up to 95% *ee*; Scheme 97).

**Scheme 98:**
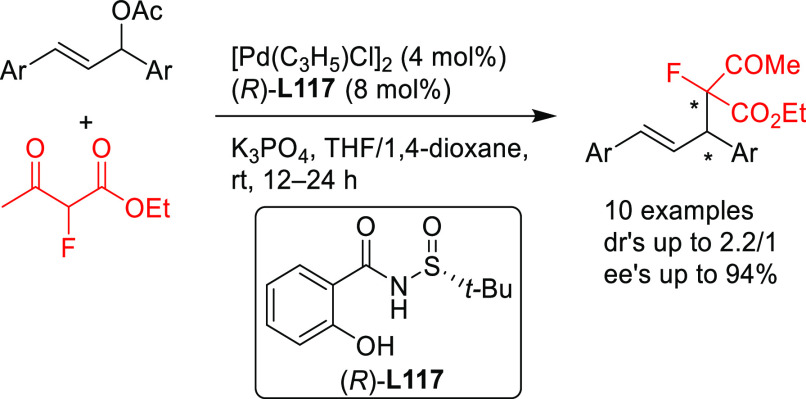
Synthesis of Fluorinated Allyl Compounds Using Pd/**L117** as Catalyst

Zhao’s group developed a new chiral sulfonamide ligand **L117**, synthesized from salicylic acid and (*R*)-*tert*-butanesulfinamide, which exerted efficient enantiocontrol in the Pd-catalyzed allylic alkylation of several 1,3-diarylallyl acetates with ethyl 2-fluoroacetoacetate (Scheme 98).^356^ The corresponding monofluorinated allylic compounds were obtained with moderate diasteoselectivities but high enantioselectivities (dr’s up to 2.2/1 and *ee* values up to 95%).

**Scheme 99:**
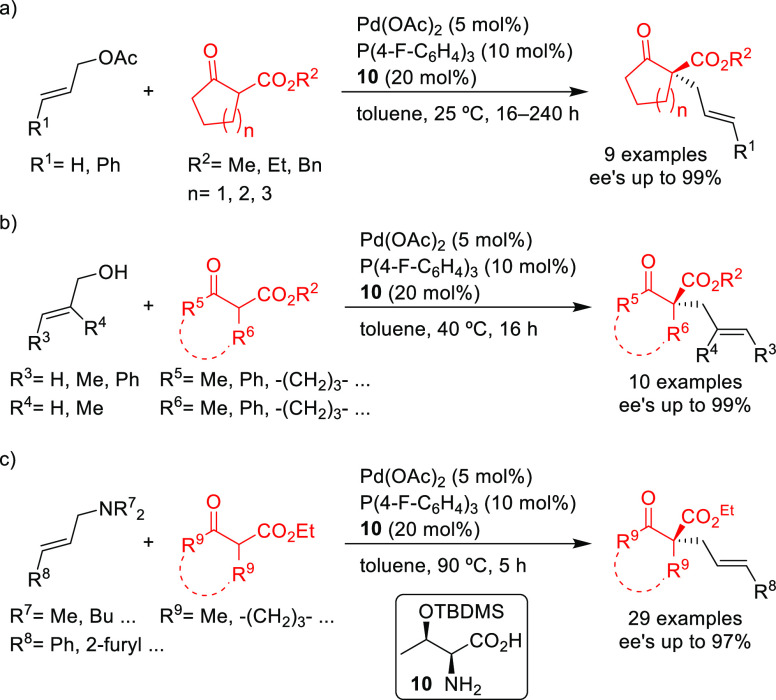
Synthesis of α-Allylated β-Ketoesters Containing a Quaternary Carbon Stereogenic Center by Synergistic Chiral α-Amino Acid Catalysis and Pd-Catalyzed Allylic Alkylation

Yoshida’s group developed an efficient new protocol for the enantioselective allylation of α-substituted ketoesters with allylic acetates^357^ (Scheme 99a) and allylic alcohols^358^ (Scheme 99b) by synergistic catalysis between an achiral palladium complex and a chiral primary amino acid **10**. Various α-allylated β-ketoesters containing a quaternary carbon stereogenic center were synthesized in high enantioselectivities (*ee* values up to 99%). Later, Tian’s group extended this protocol to allylic amines as substrates (Scheme 99c).^359^

**Scheme 100:**
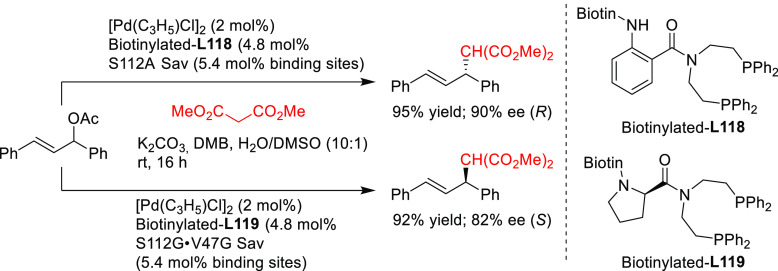
Asymmetric Allylic Alkylation Using Artificial Metalloenzymes Based on the Biotin-Avidin Technology

During this period, Pd complexes, embedded into enzymes, DNA or antibodies, were also studied as catalysts for enantioselective allylic substitutions. Ward’s group demonstrated the potential of artificial biotin–avidin-type metalloenzymes in the allylic alkylation of *rac*-1,3-diphenylallyl acetate with dimethyl malonate in the presence of didodecyldimethylammonium bromide (DMB) as surfactant to avoid hydrolysis of the starting acetate.^360^ By proper selection of the biotinylated diphosphine and the mutated avidin, both enantiomers of the alkylated product could be obtained (Scheme 100).

**Scheme 101:**
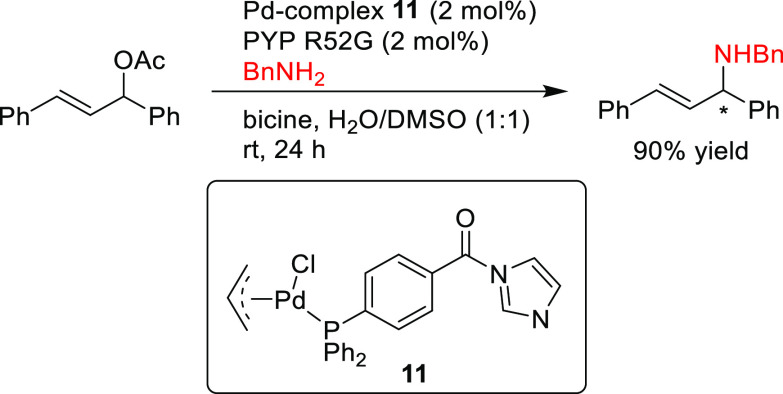
Allylic Amination Using Artificial Metalloenzymes Based on Photoactive Yellow Protein

Later, Kamer’s group developed artificial metalloenzymes using the photoactive yellow protein (PYP) as a chiral second coordination sphere.^361^ The covalent linkage was formed though cysteine-selective conjugation of the Pd-complex bearing a phosphine ligand with a reactive imidazole unit (complex **11**) to the unique cysteine of the protein, Cys69. The hybrid catalyst showed good activities in the allylic amination of *rac*-1,3-diphenylallyl acetate with benzylamine, although *ee* values were low (Scheme 101).

**Scheme 102:**
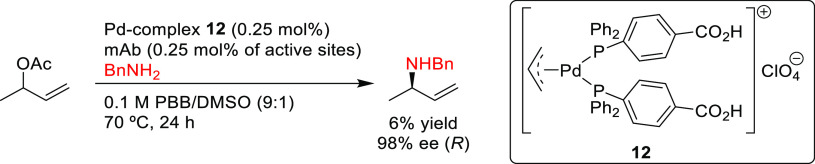
Enantioselective Allylic Amination Using an Artificial Metalloenzyme Based on Monoclonal Antibody (mAb)

More recently, Harada, Yamaguchi, and co-workers studied a hybrid catalyst, in which the Pd-complex **12** is embedded in the chiral pocket of a monoclonal antibody (mAb) through supramolecular interactions, for the allylic amination of but-3-en-2-yl acetate with benzylamine (Scheme 102).^362^ The hybrid catalyst showed excellent enantioselectivities (up to 98% *ee*), albeit with a low conversion.

### 2.3 Other C-Nucleophiles

Simple enolates not stabilized by a π-acceptor group in the β-position are challenging nucleophiles because of the high basicity, which favors side reactions, such as aldolization. Therefore, they have been much less explored than stabilized enolates.^363−370^ Most of the successful examples rely on the use of classic ligands developed for the successful Pd-catalyzed allylic alkylation reactions of malonates and related nucleophiles (e.g., PHOX, SIOCPHOX, and Trost’s ligands). It is, therefore, expected that the design of new chiral ligand specially designed for such transformations and the use of cooperative/dual catalysis methodologies (as demonstrated by Snaddon’s group, vide infra) should spur the development of the highly enantioselective Pd-catalyzed allylic alkylation of these elusive nucleophiles.

**Scheme 103:**
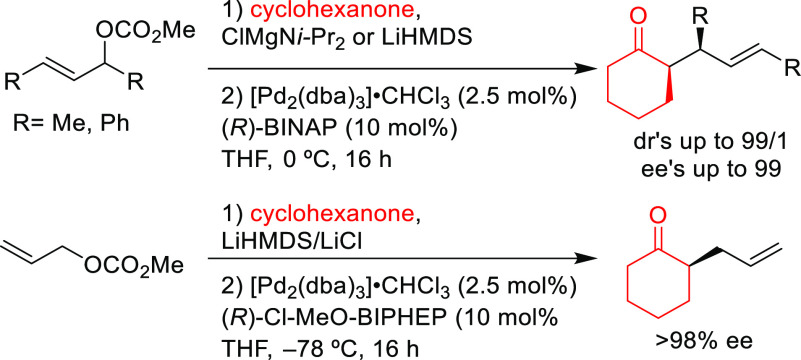
Asymmetric Pd-Catalyzed Desymmetrization of Cyclohexanone

Simple ketones have been rarely used. Among them dialkylketones, containing acidic protons at both α-and α′-positions, pose the additional challenge of regioselectivity. This problem can be avoided by using symmetrical ketones or ketones containing only one substituent with an α-CH bond. Representative examples of the former approach can be found in the desymmetrization of cyclohexanone and bicyclo[3.n.1]-3-one derivatives reported by the groups of Braun and Ding and Hou, respectively. Braun’s group reported the allylation of cyclohexanone using allyl methyl carbonate and disubstituted carbonates. High diastereo- and enantioselectivities were achieved using axially chiral biaryl diphosphines (dr’s up to 99/1 and *ee* values up to 99%; Scheme 103).^371^

**Scheme 104:**
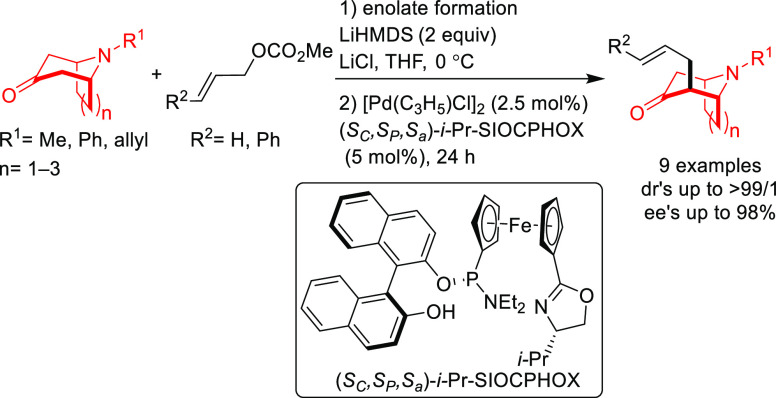
Asymmetric Pd-Catalyzed Desymmetrization of Bicyclo[3.*n*.1]-3-one Derivatives

Ding and Hou’s group later reported the allylation of bicyclo[3.n.1]-3-one derivatives with a range of allylic acetates using (*S*_C_,*S*_P_,*S*_a_)-*i*-Pr-SIOCPHOX as ligand.^372^ This protocol offers access to chiral tropanes containing three stereogenic centers, with high diastereo- and enantioselectivities (dr’s up to >99/1 and *ee* values up to 98%; Scheme 104).

**Scheme 105:**
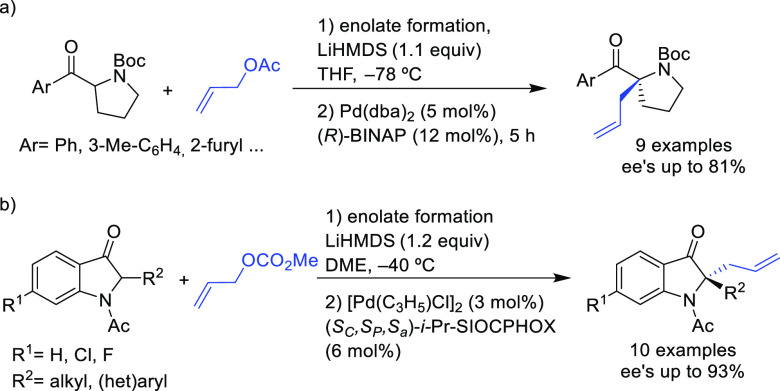
Asymmetric Pd-Catalyzed Allylation of (a) Aryl Pyrrolidyl Ketones and (b) 2-Substituted Indolin-3-ones as Nucleophiles

An example of the allylation of a ketone with only one enolizable position was reported by Zhang and co-workers who used aryl pyrrolidyl ketones as nucleophiles for the synthesis of 2,2-disubstituted pyrrolidines, which were obtained with moderate *ee* values of up to 81% (Scheme 105a).^373^ Similarly, 2-monosubstituted indolin-3-ones were allylated leading to 2,2-disubstituted indolin-3-ones, which are important structural motifs in many natural products (Scheme 105b).^374^

**Scheme 106:**
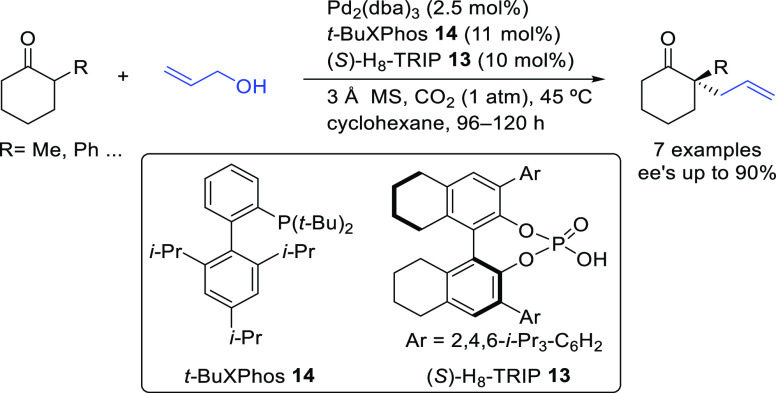
Direct Asymmetric α-Allylation of α-Branched Ketones with CO_2_ and Allylic Alcohol

List reported a direct Pd-catalyzed asymmetric α-allylation of α-substituted ketones with nonactivated allyl alcohols using catalytic amounts of CO_2_ and the chiral phosphoric acid (*S*)-H_8_-TRIP **13** as the enantioselectivity-inducing cocatalyst (Scheme 106).^375^ Allylic alcohols are activated in situ with CO_2_ by conversion into a more reactive carbonic acid ester that readily reacts with the Pd catalyst to form the required Pd η^3^-allyl intermediate. Overall, this is a highly atom-economic process with water as the sole byproduct. The formation of the thermodynamically more stable enol from cylic α-substituted ketones is mediated by chiral phosphoric acid **13**. The sterically hindered *t*-BuXPhos ligand **14** proved to be optimal, giving the quaternary allylated products with α-aryl and α-alkyl substituents in moderate to excellent yields and high enantioselectivities (up to 90% *ee*).

**Scheme 107:**
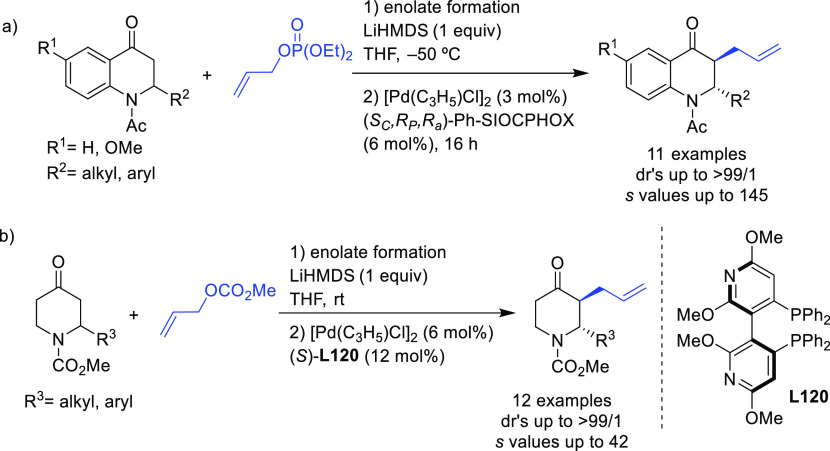
Kinetic Resolution via Pd-Catalyzed Allylic Alkylation of (a) 2-Substituted 4-Quinolones and (b) 2-Substituted 2,3-Dihydro-4-pyridones

Ketone enolates derived from 2-substituted 4-quinolones were kinetically resolved via Pd-catalyzed allylation with high selectivity factors (*s* values up to 145; Scheme 107a).^376^ The value of this transformation was demonstrated by the synthesis of the core structure of the *Martinella* alkaloids. This protocol was further extended to 2-substituted 2,3-dihydro-4-pyridones (*s* values up to 43; Scheme 107b).^377^

**Scheme 108:**
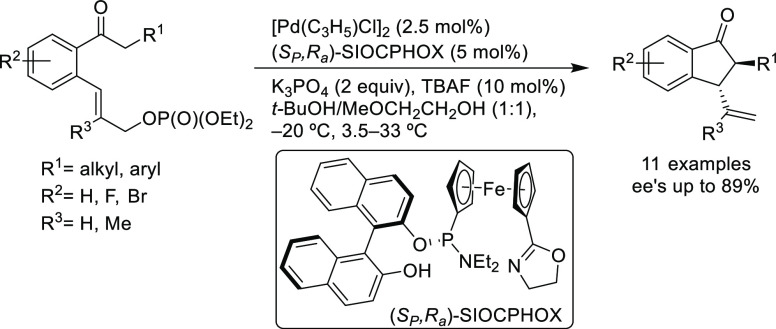
Intramolecular Asymmetric Pd-Catalyzed Allylic Alkylation Using Ketones as Pronucleophiles

An intramolecular version of this transformation was also reported to yield 2,3-disubstituted indanones with high diastereo- and enantioselectivities using Pd/(*S*_P_,*R*_a_)-SIOCPHOX as catalyst (Scheme 108).^378^

**Scheme 109:**
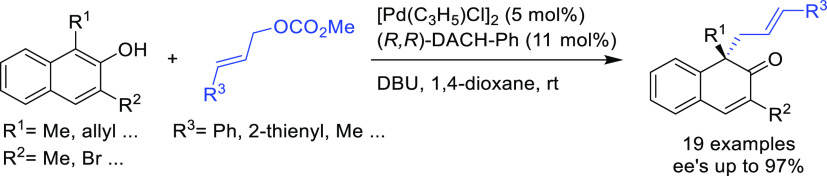
Synthesis of α-Allylated β-Naphthalenones via Asymmetric Pd-Catalyzed Allylic Dearomatization

α-Allylated carbonyl compounds can also be prepared by dearomatization of naphthol derivatives via Pd-catalyzed allylic alkylation using Trost’s (*R,R*)-Ph-DACH ligand as demonstrated by You’s group (Scheme 109).^379^ The resulting dihydronaphthalen-2-ones, bearing a quaternary stereogenic center, were obtained in excellent chemo- and enantioselectivities (*ee* values up to 97%).

**Scheme 110:**
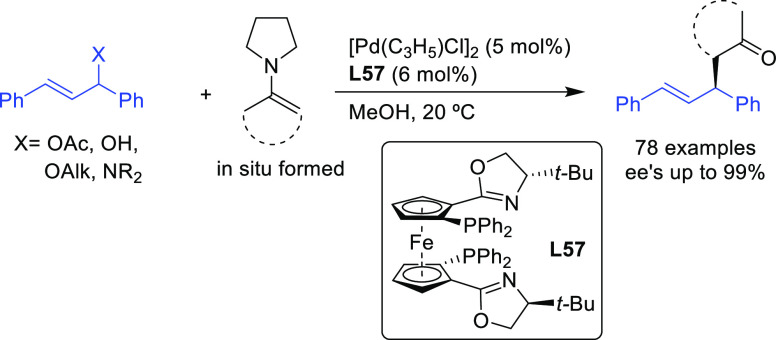
Asymmetric Pd-Catalyzed Allylic Alkylation Using In Situ Formed Enamines as Nucleophiles

As an alternative to enolates, enamines generated in situ from the corresponding ketone and pyrrolidine, may be used as shown by Zhang and co-workers for reactions of acetone and cyclohexanone with a range of substrates (Scheme 110).^380−384^

**Scheme 111:**
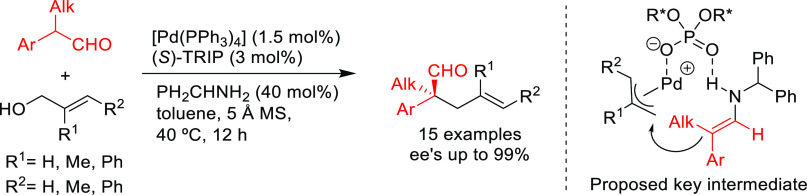
Direct Asymmetric α-Allylation of Aldehydes with Allylic Alcohols

More recently, this approach has been used by the List group in the allylation of α-branched aldehydes with allylic alcohols based on a chiral counteranion-directed strategy. In this case, the asymmetric induction is affected by a chiral phosphate anion, which is proposed to coordinate to the Pd catalyst and at the same time form a hydrogen bond with the enamine in the transition state (Scheme 111). Using a combination of [Pd(PPh_3_)_4_], the chiral Brønsted acid (*S*)-3,3′-bis(2,4,6-triisopropylphenyl)-1,1′-binaphthyl-2,2′-diyl hydrogen phosphate ((*S*)-TRIP) and benzhydrylamine (for the in situ formation of the enamine), high yields and enantioselectivities were obtained.^385^

**Scheme 112:**
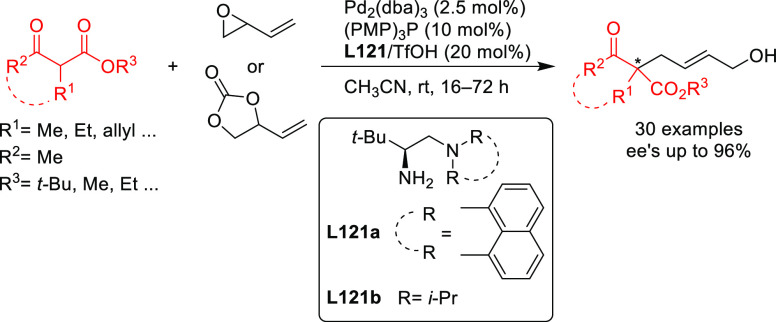
Pd-Catalyzed AAA of Vinyl Epoxides and Vinylethylene Carbonate with Several β-Ketoesters via Enamine Formation with the Chiral Amine Ligands **L121**

Notably, the enamine approach is not only applicable to simple ketones but also to stabilized β-ketocarbonyl compounds as demonstrated by the group of Zhang and Luo.^386^ Key to success was the use of a chiral primary amine **L121a** with an arene substituent that can form a π-complex with the Pd catalyst, while the primary amino group activates the carbonyl compound by enamine formation. The catalyst system containing **L121a** was successfully applied in the allylation of α-branched ketoesters with vinyl epoxide and vinylethylene carbonate (*ee* values up to 96%; Scheme 112). High enantioselectivities were also obtained using [Pd(PPh_3_)_4_], (*S*)-BINAP and a bulky aliphatic analogue of **L121a** in which the arene-amino function had been replaced by a diisopropylamino group (ligand **L121b**). Control experiments showed that the enantioselectivity was mainly controlled by the diamine catalyst. Interestingly, catalysts containing ligands **L121a** and **L121b** having the same absolute configuration induced opposite enantioselectivities.

**Scheme 113:**
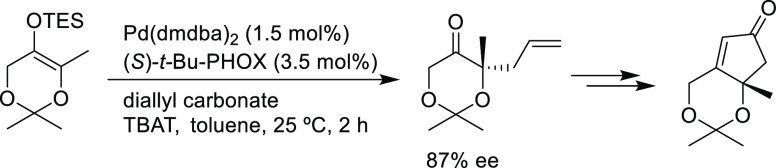
Synthesis of (*S*)-4-Allyl-2,2,4-trimethyl-1,3-dioxan-5-one by Asymmetric Pd-Catalyzed Allylic Alkylation Using a Silylenol Ether as Pronucleophile

Silylenol ethers have also been used as pronucleophiles, which allows regioselective enolate formation. In this way, Stoltz’s group was able to form quaternary stereogenic centers from 2-methyl cyclohexanone and a dioxanone analogue.^387^ From the latter, precursors of medicinally relevant hydroxymethyl-*cis*-1,3-cyclopentenediol building blocks were synthesized (Scheme 113). Paquin’s group used this strategy to synthesize α-allylated α-fluoroketones (for analogous decarboxylative transformations see section 3.1).,^388^^389^

**Scheme 114:**
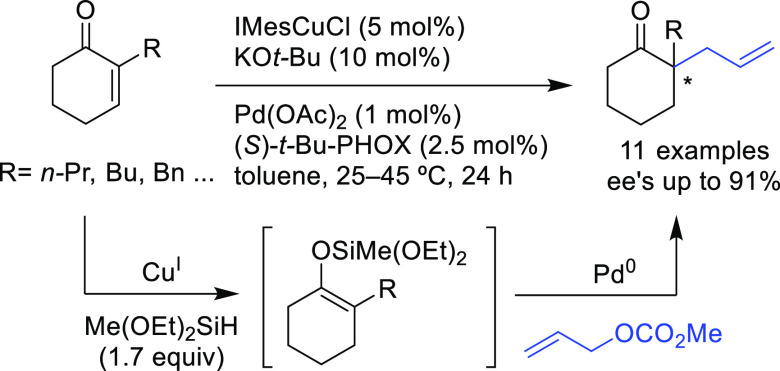
Cu^I^/Pd^0^ Dual Catalysis for the Synthesis of Quaternary α-Allylated Carbonyl Compounds from α-Substituted Cyclohexenones

Riant’s group developed a dual Cu^I^/Pd^0^ catalyst system for the conversion of 2-substituted cyclohexen-2-ones to α-allylated cyclohexanones (*ee* values up to 91%; Scheme 114).^390^ The reaction proceeds via Cu-catalyzed hydrosilylation leading to a silyl enol ether intermediate that then undergoes enantioselective Pd-catalyzed allylic substitution.

**Scheme 115:**
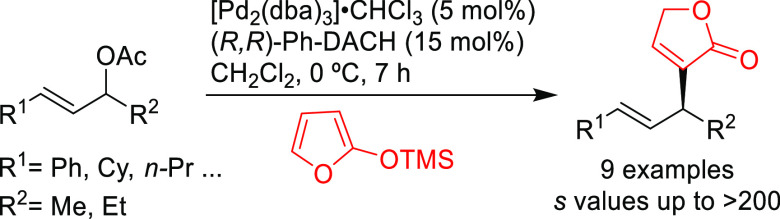
Asymmetric Synthesis of 3-Substituted γ-Butenolides via KR of Unsymmetrical Disubstituted Allyl Acetates

Feringa’s group studied the Pd-catalyzed kinetic resolution (KR) of unsymmetrical 1,3-disubstituted allyl acetates using (furan-2-yloxy)trimethylsilane as the nucleophile. In this way a range of 3-substituted γ-butenolides were synthesized in high enantiomeric purity (*s* factors up to >200; Scheme 115).^391^ Notably, under the same conditions the KR of cyclohexenyl acetate also proceed with high selectivity (*s* = 116).

**Scheme 116:**
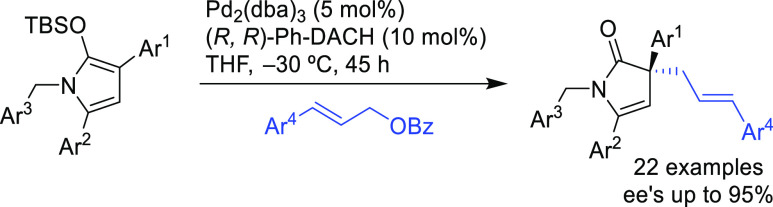
Pd-Catalyzed AAA of Cinnamyl-Type Benzoates with α,γ-Disubstituted 2-Silyloxypyrroles

More recently, Cossy and co-workers developed a highly regio- and enantioselective allylic alkylation of α,γ-disubstituted 2-silyloxypyrroles with a range of cinnamyl-type benzoates to yield chiral γ-lactams bearing an α-quaternary stereogenic center (regioselectivities up to >20/1 and *ee* values up to 95%; Scheme 116).^392^

**Scheme 117:**
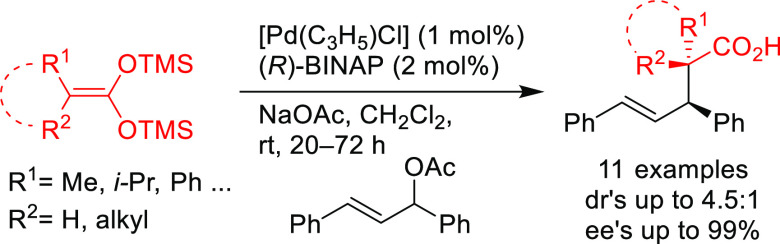
Diastereo- and Enantioselective Allylic Alkylation of *rac*-1,3-Diphenylallyl Acetate Using Bis(Trimethylsilyl)ketene Acetals as Nucleophiles

Bis(trimethylsilyl)ketene acetals were also used as nucleophiles in the Pd-catalyzed allylic alkylation of *rac*-1,3-diphenylallyl acetate (Scheme 117).^393^ The reactions proceeded with high enantioselectivities (*ee* values up to 99%), whereas the diastereoselectivities were only moderate (dr’s up to 4.5/1).

**Scheme 118:**
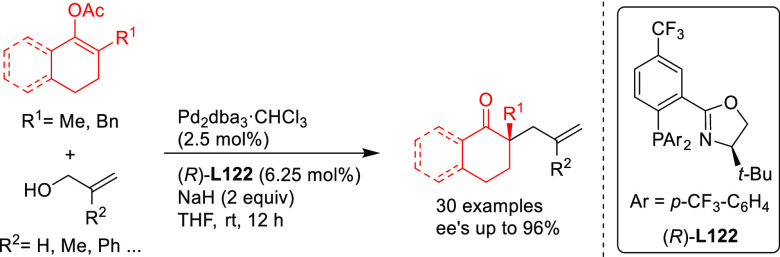
Enantioselective Allylic Alkylation of Enol Acetates Using Pd/(*R*)-**L122** Catalytic System

In 2018, Aponick’s group demonstrated that enol acetates can also be successfully employed as pronucleophiles. A range of 2-substituted cyclic enol acetates were efficiently allylated with several allylic alkoxides (*ee* values up to 96%; Scheme 118).^394^ The authors found that enantioselectivities were maximized by using (*R*)-**L122** ligand, an electron deficient analogue of *t*-Bu-PHOX ligand widely used in Pd-catalyzed decarboxylative asymmetric allylic alkylation reactions (see section 3).

**Scheme 119:**
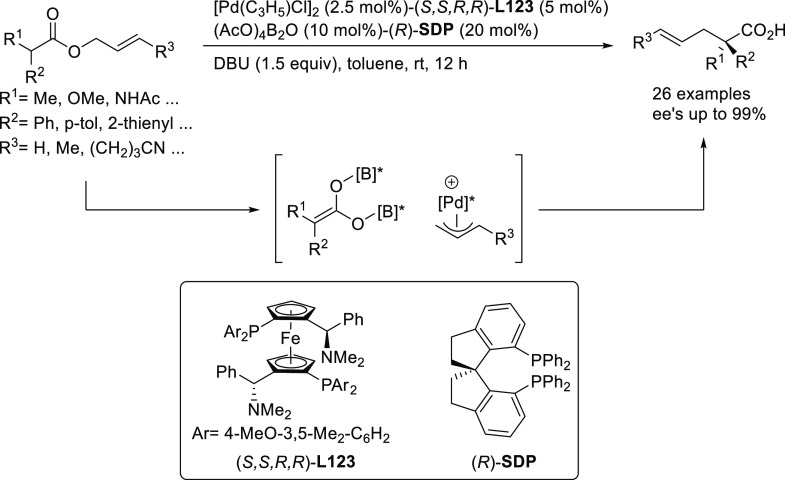
Synthesis of α-Allyl Carboxylic Acids Using a Pd/B Hybrid Catalyst

More recently, boron enolates were also used as pronucleophiles. Simuzu, Kanai and co-workers developed an efficient synthesis of α-allyl carboxylic acids using a Pd/B hybrid catalyst (*ee* values up to 99; Scheme 119).^395^ The reaction proceeds through a Pd-catalyzed ionization of α,α-disubstituted allyl esters to yield a chiral Pd η^3^-allyl complex, which is then attacked by the in situ formed α,α-disubstituted carboxylic acid-derived boron enolate.

**Scheme 120:**
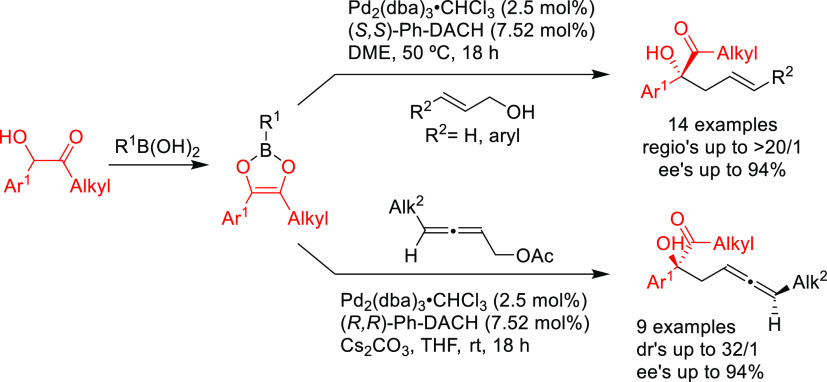
Pd-Catalyzed Allylic Alkylation of Allylic Alcohols and Allenyl Acetates Using 1,3-Dioxaboroles

Trost’s group demonstrated that 1,3-dioxaboroles, prepared by condensation of boronic acids with α-hydroxyketones, can be used as substitutes for ene-diolates.^396^ The role of the boron enolate is to block O-alkylation and control the enolate geometry. By this strategy allylic alcohols were alkylated with high regio- and enantioselectivities (Scheme 120). Moreover, the range of substrates was successfully expanded to *rac*-allenyl acetates, which reacted via a dynamic kinetic asymmetric transformation (DYKAT) with high enantio- and diastereoselectivities (Scheme 120). Subsequently, the same group also reported the allylic alkylation of allyl acetate with enol boranes, prepared in situ from α,β-unsaturated carbonyl compounds via 1,4-hydroboration, albeit with low *ee* values (up to 35%).^397^

**Scheme 121:**
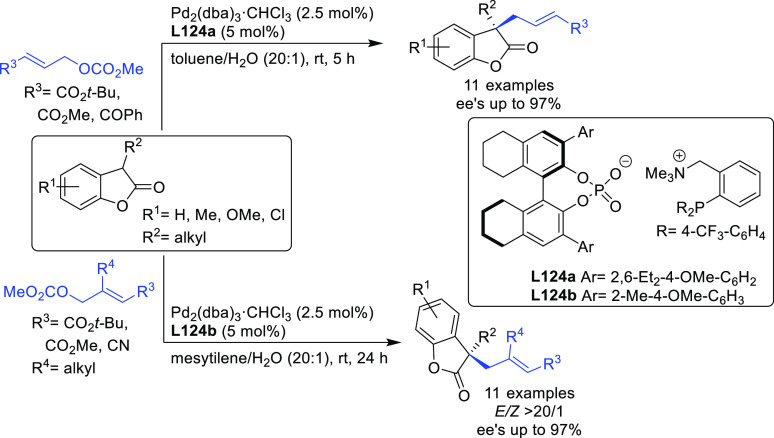
Asymmetric Pd-Catalyzed Allylic Alkylation of Benzofuranones

Enolates from esters and lactones have also been studied. An example was reported by Ooi and co-workers who developed a highly enantioselective allylation of benzofuranones with a range of linear allylic substrates using ion-paired chiral ligands consisting of a phosphinoaryl ammonium salt and a chiral biaryl phosphate (Scheme 121).^90,91^ For 1,2-disubstituted substrates, it was possible to control the *E/Z* selectivity by introducing a substituent in the 2-position, which favors the formation of the *anti* complex, since the *syn* complex is destabilized by a 1,2-steric repulsion (Scheme 121).^90^ This strategy was also successfully applied to benzothiofuranones.

**Scheme 122:**
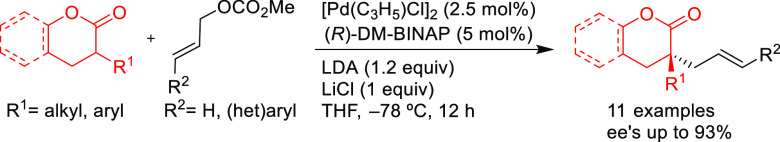
Asymmetric Pd-Catalyzed Allylation of Lactones Using Pd/(*R*)-DM-BINAP as Catalyst

The group of Hou studied the allylation of six-membered ring lactones with a range of linear substrates using BINAP-type ligands (Scheme 122, *ee* values up to 93%).^398^ This protocol also worked well for the kinetic resolution of 4-substituted-3,4-dihydrocoumarins with *s* values up to 55.^399^

**Scheme 123:**
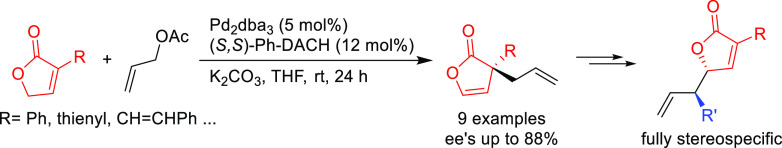
Asymmetric Pd-Catalyzed α-Allylation of 3-(Hetero)aryl- and 3-Allyl-2(5*H*)-furanones Using Pd/(*S*,*S*)-Ph-DACH as Catalyst

More recently, the successful α-allylation of 3-substituted-2(5*H*)-furanones^400^ and 3-ethyltetrahydro-2*H*-pyran-2-one^401^ was described. Arseniyadis’ group demonstrated that a Pd/(*S*,*S*)-Ph-DACH complex efficiently catalyzed the allylation of 3-(hetero)aryl- and 3-allyl-2(5H)-furanones with allyl acetate (*ee* values up to 88%; Scheme 123).^400^ The resulting α,α-disubstituted furanones were transformed to γ-butenolides bearing two adjacent stereogenic centers by sequential cross-metathesis/Cope sigmatropic rearrangement (Scheme 123). The group of Wang used a Pd/(*R*)-DM-BINAP catalyst to allylate 3-ethyltetrahydro-2*H*-pyran-2-one with allyl methyl carbonate.^401^ The resulting (*R*)-3-allyl-3-ethyltetrahydro-2*H*-pyran-2-one served as a building block for the synthesis of (−)-scholarisine G, (+)-melodinine E, (−)-leuconoxine, and (−)-mersicarpine (see section 2.5).

**Scheme 124:**
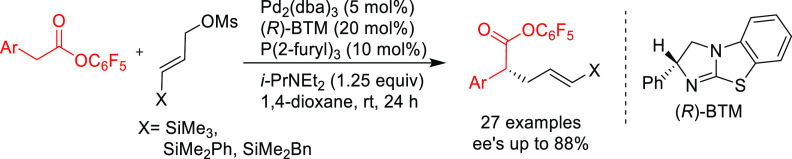
Asymmetric Pd-Catalyzed Allylation of Pentafluorophenyl Esters as Pronucleophiles

A different approach for the allylation of esters was developed by the Snaddon group using pentafluorophenyl esters as pronucleophiles, which were converted in situ to chiral ammonium enolates with the chiral Lewis base (*R*)-BTM (Scheme 124).^402^ The regioselectivity of nucleophilic attack was controlled by the silicon substituent in the allyl system.

**Scheme 125:**
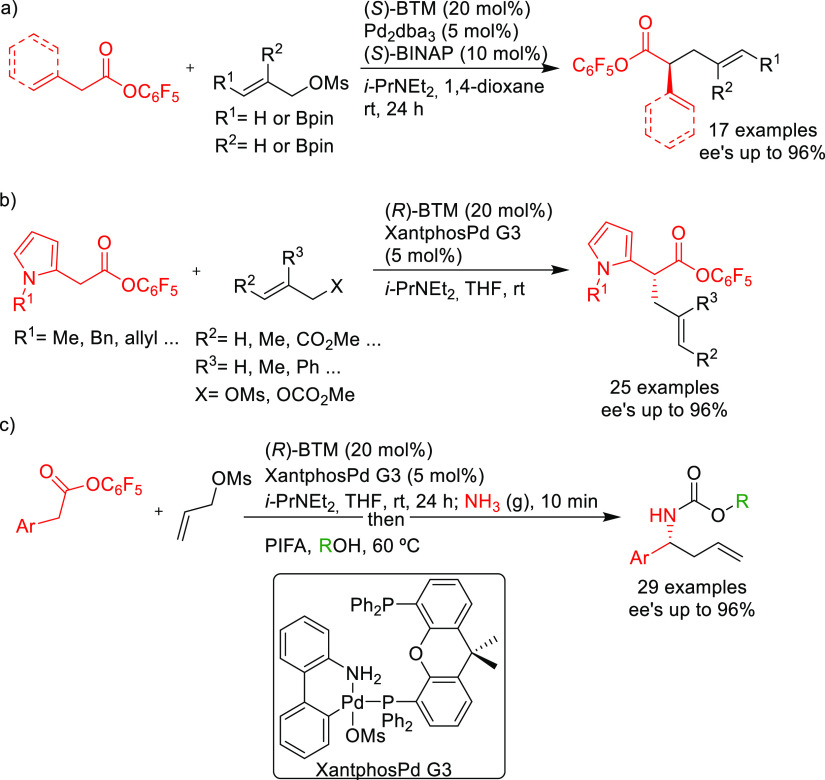
Synthesis of (a) α-Allylated Acetic Esters Containing a Vinylboronic Group, (b) α-Allylated Pyrrolyl-Acetic Esters, and (c) Carbamate-Protected Homoallylic Amines PIFA = bis(trifluoroacetoxy)iodo)benzene.

More recently, the same group expanded the range of pentafluorophenyl esters that could be successfully allylated, using a cooperative dual catalysts system consisting of the chiral bicyclic isothiourea derivative BTM and a chiral Pd-diphosphine complex.^403−405^ Various aryl- and vinyl acetic acid esters were α-allylated with Bpin-substituted allyl mesylates (*ee* values up to 96%; Scheme 125a).^403^ Pyrrolyl-acetic esters were also α-allylated with a range of allyl sulfonates or carbonates with high levels of enantioselectivity (Scheme 125b).^404^ This allylation method was also combined with a subsequent Hofmann rearrangement in a one-pot procedure. In this way, carbamate-protected branched homoallylic amines were prepared with high enantioselectivity (Scheme 125c).^405^

**Scheme 126:**
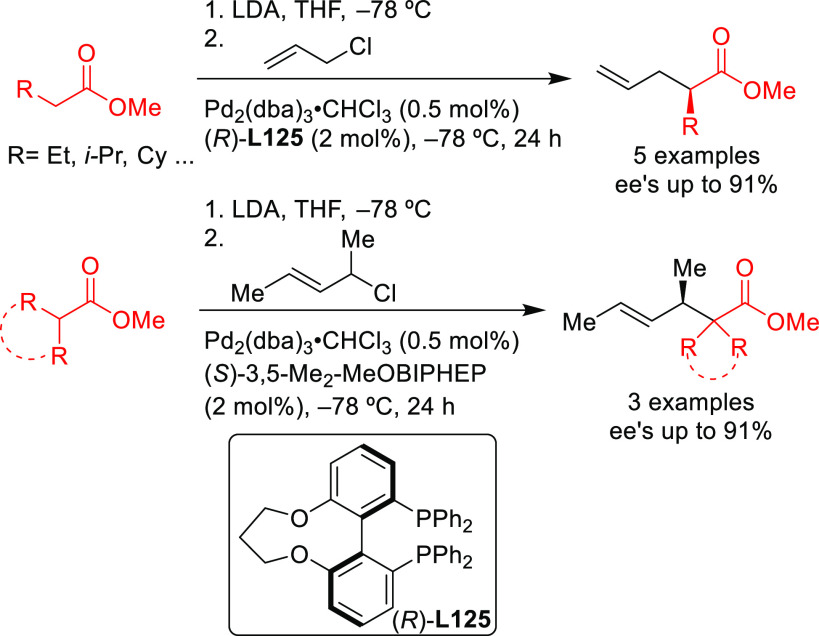
Asymmetric Allylic Alkylation of Alkanoic Acid Esters

Braun and co-workers disclosed a protocol for the Pd-catalyzed AAA of simple alkanoic acid esters via lithium enolates (*ee* values up to 91%; Scheme 126).^406^ The potential of this protocol has been demonstrated by subsequent transformation of the resulting chiral α-substituted allylic esters to succinates and lactones in few steps.

**Scheme 127:**
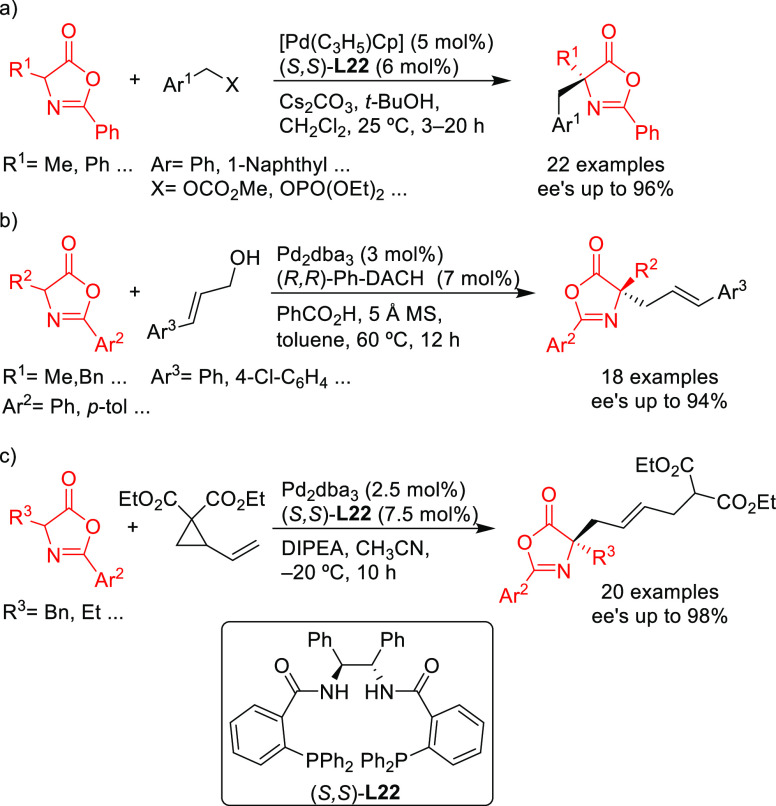
Asymmetric Pd-Catalyzed Allylic and Benzylic Alkylation of Azlactones as Pronucleophiles

Azlactones have also been used as pronucleophiles. The Trost group reported the asymmetric alkylation of a range of azlactones with several benzylic electrophiles (*ee* values up to 96%; Scheme 127a).^407,408^ The resulting α,α-disubstituted azlactone can be easily converted to α, α-disubstituted amino acids, as demonstrated by the synthesis of α-methyl-D-Dopa. Jiang and co-workers used Pd/(*R,R*)-Ph-DACH as catalyst to α-allylate azlactones with simple cinnamyl-type alcohols (*ee* values up 94; Scheme 127b)^409^ while Cai’s group successfully used activated vinylcyclopropanes as substrates (*ee* values up to 98%; Scheme 127c).^410^

**Scheme 128:**
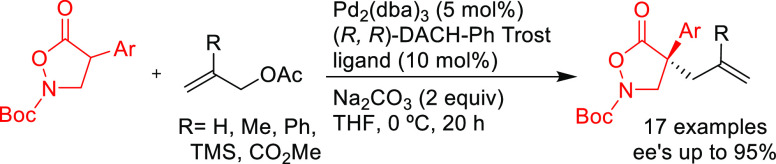
Asymmetric Pd-Catalyzed Allylation of 4-Substituted Isoxazolidin-5-ones

The Cossy group developed a highly enantioselective route to β-amino acids with a quaternary center at the 2-position through allylation of 4-substituted isoxazolidin-5-ones and subsequent reductive cleavage of the N–O bond (Scheme 128).^411^

**Scheme 129:**
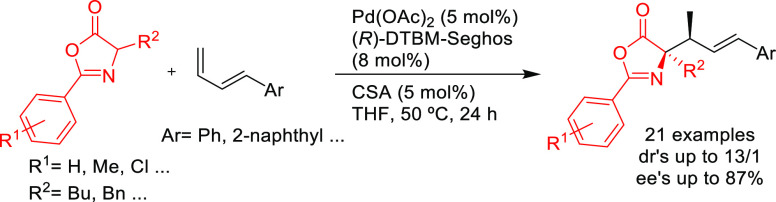
Asymmetric Pd-Catalyzed Allylic Alkylation of Azlactones with 1,3-Dienes

Xing’s group have recently reported the highly diastereo- and enantioselective allylic alkylation of azlactones with 1,3-dienes using Pd/(*R*)-DTBM-Segphos as catalyst (dr’s up to 13/1, *ee* values up to 87%; Scheme 129).^412^

**Scheme 130:**
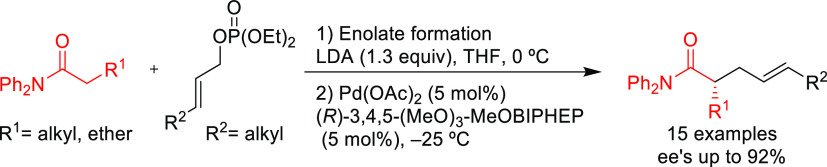
Asymmetric Pd-Catalyzed Allylation of Acyclic Amides

Enolates from amides and lactams have also been used as nucleophiles. An example is the highly enantioselective allylation of a range of alkyl substituted allylic substrates with acyclic amides (Scheme 130).^413^ The potential of this protocol has been demonstrated by the synthesis of a precursor of dubiusamine A (see section 2.5). Thioamides as well were allylated in a similar way.^414^ Similarly, enolates of 3-aryl-2-piperidinones have been used as nucleophiles.^415^

**Scheme 131:**
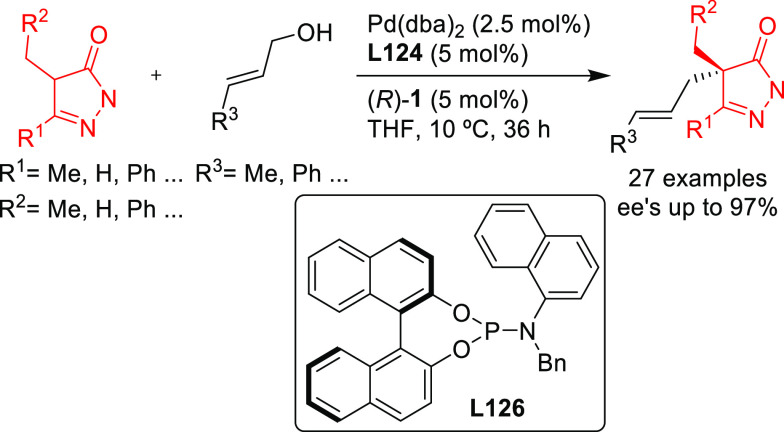
Asymmetric Pd-Catalyzed Allylic Alkylation of Pyrazol-5-ones

The highly enantioselective allylic alkylation of pyrazol-5-ones with allylic alcohols has been achieved by counteranion-directed catalysis using a combination of phosphoramidite **L126** as ligand and (*R*)-1,1′-binaphthyl-2,2′-diyl hydrogen phosphate ((*R*)-**1**; Scheme 10) as chiral Brønsted acid (*ee* values up to 97%; Scheme 131).^416^

**Scheme 132:**
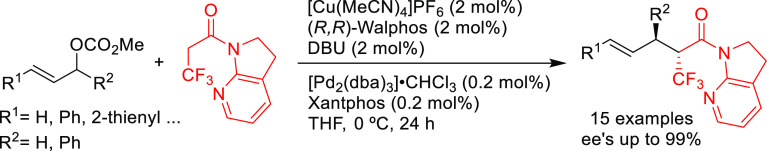
Synergistic Cu/Pd Dual Catalytic Allylation of an α-CF_3_-Acetamide

In the past few years, a new strategy based on a synergistic Cu/Pd dual catalyst system has been developed for the highly enantioselective allylic alkylation of various enolizable compounds. The chiral Cu(I) catalyst is necessary to stabilize the enolate by complexation and to direct nucleophilic attack to one of the two enantiofaces of the enolate. One of the first examples was reported by Kumagai’s and Shibasaki’s group using this strategy to allylate α-CF_3_-acetamide derived from 7-azaindoline with a range of allyl carbonates (Scheme 132).^417^ The resulting α-CF_3_-γ,δ-unsaturated amides were obtained with excellent enantioselectivities.

**Scheme 133:**
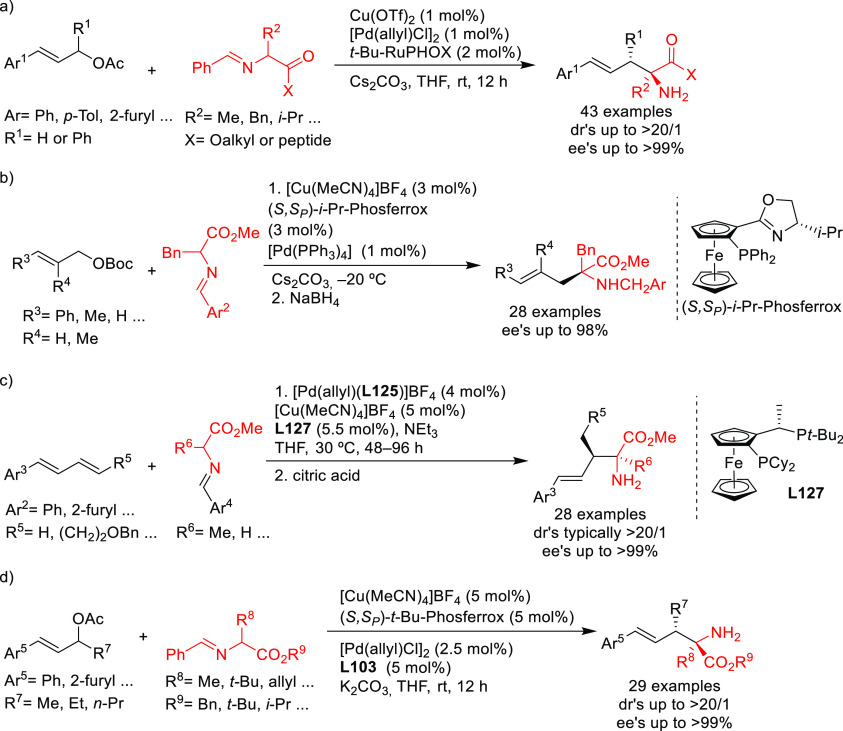
Enantioselective Allylation of Aldimine-Protected α-Amino Acid Esters Using a Dual Cu/Pd Catalyst System

The same approach has also been employed for the allylic alkylation of aldimine-protected α-amino acid esters with allylic carbonates.^55,418−420^ In this way a range of mono- and disubstituted allylic acetates were enantioselectively alkylated with Schiff base activated amino acids and small peptides using *t*-Bu-RuPHOX ligand (Scheme 133a).^418^ Subsequently, a range of α,α-disubstituted α-amino acids were prepared with high enantioselectivities using the Pd/(*S*,*S*_P_)-*i*-Pr-Phosferrox catalytic system (Scheme 133b).^419^ Electron-rich dienes were also used as allylating agents giving rise to α,α-disubstituted α-amino acids bearing two vicinal stereogenic centers with high diastereo- and enantioselectivities (dr’s typically >20/1 and *ee* values up to >99%; Scheme 133c).^420^ The same catalyst system was also applied in the dynamic kinetic resolution of racemic unsymmetrical 1,3-disubstituted allylic acetates using the same type of aldimine-protected α-amino acid derivatives (yields up to 88% and *ee* values up to >99%; Scheme 133d). Noteworthy, all four stereoisomers of the product were accessible by switching the configurations of the two ligands.^55^

**Scheme 134:**
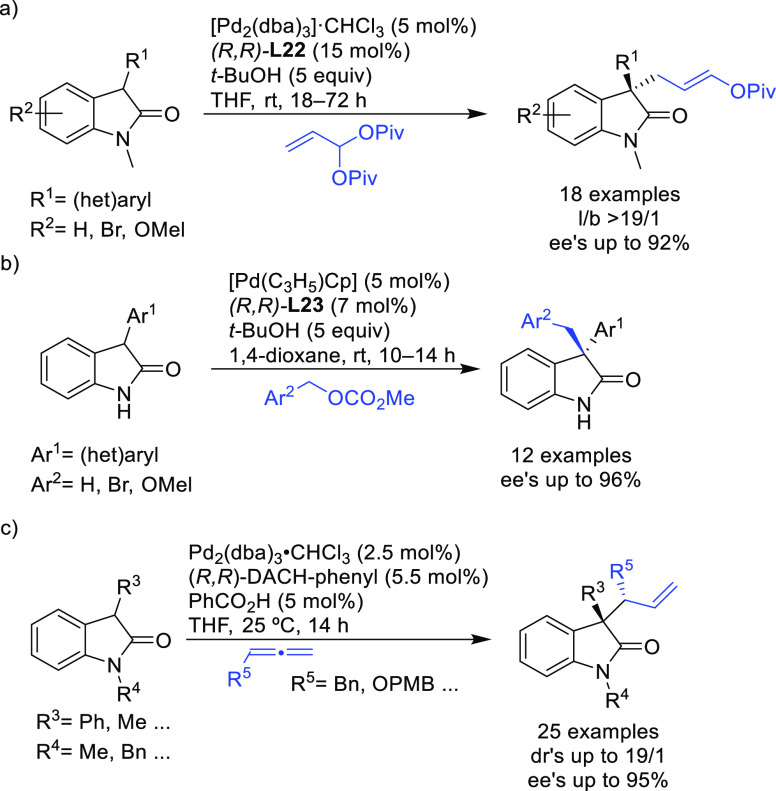
Asymmetric Pd-Catalyzed Allylation and Benzylation of 3-Aryloxindoles

**Scheme 135:**
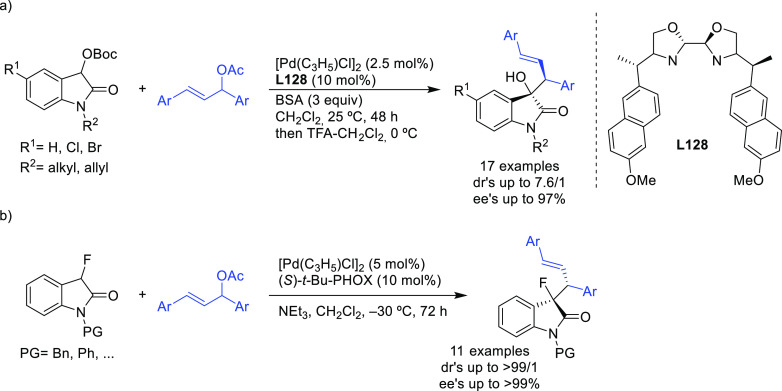
Asymmetric Pd-Catalyzed Allylation of Boc-Protected 3-Hydroxy Oxindoles

Enantioselective allylation of 3-substituted oxindoles has been reported by several groups. Trost and co-workers studied the allylation of 3-aryloxindoles with allylidene dipivalates (Scheme 134a)^421^ and *tert*-butyl (2-methylbut-3-en-2-yl) carbonate^422^ to give oxindoles with a quaternary stereogenic center. Similarly, benzylation of 3-aryl oxindoles was shown to proceed in high yields and enantioselectivities (*ee* values up to 96%; Scheme 134b) using the chiral Trost ligand (*R,R*)-**L23** (Scheme 26).^423^ A range of 3-substituted oxindoles were therefore efficiently allylated using several allenes with Pd/(*R, R*)-DACH-phenyl catalytic system in the presence of benzoic acid. The reaction proceeded smoothly at room temperature providing the alkylated products with two vicinal stereocenters in high selectivities (Scheme 134c).^424^ The allylation of Boc-protected 3-hydroxyindoles was studied by the group of Kesavan using symmetrically disubstituted 1,3-diarylallyl substrates. A range of 3-allyl-3-hydroxyoxindoles was obtained in high enantioselectivity and moderate diastereoselectivity (Scheme 135a).^425^ More recently, Wolf’s group also disclosed the allylation of 3-fluorinated oxindoles with 1,3-diarylallyl acetates in excellent diastereo- and enantioselectivities using the Pd/(*S*)-*t-*Bu-PHOX catalytic system (Scheme 135b).^426^

**Scheme 136:**
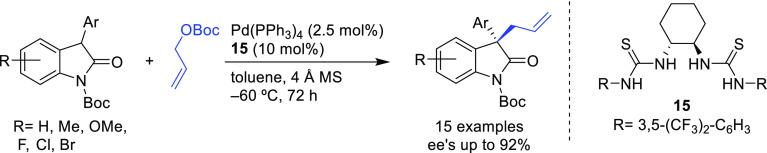
Asymmetric Allylation of 3-Aryloxindoles via Cooperative Pd Catalysis and Asymmetric Hydrogen Bonding Catalysis

Similarly, Xiao’s group allylated 3-aryloxindoles, although in this case, an achiral Pd-complex served as catalyst, while enantiofacial discrimination was achieved through hydrogen bonding to a chiral bisthiourea derivative **15** as additive (Scheme 136).^427^

**Scheme 137:**
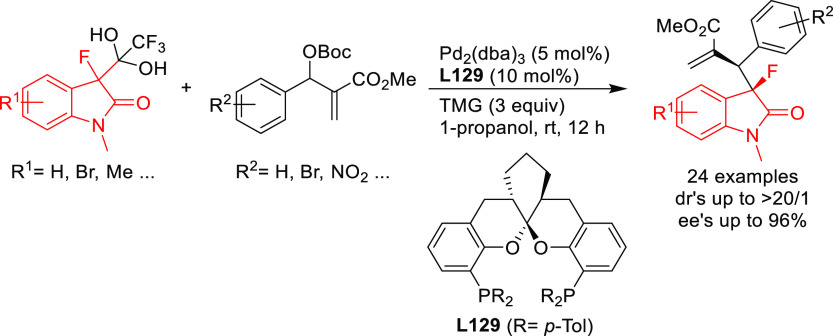
Asymmetric Allylation of Colby-Type Proenolates with Carbonates Derived from Morita–Baylis–Hillman Products TMG = 1,1,3,3-tetramethylguanidine.

Hayashi’s group recently developed a highly efficient procedure for the enantioselective allylation of 3-fluoro-oxindoles using carbonates derived from Morita–Baylis–Hillman products and Colby-type proenolates, which are converted in situ to the corresponding enolates by deprotonation with 1,1,3,3-tetramethylguanidine (TMG).^428^ In this way, oxindoles with two adjacent stereogenic centers, including a tetrasubstituted fluorinated carbon atom, were prepared with high diastereo- and enantioselectivities (dr’s up to >20/1 and *ee* values up to 96%; Scheme 137).

**Scheme 138:**
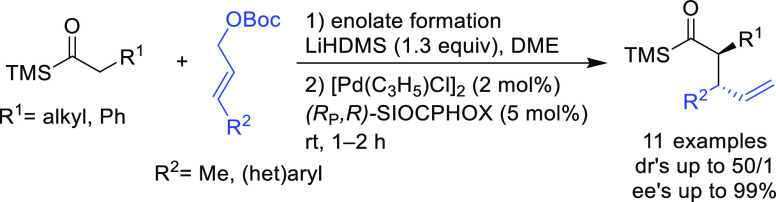
Asymmetric Allylation Using Acylsilane Enolates as Nucleophiles

Acylsilane enolates were also found to be suitable nucleophiles, reacting with high regio-, diastereo-, and enantioselectivities in the allylation of a range monosubstituted allylic substrates (Scheme 138).^429^ The regioselectivity favoring the branched product was controlled by the chiral ferrocene P,N-ligand (*R*_p_*,R*)-SIOCPHOX.

**Scheme 139:**
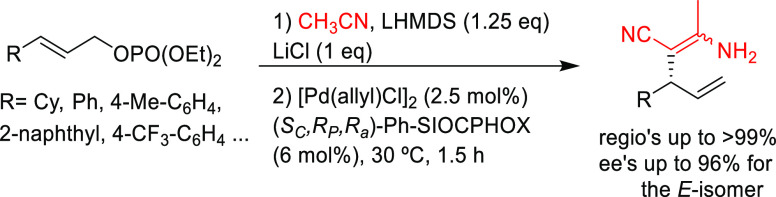
Pd-Catalyzed Allylic Alkylation of a Range of Cinnamyl Methyl Carbonates with Nitroalkanes with Pd/(*S*_C_,*R*_p_,*R*_a_)-Ph-SiOCPhox as Catalyst

Nitriles can also be used as pronucleophiles as demonstrated by Hou and co-workers. They disclosed a new way to access chiral β-enaminonitriles via Pd-catalyzed allylic alkylation of monosubstituted allylic phosphonates with a 3-imino nitrile carbanion generated in situ by a Thorpe reaction from acetonitrile (Scheme 139).^430^ The resulting β-enaminonitriles were obtained with excellent regio- (up to >99%) and enantioselectivities (*ee* values up to 96% and 98% for the *E*- and *Z*-isomers, respectively), and *E/Z* ratios of typically 9:1, favoring the more stable *E*-isomer.

**Scheme 140:**
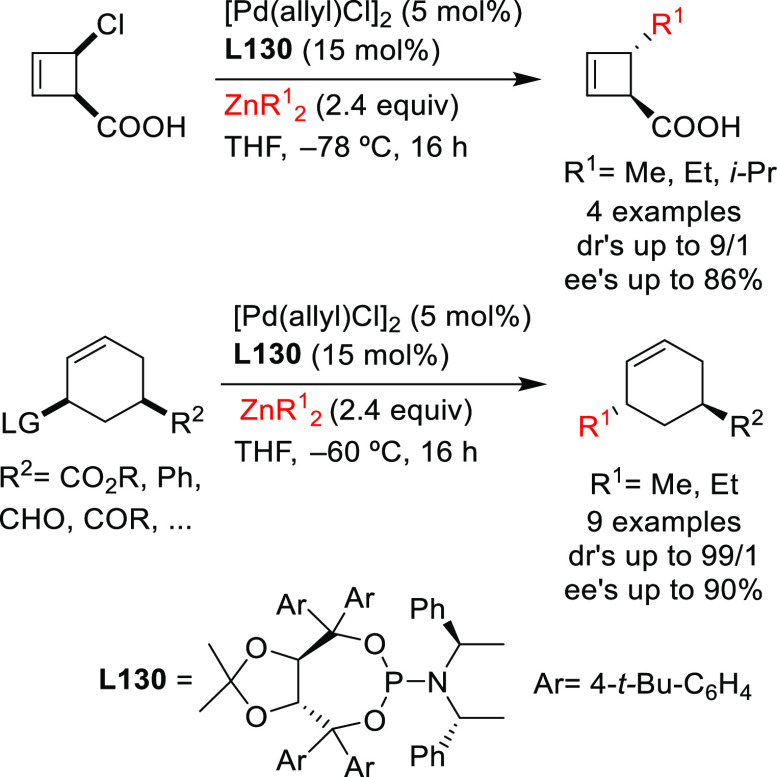
Catalytic Asymmetric Alkylation of Cyclic Substrates Using Dialkylzinc Reagents

**Scheme 141:**
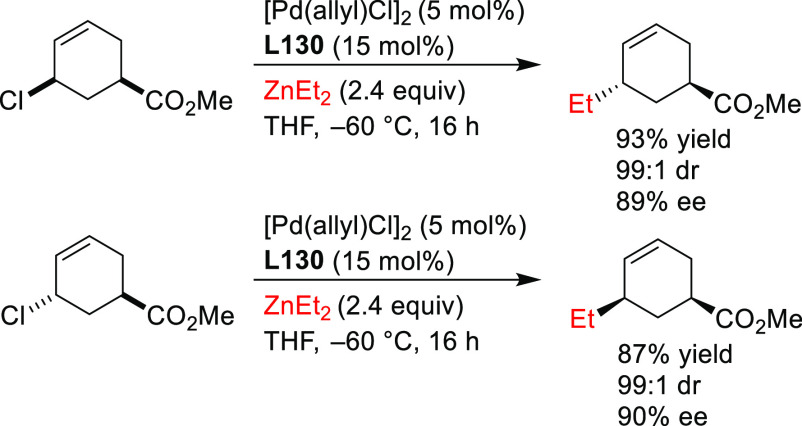
Pd-Catalyzed Allylic Substitution of Cyclic Substrates Using Diethylzinc

Although most Pd-catalyzed allylic substitutions reported in the literature focused on stabilized carbon nucleophiles such as malonates, there have been recent examples of allylations with nonstabilized carbon nucleophiles like organozinc reagents. In this context, Maulide’s group has found that by the appropriate selection of ligand, it is possible to overcome the usually observed “Umpolung” reactivity (nucleophilic nature) of the Pd-allyl species in the presence of dialkyl zinc.^431^ The use of TADDOL-based phosphoramidites, such as **L130**, allowed for the highly diastereo- and enantioselective allylic alkylation of cyclic substrates (Scheme 140). As expected for allylations with nonstabilized carbon nucleophiles the reactions proceed by overall inversion of configuration (Scheme 141).

Very recently, Fañanás-Mastral’s group has demonstrated that Cu-alkenylboranes, generated in situ from alkynes, can also be used as nonstabilized carbon nucleophiles in allylic substitutions with cyclic allylic carbonates, although the enantioselectivities were only moderate (up to 54% *ee*).^432^

**Scheme 142:**
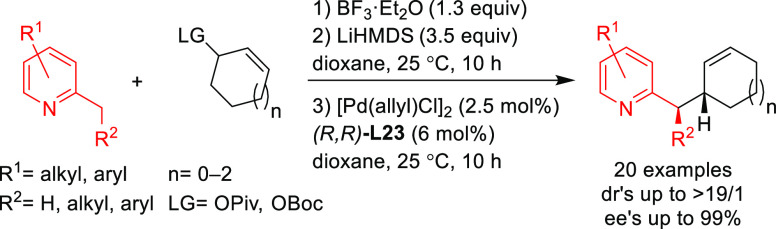
Pd-Catalyzed Asymmetric Allylic Alkylation of Methyl and 2-Substituted Alkylpyridyl Nucleophiles

Metalated 2-methyl-substituted^433^ and related 2-substituted^434^ pyridines were also successfully used as nucleophiles in this transformation by Trost and co-workers (Scheme 142). It was found that precomplexation of the pyridine unit to BF_3_·Et_2_O was necessary.

**Scheme 143:**
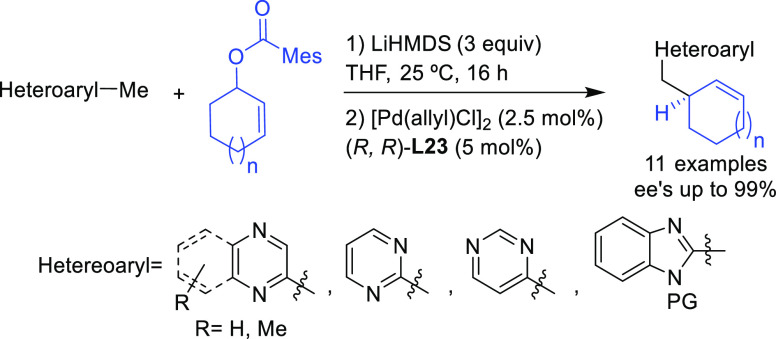
Selected Examples for the Asymmetric Pd-Catalyzed Allylic Alkylation Using Polynitrogen-Containing Heterocyclic Nucleophiles

Subsequently, the reaction was extended to other N-heterocycles such as pyrazine, pyrimidine, pyridazine, quinoxaline, and benzoimidazole derivatives (Scheme 143).^435^ In this case no precomplexation of the heterocycle with a Lewis acid was required, which rendered the reaction more atom economic. To prevent deacetylation of the allylic substrate, bulky mesityl esters were used.

**Scheme 144:**
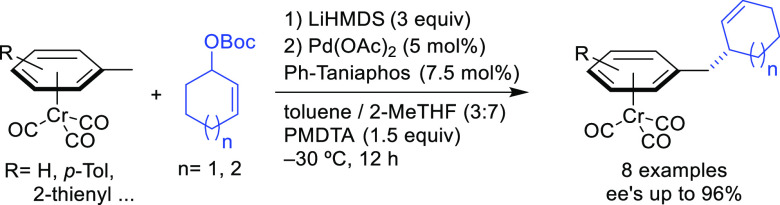
Asymmetric Pd-Catalyzed AAA Using Toluene Derivatives as Pronucleophiles

By analogy to the allylation of 2-methylpyridines, the group of Walsh used toluene derivatives as pro-nucleophiles, which were activated by complexation with chromium tricarbonyl (Scheme 144).^436^

**Scheme 145:**
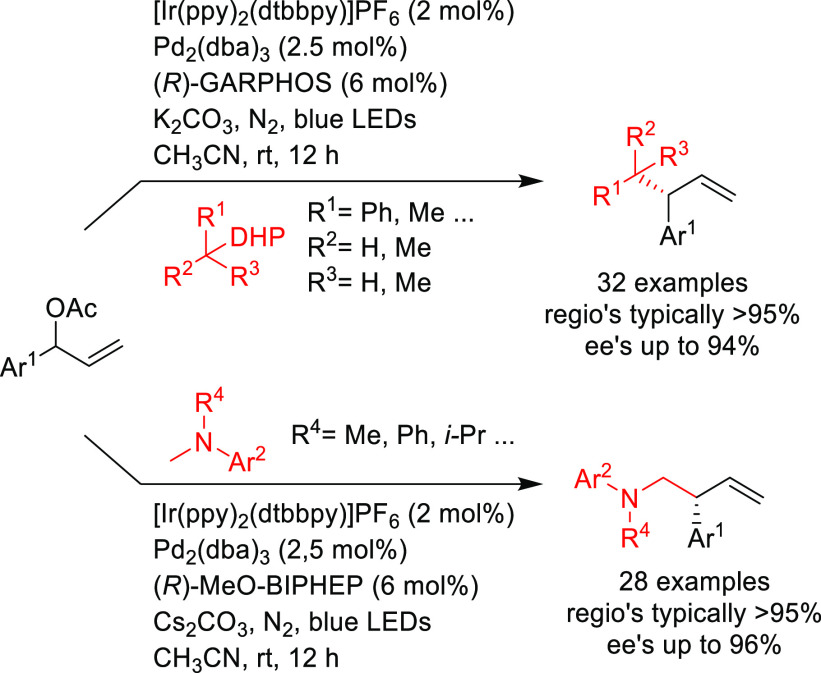
Dual Photoredox/Pd-Catalyzed Reaction of 4-Alkyl-1,4-dihydropyridines and Anilines with Allyl Acetates

Recently, You’s group developed a dual catalytic process involving photoredox and enantioselective palladium catalysis.^437,438^ In this process alkyl radical species are generated from 4-alkyl-1,4-dihydropyridines^437^ and anilines,^438^ which react as nonstabilized carbon nucleophiles with a variety of allylic esters. The reaction proceeded with high regioselectivities favoring the branched products (regioselectivities typically >19/1) and enantioselectivities (*ee* values up to 96%; Scheme 145).

**Scheme 146:**
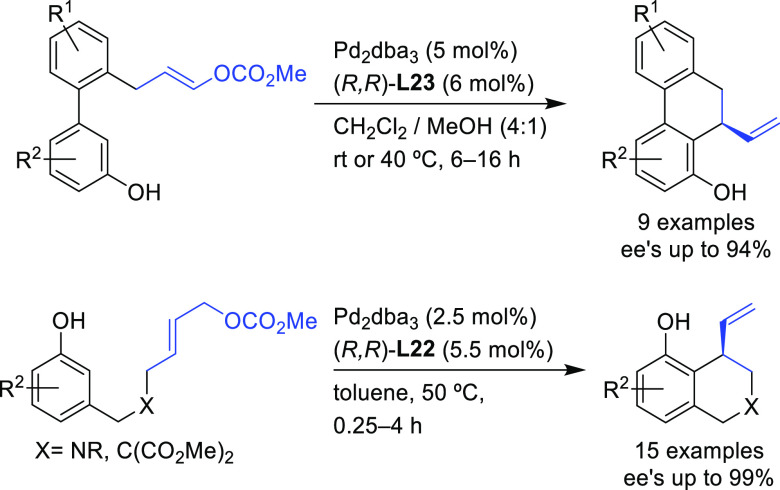
Representative Friedel–Crafts-Type Allylation Reactions of Phenols

Exploiting the electrophilic nature of Pd allyl complexes, Friedel–Crafts like allylation reactions of phenols were developed that led to a range of 9,10-dihydrophenanthrenes^439^ and at C4-substituted tetrahydroisoquinolines^440^ (Scheme 146). This approach was used for the preparation of cedralin A and methylated paralycolin B (see section 2.5).^441^ Similarly, an *ipso*-Friedel–Crafts-type allylic alkylation of phenols to yield spiro[4,5]cyclohexadienones has been reported.^442^

**Scheme 147:**
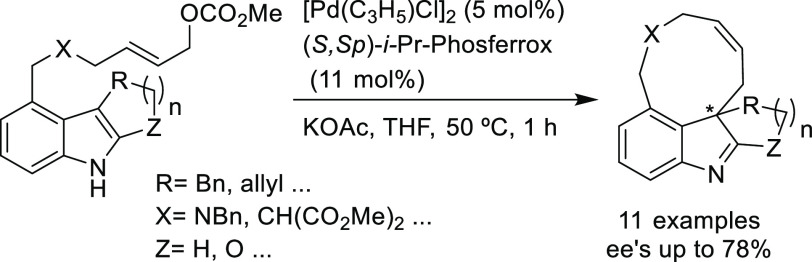
Pd-Catalyzed Friedel–Crafts-Type Allylic Alkylation Reaction of Indole Fused through C4–C3

The Friedel–Crafts-type intramolecular allylic alkylation was also expanded to indoles. An example is the Pd-catalyzed dearomatization of 3-substituted indoles studied by You’s group (Scheme 147).^443^ The resulting indole-based peri-annulated compounds, fused through C4–C3, were obtained in good yields and low-to-moderate *ee* values (up to 78%) using Pd/(*S*,*S*_p_)-*i*-Pr-Phosferrox as catalyst. It should be mentioned that the analogous Ir-catalyzed reaction proceeds with higher enantioselectivities (up to 97% *ee*).

**Scheme 148:**
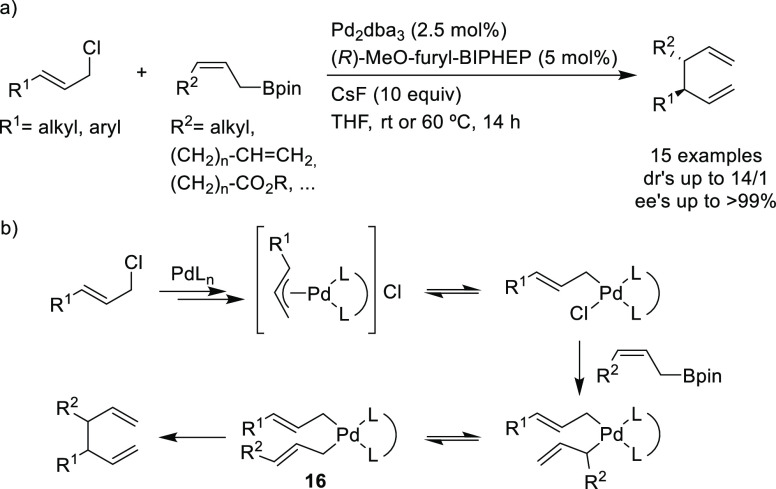
(a) Catalytic Diastereo- and Enantioselective Allylation of Monosubstituted Substrates Using a Range of Allylboronates and (b) Proposed Mechanism for This Transformation

In recent years, allylboron compounds have emerged as versatile building blocks, due to their ability to react with electrophiles.^32^ Accordingly, they have also been used as nucleophiles in Pd-catalyzed allylic substitution reactions. Morken’s group reported the reaction of several monosubstituted allylic chlorides using a range of allylboronates (Scheme 148a). This transformation, which may be considered as a hybrid between an allylic substitution and a cross-coupling reaction, yielded a range of chiral 3,4-disubstituted 1,5-dienes (Scheme 148b).^444,445^ The crucial intermediate in this transformation was proposed to be a Pd-diallyl complex **16**, which undergoes reductive elimination.^445^

**Scheme 149:**
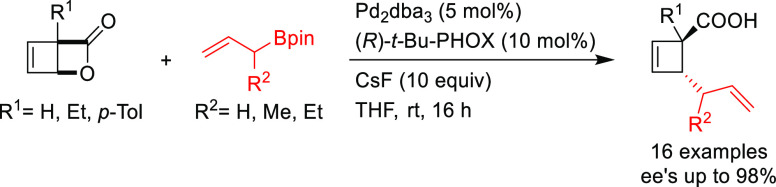
Catalytic Enantioselective Allylation of Strained Bicylic Lactones Using Several Allylboronates

Further examples of transformations of this type were reported by Maulide and co-workers starting from strained bicylic lactones to provide geminal or vicinal disubstituted cyclobut-3-enes (Scheme 149).^446^

**Scheme 150:**
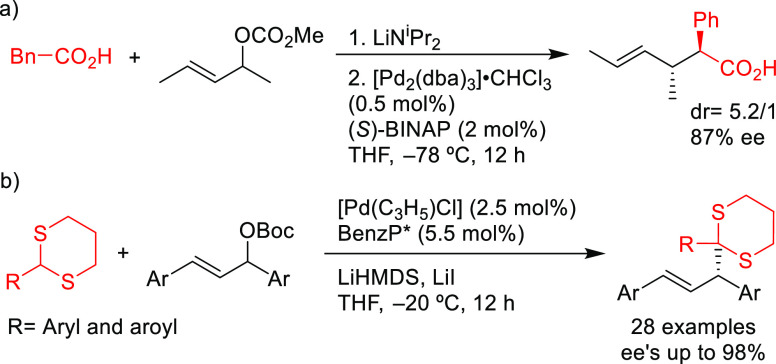
Asymmetric Pd-Catalyzed Allylic Alkylation of (a) Doubly Deprotonated Carboxylic Acids and (b) 1,3-Dithianes

Other examples of highly basic carbanions that have been used as nucleophiles are lithiated 1,3-dithianes and doubly deprotonated carboxylic acids.^447,448^ Braun and co-workers demonstrated that deprotonation of carboxylic acids with 2 equiv of LDA and subsequent Pd-catalyzed allylation with allyl carbonates led to α,β-disubstituted carboxylic acids with moderate diastereo- and enantioselectivities (dr’s up to 5.2/1 and *ee* values up to 87%; Scheme 150a).^447^ More recently, Zhang’s group developed a highly enantioselective Pd-catalyzed allylic alkylation of 1,3-dithianes as acyl anion equivalents with a range of 1,3-diarylpropenyl carbonates (*ee* values up to 98%; Scheme 150b).^448^

### 2.4 Key Mechanistic Aspects

**Scheme 151:**
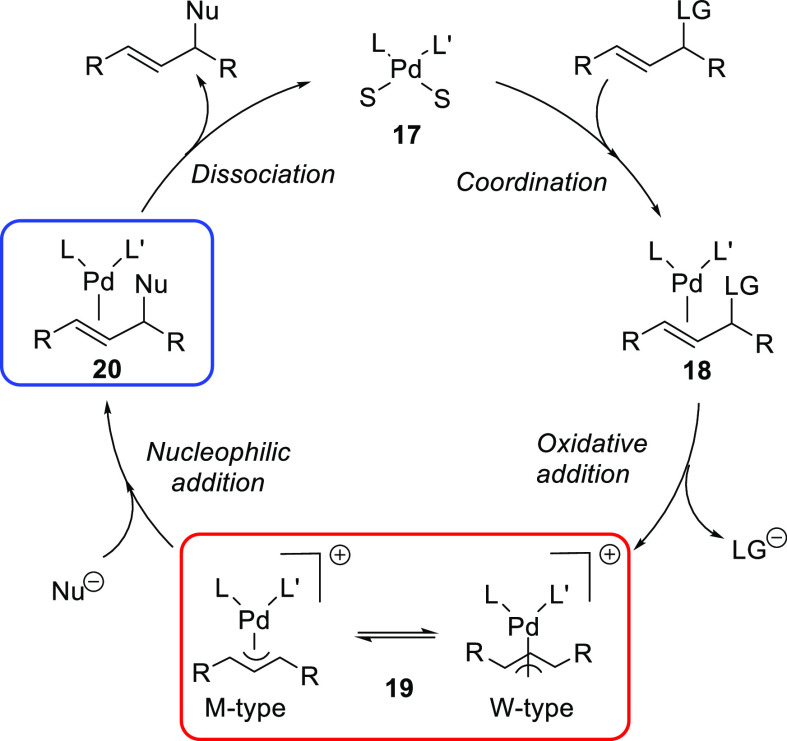
Mechanism for the Pd-Catalyzed Asymmetric Allylic Substitution L,L′ = mono- or bidentate ligand; S = solvent or vacant; LG = leaving group; Nu = nucleophile.

The universally accepted catalytic cycle for enantioselective Pd-catalyzed allylic substitution reactions with stabilized carbon nucleophiles and heteronucleophiles includes the four main steps shown in Scheme 151. The first step is the coordination of the allylic substrate to Pd **17***trans* to its leaving group, leading to olefin complex **18**. The next step is an oxidative addition with dissociation of the leaving group, leading to the two equilibrating key Pd η^3^-allyl intermediates **19** (identical for *C*_2_-symmetric ligands) and, usually minor, *syn,anti* and *anti,anti* isomers, followed by nucleophilic attack to give olefin complex **20**. The final step is the release of the substituted product olefin by dissociation and regeneration of the Pd catalyst. The enantiodetermining step can be either the oxidative addition or the nucleophilic attack. For substrates with enantiotopic leaving groups (in geminal or 1,3-positions), the enantiodiscriminating step is the oxidative addition, whereas for substrates with identical substituents at the 1- and 3-positions, enantiodiscrimination occurs during nucleophilic attack at one of the diastereotopic sites (enantiotopic in the presence of achiral ligands). For other substrates, the relative rates of the different steps, including interconversion of isomeric allyl complexes, govern in which step the enantioselectivity is determined. Use of prochiral nucleophiles, finally, may also lead to enantiodiscrimination.

**Scheme 152:**
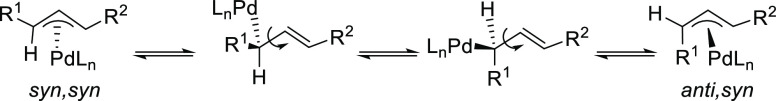
Interconversion of *syn,syn* and *syn,anti* η^3^-Allyl Complexes via η^1^-Complexes

The intermediate allylpalladium complexes undergo isomerizations under the conditions of the catalytic reactions. For noncyclic substrates, *syn,syn*, *syn,anti*, and *anti,anti* η^3^-allyl complexes equilibrate via η^1^-complexes, a process that changes the configuration at a terminal allyl carbon atom, but which does not change the relative positions of the allyl carbons and the ligands (Scheme 152).

**Scheme 153:**
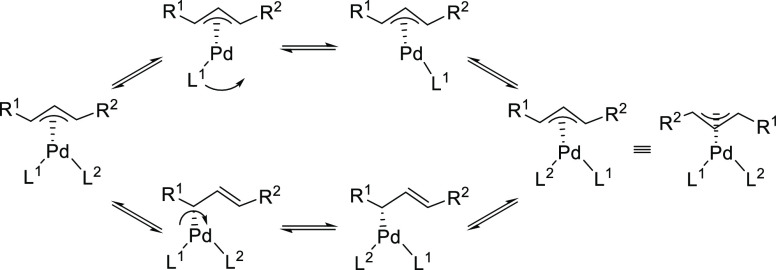
Apparent Allyl Rotation via a Dissociative Mechanism (Upper Pathway) or via Intermediate η^1^-Complexes and Rotation around the C–Pd Bond (Lower Pathway)

Another type of isomerization is apparent allyl rotation, that is, interconversion of M- and W-type isomers (Scheme 153). This process can proceed via a dissociative mechanism, or via intermediate η^1^-complexes and rotation around the C–Pd bond.^449^ A third possible mechanism involves coordination of an external ligand to form a five-coordinated Pd complex, which undergoes pseudorotation.^450^

Memory effects are sometimes observed as a result of preferential nucleophilic attack at the carbon atom originally carrying the leaving group, leading to retained chirality or constitution.^36,451^ Such memory effects may attenuate or reverse enantioselectivity and lead to different product ratios from enantiomeric substrates with the same catalyst. If equilibration of allyl complexes is rapid, no memory effects are observed, and the two enantiomers of the substrate give the same result. On the other hand, if nucleophilic attack competes with isomerization of the nonidentical η^3^-allyl Pd complexes obtained from the two enantiomers of the substrate, this leads to memory effects. Particularly strong memory effects were observed with bulky monodentate phosphine ligands.^36,451^ Different rationales for the origin of memory effects have been proposed.

**Scheme 154:**
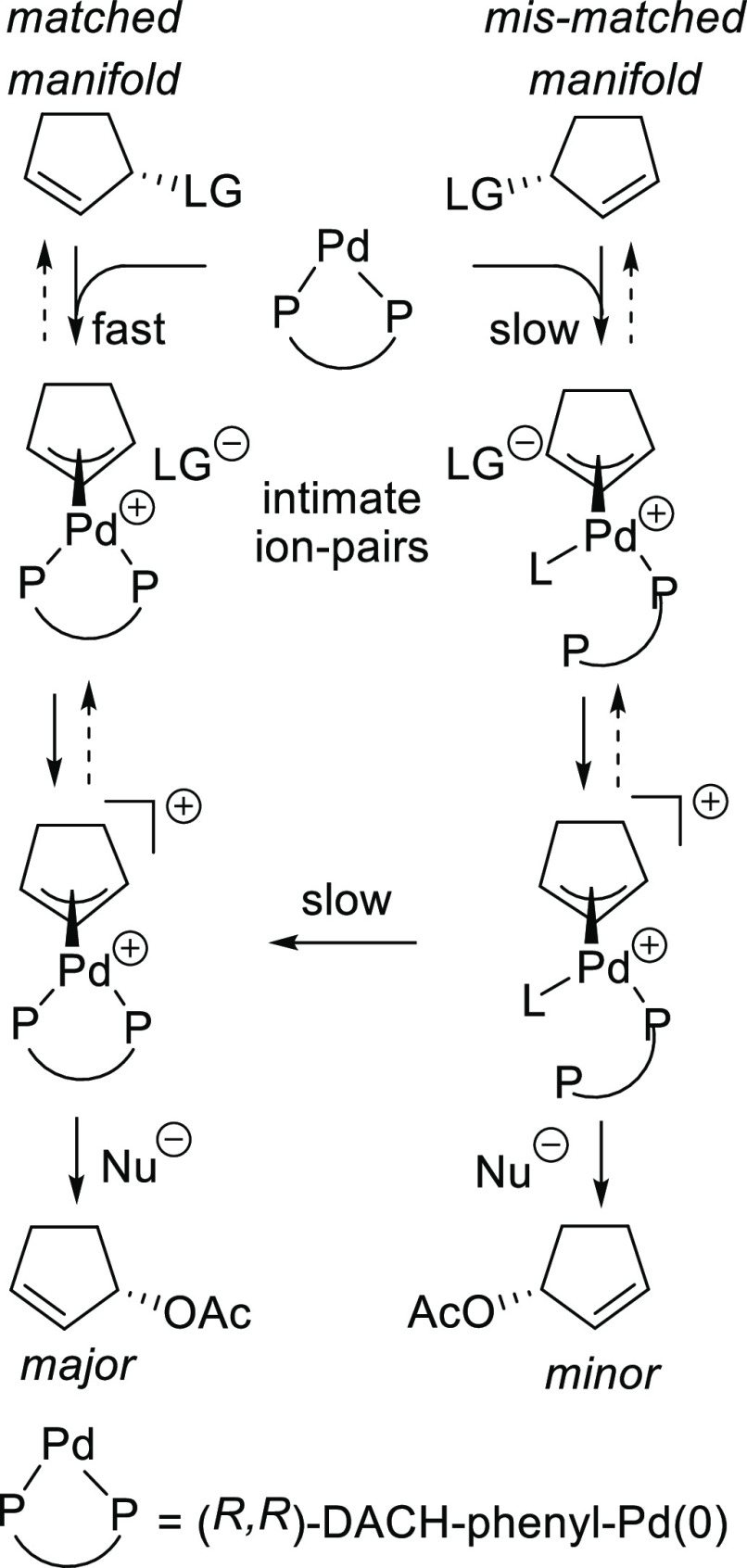
Matched and Mismatched Manifolds

For reactions with Pd-DACH catalysts, it was suggested that the observed memory effects resulted from formation of intimate ion pairs between the allyl complex and the leaving group (see Scheme 154). In these ion pairs, the leaving group stays close to the C atom to which it was originally bound and guides the nucleophile to this position by Coulombic interaction with the nucleophile counterion.^452^

Mechanistic studies by Lloyd-Jones and Stephen, employing ^2^H- and ^18^O-labeled cyclopentenyl esters as substrates together with chiral and achiral ligands, indicated that intimate ion pairs are indeed formed but are not the cause of memory effects.^453^ Chiral nonracemic ligands in combination with racemic substrates give rise to two manifolds, one matched and one slower-reacting mismatched manifold (Scheme 154). Ionization in the mismatched manifold is slow because of the disfavored torquo-selectivity induced by the DACH ligand. The resulting steric strain in the allyl complex may be reduced by dissociation of one of the Pd–P bonds, giving rise to a (P,L)-Pd η^3^-allyl complex with an enlarged coordination pocket (L = unspecified nonphosphine ligand). Formation of such a monophosphine complex, in which nucleophilic attack *trans* to the phosphine group is electronically favored, was proposed as a possible explanation of the memory effect.^453^

A considerable number of studies over the past decade have been devoted to the key aspects of this mechanism, mostly with the goal of finding a rationale for the observed regio- and the enantioselectivity. In this respect, catalyst design relies increasingly on structural information, and computational studies (primarily due to the advance in computational power and methods) are increasingly used, moving away from the costly and time-consuming trial-and-error based discovery. The computational approaches to this problem were reviewed by Kleimark and Norrby in 2012.^37^

From a general perspective, mechanistic studies on Pd-catalyzed allylic substitutions have been focused on enantiodetermining nucleophilic additions. Depending on the ligands involved, the nucleophile and the reaction conditions, the transition state (TS) can be either early or late.^454−457^ Recent studies involving a variety of heterodonor P,N- and P,S-ligands have shown the occurrence of early transition states for this step, and the stereoselectivity is therefore largely governed by the relative stability of the Pd η^3^-allyl complexes as well as by the electrophilicity of the allylic terminal carbon atoms under the conditions of the experiments. Consequently, structural elucidation of the Pd η^3^-allyl intermediates **19** and the quantification of their relative reactivity toward the nucleophile have been used to rationalize the catalytic behavior and the observed enantioselectivities in these cases. Actually, in the past decade, a significant part of the mechanistic investigations was focused on the study of Pd η^3^-allyl complexes with chiral ligands by a combination of NMR spectroscopic techniques, DFT calculations and X-ray crystallography. NMR spectroscopy and DFT calculations are much more complex for monodentate than for bidentate ligands, due to the high conformational flexibility of the involved species. To overcome this difficulty, Thiele and co-workers demonstrated that the structure of the key intermediate can be determined by combining the results of a preliminary computational study with the determination of residual dipolar couplings (RDC).^73,458^ Alternatively, when a late TS is operative, the enantioselectivity of the reaction may be explained by the relative stability of the Pd-olefin complexes **20**; in this case, the formation of the most stable Pd-olefin complex controls the enantioselectivity of the process. The relative stability of the Pd-olefin complexes, as well as that of the Pd η^3^-allyl complexes used for estimating the selectivity in processes with early TS, may however differ from the relative energy of the TSs, so that for more accurate results the different transition states potentially involved in the processes need to be computationally characterized using reliable (DFT) procedures.

**Scheme 155:**
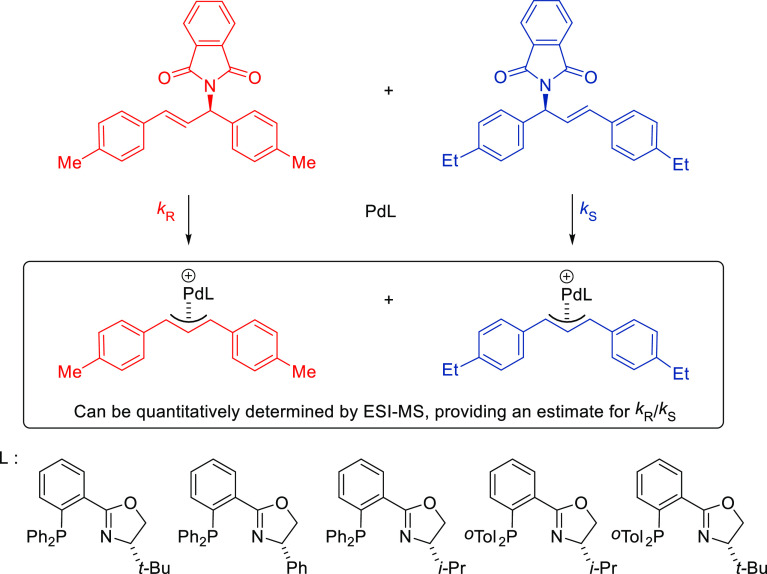
Mass Spectrometric Catalyst Screening of Pd-Catalyzed Allylic Alkylation Reactions by Monitoring the Back Reaction

The occurrence of Pd-allyl intermediates in reaction media can be easily monitored by ESI-MS. On the basis of the mass spectrometric quantification of allyl intermediates, a new method for screening chiral Pd catalysts in asymmetric allylic substitutions was developed by Pfaltz and co-workers.^459−462^ By monitoring the back reaction of quasienantiomeric allylation products, it was possible to determine the enantioselectivity from the observed ratio of the mass-labeled Pd η^3^-allyl complexes arising from the corresponding quasienantiomeric allylation products. (Scheme 155). According to the principle of microscopic reversibility, this ratio corresponds to the enantioselectivity of the forward reaction.^460^ In the same way the selectivity factors in the kinetic resolution of allylic acetates can be determined.^459^ Subsequently, back reaction screening was also successfully applied to various other catalytic reactions.^461^ Screening by ESI-MS is fast and operationally simple, as it does not require workup or purification steps and only minimal amounts of substrate are needed. Moreover, mixtures of catalysts with different molecular masses can be screened simultaneously, which is not possible with methods relying on product analysis.

Mass spectrometric studies also revealed that dinuclear allyl-bridged Pd^I^ complexes are formed reversibly during allylic substitution reactions.^462^ These complexes, which were characterized by NMR spectroscopy and crystal structure analysis, represent a reservoir, from which catalytically active mononuclear Pd^0^ and Pd^II^ complexes are released under the reaction conditions.

Pd-catalyzed allylic alkylation reactions were initially carried out with chiral bidentate diphosphines as ligands. However, in contrast to their high effectiveness in asymmetric hydrogenation, only a few diphosphines provided useful enantioselectivities. The low efficiency of these ligands was attributed to the fact that the enantiodiscriminating nucleophilic attack on the Pd η^3^-allyl intermediate occurs outside the coordination sphere, and thus makes it difficult for the ligand to control the stereochemical course of the process.^449^ New successful ligands appeared later. Here they are grouped into four main categories based on the underlying design principles. In the following, we discuss the most representative advances for each ligand category.

**Figure 10:**
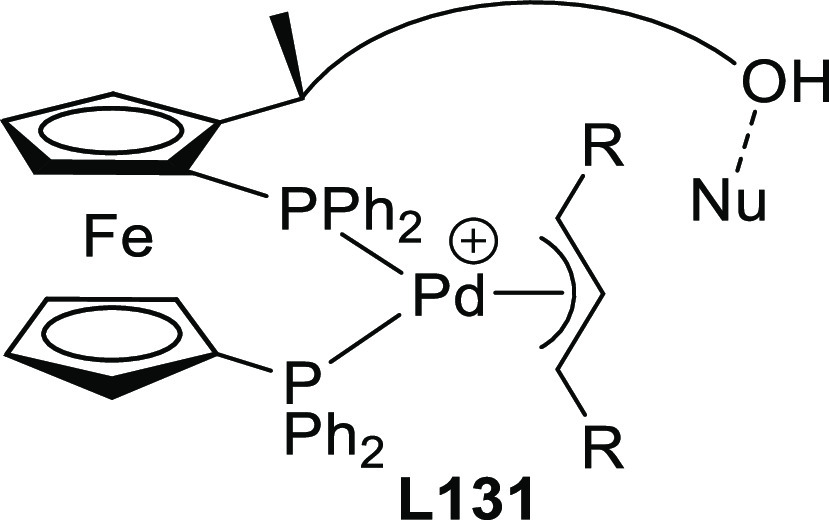
Ferrocene diphosphine ligand **L131** directing nucleophilic attack to one of the allylic termini by a secondary ligand–nucleophile interaction.

In the first category the design strategy relies on secondary interactions of the nucleophile with the chiral ligand, able to direct the nucleophilic attack toward one of the allylic terminal carbon atoms. For example, Hayashi and co-workers introduced a side chain in the diphosphine ferrocene-based ligand **L131** (Figure 10) with the appropriate length to form a link to the nucleophile by hydrogen bonding and preferentially guiding nucleophilic attack to one of the allylic termini.^463^ This was the first example of a ligand that induced significant enantioselectivity (81% *ee*) in the allylation of 1,3-dicarbonyl compounds.

**Figure 11:**
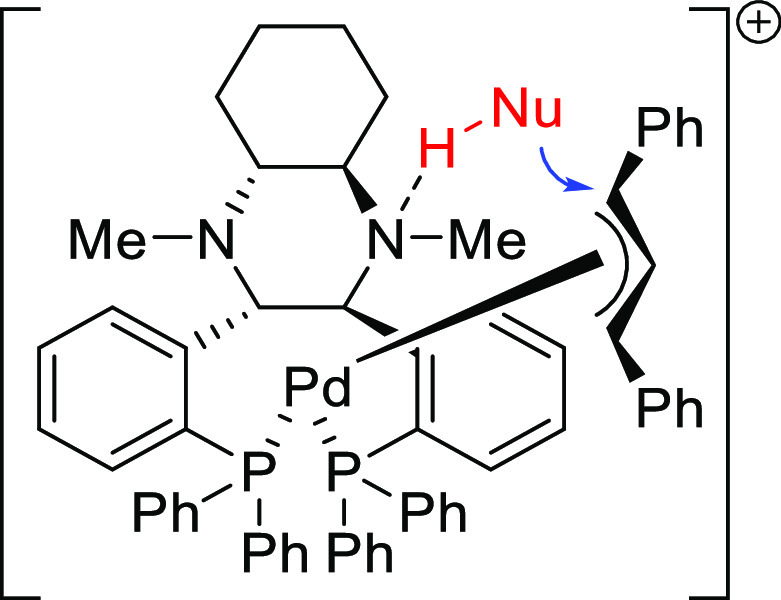
Proposed model of the Pd-catalyzed allylic substitution using the Pd/Fei-Phos catalyst.

In the past decade, further examples were reported showing how hydrogen bonding interactions between the nucleophile and the ligand, in combination with the steric effects conferred by the ligand, direct the attack of the nucleophile to one of the allylic terminal carbon atoms in reactions of symmetrically substituted allylic substrates. Secondary interactions such as hydrogen bonding or ion pair formation have also been implemented in other design strategies (see, e.g., the Trost ligand discussed below). Recent examples include ligand **L33** (Fei-Phos ligand, Figure 11) that provided high *ee* values with a variety of C-, O-, and N-nucleophiles.^125,126^ From X-ray, ESI-MS, and NMR spectroscopic studies, Xu and co-workers concluded that it is a hydrogen bond with the amino group of the ligand that directs the nucleophile toward one of the enantiotopic allylic termini.

Strategies based on secondary interactions have also been applied to control the regioselectivity in unsymmetrical allyl systems, for instance, by attaching hydrogen donors to the allylic substrate, which can steer the nucleophile in the desired direction through hydrogen bond formation. While regioselectivity in this case is determined by substrate control, enantioselectivity results from the interaction of the allyl system with the chiral ligand. This approach was pioneered by Trost who observed a switch from linear to branched products when an alkoxide group was attached at one end of a linear allylic system.^464^ Further examples of regiocontrol by directing groups acting through hydrogen bonding, ionic or other electronic interactions have also been reported and this has been the subject of a recent review.^465^

**Figure 12:**
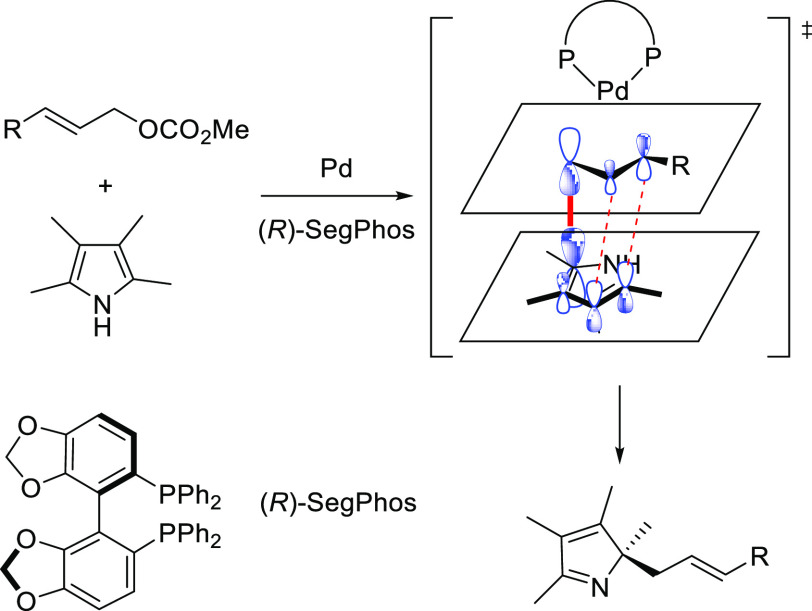
Orbital interactions in the TS of the regio- and enantiodetermining step in the asymmetric allylic dearomatization of multisubstituted pyrroles.

Regio- and enantioselectivity can also be controlled by orbital interactions between the nucleophile and the allyl system, as shown by Zheng, Zhuo, and You, who studied the origin of the remarkable regio- and enantioselectivity in the Pd-catalyzed asymmetric allylic dearomatization of multisubstituted pyrroles with the diphosphine (*R*)-SegPhos (Figure 12).^141^ The results of density functional theory (DFT) calculations, which were in line with the observed selectivity, indicated that orbital interactions strongly influence the reaction course, while steric effects seem to play a minor role. Bond formation preferentially occurs at the positions with the largest coefficient in the HOMO of the pyrrole π-system and the LUMO of the allyl π-system. In contrast to most allylic substitutions reported in the literature, in which the catalyst controls the formation of a stereogenic center in the allyl system, in this case the enantioselectivity results from generation of a stereogenic center in the nucleophile. The chirality transfer from the chiral catalyst to the nucleophile occurs in an indirect manner. While the catalyst binds the allyl system selectively at one of the enantiofaces, orbital interactions between the pyrrole π-system and the allyl system, which functions as a relay, control the formation of the stereogenic center in the product.

**Figure 13:**
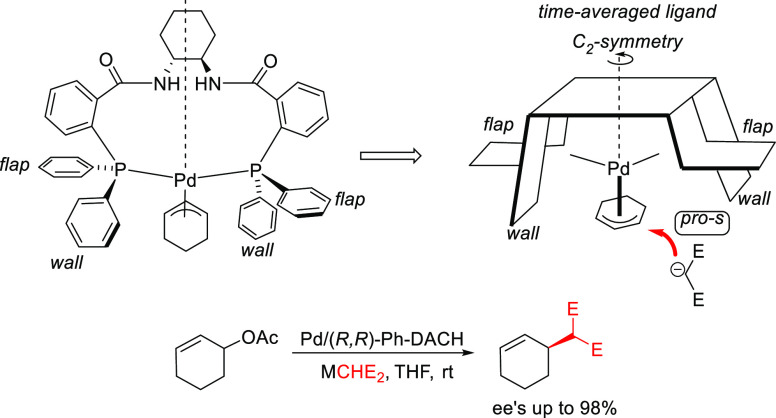
Trost’s wall-and-flap model rationalizing the stereochemical course of the Pd-catalyzed AAA reaction of cyclohexenyl acetate using Pd/(*R,R*)-Ph-DACH as catalyst.

A second successful design principle was introduced by Trost with the development of diphosphine ligands, such as (*R,R*)-Ph-DACH, which bind to the Pd center exclusively through the phosphorus atoms.^45,466−468^ The basic idea was to increase the ligand’s bite angle by enlarging the chelate ring, thus creating a more confined chiral cavity, which interacts more strongly with the substituents of the allyl system and the nucleophile. Ligands of this type represent one of the most effective ligand families for asymmetric allylic substitution, which has found widespread use in natural product synthesis. The mechanistic model that was originally proposed to explain the observed enantioselectivities is shown in Figure 13.^469^ According to this model the enantioselectivity results from steric interactions with the four P-phenyl groups forming the chiral cavity, which block one of the allylic termini against nucleophilic attack. The model was in accordance with the observed absolute configuration of the products and also provided a possible explanation for why substrates with small substituents at the allylic C atoms, such as unsubstituted cycloalkyl acetates or 1,3-dimethyl acetate, gave high enantioselectivities and high yields, while sterically more demanding substrates, which do not fit into the chiral cavity, such as 1,3-diphenylallyl acetate, reacted sluggishly and with low enantioselectivity. However, it was not clear why the sodium salt of diethyl malonate gave much lower *ee* than analogous tetraalkylammonium salts.

**Figure 14:**
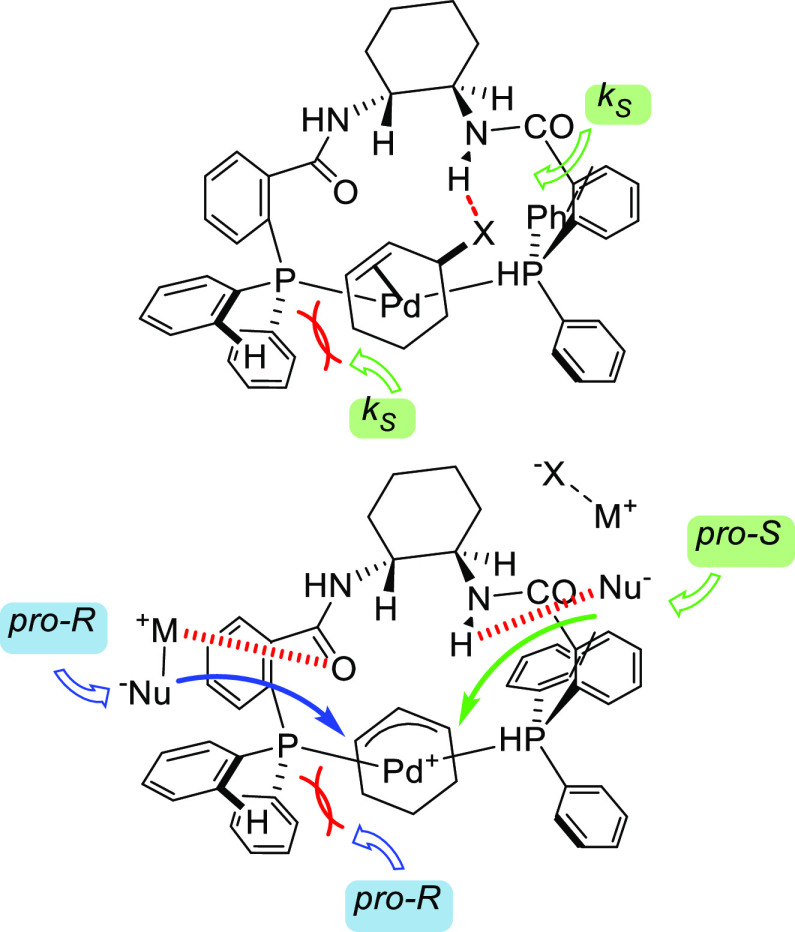
Pictorial representation of the Lloyd-Jones/Norrby transition state model for Pd-AAA reactions rationalizing the enantioselectivity in kinetic resolutions (top) and allylic alkylations (bottom).

A refined model, which rationalized the observed counterion effect and also provided a deeper insight into the relevant enantioselectivity-determining interactions, was reported in 2009 by Lloyd-Jones, Norrby, and co-workers.^470^ The underlying work comprised the elucidation of the solution-phase structures of the cationic η^3^-propenyl- and η^3^-cyclohexenylpalladium complexes with ligand (*R,R*)-Ph-DACH by a combination of NMR spectroscopic studies, isotopic labeling and DFT calculations. On the basis of these studies and additional experiments, the model shown in Figure 14 rationalizes the observed enantioselectivities in kinetic resolutions and allylic alkylation reactions. According to this model, three factors govern the regioselectivity (pro-*S* vs. pro-*R*) of nucleophilic attack on the Pd η^3^-cyclohexenyl complex and, thus, the *ee* of the product: (i) a pro-*R* torquoselective bias is induced by steric interaction of the η^3^-cyclohexenyl moiety with one phenyl ring of the ligand; (ii) pro-*S* delivery of the nucleophile is favored by hydrogen-bonding with the concave oriented amide N–H; and (iii) pro-*R* delivery of the nucleophile is favored by the counterion (M^+^) in salt-type nucleophiles, binding to the concave orientated amide carbonyl group. As the result of the latter two opposing interactions, the enantioselectivity is sensitive to the nature of X^–^ and M^+^. This explains the observed strong counterion effect mentioned above. With the sodium salt of diethyl malonate the pro-*S* and the pro-*R* pathway compete, resulting in low enantioselectivity, whereas the corresponding tetraalkylammonium salts do not bind to the amide carbonyl groups and, therefore, approach the allyl system with high preference in the pro-*S* direction. In kinetic resolutions, the N–H bond in the concave region of the [Pd-(*R,R*)-Ph-DACH]^+^ complex is able to activate the leaving group of the allylic ester by hydrogen bonding to its carbonyl group. This interaction, only feasible for the (*S*)-enantiomer of the substrate, is expected to induce a highly selective kinetic resolution, in full agreement with experimental results. The results of this study demonstrate that the enantioselectivity induced by Trost’s ligands results from an interplay between steric interactions imposed by the chiral cavity of the ligand and H-bond and electrostatic interactions of the amide groups with the nucleophile. Moreover, the model shown in Figure 14 also provides a basis for the development of new ligands.

With the aim of adapting the Trost ligand to reactions with sterically demanding substrates, Hitchcock and co-workers replaced one of the amido groups by an ester group (ligand (*S*)-**L31**, Scheme 38).^122,471^ As a result, the *tert*-leucinol-derived diphosphine (*S*)-**L31** provided excellent *ee* values (up to 99%) in the allylic alkylation of dimethyl and diethyl malonate with 1,3-diphenylallyl acetate. Mechanistic studies confirmed that nucleophilic attack is assisted by hydrogen bonding with the amido group.

**Scheme 156:**
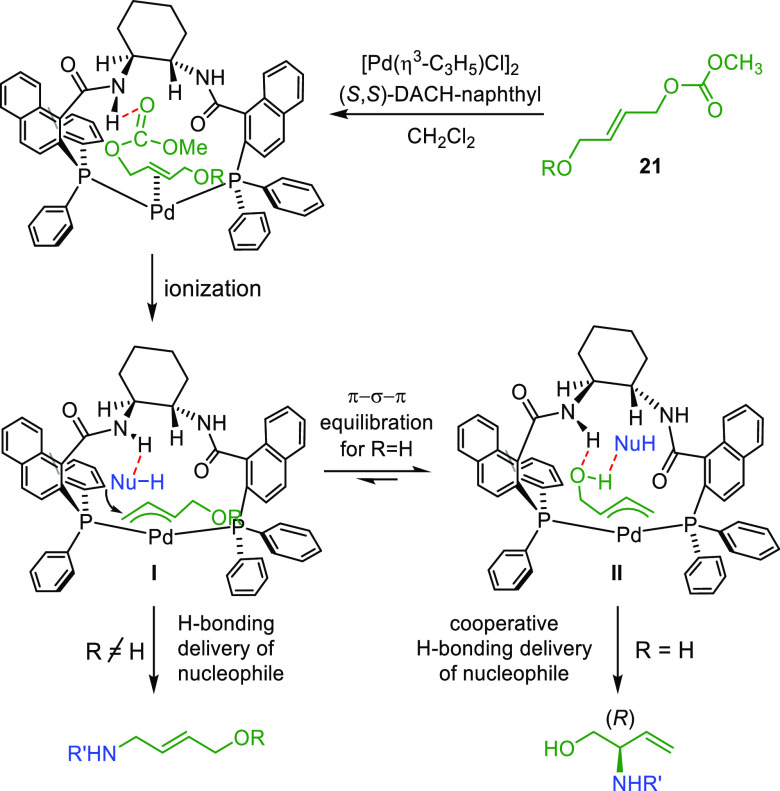
Control of the Regioselectivity in the Pd-Catalyzed AAA Reaction by Secondary Interactions between the Substrate and the Ligand

In the course of their studies toward an enantioselective synthesis of fagomine,^472^ Castillón, Díaz, and co-workers, found that very high enantio- and regioselectivity (98% *ee*, >98:2 branched:linear) was induced in the Pd-catalyzed allylic amination of hydroxy-functionalized allyl carbonate **21** (R = H) by the Trost ligand (*S,S*)-DACH-naphthyl (Scheme 156). The results have been explained by hydrogen bonding between the hydroxy group in the substrate and one of the amido groups of the ligand. Consistent with this rationale, a dramatic change in regioselectivity was observed, when the hydroxy group in **21** was protected or replaced by an alkyl chain (>98/2 l:b; R = trityl).

**Scheme 157:**
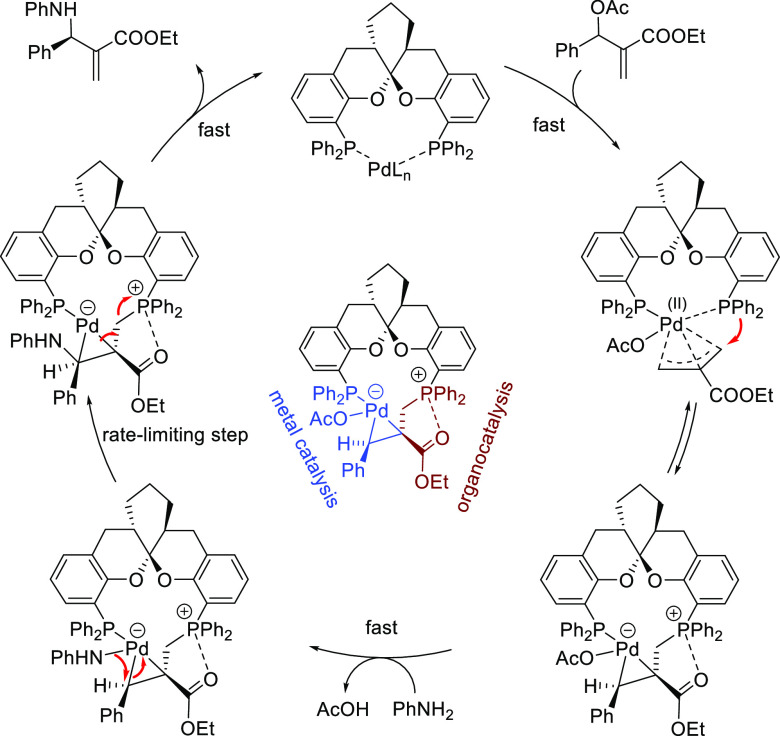
Proposed Dual Catalytic Mode (Metal Catalysis + Organocatalysis) in the **L34**/Pd-Catalyzed AAA of Morita–Baylis–Hillman Adducts

Ding and co-workers found that spiroketal-based diphosphine ligands such as (*S*,*S*,*S*)-**L34** displayed very high efficiency (TON > 4700, branched/linear: 97/3, 92% *ee*) in the Pd-catalyzed asymmetric allylic amination of Morita–Baylis–Hillman adducts.^135^ Crystal structure data showed that the intramolecular P,P distance in the **L34** ligand is much larger (6.29 Å) than in conventional diphosphines. This finding prompted a detailed mechanistic study, which provided evidence for an unusual reaction course that strongly differed from the commonly accepted catalytic cycle shown in Scheme 151. It was concluded that due to the long P,P distance the chelate ring was less stable than in conventional diphosphine complexes, and as a result, one of the phosphine groups could easily dissociate from the Pd atom. Consequently, it was proposed that the free and the coordinated phosphine group fulfill a dual cooperative function (Scheme 157). Thus, one of the phosphorus atoms acts as a nucleophile, forming a temporary C–P σ-bond with the terminal carbon atom of the allyl moiety, while the other phosphorus atom coordinates to Pd. In contrast to the standard reaction mode of heteronucleophiles like amines, in this case the C–N bond is formed by transfer of the nucleophile from the Pd atom to the substrate rather than back-side attack. The reaction, which displays high turnover numbers, excellent regioselectivity and very high enantioselectivity may be formulated as a hybrid of an organo- and metal-catalyzed process.

There is a third category of ligands, which neither form a chiral cavity around the metal center nor possess a functionalized side chain that can interact with the nucleophile, but still induce high enantioselectivity in allylic substitutions with symmetrically substituted allyl substrates. The regioselectivity of nucleophilic attack in this case results from interactions of the ligand with the allyl system, which influence the reactivity at the terminal carbon atoms.

**Figure 15:**
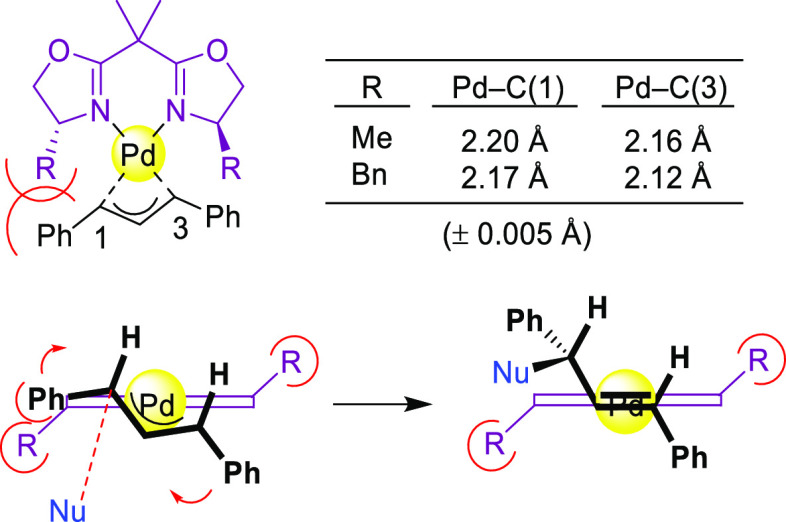
Steric effects responsible for the enantioselectivity of Pd-BOX catalysts.

X-ray crystallographic and NMR spectroscopic studies of Pd allyl complexes with C_2_-symmetric bisoxazolines have revealed how repulsive steric interactions can selectively enhance the reactivity at one of the allylic termini by lengthening one of the Pd–C bonds (Figure 15).^473^ From the absolute configuration of the allylation product, which is formed with high *ee*, it can be inferred that the nucleophile preferentially attacks the longer, more strained Pd–C bond. Moreover, steric interactions between the allylic phenyl groups and the substituents at the stereogenic centers of the ligand also promote rotation of the allyl system in the direction indicated in Figure 15, leading to a reduction of steric strain. Of particular note, rotation in the opposite direction, taking place upon nucleophilic attack at the other allyl terminus, would result in strain increase.,^474^^475^

**Figure 16:**
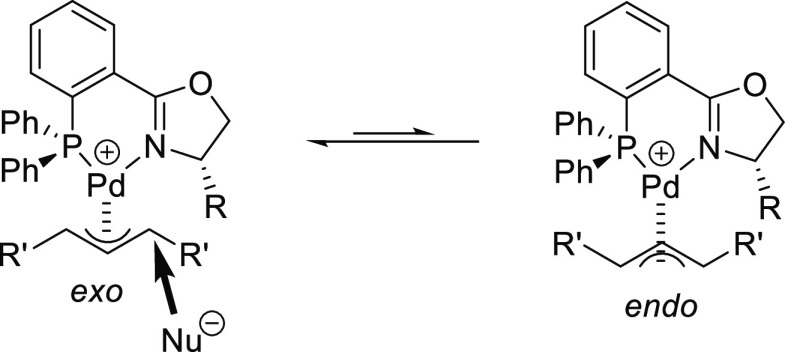
Nucleophilic attack *trans* to the donor atom with the strongest *trans* influence in Pd-allyl complexes with PHOX ligands.

The reactivity at the allylic termini can also be modulated by electronic interactions with the ligand, which are transmitted by the *trans* influence of the donor atoms coordinated to the metal center.^47^ If the Pd atom is coordinated by two electronically different donor atoms, the allylic termini become electronically nonequivalent and, thus, are expected to exhibit different reactivity. On the basis of this concept, phosphinooxazoline (PHOX) ligands with a nitrogen and a phosphorus donor atom were developed by the groups of Helmchen, Pfaltz, and Williams (Figure 16).^42−44,476^ Crystal structure data of allyl Pd-PHOX complexes revealed that the Pd–C bond *trans* to the P atom is distinctly longer than the bond *trans* to the N atom, indicating enhanced reactivity. Under the usual reaction conditions, the Pd allyl intermediates rapidly equilibrate between the *exo* and *endo* forms. Extended NMR spectroscopic studies demonstrated that nucleophilic attack preferentially takes place on the more stable *exo* isomer at the longer Pd–C bond *trans* to the P atom.^476^

Subsequently, many other heterodonor ligands, mainly P,N-ligands (P = phosphine or phosphinite, N = oxazoline, pyridine, oxazole, imidazole, etc.) have been developed.^10^ While these ligands induce high *ee* values in allylic substitutions with sterically demanding substrates, such as 1,3-diphenylallyl acetate, most of them give only moderate to low enantioselectivities with substrates having small substituents on the allyl system, such as cycloalkenyl or 1,3-dimethylallyl esters. In this respect, the P,N ligands and the Trost diphosphine ligands have complementary scope.

**Figure 17:**
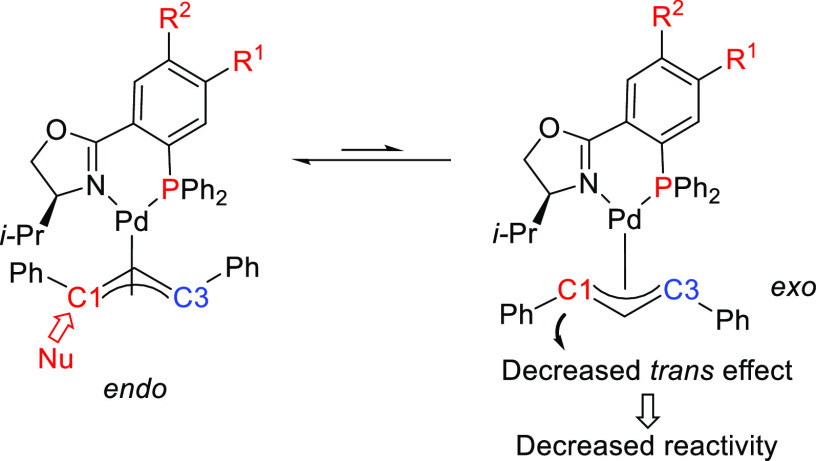


In 2012, Bunt and co-workers reported a refined study of the electronic origin of asymmetric induction in Pd-catalyzed allylic substitutions with PHOX ligands based on linear free energy relationships (LFER) and NMR analyses of the corresponding (η^3^-1,3-diphenylallyl) Pd intermediates (Figure 17).^477^ By variation of the R^1^ and R^2^ substituents, they proved how electronic effects influenced the regioselectivity of nucleophilic attack. By Hammett analysis of the ^13^C NMR chemical shifts of the C1 and C3 allylic carbon atoms, they could show that the corresponding signals of the major *endo* complex were little affected from changing the substituents on the aryl unit, while the corresponding signals of the minor *exo* complex shifted substantially. From these results, it was concluded that the *trans* effect in the *exo* isomer is weaker, explaining its lower reactivity and why the enantioselectivities achieved with PHOX ligands usually exceed the *endo*/*exo* ratios observed in reaction solutions. Swain–Lupton analysis of the NMR data also revealed the importance of both resonance and field effects by the R^1^ and R^2^ substituents regardless of their location and supported the overall electronic control model for enantioselection by PHOX ligands.

**Scheme 158:**
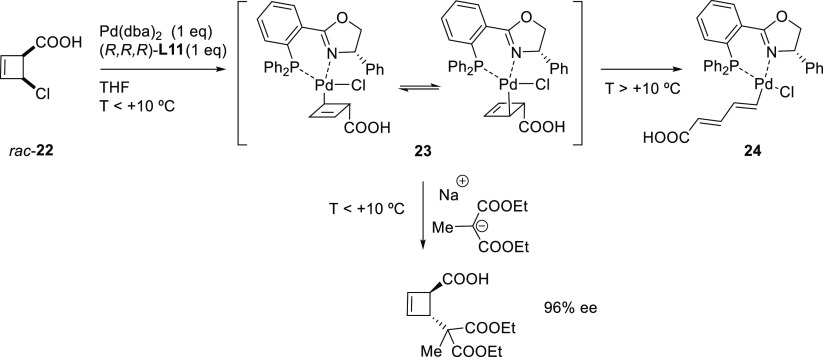
Formation of η^1^-Allyl Complexes and Subsequent Electrocyclic Ring Opening

Using Pd-PHOX catalysts, Maulide and co-workers developed an enantioselective diastereodivergent synthesis of 3,4-disubstituted cyclobutenes through a deracemizing Pd-catalyzed asymmetric allylic alkylation of *cis*-4-chloro-2-cyclobutenecarboxylic acid (**22**) (Scheme 158).^75^ Both *rac-***22** and the corresponding *trans* isomer are converted to the *trans* product with high enantioselectivity. Remarkably, the same reaction with a chiral Pd-phosphoramidite catalyst led to the corresponding *cis* product. Mechanistic studies^199^ showed that the reaction proceeded through Pd allyl intermediates, which existed in the rarely observed η^1^-bonded form as rapidly equilibrating stereoisomers (2 *cis* and 2 *trans* isomers; **23**). These intermediates are stable at low temperature and are efficiently trapped by nucleophiles, whereas above 10 °C they undergo an unprecedented electrocyclic ring opening to the Pd vinyl complex **24**.

**Figure 18:**
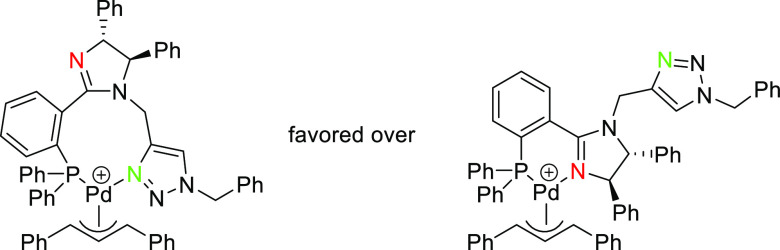
Change of the palladium coordination mode in a phosphinoimidazoline (PHIM) complex induced by a remote triazolyl group.

While evaluating phosphinoimidazolines (PHIM) as potential alternative ligands to phosphinooxazolines in Pd-catalyzed asymmetric allylic aminations, Pericàs, Claver, Castillón and co-workers found that attachment of a triazolylmethyl unit to the imidazolidine ring led to a significant increase in enantioselectivity (up to 99% *ee*).^256^ A combined computational (DFT) and NMR (NOESY) spectroscopic study of the intermediate Pd η^3^-diphenylallyl complexes showed that the remote triazole ring induced a dramatic change in the coordination mode by replacing the oxazoline ring as the coordinating unit, indicating that the formation of an enlarged chelate ring was responsible for the increased enantioselectivity (Figure 18).

**Figure 19:**
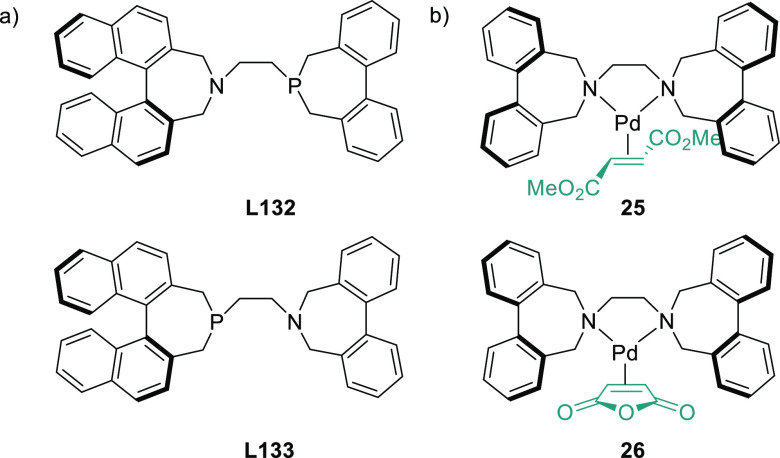
(a) Flexible phosphepine and azepine ligands **L132** and **L133** and (b) Pd-olefin complexes with *C*_2_- and *C*_s_-symmetry, respectively.

A fourth design principle, which has emerged from the search for ligands displaying wider substrate scope, focuses on conformational flexibility. Traditionally, chiral ligands were designed based on conformationally rigid structural elements that allow straightforward prediction of steric interactions with a substrate. However, more recently evidence has been accumulated that a certain degree of flexibility can be beneficial for inducing enantioselectivity.^478^ The work of Moberg and co-workers with flexible phosphepine and azepine ligands **L132** and **L133** is an example (Figure 19a).^479,480^ Through a combined in-depth NMR spectroscopic and DFT study of the conformational behavior of these ligands in Pd(II)-allyl and Pd(0)-olefin complexes, they showed that the ligands adapt their conformations to the structure of a bound substrate.^481^ By using analogous bis-azepine ligands containing two conformationally flexible biaryl moieties as models (Figure 19b), they found that in Pd-olefin complex **25**, mimicking the olefin complexes from the reaction of hindered linear substrates, an *R*,*R* (*C*_2_) configuration was adopted. On the other hand, in Pd-olefin complex **26**, mimicking olefin complexes from the reaction of unhindered cyclic substrates, an *R*,*S* (*C*_S_) configuration was adopted. In contrast, in the Pd η^3^-allyl complexes an *R*,*S* configuration of the ligand was observed for (*E*)-1,3-diphenyl-2-propenyl acetate and also for 3-cyclohexenyl acetate. However, this self-adaptation mode proved to be less effective than desired because the conformational changes in ligands **L132** and **L133** were slow in comparison with nucleophilic attack. Therefore, the flexible ligands behaved essentially as a mixture of the analogous rigid ligands.^482^

**Figure 20:**
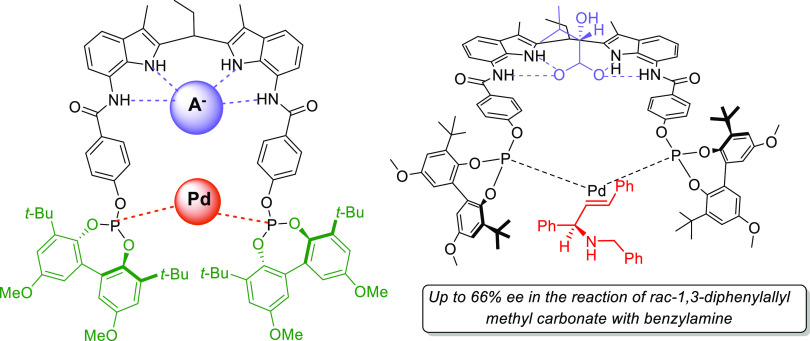
Model for the cofactor-controlled chirality of the tropos ligand **L134** (left) and working model for prediction of enantioselectivity (right).

Metal complexes based on flexible tropos ligands,^483^ which rapidly equilibrate between enantiomeric axially chiral conformers, have been successfully used in a variety of enantioselective catalytic processes. Moberg, Reek and co-workers developed a new type of tropos ligand (**L134**), which was used in Pd-catalyzed AAA reactions.^153^ The ligand features an integrated anion receptor site, which, upon complexation with chiral anions, such as (*S*)-2-hydroxy-3-methylbutyrate or 6,6′-disubstituted BINOL-derived phosphates, acting as cofactors, exerts control over the chirality of a Pd complex (Figure 20, left). The ability of the chiral anions to determine the conformation of the flexible biaryl phosphite units was demonstrated by the formation of enantiomerically enriched products in the Pd-catalyzed substitutions of allylic carbonates with sodium dimethyl malonate and benzylamine. ^1^H and ^13^C NMR spectroscopic studies led to a model that explains the observed enantioselectivities (Figure 20, right).

**Figure 21:**
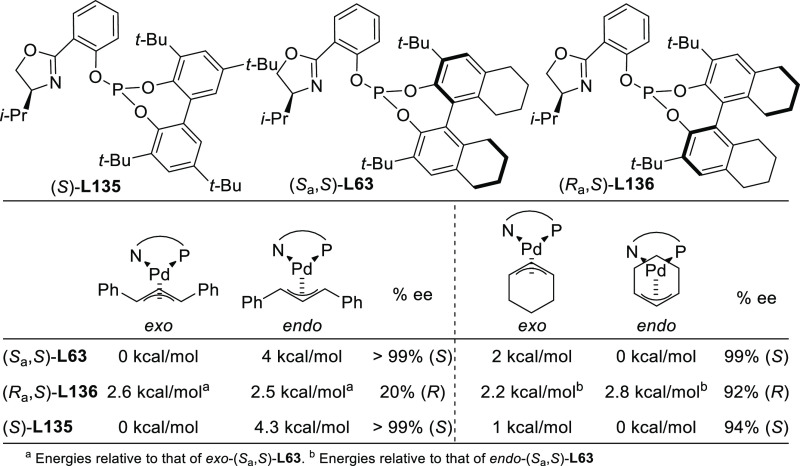
Calculated relative energies (in kcal/mol) for the transition states of nucleophilic attack at *exo* and *endo* Pd η^3^-allyl intermediates for 1,3-diphenylallyl and cyclohexenyl acetates using NH_3_ as nucleophile. Experimental enantioselectivites (in % *ee*) achieved in the allylic alkylation of 1,3-diphenylallyl and cyclohexenyl acetates using dimethyl malonate.

Another approach to self-adaptable Pd-catalysts that overcomes the limited substrate scope of Pd-catalyzed allylic substitutions was based on the introduction of a flexible biaryl phosphite group into heterodonor P,N ligands.^16,484^ The first examples of this approach were the PHOX derivatives **L63** (Figure 4), **L135** and **L136** (Figure 21), in which the phosphine group had been replaced by biaryl phosphite moieties.^49,229^ Pd complexes of these ligands proved to be very effective catalysts for reactions of both hindered and unhindered linear and cyclic substrates, outperforming Pd-PHOX catalysts, which give outstanding enantioselectivities with *rac*-(*E*)-1,3-diarylallyl substrates, moderate to good enantioselectivities with 1,3-dialkylallyl substrates but provide essentially racemic products with cyclic substrates.^485^ The wide substrate scope of the Pd/**L63** system was rationalized by NMR spectroscopic studies and DFT calculations of its Pd-η^2^-olefin and Pd-η^3^-allyl complexes.^229^ In contrast to previously reported flexible ligands, it was found that the biaryl phosphite group in ligand **L135** adopts an (*S*)-configuration in the complexes mimicking product olefin complexes of hindered as well as unhindered substrates. Although the olefins coordinated with the same face to Pd in complexes with the corresponding rigid ligands **L63** and **L136**, products with opposite absolute configuration were obtained because of the different energies of the transition states of nucleophilic attack at the Pd η^3^-allyl intermediates (Figure 21). These findings indicate that the broad substrate scope of Pd/biaryl phosphite-oxazoline systems results from their capacity to adjust the size of the binding pocket to the substrate type, a feature that also explains their excellent performance in other asymmetric reactions.^486−488^

Subsequently, a large variety of heterodonor biaryl phosphite-containing ligands, mainly belonging to the P,N (N = oxazoline, pyridine, oxazole, thiazole, etc.)^197,227,228,230,255,273,489^ and P,thioether^299,302−304^ types, have been developed. Mechanistic studies of allylic substitutions with all these ligands show an early TS, in which the stereochemistry of the reaction is governed by the relative populations of the *exo* and *endo* Pd-η^3^-allyl complexes and the electrophilicity of the allylic terminal carbon atoms. In the following, we highlight two recent ligand families belonging to this category. One of them comprises P-thioether ligands **L99** derived from indene (Figure 7).^303^ Within this family, the use of DFT studies was crucial to identifying the optimal phosphite-thioether ligand **L99**, which provided excellent enantioselectivities for 40 substrates including linear and cyclic allylic esters and a broad range C-, N-, and O-nucleophiles. These studies also showed that in the case of linear substrates the enantioselectivity is mainly governed by the different reactivity of the *endo* and *exo* Pd allyl intermediates toward the nucleophile, rather than the population of the *endo* and *exo* isomers, as it was found for cyclic substrates.

The second family is formed by the phosphite-oxazoline ligands **L64** and **L65** (Figure 5), which displayed even broader substrate scope (70 compounds in total).^230^ Mechanistic studies by NMR spectroscopy and DFT calculations showed that the ratio of the Pd allyl intermediates that provide the two enantiomeric substitution products is influenced by the ligand design. The enantioselectivity is mostly governed by the relative ratio of the *endo* and *exo* isomers. However, while the ratio of *endo* and *exo* isomers of cyclic substrates is mostly controlled by the configuration of the phosphite moiety and the substituent in the alkyl backbone chain, the oxazoline substituent as well plays a key role in linear substrates.

**Scheme 159:**
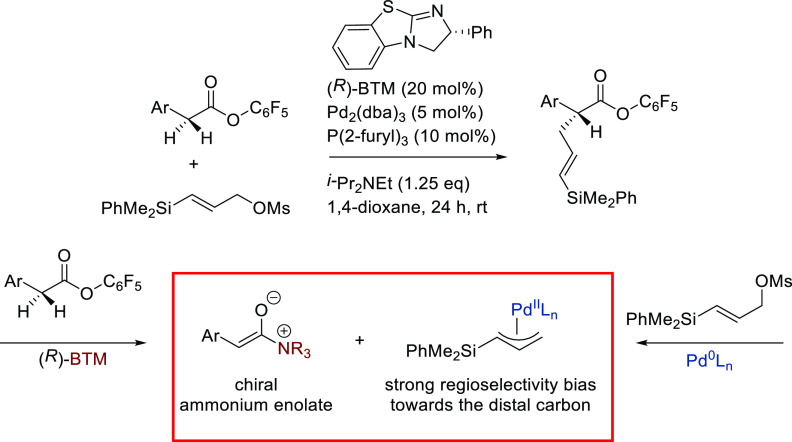
Cooperative Pd/Organocatalysis in the Alkylation of Silyl-Substituted Allylic Systems with Chiral Ammonium Enolates

As an alternative to enantiocontrol by a chiral Pd catalyst, enantioselectivity can also be induced by a chiral cocatalyst. The combination of a chiral or achiral Pd catalyst with a chiral organocatalyst that temporarily converts the nucleophile into a chiral reactant has emerged as a promising concept. The dual cooperative Pd/organocatalyst system developed by Snaddon and co-workers is an example (Scheme 159). It enables the direct enantioselective α-allylation of trifluorophenyl arylacetates with Si-substituted allyl mesylates (up to 88% *ee*).^402^ While the Si-substituent controls the regioselectivity, the enantioselectivity is induced by (*R*)-benzotetramisole, which generates a chiral ammonium enolate from the ester that adds to the achiral Pd allyl intermediate with tris(2-furyl)phosphine as an ancillary ligand.

**Scheme 160:**
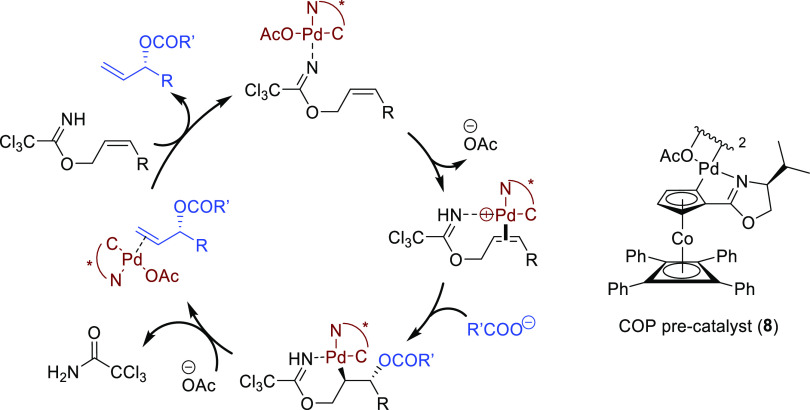
Proposed Mechanism of COP-Catalyzed Bimolecular Allylic Substitution Reactions

Finally, it should be noted that not all Pd-catalyzed allylic substitutions proceed by the commonly accepted mechanism shown in Scheme 151. The dual cooperative organo/metal-catalyzed reaction, shown Scheme 157, is such an example. A further remarkable example, the enantioselective synthesis of allyl esters, amides, or amines from (*Z*)-allyl trichloroacetamidates, was reported by Overman and co-workers (Scheme 160).^31^ A chiral cobalt oxazoline palladacycle (COP) **8** serves as catalyst in this case. Although the overall transformation corresponds to a Pd-catalyzed asymmetric allylic substitution, the catalytic cycle does not proceed via a Pd allyl intermediate but instead follows a novel course that involves an enantioselective nucleopalladation as the key step.

According to the proposed mechanism, which is based on experimental data and DFT calculations, the active catalyst is a monomeric Pd(II) complex.^490^ Coordination of the allylic imidate to the catalyst, which produces a cationic Pd(II) chelate complex, activates the alkene toward the external nucleophilic attack by the carboxylate anion. This nucleopalladation is the enantiodetermining step in the catalytic cycle. Deuterium labeling experiments showed that the overall reaction proceeds in an overall antarafacial fashion, which according to DFT studies results from an *anti*-oxypalladation/*syn*-deoxypalladation sequence.

**Scheme 161:**
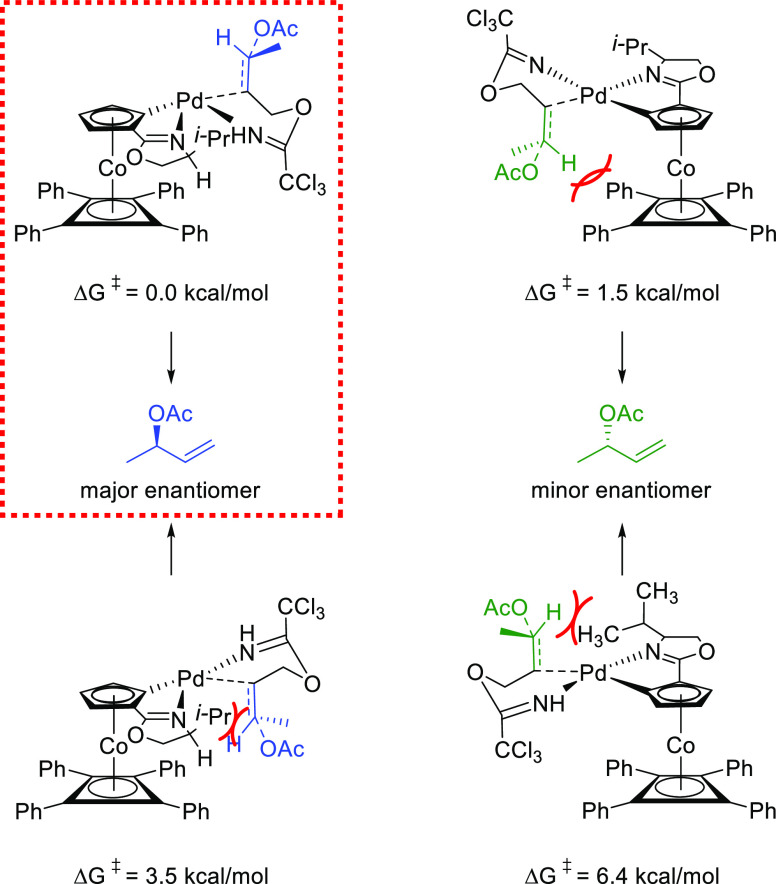
Model for Enantioselection in COP-Catalyzed Allylic Substitution Reactions

A mechanistic model for the observed enantioselection, which is based on computational studies, is shown in Scheme 161. According to this model, the tetraphenylcyclobutadiene moiety plays an overriding role in the enantioselectivity-determining step by forming an extended steric shield at the bottom side of the catalyst. The high enantioselectivities induced by the COP catalyst are remarkable, since antarafacial nucleopalladations are usually difficult to render enantioselective.

### 2.5 Application in Total Synthesis

For its excellent characteristics of broad scope, controllable regioselectivity, and high enantioselectivity and yield, the Pd-catalyzed reaction of allyl systems with nucleophiles has found ample application in the total synthesis of chiral, nonracemic compounds. The topic has been often reviewed in journals^8,24,491,492^ and book chapters,^33^ and encompasses synthetic operations such as the creation of quaternary all-carbon stereocenters and the formation of carbon–nitrogen and carbon–oxygen bonds at stereogenic centers. Since the aforementioned systems are racemization free or, at least, not racemization prone, metal-mediated asymmetric allylic substitutions are normally found in early stages of total synthesis and serve quite often to determine not only the absolute configuration of the different intermediates along the synthetic pathway, but also to define the whole synthetic strategy. Making an analogy with chess, it could be said that these reactions are opening moves rather than end games.

In this section, we have covered contributions from the period 2008–2020. The material has been organized according to the nature of the bond being created (C–C, C–N, C–O, and C–S). For succinctness, the discussion is mostly focused on the asymmetric allylic substitution step, rather than discussing the complete syntheses in detail.

#### 2.5.1 Carbon Nucleophiles

**Scheme 162:**
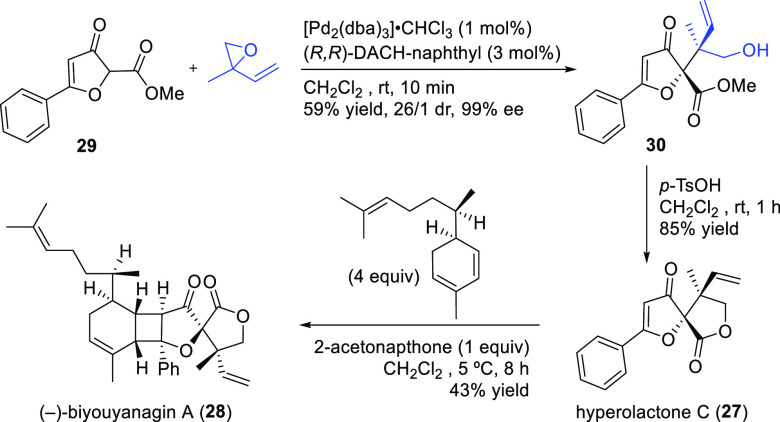
Synthesis of Hyperolactone C (**27**) and (−)-Biyouyanagin A (**28**)

Xie and co-workers reported in 2009 the total synthesis of the bioactive terpenoids hyperolactone C (**27**) and (−)-biyouyanagin A (**28**).^493^ The key step in the synthesis (Scheme 162) was the alkylation of isoprene monoepoxide with the dicarbonyl intermediate **29** using the chloroform complex of Pd_2_(dba)_3_ as the Pd source and the Trost (*R*,*R*)-DACH-naphthyl ligand. The reaction took place with 2.3/1 regioselectivity in favor of the branched isomer **30**, which could be isolated in 59% yield with excellent diastereo- (26/1) and enantioselectivity (99% *ee*). Remarkably, this was the first case when a Pd-catalyzed AAA reaction was used to install two vicinal quaternary carbon centers. Then, treatment of **30** with a catalytic amount of *p*-toluenesulfonic acid in CH_2_Cl_2_ at room temperature promoted fast conversion into **27** (85% yield). The conversion of hyperolactone C into natural (−)-biyouyanagin A (**28**) was readily achieved in 43% yield by photochemical [2 + 2] cycloaddition with *ent*-zingiberene.

**Scheme 163:**
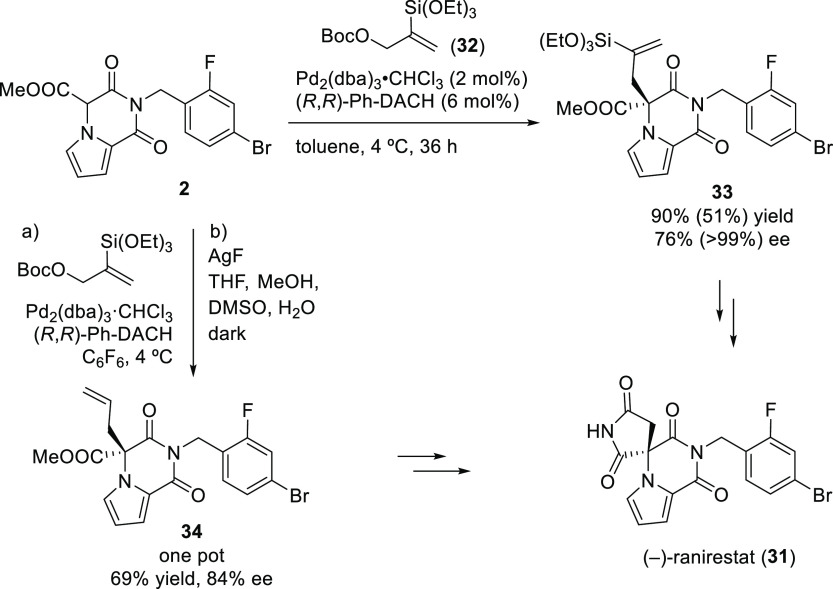
Synthesis of (−)-Ranirestat (**31**)

The enantioselective formation of a quaternary stereocenter was also the key step in the synthesis of (−)-ranirestat (**31**), a potent aldose reductase inhibitor.^97^ Trost and co-workers developed a concise, enantioselective approach to **31** involving as the key step a Pd-catalyzed asymmetric allylic alkylation of diketopiperazine **2** with 2-triethoxysilylallyl carbonate **32** using (*R*,*R*)-Ph-DACH as the chiral ligand. This resulted in the formation of the tetrasubstituted stereogenic center that would later become the spiranic center in the target molecule (Scheme 163). The product of this reaction (**33**) was obtained in 90% yield with an enantiomeric purity of 76% *ee*. Interestingly, a single recrystallization allowed enantioenrichment to >99% *ee*. Intermediate **33** was converted to **31** through a short sequence involving desilylation as the first step. It was also possible to access the desilylated product (**34**) directly from **32** by performing the Pd-catalyzed allylation and the protodesilylation in a one pot manner. In this way, **34** was obtained in 69% yield with 84% *ee*. Altogether, the synthesis of (−)-ranirestat involved 8 steps and took place in 14% overall yield with minimal use of chromatographic purifications.

**Scheme 164:**
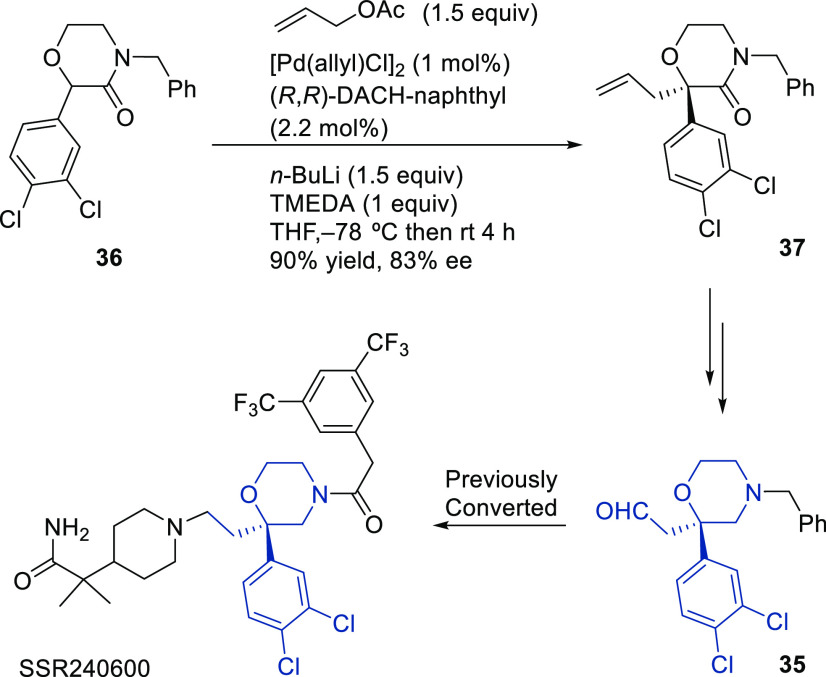
Synthesis of 2-Aryl-2-formylmethylmorpholine **35**, A Key Intermediate for the Preparation of SSR240600

Agbossou-Niedercom and co-workers developed an efficient route to optically enriched 2-aryl-2-formylmethylmorpholine **35**, a key intermediate for the preparation of potent neurokinin antagonists, such as SSR240600, featuring a quaternary carbon center (Scheme 164).^494^ For the creation of this stereocenter with the (*R*)-configuration, the authors used a Pd-catalyzed asymmetric allylic alkylation of **36** with allyl acetate using a (*R*,*R*)-DACH-naphthyl complex as catalyst. In this manner, the allylated product **37** was obtained in 90% yield with 83% *ee*. From this intermediate, **35** was prepared in 63% overall yield by amide reduction with LiAlH_4_ followed by ozonolysis/oxidation of the terminal double bond.

**Scheme 165:**
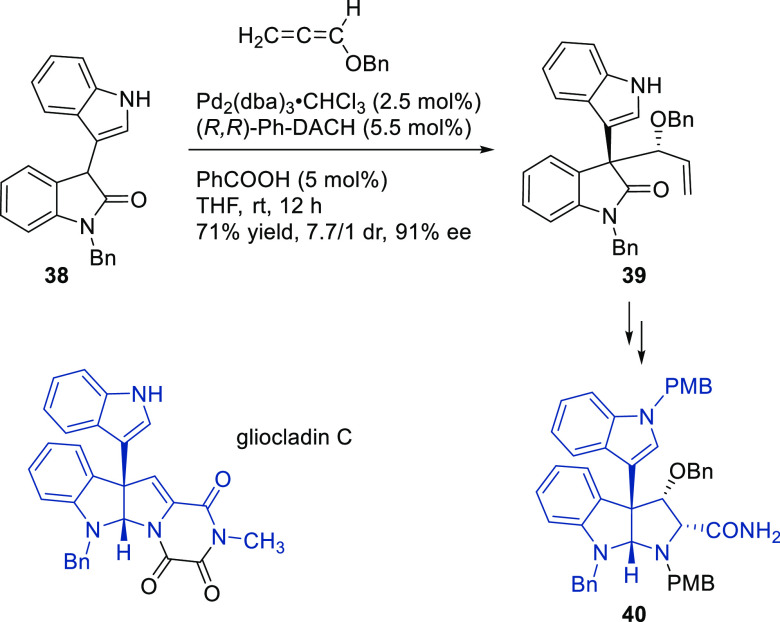
Synthesis of Gliocladin Indole Alkaloids

Trost and co-workers also developed an intriguing atom economical Pd-catalyzed allene hydrocarbonation reaction (i.e., the addition of a C–H compound across one of the C=C bonds of the allene) using oxindoles like **38** as nucleophiles.^424^ This protocol allows the formation of formal AAA reaction products (like **39**) without the need for allyl equivalents bearing activated leaving groups, and leads to branched products with high regioselectivity. Oxindoles bearing one quaternary and one tertiary vicinal stereocenter are obtained in excellent yields, diastereoselectivities, and enantioselectivities. The potential of this method was demonstrated by conversion of the 3,3-disubstituted oxindole products (**39**), resulting from the hydrocarbonation reaction, into the pyrrolidinoindoline core (**40**) of the gliocladin indole alkaloids in a concise and efficient manner (Scheme 165).

**Scheme 166:**
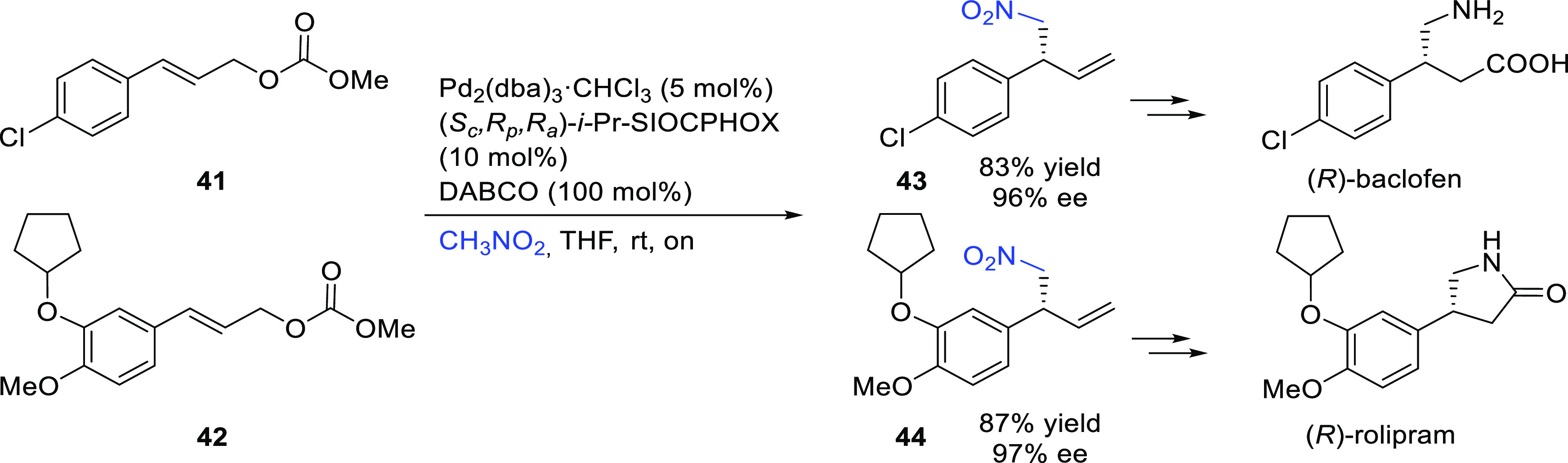
Synthesis of (*R*)-Baclofen and (*R*)-Rolipram

Hou and co-workers used modular, ferrocene-derived chiral P,N-ligands like (*S*_c_,*R*_p_,*R*_a_)-*i*-Pr-SIOCPHOX to mediate the Pd-catalyzed asymmetric allylic alkylation of monosubstituted allylic substrates with nitromethane, a niche that had remained largely unexplored.^215^ Using DABCO as a base and Pd_2_(dba)_3_ as the Pd source, the reactions took place with high regioselectivity in favor of the branched isomers, which were obtained with high *ee*. Starting from cinnamyl carbonates **41** and **42**, the corresponding allylated nitro compounds **43** and **44** were obtained in high yields (83 and 87%, respectively) and excellent enantioselectivities (96 and 97% *ee*, respectively). These products were converted into (*R*)-baclofen, an antispasmodic agent, and (*R*)-rolipram, an antiinflammatory and antidepressant drug, through straightforward procedures (Scheme 166).

**Scheme 167:**
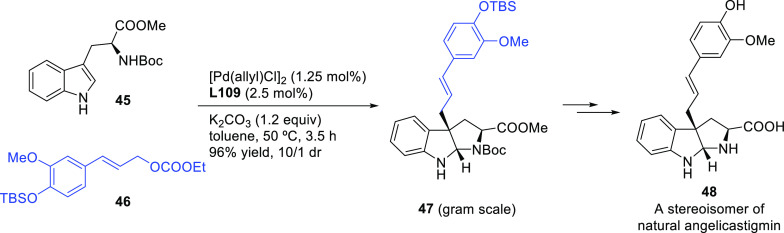
Synthesis of Angelicastigmin **48**

Liu and Du studied the Pd-catalyzed asymmetric allylic alkylation of 3-substituted indoles using BINOL-derived phosphoramidite ligands, in which the nitrogen atom bears a chiral, enantiopure allyl substituent (P/olefin ligands).^331^ Among various phosphoramidites, they identified **L109** (Figure 9) as the most effective ligand. This alkylation reaction was successfully used for the preparation of a variety of indolenines containing a quaternary carbon stereocenter in high yields with up to 87% *ee*. As an application of this approach, the development of a total synthesis of angelicastigmin, an alkaloid isolated from the root of *Angelica polymorpha* maxim, was attempted (Scheme 167). Thus, the asymmetric allylic alkylation of the enantiopure tryptophan derivative **45** with allyl carbonate **46** led in excellent yield (96%) and high diastereoselectivity (10/1 dr) to **47**, already containing the skeleton of angelicastigmin, in a process that can be operated at the gram scale. Sequential deprotection of the NBoc, OTBS, and methyl ester groups led to **48** that turned out to be a diastereomer of the natural product. Interestingly, the use of *ent*-**L109** in the alkylation reaction also led to a stereoisomer of natural angelicastigmin.

**Scheme 168:**
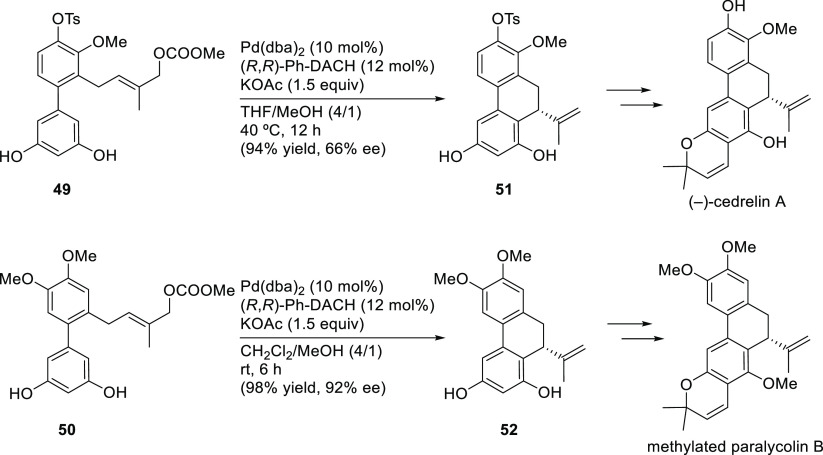
Synthesis of Cedrelin A and Methylated Paralycolin B

Cedrelins and paralycolins are highly oxygenated 9,10-dihydrophenanthrenes isolated from the bark of *Cedrelinga catenaeformis* and from the roots of *Clusia paralycola*, respectively. These substances exhibit cytotoxicity against KB and P388 cells (paralycolins) and bacteria such as *Staphylococcus aureus* and *Bacillus subtilis* (cedrelins), being thus interesting synthetic targets. Hamada and co-workers^441^ developed the first enantioselective total syntheses of cedrelin A and methylated paralycolin B (the isolated form of paralycolin B), employing a Pd-catalyzed asymmetric intramolecular Friedel–Crafts-type allylic alkylation of phenol precursors **49** and **50** as the key step (Scheme 168). Using the Trost ligand (*R*,*R*)-Ph-DACH and Pd(dba)_2_ as the Pd source, cyclization of **49** took place in high yield (94%), but only moderate enantioselectivity (66% *ee*) to afford **51**, which was converted to cedrelin A by completing a 12-step synthesis with 16.5% overall yield. The cyclization of **50**, in turn, proceeded under milder conditions and with very high enantioselectivity (92% *ee*) to afford **52** in 98% yield. Formation of the chromene unit and subsequent methylation completed the synthesis of methylated paralycolin B (10 steps, 32.5% overall yield).

**Scheme 169:**
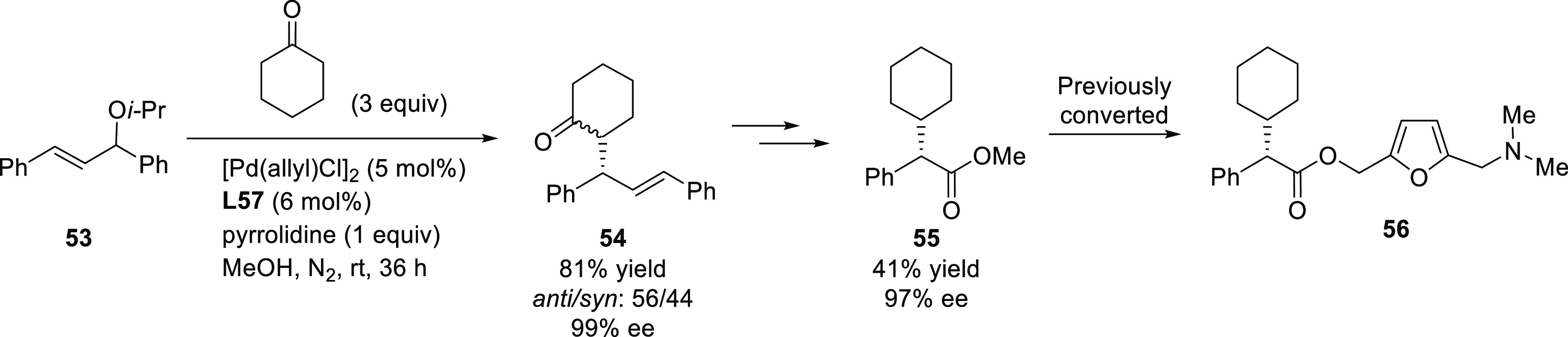
Synthesis of Antimuscarinic Agent **56**

Liu, Zhang, and co-workers developed a hydrogen bond-induced, Pd-catalyzed allylic alkylation of carbonyl compounds (mostly cyclic ketones) with simple alkyl allyl ethers.^383^ In this procedure, methanol, the reaction solvent, activates the initial allyl system through hydrogen bonding, likely assisting the generation of the Pd η^3^-allyl intermediate. The reaction was mainly developed in the racemic series, with dppf as the ligand of choice. An enantioselective version of the reaction was also described using the enantiopure dppf analog **L57** (Scheme 65) as a ligand. Starting from the allyl isopropyl ether **53**, reaction with cylohexanone led to the AAA product **54** with poor diastereoselectivity but excellent enantioselectivity. From this intermediate, ester **55** was prepared in a diastereoconvergent manner in 41% yield and 97% *ee*.^495^ This ester had been previously converted to the selective antimuscarinic agent **56** (Scheme 169).

**Scheme 170:**
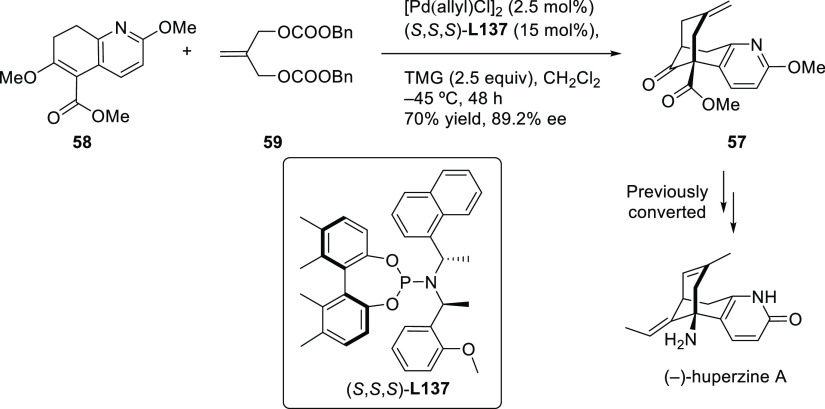
Synthesis of (−)-Huperzine A

Ojima and co-workers explored the use of a modular family of bisphenol-derived monodentate phosphoramidite (MPN) ligands in a Pd-catalyzed tandem asymmetric allylic alkylation devised for the preparation of **57**, a critical key intermediate in a formal total synthesis of (−)-huperzine A (Scheme 170).^496^ This is a sesquiterpene alkaloid isolated from *Lycopodium serratum*, which was identified as a selective and potent inhibitor of acetylcholine esterase and received attention as a potential drug for the treatment of Alzheimer’s disease. As shown in Scheme 9, these authors identified (*S*,*S*,*S*)-**L137** as the optimal ligand for the preparation of **57** from dihydroquinoline **58** and bis(carbonate) **59**, allowing for the preparation of this intermediate in good yield (70%) with high enantiopurity (89.2% *ee*).

**Scheme 171:**
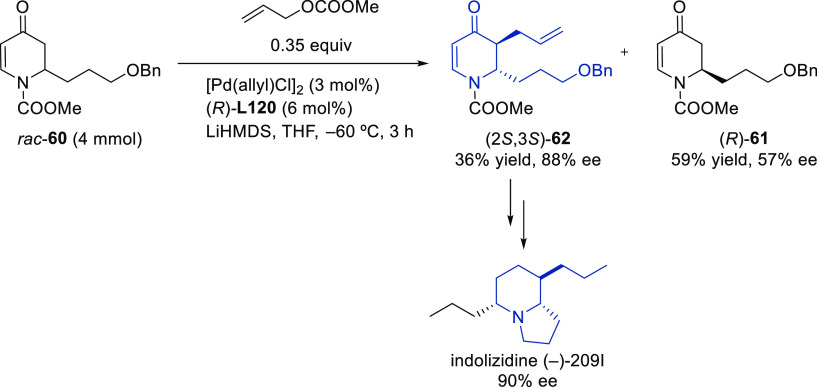
Synthesis of Indolizidine (−)-209I

Ding, Hou, and co-workers explored the use of a wide variety of ligands for the kinetic resolution of 2-substituted-dihydro-4-pyridones via Pd-catalyzed asymmetric allylic alkylation, finding that the P-PHOS ligand **L120** (Scheme 106) was optimal for this application.^377^ After a thorough optimization of reaction conditions, *rac*-**60** was submitted to the alkylation reaction with 0.35 eq of allyl methyl carbonate using the ligand **L120** (Scheme 171). In this way, (*R*)-**61** with 57% *ee* was recovered in 59% yield, together with the allylated dihydropyridone (2*S*,3*S*)-**62** (36% yield, 88% *ee*). This intermediate was used in a catalytic asymmetric total synthesis of indolizidine (−)-209I, an alkaloid found in the skin of poisonous frogs.

**Figure 22:**
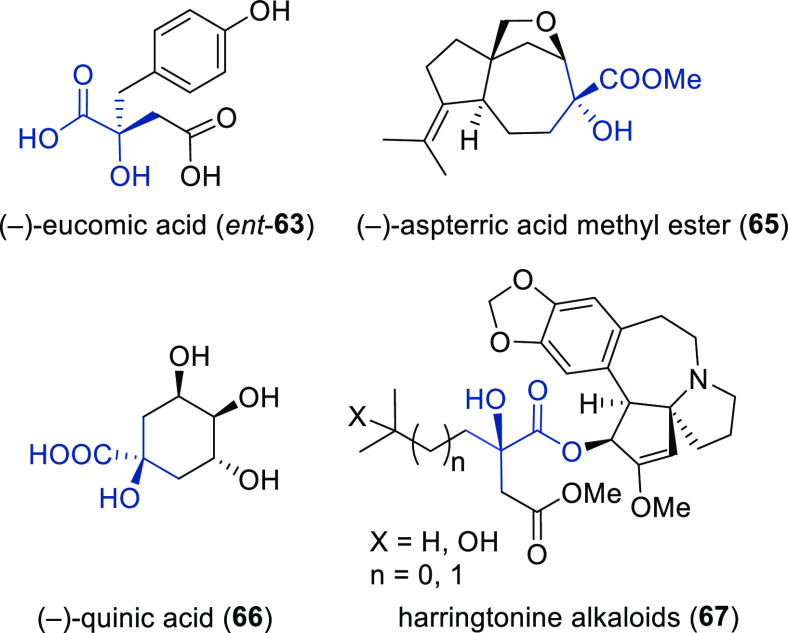
Natural products containing stereodefined tetrasubstituted α-oxycarbonyl moieties.

Stoltz and co-workers conceived a strategy for the catalytic enantioselective synthesis of (+)-eucomic acid (**63**) based on intermediate **64** as the carrier of the stereodefined tetrasubstituted α-hydroxyacid present in its structure.^497^ Naturally occurring (**−**)-eucomic acid (*ent*-**63**) is involved in cytochrome C oxidase activity and respiratory functions in human keratinocytes, being a potential component for protective skin antiaging therapies. The tetrasubstituted α-oxycarbonyl moiety designed as the key intermediate in the Stoltz synthesis is also present in other important natural products, such as (−)-aspterric acid methyl ester (**65**), quinic acid (**66**), and the harringtonine alkaloids (**67**), thus adding interest to the catalytic enantioselective preparation of this motif (Figure 22).

**Scheme 172:**
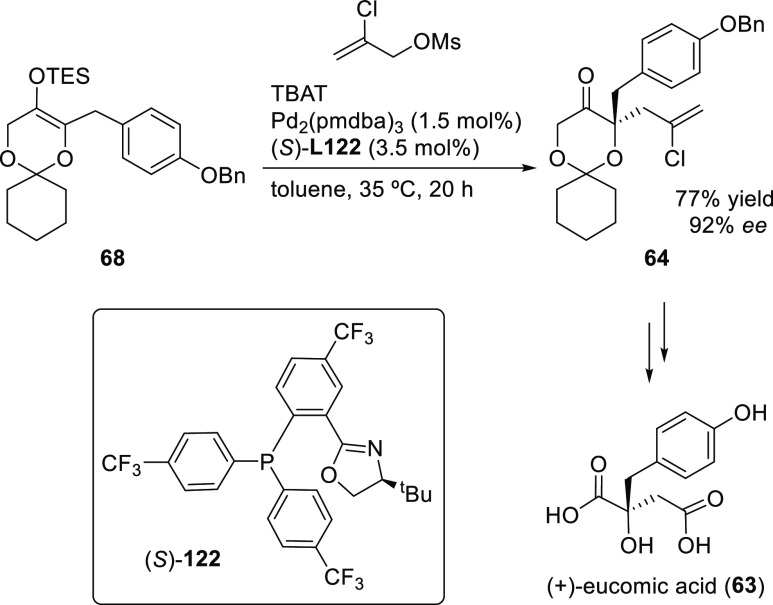
Synthesis of (+)-Eucomic Acid (**63**)

For the preparation of intermediate **64**, a Pd-catalyzed AAA reaction of **68** with 2-chloroallyl mesylate using PHOX ligand (*S*)-**L122** was envisaged (Scheme 172). Through this procedure, **64** was prepared in 77% yield with 92% *ee*. The conversion of this intermediate into the target (+)-eucomic acid (**63**) took place uneventfully. In this manner, the first enantioselective total synthesis of the unnatural enantiomer of eucomic acid was completed in a longest linear sequence of 13 steps.

**Scheme 173:**
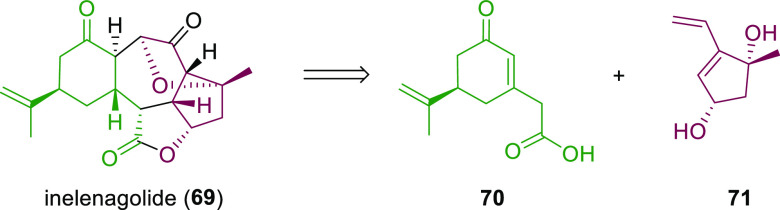
Synthesis of Ineleganolide (**69**)

Stoltz and co-workers developed an enantioselective, convergent approach to the tetracyclic core of the norditerpenoid ineleganolide (**69**), a representative example of the norcembranoids.^498^ In a retrosynthetic analysis sense, they disconnected the molecule into two main enantiopure fragments: carboxylic acid **70** and diol **71** (Scheme 173).

**Scheme 174:**
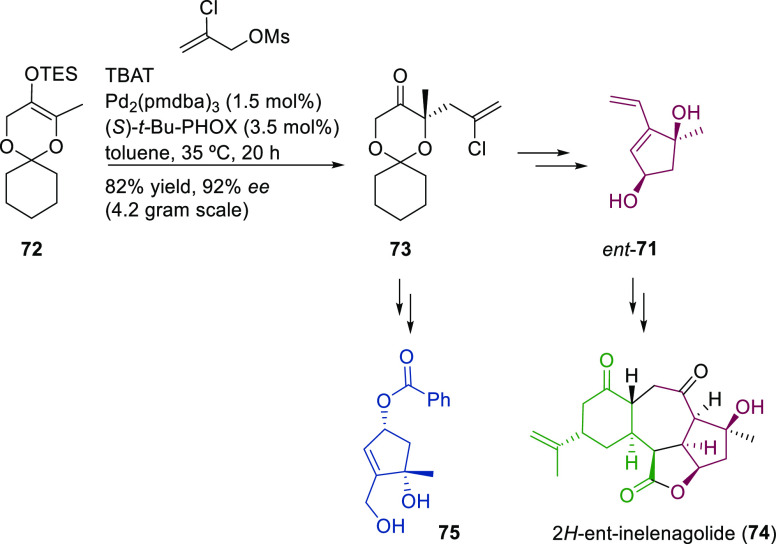
Synthesis of Key Intermediates *ent*-**71** and **75** and Natural Product 2*H*-*ent*-Inelenagolide (**74**)

The preparation of this diol (Scheme 174) was envisaged through a Pd-catalyzed enantioselective allylic alkylation of silyl enol ether **72** affording ketone **73** in 82% yield and 92% *ee* in a process operated at multigram scale. When the more readily available, less expensive (*S*)-*t*-Bu-PHOX ligand was used, ketone **73** was obtained in the (*S*)-configuration. This ketone was stereoselectively transformed into the diol *ent*-**71**, which was used as a building block for norcembranoids in the non-natural enantiomeric series. Development of the original synthetic plan starting from *ent*-**70** and *ent*-**71** ultimately led to 2*H*-ent-inelenagolide (**74**), whose final conversion into the enantiomer of the natural product proved problematic. It is worth mentioning that in a previous effort,^387^ the same group converted ketone **73** into diol **75**, a potential alternative building block for the preparation of norcembranoids in the non-natural enantiomeric series.

**Figure 23:**
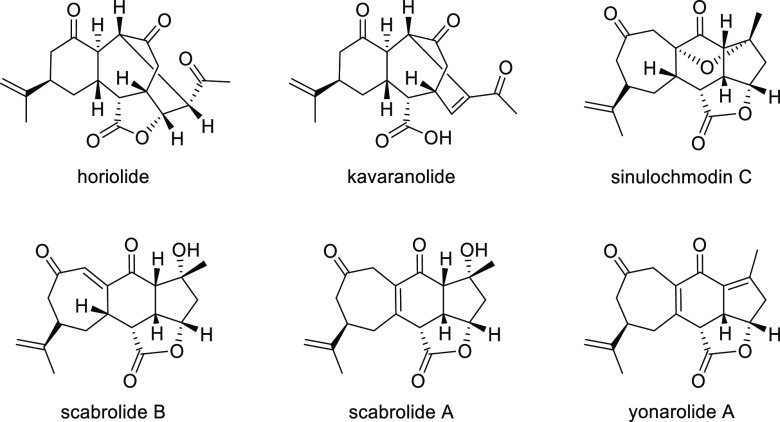
Furanebutenolide-derived polycyclic norcembranoid diterpenes prepared from diol *ent*-**71**.

Subsequently, the synthetic strategy designed to convergently build the [6,7,5,5] tetracyclic core of ineleganolide (**69**) was extended to provide divergent access to the isomerized carbon skeletons of horiolide, kavaranolide, sinulochmodin C, scabrolide B, scabrolide A, and yonarolide (Figure 23).^499^

**Scheme 175:**
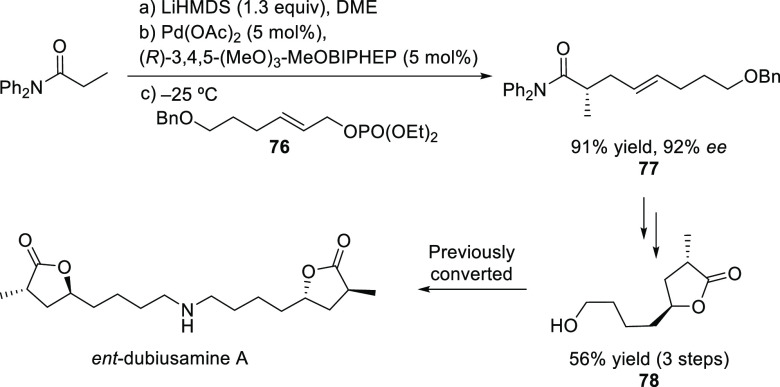
Synthesis of Dubiusamine A

Hou and co-workers explored the Pd-catalyzed asymmetric allylic alkylation of alkyl-substituted allyl esters with enolates of *N*,*N*-diphenylamides.^413^ Axially chiral, biaryl-derived diphosphines like (*R*)-3,4,5-(MeO)_3_-MeOBIPHEP were found to be optimal ligands for this process. This reaction, which represented a novel combination of alkyl-substituted allyl systems with a class of poorly stabilized carbon nucleophiles, exhibited broad applicability and high regioselectivity in favor of the linear product, as well as high yield and enantioselectivity. When the reaction was performed with *N*,*N*-diphenylpropionamide and allyl phosphate **76**, using Pd(OAc)_2_ as the Pd source (Scheme 175), the alkylation product **77** was obtained in 91% yield with 92% *ee*. This intermediate could be transformed in 56% yield and without erosion in enantiomeric purity into lactone **78** through a three-step sequence. This lactone (with the opposite configuration) had been previously converted to the natural product dubiusamine A.

**Scheme 176:**
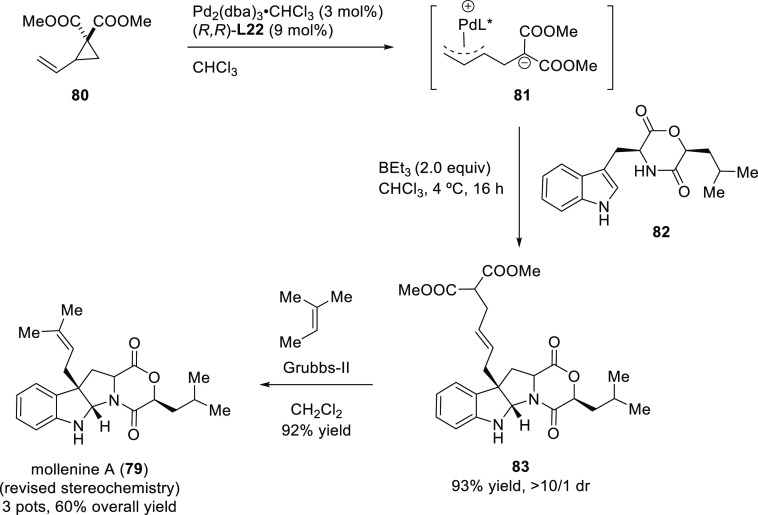
Synthesis of Alkaloid Mollenine A (**79**)

Very recently, Trost and co-workers reported for the first time the use of vinylcyclopropanes as electrophiles in the Pd-catalyzed asymmetric allylic alkylation of C3-substituted 1*H*-indoles and tryptophan derivatives.^96^ A broad range of 3,3-disubstituted indolenines and indolines were prepared in up to gram amounts by this method in a highly regio- and stereocontrolled manner. Starting from enantiopure tryptophan derivatives, the stereochemical outcome of the Pd-catalyzed AAA reactions is controlled by the chiral ligands employed, allowing for the development of an efficient synthesis of alkaloid mollenine A (**79**), as shown in Scheme 176. Thus, vinylcyclopropane **80** was used as starting material to generate the zwitterionic Pd η^3^-allyl complex **81** using the chloroform complex of Pd_2_(dba)_3_ as the Pd source and the stilbene-derived Trost ligand (*R*,*R*)-**L22** (Scheme 19). Trapping of this complex with the l-tryptophan-derived indole **82** led to the tetracyclic advanced intermediate **83** with >10/1 dr and 93% yield. A final cross metathesis with 2-methyl-2-butene using the Grubbs II catalyst led to mollenine A (**79**) in 92% yield. Remarkably, this approach involves only three reaction stages (from the tryptophan precursor of **82**) and takes place with 60% overall yield.

**Scheme 177:**
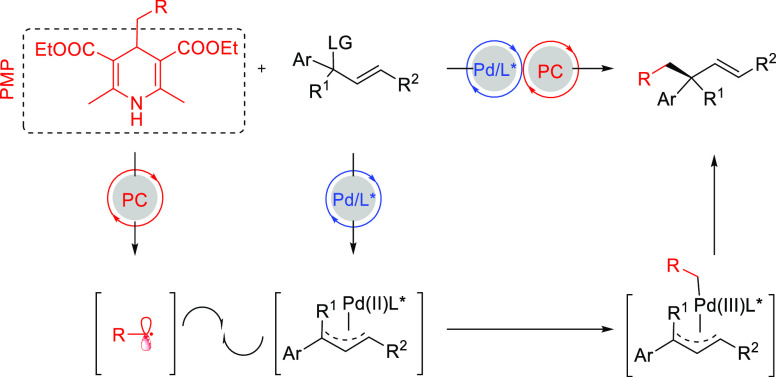
Merger of Photoredox and Pd-Catalysis for the Allylic Alkylation Reaction

Yu and co-workers reported the highly regio- and enantioselective allylic alkylation with mere alkyl nucleophiles by the merger of photoredox and palladium catalysis.^437^ In this dual catalytic process, alkyl radicals generated from 4-alkyl-1,4-dihydropyridines (Hantzsch esters) act as coupling partners of in situ generated Pd η^3^-allyl complexes (Scheme 177). Noteworthy, this mechanistically novel strategy expands the scope of the traditional Pd-catalyzed asymmetric allylic alkylation reaction to alkyl groups derived from nonacidic precursors.

**Scheme 178:**
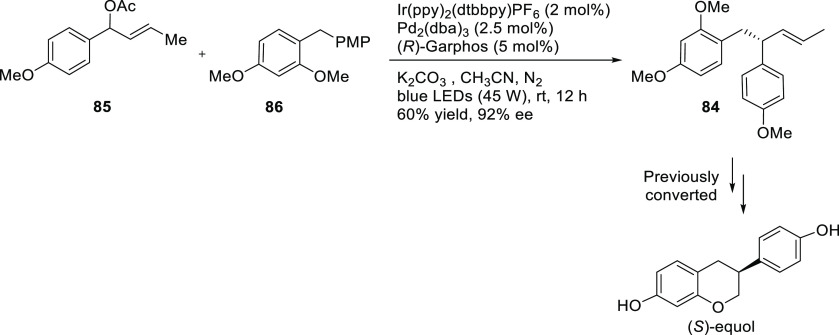
Synthesis of (*S*)-Equol

The dual-catalyzed approach proved to be very general, enabling the reaction of a variety of allyl esters with 4-alkyl substituted Hantzsch esters. As an illustration of its potential, the key intermediate **84** for the enantioselective synthesis of (*S*)-equol, a natural estrogenic metabolite, could be prepared in good yield (60%) through a highly regioselective (branched/linear: 91/9) and enantioselective (92% *ee*) reaction of allyl acetate **85** with Hantzsch ester **86** using [Ir(ppy)_2_(dtbbpy)]PF_6_ as the photocatalyst and (*R*)-Garphos as the ligand for Pd (Scheme 178).^437^

**Scheme 179:**
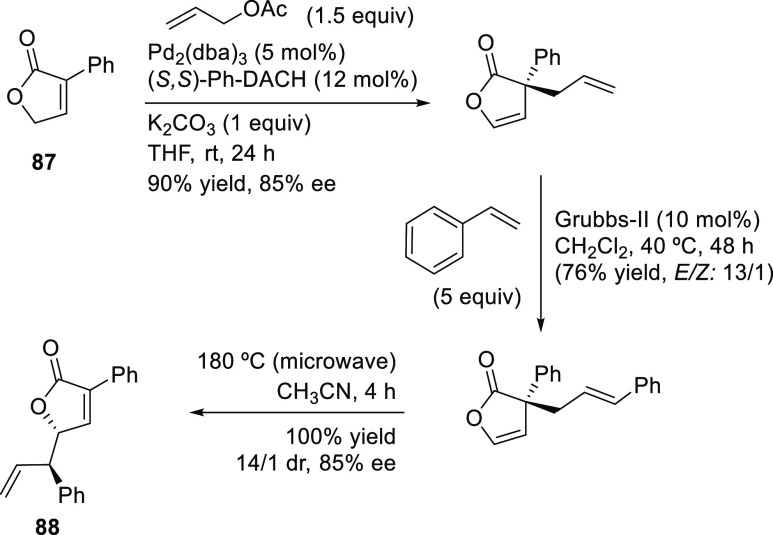
Synthesis of γ-Butenolides

Arseniyadis and co-workers developed a clever combination of reactions involving sequential Pd-catalyzed asymmetric allylic alkylation, (*E*)-selective cross-metathesis and [3,3]-sigmatropic Cope rearrangement for the synthesis of γ-butenolides bearing two vicinal stereogenic centers, a structural motif found in many natural products.^400^ The application of this methodology, starting from readily available α-substituted (5*H*)-furan-2-ones **87** to a representative example (**88**) is illustrated in Scheme 179. Noteworthy, the highly enantio- and diastereoselective combination of Pd-catalyzed asymmetric allylic alkylation and cross-metathesis can be applied to the synthesis of spirocyclic frameworks starting from α-styryl substituted (5*H*)-furan-2-ones.

**Scheme 180:**
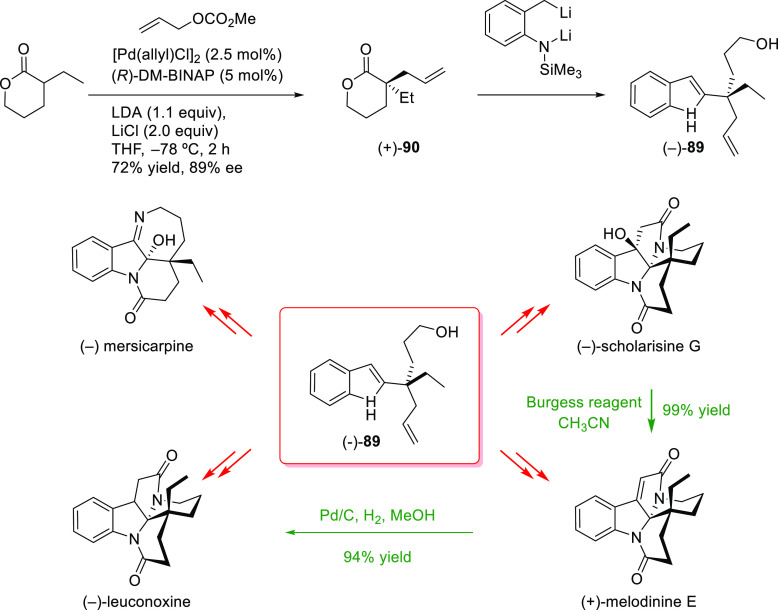
Synthesis of the Key Intermediate (−)-**89**

Liu and Wang developed a unified strategy for the enantioselective syntheses of the aspidosperma-derived monoterpene indole alkaloids (−)-scholarisine G, (+)-melodinine E, (−)-leuconoxine, and (−)-mersicarpine from a common 2-alkylated indole intermediate bearing an all-carbon quaternary stereogenic center (−)-**89** (Scheme 180).^401^ The preparation of this key intermediate was achieved via the Smith modification of the Madelung indole synthesis^500^ that allowed for the straightforward coupling of lactone (+)-**90**, prepared by Pd-catalyzed asymmetric allylic alkylation, with *o*-toluidine. The target alkaloids could be then prepared from (−)-**89** through highly efficient, protecting group-free reaction sequences.

**Scheme 181:**
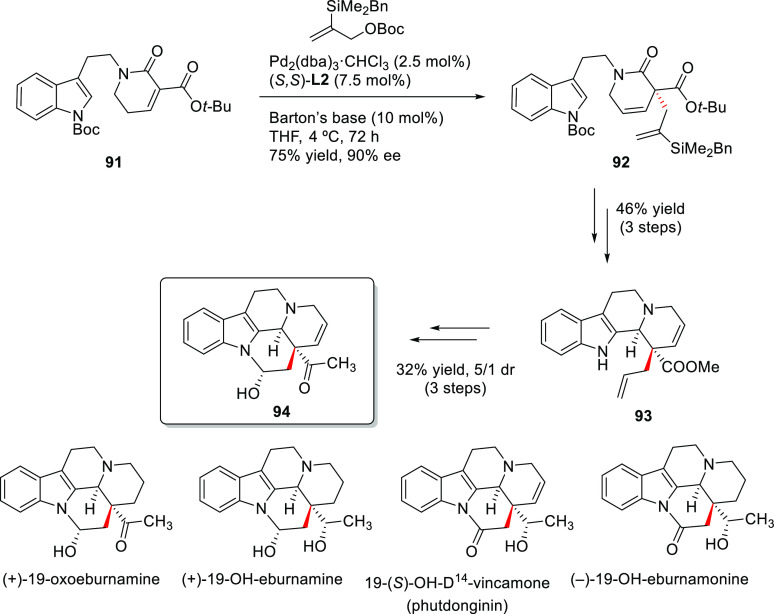
Synthesis of the Key Intermediate **94**

Eburnane indole alkaloids are a family of structurally diverse natural products mainly isolated from the plants of the genus *Kopsia*, which show potent bioactivity on the cardiovascular system and brain functions. Likewise, some members of this family of compounds oxidized at C19 possess favorable antitumor activity. Trost and co-workers recently reported a divergent enantioselective approach toward some representative examples of this family of alkaloids using a Pd-catalyzed asymmetric allylic alkylation of an *N*-alkyl-α,β-unsaturated lactam (**91**) to create the key stereocenter, ultimately controlling the configuration of all stereogenic centers in these structures.^95^ Thus, reaction with the silyl-substituted allyl carbonate catalyzed by Pd/(*S*,*S*)-**L2** (Scheme 3) delivered **92** in 75% yield with 90% *ee* (Scheme 181). A short sequence involving a Bischler-Napieralski cyclization as the key step afforded the tetracyclic intermediate **93** in 46% overall yield, and a final sequence involving a completely chemoselective conversion of the methyl ester into a methyl ketone with MeLi, dihydroxylation of the terminal olefin with OsO_4_/NMO and oxidative cleavage, produced an aldehyde which spontaneously cyclized into the pentacyclic key intermediate **94**, obtained as a 5:1 mixture of diastereomers in 32% yield. From **94**, (+)-19-oxoeburnamine, (+)-19-OH-eburnamine, 19-(*S*)-OH-D14-vincamone (phutdonginin), and (−)-19-OH-eburnamonine were accessible.

#### 2.5.2 Nitrogen Nucleophiles

**Scheme 182:**
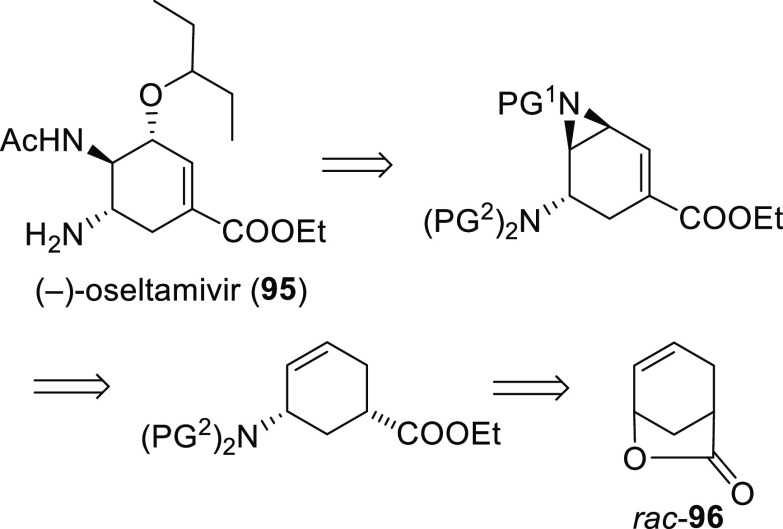
Retrosynthetic Analysis of (−)-Oseltamivir **95**

**Scheme 183:**
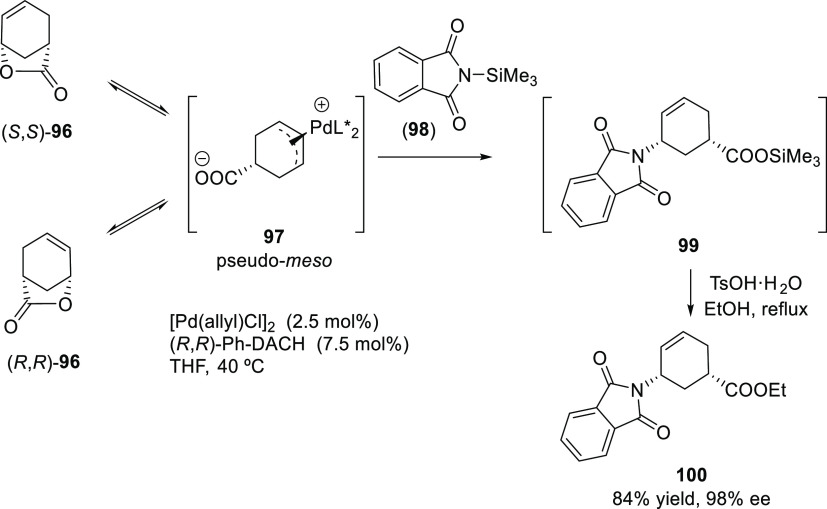
Synthesis of the Key Intermediate **100** for the Preparation of (−)-Oseltamivir

Oseltamivir (Tamiflu) (**95**), a drug used for the prevention and treatment of influenza caused by A and B-type viruses, became the object of intense synthetic efforts in 2005–2006, when it was widely used during the H5N1 avian influenza epidemic in Southeast Asia. In this context, Trost and Zhang reported in 2008 a concise enantioselective synthesis of (−)-**95**.^501,502^ The key step in the retrosynthetic analysis (Scheme 182) was a then novel Pd-catalyzed asymmetric allylic substitution with a nitrogen-centered nucleophile, opening the *cis*-lactone ring of racemic **96** and setting the requisite stereochemical course for the entire synthesis. This reaction takes place through a pseudo-*meso-*Pd allyl intermediate (**97**, Scheme 183) and leads to the deracemization of **96**.

In practice, to overcome the repulsion between the negatively charged nitrogen nucleophile and the carboxylate leaving group that could inhibit the nucleophilic addition, a strategy was devised that allows for the trapping of the carboxylate anion as its TMS ester by silyl transfer from trimethylsilylphthalimide (**98**). Silyl transfer, which is driven by the oxophilicity of silicon, simultaneously generates the required nucleophilic phthalimide anion. (Scheme 183). The reaction was performed with [(η^3^-C_3_H_5_)PdCl]_2_ as the palladium source and the Trost ligand (*R*,*R*)-Ph-DACH. Trapping of the intermediate Pd η^3^-allyl complex **97** by **98** led to the trimethylsilyl ester **99**, which was converted into ethyl ester **100** by a one pot procedure (84% yield, 98% *ee*). The conversion of **100** into (−)-**95** was completed in a straightforward manner through a sequence involving as its main steps the introduction of a double bond conjugated to the ester group, a regio- and stereoselective Rh-catalyzed aziridination taking place at the distal double bond, and the regioselective ring-opening of the aziridine with 3-pentanol. Altogether, the synthesis involved 8 steps with a 30% overall yield.

**Scheme 184:**
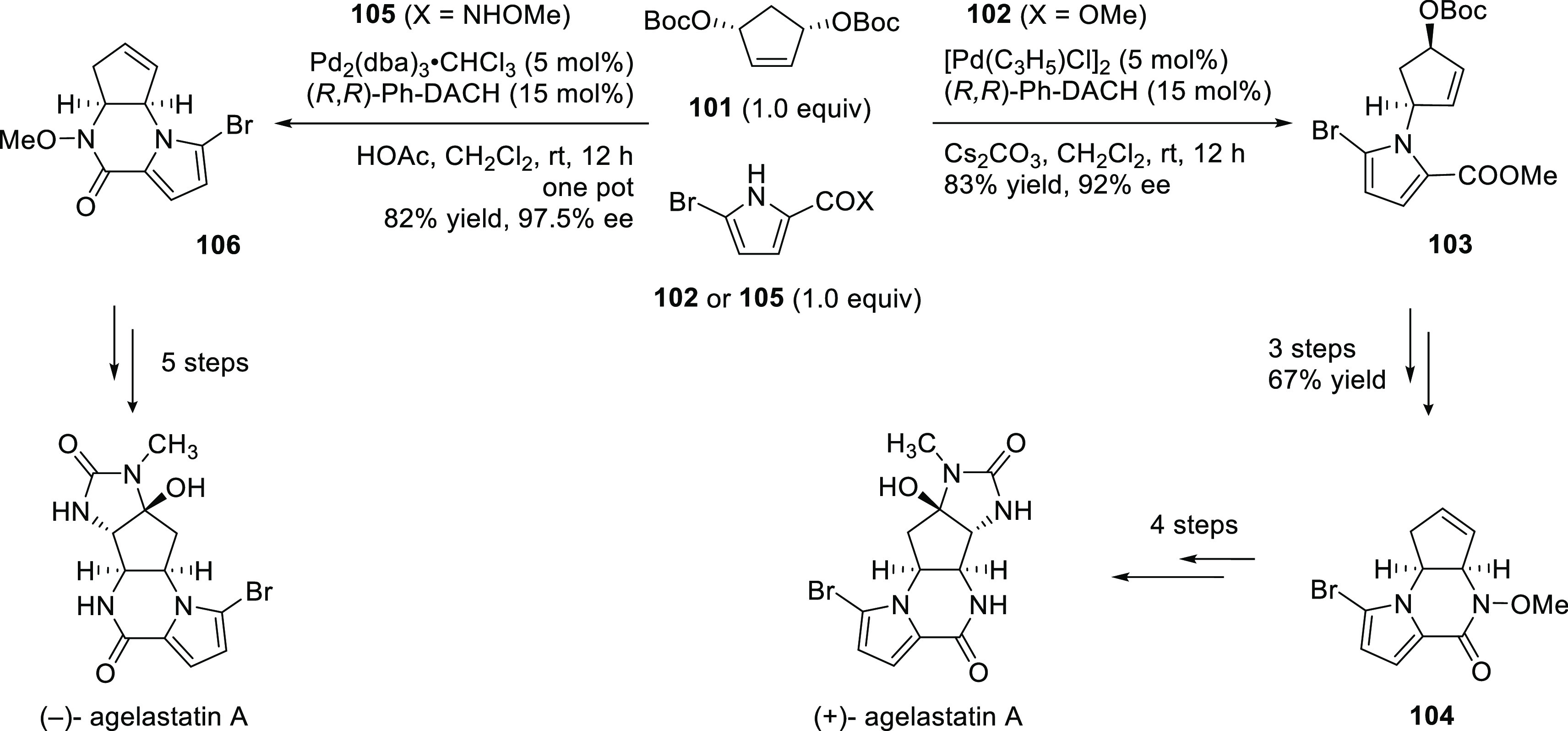
Synthesis of (+)-Agelastatin and (−)-Agelastatin

In 2009, Trost and Dong introduced the use of two new classes of nucleophiles, pyrroles and *N*-alkoxyamides, in Pd-catalyzed asymmetric allylic amination reactions.^503^ Starting from the *meso*-diol derivative **101**, the reactive position of the pyrrole nucleophile could be efficiently controlled by simple selection of the functional group (a methoxycarbonyl or a *N*-methoxycarboxamido group) at the 2-position of this species (Scheme 184). With the methoxycarbonyl derivative (**102**), the N-alkylated pyrrole **103** was obtained in 83% yield and 92% *ee*, and this intermediate could be converted to the pyrrolopiperazinone **104** in 67% yield through a three-step sequence. On the other hand, the *N*-methoxycarboxamido derivative (**105**) led, upon asymmetric cascade allylic amination, to the regiosomeric pyrrolopiperazinone **106** (82% yield, 97.5% *ee*). The tricyclic derivatives **104** and **105** were then converted into (+)-agelastatin A and (−)-agelastatin A, respectively. Interestingly, the same ligand with the same configuration (*R,R*)-Ph-DACH could be used for the preparation of both enantiomers of this marine alkaloid by selecting the appropriate activated pyrrole derivative used in the asymmetric allylic amination step.

**Scheme 185:**
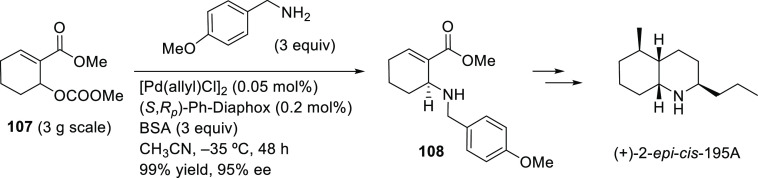
Synthesis of (+)-2-*epi*-*cis*-**195A**, the C2 Epimer of Pumiliotoxin C

In 2011, Hamada and co-workers reported a new procedure for the enantioselective synthesis of 2-substituted hexahydroquinolin-4-ones ultimately relying on a Pd-catalyzed asymmetric allylic amination using a chiral diaminophosphine oxide ((*S*,*R*_p_)-Ph-Diaphox) as a preligand.^504^ These pentavalent phosphorus compounds are activated in situ by *N*,*O*-bis(trimethylsilyl)acetamide (BSA), which induces tautomerization toward trivalent phosphorus compounds that are the actual ligands for Pd. As illustrated in Scheme 185, the asymmetric allylic amination of **107** with *p*-methoxybenzylamine under these conditions, a process that could be operated at the multigram scale, afforded the chiral amine **108** in excellent yield (99%) with high enantioselectivity (95% *ee*). In combination with a subsequent diastereoselective intramolecular Mannich reaction, this process allowed for the development of a catalytic asymmetric synthesis of (+)-2-*epi*-*cis*-195A, the C2 epimer of pumiliotoxin C.

**Scheme 186:**
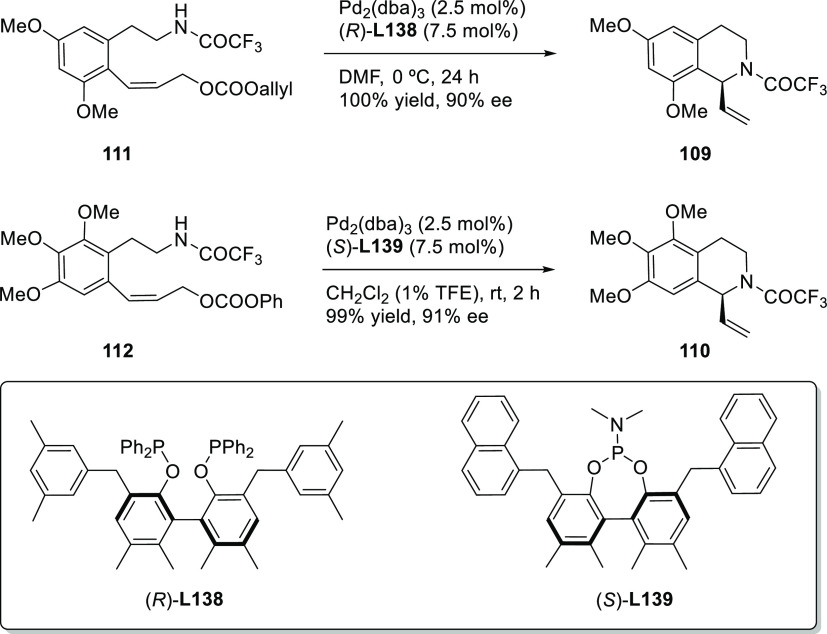
Synthesis of 1-Vinyltetrahydroisoquinolines **109** and **110**

1-Vinyltetrahydroisoquinolines are versatile intermediates for the preparation of naturally occurring isoquinoline alkaloids and thus attract much synthetic interest. Ojima and co-workers developed an efficient approach toward two representative molecules of this family, 1-vinyl-6,8-dimethoxytetrahydroisoquinoline (**109**) and 1-vinyl-5,6,7-trimethoxytetrahydroisoquinoline (**110**), suitable for the preparation of the Schulzeine alkaloids, and (−)-*O*-methylthaicanine or isopyruthaline, respectively (Scheme 186).^505^ Starting from allyl carbonates **111** and **112**, and using Pd-catalyzed intramolecular asymmetric allylic amination reactions mediated by the biaryl ligands developed in the Ojima laboratory, BOP-Lg (**L138**) and MPN-Lj (**L139**), tetrahydroisoquinolines **109** and **110** were obtained in high yield with >90% *ee*.

**Scheme 187:**
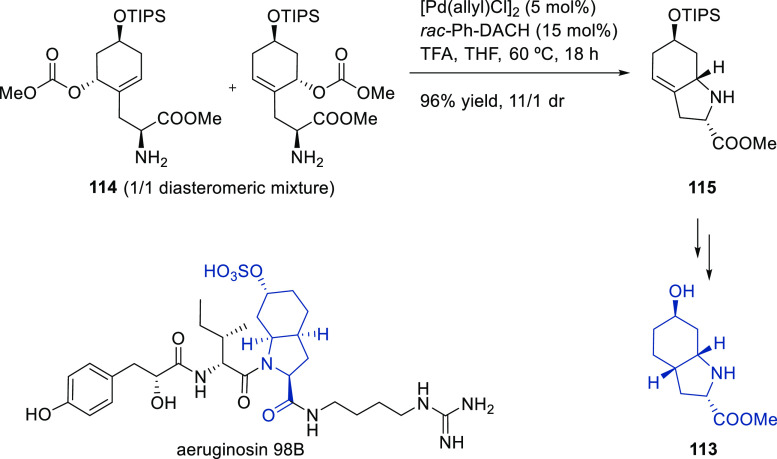
Synthesis of the Key Intermediate **113**

In 2012, Trost and co-workers completed the first total synthesis of aeruginosin 98B in eight steps through the convergent integration of four fragments.^506^ One of these fragments was the bicyclic proline analogue **113** (highlighted in blue in Scheme 187). For its preparation, a Pd-catalyzed intramolecular AAA reaction of a 1/1 diastereomeric mixture of the enantiopure allyl carbonates **114** [the amino acid side chain in these molecules derives from 3-iodo-(*S*)-alanine] in the presence of the Trost Ph-DACH ligand in racemic form was used. The cyclization process provided hexahydroindole derivative **115** with high diastereoselectivity and enantiopurity. Interestingly, the use in this reaction of *rac*-Ph-DACH leads to much higher yield and diastereoselectivity than either of the enantiomeric forms of the ligand, thus suggesting that each enantiomer of the racemic ligand has a clear preference for one of the diastereomers of **114**. This illustrates the importance of the kinetic prevalence of matched substrate/catalyst combinations over the corresponding mismatched ones for the achievement of diastereoconvergence.

**Scheme 188:**
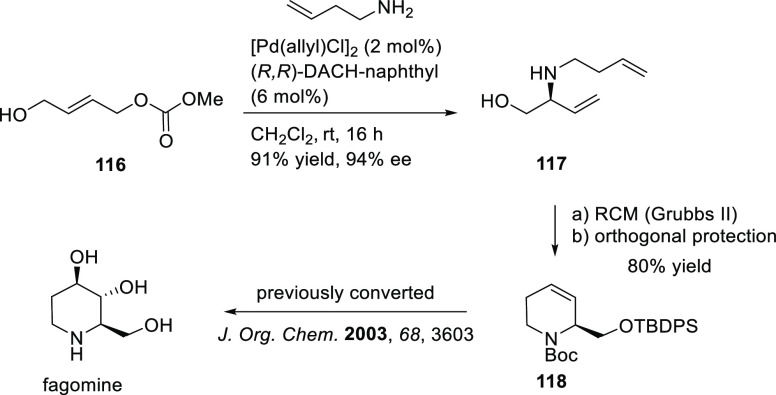
Synthesis of Fagomine

Castillón, Díaz, and co-workers studied the Pd-catalyzed asymmetric allylic amination of carbonate **116** with homoallylamine using the Trost DACH-naphthyl ligand (Scheme 188).^472^ The process turned out to be highly regioselective in favor of the branched product, and this was attributed to hydrogen bonding interactions between the hydroxy group in the substrate and the ligand in the transition state leading to the branched product. The resulting amino alcohol (**117**) was obtained in high yield (91%) and with very high enantioselectivity (94% *ee*). Subsequent ring closing metathesis with Grubbs II catalyst (92% yield) and orthogonal protection of the amino and hydroxy groups (87% yield) led to **118**, thus completing a short formal enantioselective synthesis of fagomine, a glucosidase inhibitor.

**Scheme 189:**
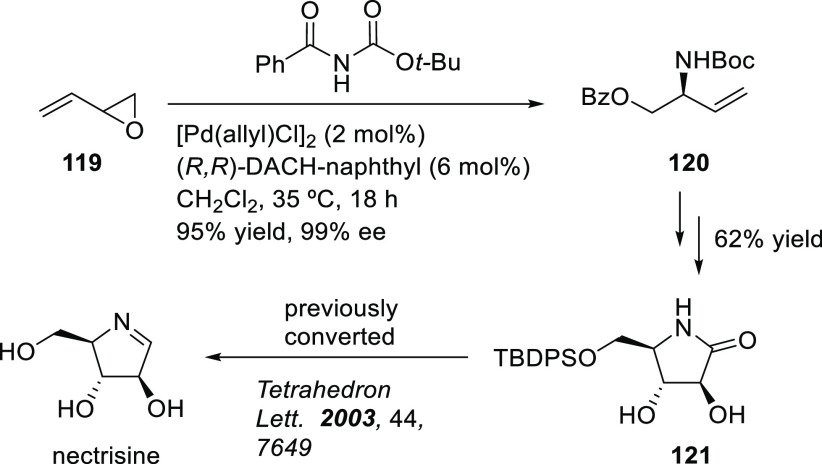
Synthesis of Nectrisine

In 2016, the same group reported the first enantioselective formal synthesis of the glucosidase inhibitor nectrisine in 7 steps and 48% overall yield from the commercially available racemic butadiene monoepoxide (**119**).^507^ A Pd-catalyzed dynamic kinetic asymmetric transformation (DYKAT) with the (*R*,*R*)-DACH-naphthyl ligand was used to convert the racemic monoepoxide into the protected amino alcohol **120** in 95% yield and 99% *ee*. From this intermediate, the advanced precursor **121** was obtained in 62% yield through a sequence involving cross metathesis with ethyl acrylate and dihydroxylation as the key steps (Scheme 189).

**Scheme 190:**
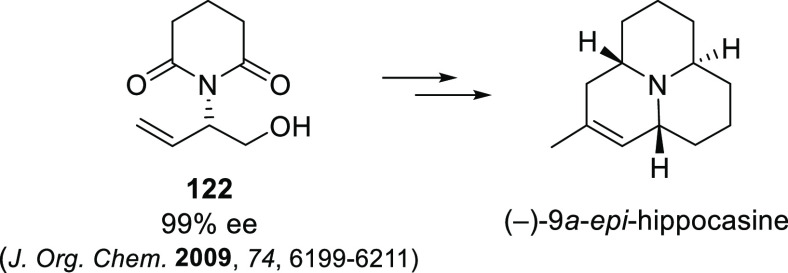
Synthesis of (−)-9*a*-*epi*-Hippocasine

Bayón, Figueredo, and co-workers used enantiopure **122** to develop a new strategy for the stereoselective synthesis of perhydro-9*b*-azaphenalene alkaloids.^508^ The starting material in this approach (**122**) is available in high yield and enantiomeric purity by Pd-catalyzed asymmetric allylic amination of butadiene monoepoxide (**119**) with glutarimide,^509^ and the authors successfully developed the first synthesis of (−)-9*a*-*epi*-hippocasine by forging the additional stereocenters in the molecule in an iterative manner from the chiral information contained in **122** (Scheme 190).

**Scheme 191:**
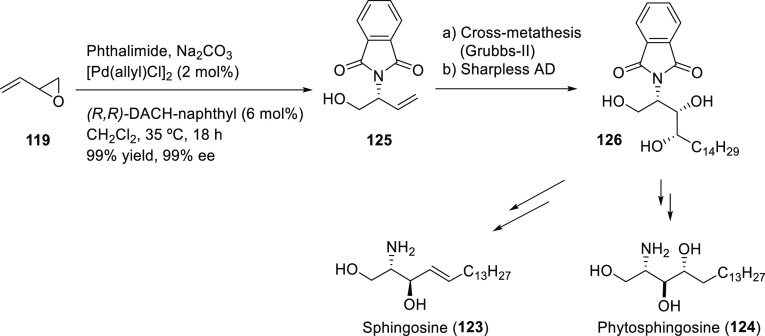
Synthesis of Sphingosine (**123**) and Phythosphingosine (**124**)

In 2009, Castillón, Matheu, and co-workers developed a straightforward procedure (Scheme 191) for the enantioselective synthesis of sphingosine (**123**) and phythosphingosine (**124**).^510^ The configuration of the carbon atom bearing the amino substituent in these compounds is established through a Pd-catalyzed DYKAT of racemic butadiene monoepoxide **119** with phthalimide using the Trost (*R*,*R*)-DACH-naphthyl ligand, as already discussed for similar cases. In this manner, the protected amino alcohol **125** was obtained in excellent yield with excellent enantioselectivity. A subsequent two-step sequence involving a cross-metathesis with the Grubbs II catalyst and a Sharpless asymmetric dihydroxylation produced the key intermediate **126** that was readily converted to the target compounds **123** and **124**.

**Figure 24:**
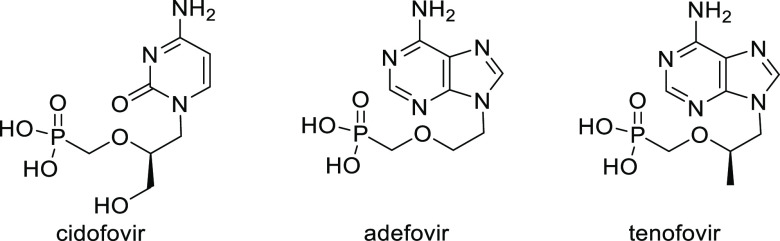
ANPs with antiviral activity.

A similar approach was very recently applied by the same authors to develop a short enantioselective synthesis of acyclic nucleoside phosphonates (ANPs).^108^ These substances are modified nucleosides, in which the sugar moiety has been replaced by a functionalized acyclic chain linking the nucleobase and the phosphonic acid moiety. They are of current interest for the antiviral activity displayed by some members of this family, such as cidofovir, adefovir, and tenofovir, that can be easily modified to allow oral administration (Figure 24).

**Scheme 192:**
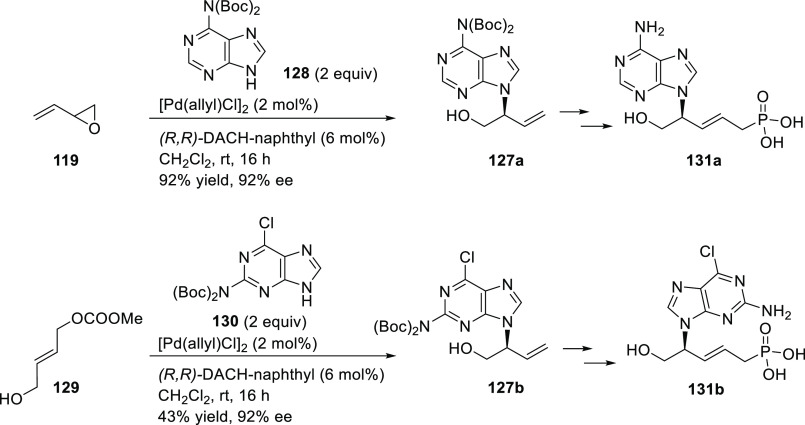
Synthesis of Target Acyclic Nucleoside Phosphonates **131a** and **131b**

For the synthesis of analogs of these substances, intermediates **127a**–**b** were prepared by reacting racemic butadiene monoepoxide (**119**) with protected adenine **128** or allyl carbonate **129** with the guanine derivative **130** in the presence of a Pd source and the Trost (*R*,*R*)-DACH-naphthyl ligand. In this manner, compounds **127a** and **127b** were obtained with high enantiomeric purity. Subsequent cross-metathesis with diethyl allylphosphonate (Grubbs II catalyst) and deprotection afforded the target acyclic nucleoside phosphonates **131a** and **131b** (Scheme 192).

#### 2.5.3 Oxygen Nucleophiles

**Scheme 193:**
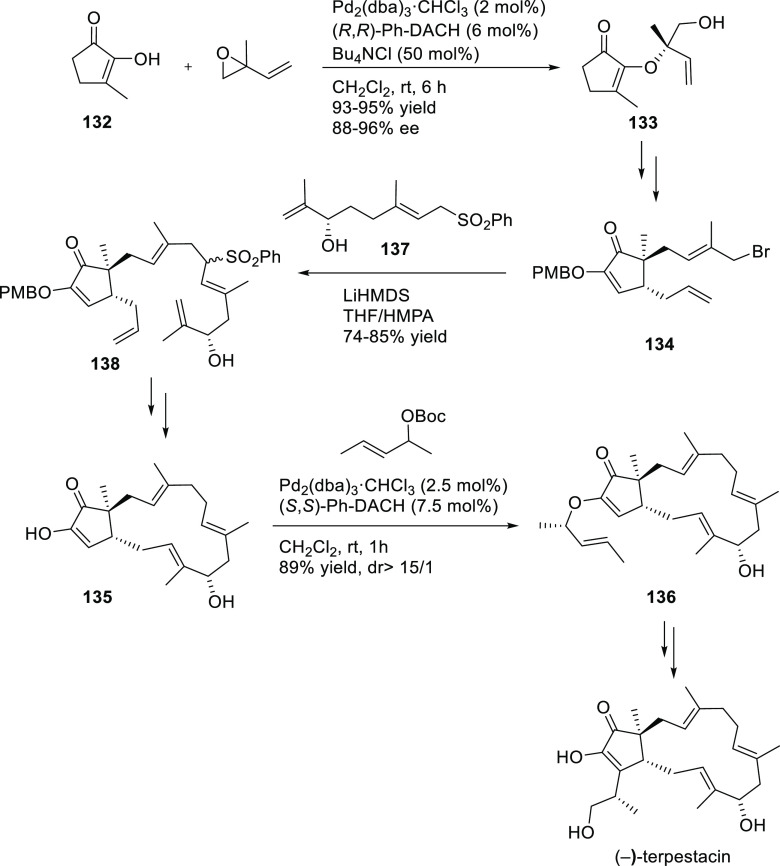
Synthesis of (−)-Terpestacin

Trost and co-workers developed a strategy for the enantioselective total synthesis of the terpenoid (−)-terpestacin.^511^ Their approach started from cyclic 1,2-diketone diosphenol (**132**) (Scheme 193), which was reacted with the Pd η^3^-allyl complex generated from racemic isoprene monoepoxide in the presence of the Trost (*R*,*R*)-Ph-DACH ligand, to afford ether **133** regioselectively in very high enantiomeric purity and very high yield. In combination with a subsequent Claisen rearrangement, this transformation allows the installation of a stereodefined quaternary stereocenter α to the carbonyl group (see **134**). This tactical combination is used again in the final stages of the synthesis in a sequence starting from **135**, which is converted to **136** via a highly diastereoselective Pd-AAA mediated by Pd/(*S*,*S*)-Ph-DACH. Overall, this synthetic scheme provides very efficient control of the configuration of three stereocenters in the final molecule. Other relevant features are the integration of the enantiopure sulfone **137** and allyl bromide **134** by alkylation, and the regioselective ring closing metathesis of **138** leading to the 15-membered carbocycle **135**.

**Scheme 194:**
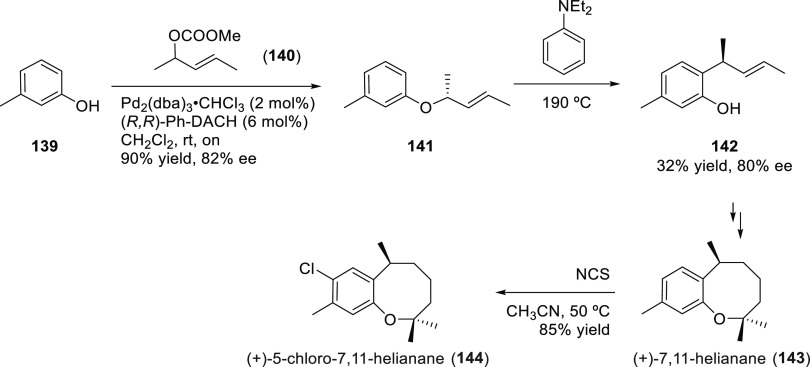
Synthesis of (*S*)-(+)-5-Chloro-7,11-helianane (**144**)

Papeo and co-workers used a Pd-catalyzed, asymmetric allylic *O*-alkylation of *meta*-cresol **139** with allyl carbonate **140** mediated by Pd/(*R,R*)-Ph-DACH to prepare enantioenriched ether **141** with 82% *ee*.^512^ This ether was then submitted to a stereoselective aromatic Claisen rearrangement to afford phenol **142** in low yield (30%) but with preservation of enantiomeric purity in spite of the harsh reaction conditions. From this intermediate, the marine sesquiterpene (*S*)-(+)-7,11-helianane (**143**) was obtained with the same enantiomeric purity by a sequence involving ring closing metathesis with the Grubbs II catalyst as the key step. Moreover, the moderately cytotoxic (*S*)-(+)-5-chloro-7,11-helianane (**144**) was also prepared by simple halogenation of **143** (Scheme 194).

**Scheme 195:**
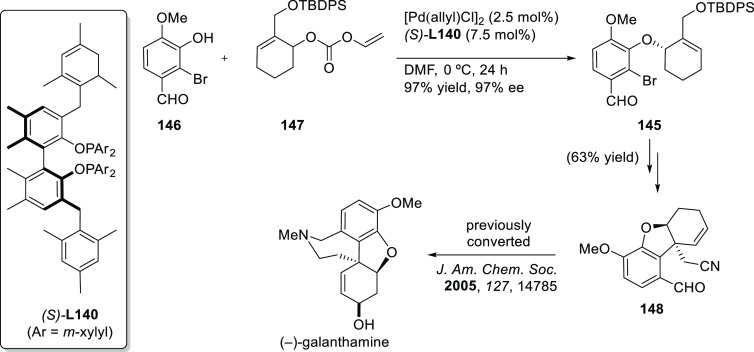
Synthesis of (**−**)-Galanthamine

Zhang and Ojima developed a new family of axially chiral biphenol-based diphosphinite (BOP) ligands that exhibit excellent efficacy in terms of catalytic activity and enantioselectivity when applied to the Pd-catalyzed asymmetric allylic etherification (AAE).^513^ Their potential was demonstrated (Scheme 195) with the preparation of **145** in 97% yield and 97% *ee*, using Pd/(*S*)-XBOP (**L140**) as catalyst. Compound **145** served as the key intermediate in a formal total synthesis of (**−**)-galanthamine from phenol **146** and allyl vinyl carbonate **147** A three step sequence involving deprotection of the silyl ether in **145**, introduction of the cyano group by mesylation/substitution, and intramolecular Heck reaction led to the tricyclic derivative **148** in 63% overall yield. This intermediate had been previously converted to (−)-galanthamine by the Trost group.^514^

**Scheme 196:**
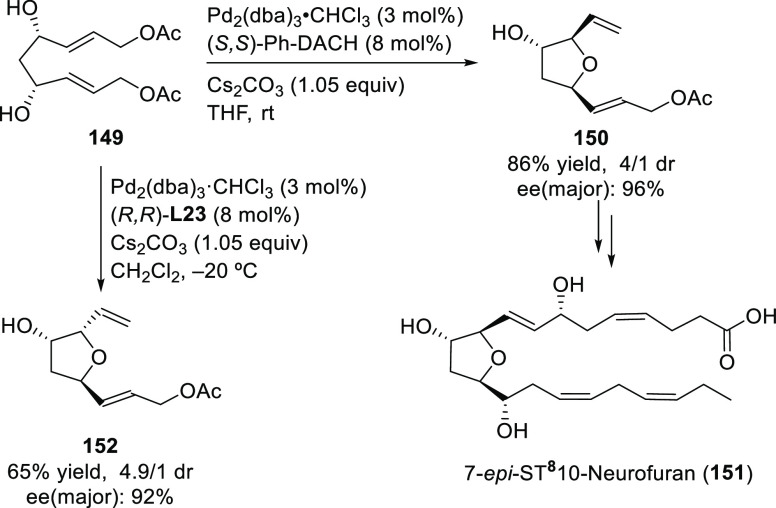
Synthesis of 7-*epi*-ST-Δ^8^-10-Neurofuran

Neurofurans are produced by peroxidation of docosahexaenoic acid esters in neuron membranes and have been suggested as possible biomarkers of oxidative stress, which is considered the principal cause of neurodegenerative diseases. Zanoni and co-workers developed a versatile strategy that, in principle, has the potential to give access to neurofurans of the ST and AC classes from the common *meso* building-block **149**.^515^ A highly enantio- and diastereoselective Pd-catalyzed asymmetric allylic etherification-type cyclization protocol was used to prepare the tetrahydrofuran ring of the ST series of neurofurans **150** by using (*S*,*S*)-Ph-DACH as ligand (Scheme 196). This cyclization product was converted to 7-*epi*-ST-Δ^8^-10-neurofuran (**151**) in a highly convergent manner. Interestingly, by simply switching to the (*R*,*R*)-**L23** ligand (Scheme 26) in the cyclization step, the diastereomeric tetrahydrofuran **152** was formed with high enantiomeric purity. Subjecting compound **152** to the same sequence of reactions used to prepare **151** from **150**, provides access to neurofurans of the AC class.

**Scheme 197:**
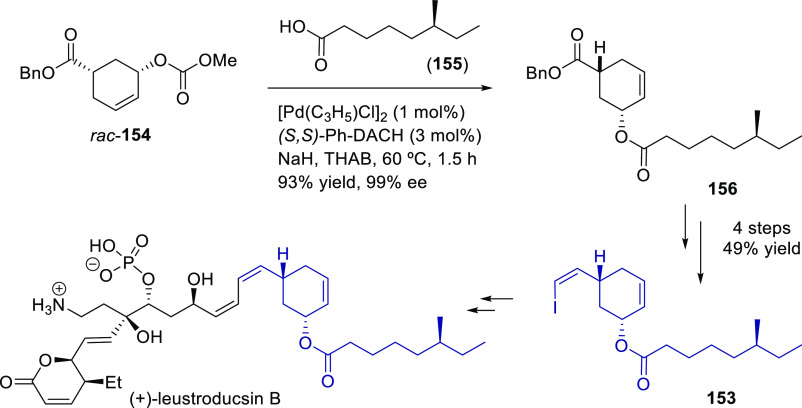
Synthesis of (+)-Leustroducsin B

In 2015, Trost and co-workers reported a highly convergent total synthesis of (+)-leustroducsin B,^516^ a compond belonging to the phoslactomycin family that display interesting bioactivities, such as potent in vitro induction of citokine production by KM-102 cells, increased in vivo resistance to infection by *E. coli*, and trombocytosis induction in mice. The strategy devised for the synthesis of this important target (Scheme 197) was based on the preparation of three key intermediates of similar complexity in terms of size and stereochemistry that could be easily assembled to build the target molecule. For the synthesis of one of them (**153**, highlighted in blue) a Pd-catalyzed deracemization of allyl carbonate **154** with the carboxylate nucleophile **155** was employed. Using the Trost ligand (*S*,*S*)-Ph-DACH, the diester **156** was obtained in high yield (93%) and excellent enantiomeric purity (99% *ee*). A sequence involving chemoselective hydrogenolysis of the benzyl ester, borane reduction of the carboxylic acid, oxidation of the primary alcohol with the Dess–Martin periodinane and Stork–Zhao variation of the Wittig olefination afforded ready-to-couple iodide **153** in 49% yield, with preservation of the enantiomeric purity.

#### 2.5.4 S-Nucleophiles

**Scheme 198:**
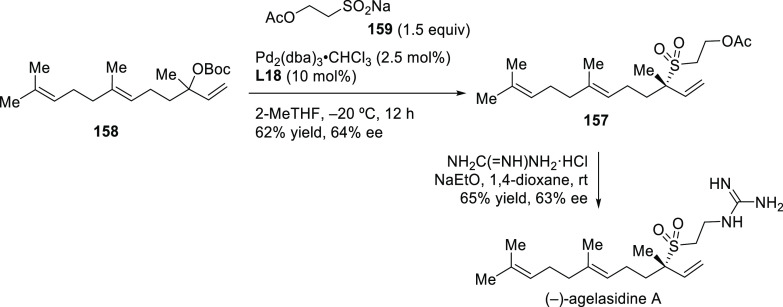
Synthesis of (−)-Agelasidine A

In 2019 Cai and Kleij developed the first general asymmetric approach to sterically encumbered α,α-disubstituted allylic sulfones (**157**) via Pd-catalyzed asymmetric allylic substitution.^87^ The design and use of a new, highly efficient phosphoramidite ligand **L18** (Scheme 15) played a fundamental role in the success of this approach. A wide variety of challenging allylic sulfones featuring quaternary stereocenters could be prepared in this way in high yield with generally excellent regio- and enantioselectivity. The practical value of this method was demonstrated with the development of a synthesis of (−)-agelasidine A, a natural sesquiterpene with antifungal and antimicrobial activity isolated from marine sponges of the genus *Agelas*. To this end, allylic carbonate **158** was treated with sodium alkylsulfinate **159** under optimized reaction conditions to afford the enantioenriched allylic sulfone **157** in 62% yield and 64% *ee*. Treatment of **159** with excess guanidine afforded agelasidine A in 65% yield (Scheme 198).

**Scheme 199:**
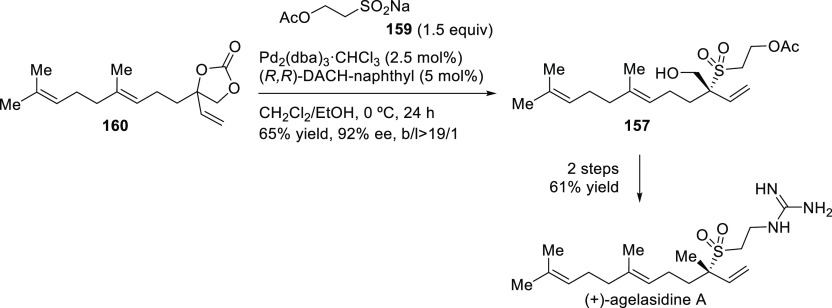
Synthesis of (+)-Agelasidine A

Khan, Zhao, and co-workers very recently developed a similar protocol for the Pd-catalyzed regio- and enantioselective sulfonylation of vinyl cyclic carbonates (such as **160**) with sodium sulfinates.^517^ These authors demonstrated the suitability of this approach for forging sulfone-bearing quaternary carbon stereocenters in high yield and excellent enantioselectivity using the Trost (*R,R*)-DACH-napththyl as a universal ligand. In addition to probing the broad scope of this method with respect to both coupling partners, they also selected (+)-agelasidine A as a target to demonstrate its applicability in total synthesis. The advanced intermediate **160**, synthesized from (*E*)-geranylacetone, could be coupled with sodium 2-acetoxyethane-1-sulfinate (**159**) under the optimized reaction conditions to provide the tertiary allylic sulfone **157** in 65% isolated yield with high regio- and enantioselectivity (>19/1 branched to linear, 92% *ee*). Finally, reduction of the primary alcohol via the corresponding tosylate (85% yield) and subsequent treatment with an excess of guanidine afforded (+)-agelasidine A in 72% yield (Scheme 199).

#### 2.5.5 Oxidation

**Scheme 200:**
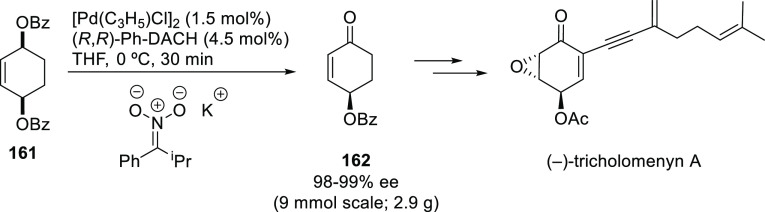
Synthesis of the Enantiopure Epoxyquinoid (−)-Tricholomenyn A

In 2014, Trost and co-workers reported an efficient method for the preparation of chiral cycloalkenone derivatives via asymmetric Pd catalysis.^100^ The enantioselective oxidation of *meso*-cyclohex-2-ene-1,4diylbenzoate (**161**) with a nitrate using Pd/(*R*,*R*)-Ph-DACH as catalyst led to compound **162**, which was then further transformed into enantiopure epoxyquinoid (−)-tricholomenyn A (Scheme 200).

#### 2.5.6 Summary and Outlook

The examples discussed in sections 2.5.1 to 2.5.5 clearly illustrate the potential of asymmetric allylic substitutions in total synthesis. However, as it is not uncommon with other reactions, only highly trusted, well established procedures have been regularly selected to be integrated in total synthesis. As a consequence of this understandable, yet rather conservative way of thinking, only a small subset of the huge amount of knowledge accumulated on asymmetric allylic substitution has found application in total synthesis to date.

Carbon nucleophiles (section 2.5.1), useful for forging all-carbon stereocenters, have been widely used in this field, but the variety of allylic substrates regularly used is rather limited. Thus, heavily substituted acyclic allylic systems, as well as cyclic allylic systems in general, have been barely used as substrates in total synthesis, although efficient chiral catalysts with a high chance to work are available. However, because the failure of a single reaction in a multistep synthetic sequence can have severe consequences, only rather safe reactions with ample precedence are generally chosen. This limitation is less distinctive for nitrogen nucleophiles (section 2.5.2), fundamental for the preparation of chiral enantioenriched allylamines. Cyclic allylic substrates have found ample application in these cases, but the scope of transformations based on acyclic substrates remains narrow. Oxygen nucleophiles (section 2.5.3), in spite of promising results obtained with them, have only found minor applications in total synthesis.

The availability of a chiral ligand or catalyst is another important factor that may impede its application. Often, suitable ligands have to be prepared through multistep syntheses as only very few of the many chiral ligands applied in asymmetric allylic substitution have been commercialized. Hopefully, more chiral ligands will become commercially available in coming years, which will foster the application of asymmetric allylic substitution reactions in total synthesis.

Clearly, the current level of development of asymmetric allylic substitution reactions will lead to a more intense use in total synthesis in the future with the inclusion of many further allylic substrates and nucleophiles. For instance, there has been substantial progress in the development of dual Pd/organocatalyst systems that open up new possibilities for applying asymmetric allylic substitution in complex molecule synthesis. The increasing availability of high-throughput experimentation (HTE) methods, allowing the fast screening of ligands, metals and reaction conditions, may also help in overcoming the restrictions that have prevented until now a wider use of asymmetric allylic substitution in total synthesis.

### 2.6 Comparison with Other Metals

In addition to complexes with Pd, catalysts derived from other metals, such as Ir, Rh, Co, Mo, W, Ru, Fe, Cu, and Ni, have been employed in enantioselective allylic substitutions.^10^ Among these, complexes with Ir have turned out to be particularly versatile in organic synthesis.^56,518−521^

Enantioselective Ir-catalyzed allylations have been known since 1997.^522^ They are characterized by the formation of branched, chiral products from both branched and linear allylic carbonates and acetates and, thus, exhibit a regioselectivity complementary to that of Pd. The reactions proceed with a high degree of regio- and enantioselectivity when linear substrates are used. In contrast, reactions with racemic branched allylic substrates usually occur with lower enantioselectivity as a result of π–σ–π isomerization being slow compared to nucleophilic attack. This memory effect is, however, a function of the ligand, and certain Ir catalysts are known that provide products with excellent regio- and enantioselectivity also from branched substrates. The problem can otherwise be overcome by sequential Pd-catalyzed isomerization, to convert the racemic branched allylic substrates into their linear isomers, followed by Ir-catalyzed allylic substitution.^523^

Phosphoramidite ligands have proven to be particularly successful for Ir-catalyzed allylic substitutions, but several other types of ligands have also been used. In addition to stabilized carbon nucleophiles, a wide range of O-, N-, and S-nucleophiles, as well as F^–^ can be employed, thus allowing a multitude of chiral building blocks to be prepared. Products with quaternary stereogenic centers have been prepared with high enantioselectivity. The reactions are tolerant to a wide range of functional groups and have been applied in stereoselective total syntheses of a variety of complex chiral molecules. Several examples of diastereoselective reactions with prochiral nucleophiles are known, although there are no general methods yet for efficient stereochemical control of prochiral nucleophiles.

The Ir-catalyzed processes proceed by inversion–inversion mechanisms, and thus, like the Pd-catalyzed reactions, with overall retention of configuration. Basic reaction conditions are needed to promote the reactions effectively. The catalytically active complex is a metallacycle with an Ir–C bond, formed via C–H activation of the ligand.

An alternative procedure, which results in high enantioselectivity from branched substrates, uses branched allylic alcohols under acidic conditions.^524^ A particular feature of reactions under these conditions is that weakly activated alkenes can be used as nucleophiles, a result of the highly electrophilic allylic intermediates.

Rhodium allyl complexes isomerize slowly and processes catalyzed by them therefore proceed with a high degree of conservation of the stereochemistry of the branched substrates, whereas linear substrates predominantly afford linear products.^525,526^ By selecting conditions under which nucleophilic addition becomes slow compared to isomerization of the intermediate allyl complexes, high enantioselectivity may be achieved in Rh-catalyzed reactions. Rh complexes catalyze allylic substitutions of a range of stabilized and nonstabilized carbon nucleophiles, as well as aminations and etherifications. The reactions have been proposed to proceed via configurationally stable distorted η^3^-allyl or enyl intermediates and involve double inversion of configuration for stabilized and overall inversion for nonstabilized nucleophiles. A few successful examples of the use of prochiral nucleophiles in combination with achiral allylic substrates are known. The scope of the process is, however, limited compared to Pd- and Ir-catalyzed reactions.

Cobalt-catalyzed allylic substitutions have been only scarcely studied.^527^ Recently, however, allylic aminations^528^ and alkylations^529^ of branched substrates were achieved with high regio- and enantioselectivity. Reactions with racemic branched carbonates give branched products with high enantioselectivity in the presence of oxazoline-based NPN-ligands, whereas linear substrates react slowly, but with similar selectivity. Vicinal quaternary carbon centers can be constructed by use of tertiary allylic carbonates.^530^

Mo-catalyzed allylations have been limited to stabilized C-nucleophiles.^531,532^ Recently, however, sodium sulfinates were used in combination with achiral ligands to produce racemic tertiary sulfones.^533^ Readily available modular bispyridylamides,^534^ and bisdihydrooxazoleamides^535^ serve as efficient chiral ligands. Rapid equilibration of intermediate allyl complexes may lead to a single major complex, and therefore high regio- and enantioselectivities are observed from linear, as well as branched substrates; the two types of substrates typically lead to essentially identical results. The reactions proceed by overall retention of configuration, but unlike reactions with Pd and Ir catalysts; this is a result of a double-retention mechanism.^536^ Since molybdenum compounds are inexpensive and Mo(0) can be employed in the form of stable Mo(CO)_6_, together with stable ligands for in situ preparation of the catalyst under microwave conditions, not requiring inert conditions,^537^ the Mo-catalyzed allylation is the method of choice for reactions with certain stabilized carbon nucleophiles. Mo-catalyzed allylations have been applied to the synthesis of several biologically active compounds.

Tungsten complexes with phosphinooxazoline ligands can be used for enantioselective substitutions using linear substrates, although the enantioselectivity is lower than with Mo.^538^ Reactions catalyzed by W are stereospecific, and therefore, no enantioselectivity is observed in reactions with branched racemic substrates.

Iron catalysts are attractive because of their low price and low toxicity and the high abundance of the metal. Fe allyl complexes isomerize slowly and allylic substitutions therefore proceed by a high degree of conservation of the stereochemistry and substantial regiochemical memory effects, the extent of which is a function of ligand structure.^527,539^ A range of allylic carbonates have been reacted with C-, as well as N-, O-, and S-nucleophiles.

Like Fe catalysts, but in contrast to Mo and W catalysts, complexes with Ru react with heteroatom (N, O, S), as well as C-nucleophiles.^527^ Branched products are preferentially formed starting from linear as well as branched substrates, although under certain conditions memory effects are observed, resulting in retained stereochemistry of branched substrates. Only a few examples of enantioselective Ru-catalyzed substitutions are known. Recently, however, a branched-selective allylic alkylation catalyst was shown to provide N-alkylated isatins with high regio- and enantioselectivity.^540^

For enantioselective allylic alkylations with nonstabilized carbanions, such as organozinc reagents, Cu catalysts are most frequently employed.^541−544^ The reactions result in the enantioselective installation of alkyl groups at the allylic position, and they serve as valuable complements to catalysts with Pd and Ir. All-carbon quaternary stereogenic centers can be constructed via copper-catalyzed enantioselective allylic alkylations of (*E*)- and (*Z*)-trisubstituted allyl bromides. Cyclic and acyclic allylic acetates, carbonates, phosphates, halides, and ethers react, and organozinc, organomagnesium, organoaluminum, organozirconium, and organolithium compounds can be used as nucleophiles. The reactions tolerate various functional groups. A variety of different ligands have been used, including phosphoramidites, phosphines, phosphites, N-heterocyclic carbenes, and peptide-based ligands.

The reactions usually occur via an S_N_2′-type mechanism and are therefore regiospecific. They proceed by transmetalation to form a Cu(I) complex, followed by π-complex formation and subsequent oxidative addition to give a Cu(III) σ-allyl complex. The oxidative addition is also the enantiodiscriminating step.

In recent years chiral racemic allylic substrates have been subjected to kinetic resolutions or dynamic kinetic resolution using Cu catalysts. In an enantioconvergent process, in which both enantiomers of the starting material react via two different reaction routes, close to enantiopure product could be obtained from racemic starting material.^545^

Nickel catalysts can be used for reactions with stabilized and nonstabilized carbanions as well as heteroatom nucleophiles.^527^ With nonstabilized carbanions net inversion of configuration is observed, while all other nucleophiles react with overall retention. Recently several examples of Ni-catalyzed enantioselective allylation of β-ketoesters using allylic alcohols have been reported, which allow for the construction of quaternary all-carbon stereocenters.^546^

This comparison reveals that the most versatile catalysts for enantioselective allylic substitutions are those based on Pd, Ir, and Cu. The three types of catalysts are highly complementary, with different scope, different selectivities, and different outcomes. As a rule, Pd and Ir complexes are the superior catalysts for reactions with stabilized nucleophiles. The constitution of the product is dictated by the metal, with Pd catalysts normally giving products from nucleophilic attack at the least hindered site of unsymmetric allylic substrates, and Ir catalysts at the more hindered site. Catalysts with Mo show the same site selectivity as Ir catalysts but are cheaper and more easily handled, although considerably narrower in scope. In reactions with nonstabilized nucleophiles, the optimal choice is usually a catalyst with Cu. The different behavior of the catalysts broadens the synthetic versatility of allylic substitutions, making these reactions among the most powerful enantioselective synthetic processes.

Complexes with first row elements have so far received limited attention as catalysts for enantioselective allylic substitutions, but are presently gaining increasing interest. Future development may well widen their scope, thereby providing access to more abundant and more sustainable catalysts.

## 3 Asymmetric Decarboxylative Allylic Substitution

### 3.1 Decarboxylative Allylation of Enolates

**Scheme 201:**
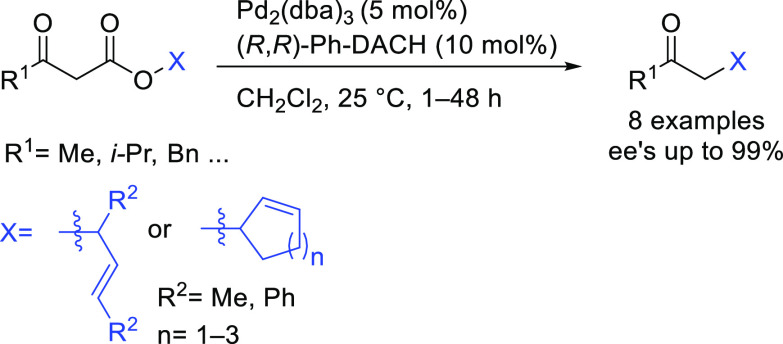
DAAA of Several β-Keto Allyl Esters

The first decarboxylative asymmetric allylic alkylation (DAAA) was reported in 2004 by Burger and Tunge who used several linear and cyclic β-keto allyl esters as substrates (Scheme 201).^364^ With Trost’s ligand (*R,R*)-Ph-DACH and Pd_2_(dba)_3_, ee’s of up to 99% and high yields could be obtained in the formation of several α-allyl ketones. In this case, the stereocenter is formed at the β-position through stereocontrol at the electrophilic allylic unit, which is directly bonded to the palladium complex. The reaction allows regioselective formation and allylation of enolates through Pd-mediated cleavage of the allyl ester into a Pd-allyl complex and a β-keto carboxylate, followed by decarboxylation (for mechanistic aspects, see section 3.3).

**Scheme 202:**
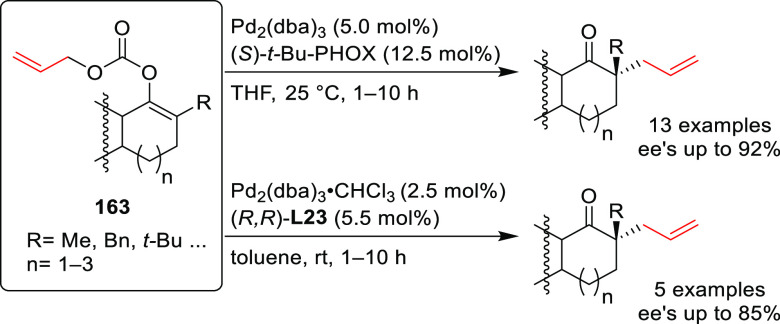
DAAA of Cyclic Allyl Enol Carbonates Using (*S*)-*t*-Bu-PHOX and Trost’s Ligand (*R,R*)-**L23**

Also in 2004, Stoltz published the first DAAA using cyclic allyl enol carbonates of type **163** as substrates in a study, in which a wide range of chiral ligands were tested.^365,547^ In this reaction the stereogenic center is introduced at the α-position of the pro-chiral nucleophilic enolate intermediate. The ligand screen demonstrated that P,N ligands were optimal at generating the quaternary stereocenters. With (*S*)-*t-*Bu-PHOX and Pd_2_(dba)_3_, the first enantioselective preparation of 2-allyl-2-methyl cyclohexanone (R = Me, 89% *ee*) was accomplished (Scheme 202). This was noteworthy as the product cyclohexanone was not available heretofore via asymmetric allylic alkylation because of problems with enolate scrambling in situ. Stoltz also showed that the product was accessible from silyl enol ethers with *ee* values up to 92% generating the Pd-allyl complex externally from diallyl carbonate. This strategy has been further used for the synthesis of enantioenriched α-quaternary cycloheptanones, which has been further transformed to a range of cyclopentanoid and cycloheptanoid core structures with all-carbon quaternary stereocenters.^548−550^

The following year, Trost reported an evaluation of Pd Trost ligand complexes for this decarboxylative allylation (Scheme 202)^551^ and the optimal results were found using ligand (*R,R*)-**L23** (Scheme 26). This study also successfully addressed the enantioselective synthesis of compounds containing α-allyl tertiary centers (e.g., (*R*)-2-allylcyclohexan-1-one, (*S*)-2-allyl-2,3-dihydro-1*H*-inden-1-one, ...), which were obtained in up to >99% *ee*. Moreover, it was noted that a wider range of allyl groups was tolerated when this ligand class was employed. As side products, ketones with α-methyl tertiary centers were observed (yields ranging from 0 to 26%), which were proposed to be formed by the protonation of the Pd-enolate.^552−558^

**Scheme 203:**
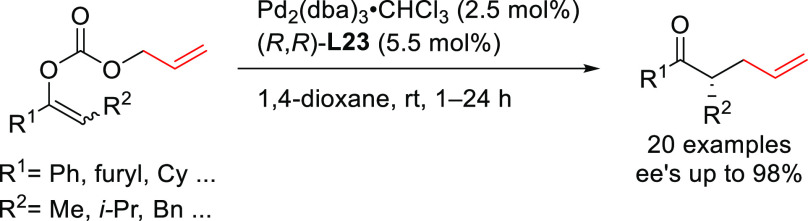
DAAA Forming Acyclic Ketones Using Pd/**L23** Catalyst

Trost further demonstrated that the DAAA was amenable to linear substrates (Scheme 203).^366^ A selection of important points were observed during this investigation. The diallylated product was formed when toluene and THF were used, but changing to 1,4-dioxane instead allowed this problem to be overcome. It was noted that the substituent branching, as well as the starting material *E*/*Z* configuration, had a major effect on catalytic performance. The (*E*)-isomer gave higher *ee* values in a significantly lower reaction time compared to the corresponding (*Z*)-isomer. It was found that the *E*/*Z* configuration did not play a role in the regioselectivity observed while the R^1^ substituent influenced the level of enantioselectivity dependent on its electronic nature, with electron-withdrawing substitutents exhibiting a lower *ee*. These results suggest that asymmetric induction is due to a combination of steric and electronic components.

**Scheme 204:**
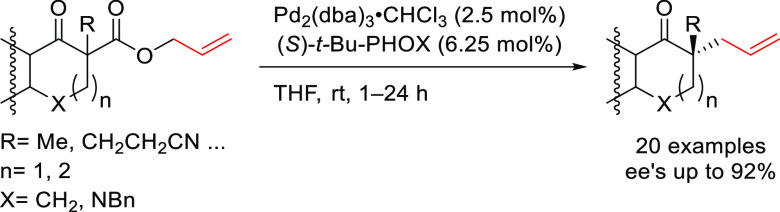
DAAA on β-Keto Allyl Esters Using Pd/*t*-Bu-PHOX Catalyst

The issues faced with the regioselective synthesis of cyclic allyl enol carbonates of type **163**, although very successful DAAA substrates, limited the scope and applications in synthesis. To deal with this issue, Stoltz and co-workers developed an alternative approach inspired by previous work of Tsuji and Saegusa.^559,560^ They showed that the bench-stable β-keto allyl esters, which possess a quaternary center, gave excellent yields and enantioselectivities in the DAAA reaction (Scheme 204).^561^ Stoltz described this process as “stereoablative enantioconvergent catalysis”, that is, the stereogenic center of the β-keto allyl ester substrate is removed yielding an achiral intermediate, which is subsequently transformed into an enantioenriched product. Stoltz’s group later demonstrated that the use of ligand **L122** allowed the Pd-catalyzed DAAA using low Pd concentrations.^562^

**Scheme 205:**
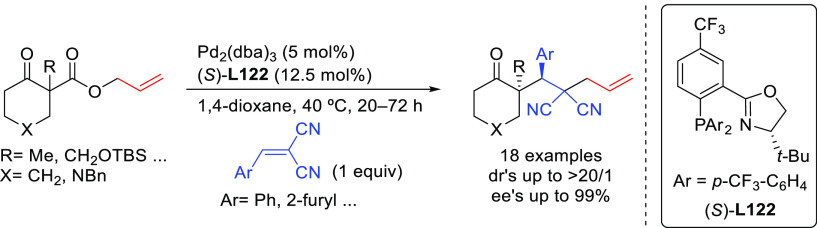
Pd-Catalyzed Enolate Alkylation Cascade

Stoltz also early demonstrated that the intermediate Pd-enolate species, in situ generated via DAAA, can be trapped with activated Michael acceptors.^563^ As a result, ketones containing adjacent quaternary and tertiary stereocenters were prepared in high diastereo- and enantioselectivities (dr’s up to >20/1 and *ee* values up to 99%; Scheme 205).

**Scheme 206:**
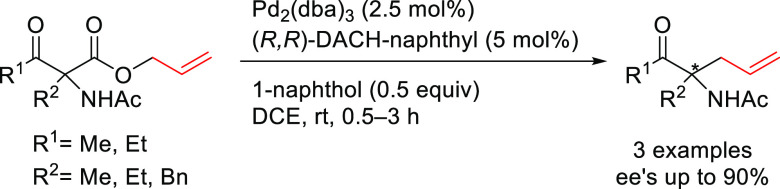
DAAA Employing Acyclic Allyl α-Acetamido-β-ketocarboxylates

Murakami and co-workers subsequently published their work on the DAAA employing acyclic allyl α-acetamido-β-ketocarboxylates employing the (*R*,*R*)-DACH-naphthyl Trost ligand (Scheme 206).^564^ They noted that the use of phenol derivatives (i.e 1-naphthol) was necessary to observe very high levels of enantioselectivity and suggested that hydrogen bonding between this protic source and the α-acetamido unit was critical for the enhancement of the enantioselectivities. Interestingly, they also found that the DAAA of acyclic β-ketocarboxylates without an α-acetamido moiety led to no selectivity, hinting at the important role of the α-acetamido group in the asymmetric induction under these reaction conditions.

**Scheme 207:**
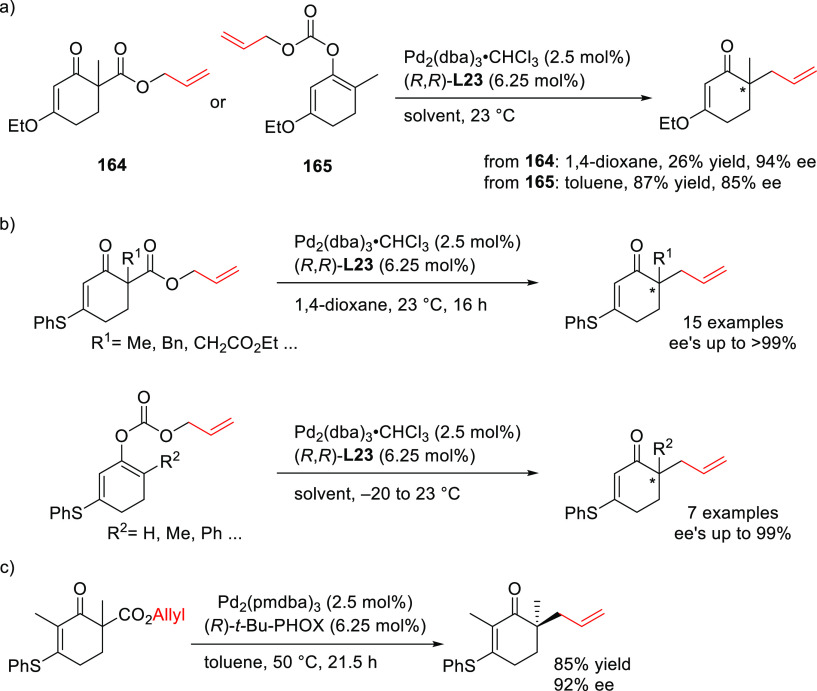
DAAA of Cyclic Vinylogous Thioesters

The Trost group extended the scope of the DAAA to include cyclic vinylogous esters and thioesters (Scheme 207), which are valuable substrates as they behave as masked 1,3-dicarbonyls.^565^ Initially β-keto allyl esters **164** were used but they needed long reaction times and furnished rather low yields (Scheme 207a). The low reactivity of **164** was explained by the relatively high energy required to break the C–C bond in the decarboxylation step because of the low electrophilicity of the carbonyl group of the vinylogous ester. Substituting the ethoxy group in **164** by a thioether group (which has lower π-donating ability), or by using allyl enol carbonates **165**, solved these problems, providing the allylated compounds in high yields and enantioselectivities (Scheme 207b). The Stoltz group made similar observations during their investigations on vinylogous thioesters, which were found to be substantially more reactive than the corresponding vinylogous esters (Scheme 207c).^566^ The DAAA transformation was applied in the total synthesis of (+)-carissone, the formal synthesis of (−)-α-eudesmol and (+)-cassiol (see section 3.5).^567^

**Scheme 208:**
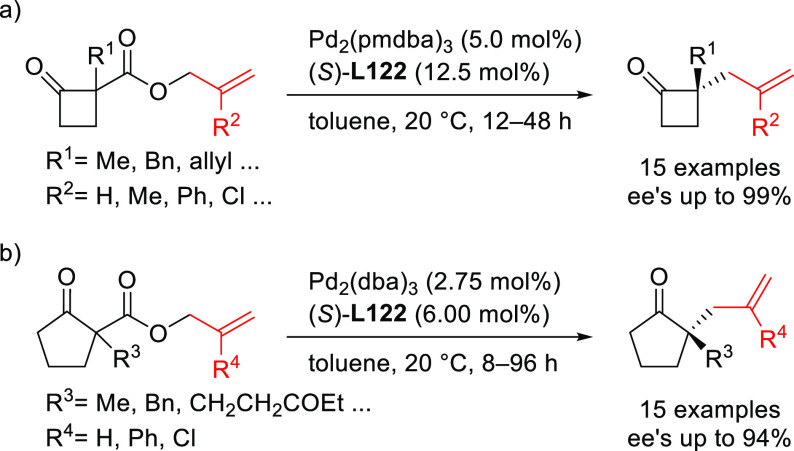
DAAA of Cyclobutanones and Cyclopentanones Using Pd/**L122** Catalyst

In addition to cyclohexanone, cycloheptanone, and cyclooctenone derivatives, which are the most widely used substrates for the DAAA reaction, the Stoltz group also investigated cyclobutanone-derived β-keto allyl estersin 2013 (Scheme 208a).^568^ The electron-deficient *p*-(CF_3_)_3_-*t*-Bu-PHOX ligand **L122** was found to induce better *ee* values compared to the parent *t*-Bu-PHOX ligand. A variety of allyl fragments were screened and high levels of enantioselectivity were afforded throughout. Stoltz showed the synthetic usefulness of the process by subsequently forming γ-lactones, cyclopentanones, γ-lactams, and spirocyclic cyclobutanones with conservation of *ee*. Cyclopentanones are more problematic substrates than cyclohexanones, often providing lower catalytic performances. The Stoltz group reinvestigated this limitation and reported an enantioselective synthesis of α-alkyl and α-benzyl cyclopentanones in 2015 (Scheme 208b).^569^ While the *ee* values were consistently high, it was observed that the reactivity depended on the electronic properties of the aryl group of α-benzyl cyclopentanone derivatives. As an example, cyclopentanone with electron-donating *p*-methoxybenzyl substituents at R^4^ were formed in shorter reaction times (8 h, >99 yield) compared to an analog with an electron-withdrawing *p*-CF_3_-benzyl substituent, which was formed in moderate yield of 56% after 96 h.

**Scheme 209:**
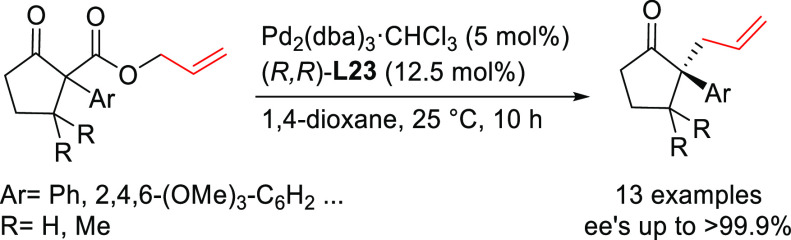
DAAA of α-Aryl Cyclopentanones Using Pd/**L23** Catalyst

Guiry and co-workers exploited the DAAA for the highly enantioselective formation of α-allyl-α-aryl cyclopentanones (Scheme 209).^570^ α-Aryl β-keto allyl ester substrates afforded sterically hindered products in excellent *ee* values with Trost-type ligands. While the (*R,R*)-Ph-DACH ligand afforded an 86% *ee* for the 2,4,6-trimethoxyphenyl-containing model substrate, the (*R,R*)-ANDEN phenyl Trost ligand **L23** gave exceptional levels of selectivity for the enantioselective synthesis of an all-carbon quaternary stereocenter (up to 99.9% *ee*). A study of the substrate scope showed that a range of aryl groups were tolerated at the α-position with cyclopentanones possessing di-*ortho-*substitutions affording the highest levels of enantioselectivity. The usefulness of this transformation was shown when it was exploited as the key enantioselective step for the preparation of the marine natural product (+)-tanikolide (s*ee*section 3.5).

**Scheme 210:**
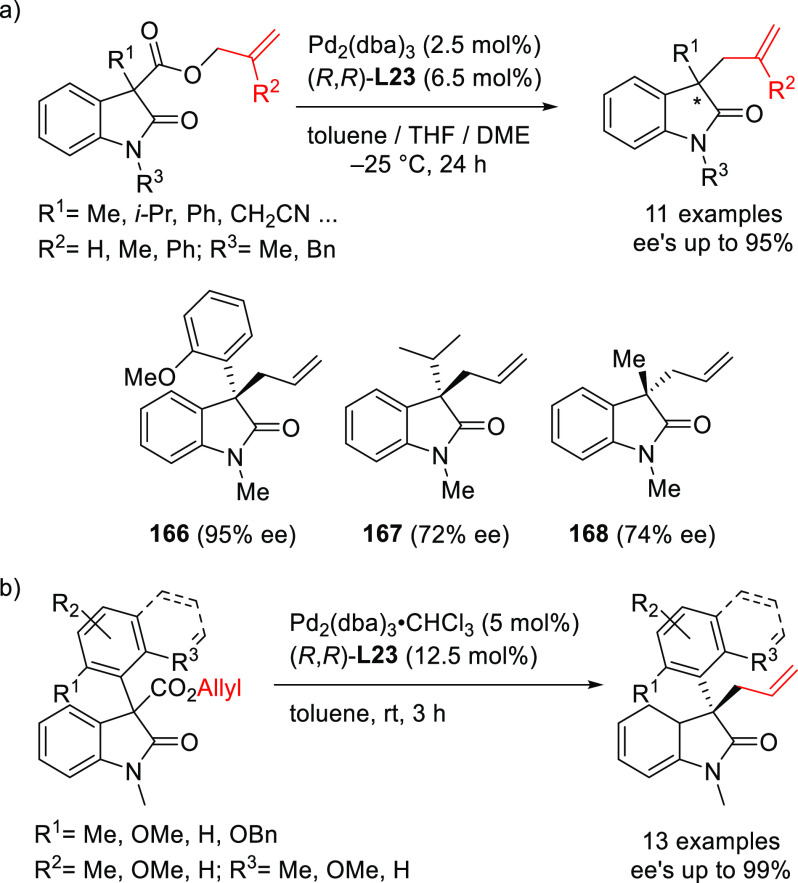
DAAA of α-Alkyl or α-Aryl Oxindoles Using Pd/**L23** Catalyst

Taylor, and subsequently Guiry, developed the Pd-catalyzed DAAA enabling the installation of quaternary stereocenters at the 3-position of oxindoles.^571,572^ Both groups showed that ligands of the PHOX type were inferior to the Trost type ligands with ligand (*R*,*R*)-**L23** being optimal. Taylor showed that for α-alkyl and monosubstituted α-aryl oxindoles, high enantioselectivities (up to 95% *ee*) could be obtained when reactions were performed at −25 °C (Scheme 210a).^571^ Interestingly, they found that the absolute configuration of the products depended on the size of the R^1^ substituent (Scheme 210a, compounds **166** and **167** vs **168**). Guiry had a focus on substrates with two aryl *ortho*-substituents and naphthyl substituents, which afforded products with excellent levels of enantioselectivity (up to 99% *ee*) (Scheme 210b).^572^

**Scheme 211:**
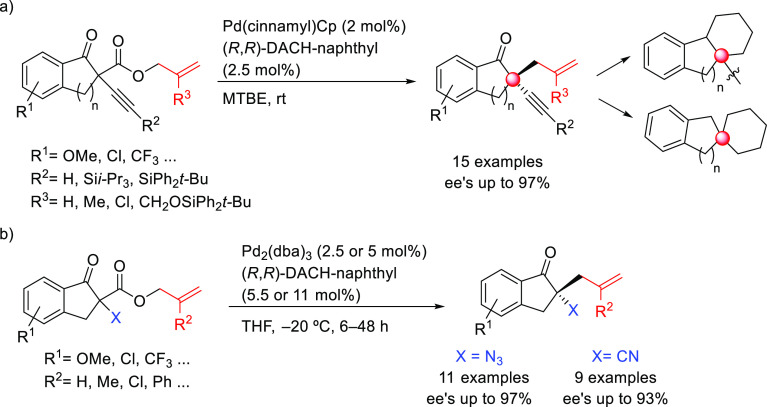
DAAA of α-Substituted Indanones and Tetralones

The extension of Pd-catalyzed DAAA to include tetralone and indanone substrates possessing α-alkynyl substituents was reported by Waser in 2014 (Scheme 211a).^573^ The α-alkynyl β-keto allyl esters were prepared using hypervalent iodine reagents under benign conditions. High enantioselectivities (up to 97% *ee*) were obtained in ethereal solvents such as MTBE with the Pd complex formed from Pd(cinnamyl)Cp and the (*R*,*R*)-DACH-naphthyl ligand. Studies of the substrate scope covered allyl substituents and modifications of the aromatic ring influencing the electronic properties. The enantioenriched 1,5-enynes formed in the DAAA step were transformed using ring-closing metathesis or cycloisomerization into fused tricyclic and spirocyclic products. In 2015 Waser subsequently developed the DAAA of α-azido and α-cyano β-keto allyl esters with very high levels of enantioselectivity of up to 97% *ee* and 93% *ee*, respectively (Scheme 211b).^574^ Benziodoxole hypervalent iodine reagents were again used to prepare the substrates used in catalysis by electrophilic azidation or cyanation. The products formed in the DAAA process with (*R*,*R*)-DACH-naphthyl were converted in a facile manner into important nitrogen-containing functional groups such as triazoles, amides, and amines.

**Scheme 212:**
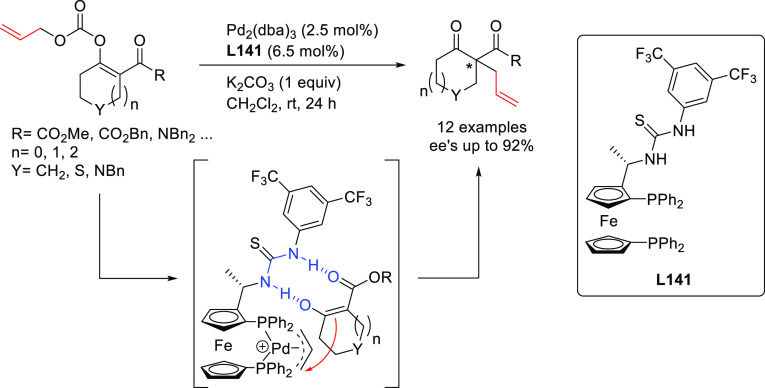
Thiourea-Assisted DAAA of β-Ketoesters

Zhang and Chung developed the Pd-catalyzed DAAA of β-keto esters employing the thiourea-containing ligand **L141** (Scheme 212).^575^ The reaction proceeded with high levels of enantioselectivity (up to 92% *ee*) for six-membered ring allyl enol carbonates containing a variety of ester substituents at the α-position. However, only low levels of enantioselectivity were observed for substrates possessing alternative ring sizes or heterocycles (such as tetralones, cyclopentanones, etc.). Key to the success of the reaction was the addition of one equivalent of K_2_CO_3_. The success of the thiourea catalyst was rationalized by the hydrogen-bonding interactions between the substrate and the catalyst, whereas the role of K_2_CO_3_ was not discussed.

**Scheme 213:**
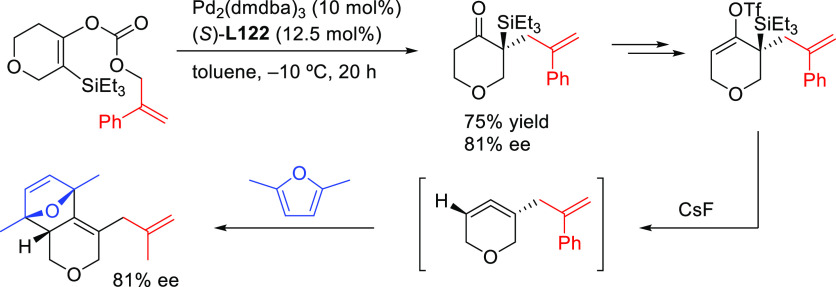
DAAA of α-Silyl-Substituted Enol Carbonates

Houk, Stoltz, Garg, and co-workers recently disclosed the Pd-catalyzed allylic alkylation of an α-silyl-substituted enol carbonate, which allowed access to an enantioenriched silyl triflate precursor (Scheme 213).^576^ Such a precursor was key to demonstrate that the trapping of the enantioenriched oxacyclic allene by Diels–Alder reaction occurs with complete transfer of stereochemical information.

**Scheme 214:**
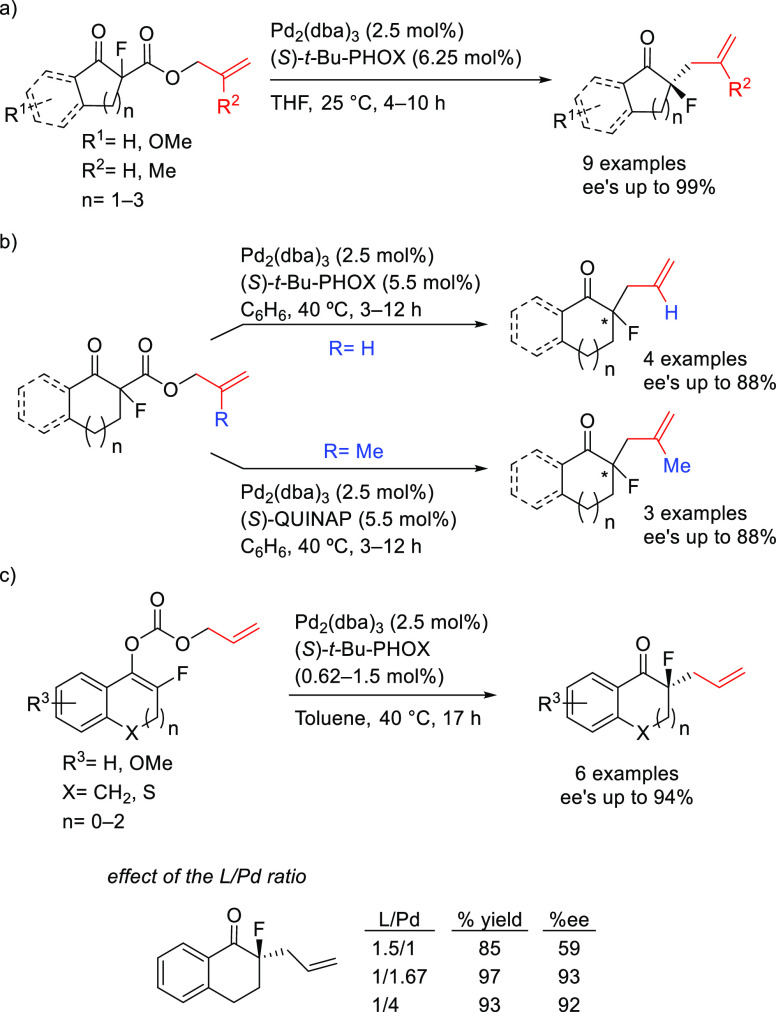
Enantioenriched α-Fluoroketones by DAAA

The introduction of fluorine atoms into biologically active compounds is of great importance in both the agrochemical and pharmaceutical industries.^577,578^ Pd-catalyzed DAAA reactions offer a potential enantioselective approach to α-fluoroketones.^579−583^ Stoltz demonstrated the application of DAAA to prepare an enantioenriched tertiary fluoride, 2-allyl-2-fluorocyclohexanone, in 91% *ee* (Scheme 204, R = F).^561^ Contemporaneously, Nakamura described the synthesis of a range of enantioenriched α-fluoroketones using the same Pd-(*S*)-*t*-Bu-PHOX ligand system (Scheme 214a).^584^ While excellent enantioselectivities (up to 99% *ee*) were obtained for a series of cyclic α-fluoro substrates, acyclic α-fluoro substrates afforded products with only moderate enantioselectivities (up to 51% *ee*).

The Tunge group also reported the catalytic asymmetric synthesis of cyclic α-allylated α-fluoroketones and found that Pd complexes of P,N-ligands proved to be optimal.^585^ The Pd/DACH-phenyl-Trost complex, which is particularly successful in DAAA transformations, did not catalyze the reaction. Comparison of the results obtained using (*S*)-QUINAP and (*S*)-*t*-Bu-PHOX showed that the efficacy of the ligand depended on the substrate type tested. In general (*S*)-QUINAP proved to be superior for those substrates possessing methyl groups on the allyl unit (up to 88% *ee*), whereas (*S*)-*t*-Bu-PHOX performed better with cyclic α-fluoro substrates with unsubstituted allyl systems (up to 88% *ee*) (Scheme 214b). Paquin showed that the Pd/ligand ratio was important to obtaining high levels of enantioselectivity in the DAAA to form α-fluoroketones via allyl enol carbonates (Scheme 214c).^586^ Using normal Pd/ligand ratios (1/1.25), α-fluoroketones were generated in low *ee* values (e.g., 59% for (*R*)-2-allyl-2-fluoro-3,4-dihydronaphthalen-1(2*H*)-one). In contrast, employing Pd/ligands ratios between 1.67/1 and 4/1 afforded high enantioselectivities of up to 94% *ee*. This effect of the Pd/ligand ratios did not translate to DAAAs with analogous β-ketoester or silyl enol ether substrates.

**Scheme 215:**
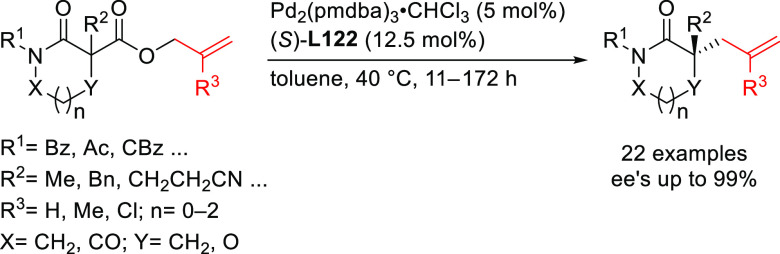
DAAA of α-Alkyl Lactams or Imides

The DAAA has also been applied to heterocyclic substrates owing to the prevalence of heterocyclic motifs in natural products and biologically active compounds. Stoltz reported the enantioselective preparation of quaternary all-carbon containing stereocenters in *N*-heterocycles from lactams and imides containing an α-allyl ester (Scheme 215).^587^ Poor reactivity was observed for *N-*alkyl substrates containing electron withdrawing *N*-protecting groups. Pd complexes of the electron-deficient *p*-(CF_3_)_3_-*t*-Bu-PHOX ligand **L122** (Scheme 205) exhibited higher reactivity and yielded higher *ee* values than the parent *t*-Bu-PHOX complex. Piperidinones, pyrrolidinones, and caprolactams were found to be excellent substrates. The synthetic utility of the reaction was demonstrated by applying it to the formal synthesis of the microtubule-disrupting agent (+)-rhazinilam and the *Aspidosperma* alkaloid (+)-quebrachamine (see section 3.5). The higher levels of asymmetric induction obtained with lactam and imide substrates compared to cyclic ketone substrates prompted a study of the contributions of steric, electronic, and stereoelectronic factors in each substrate type.^588^ New enaminone substrates that provided a comparison of the electronic properties of the lactam enolates were tested in DAAA and the results showed that the high levels of enantioselectivity seen with lactams and imides were due to the α′-functionality rather than the electronic properties of the enolate. The screening of a series of *N-*protected enaminones showed that those possessing electron-rich protecting groups reduced the reaction rate as well as the *ee*. With such substrates, it was seen that the *t*-Bu-PHOX ligand demonstrated enhanced reactivity compared to the (CF_3_)_3_-*t-*Bu-PHOX analog **L122**.

**Scheme 216:**
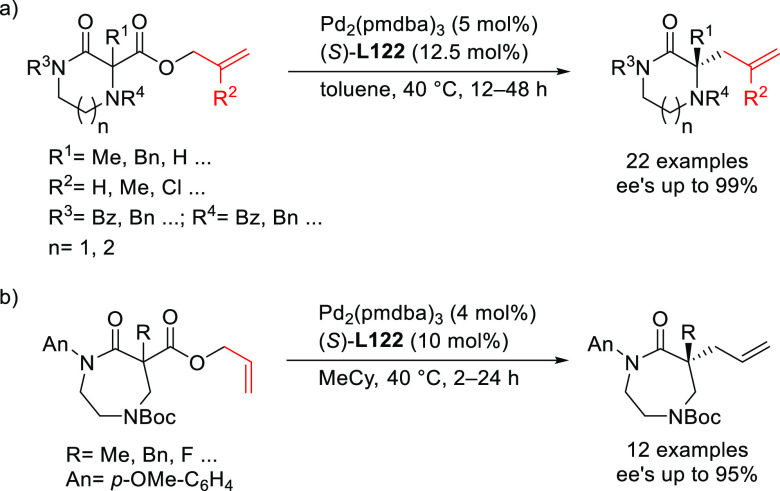
Pd-Catalyzed DAAA of (a) Piperazinones and (b) 1,4-Diazepan-5-ones

The Stoltz group also applied the DAAA method for the enantioselective preparation of α-tertiary- and quaternary-substituted piperazin-2-ones (Scheme 216).^589^ It was deemed necessary to protect both piperazinone nitrogen atoms and, after several protecting groups were screened, excellent *ee* values of up to 97% were obtained (Scheme 216a). Both sp^2^-hybridization at the N4 position and use of a benzoyl-protecting group at N1 with *ortho*-substitution had a negative effect on the *ee*. The synthetic utility of this transformation was demonstrated by converting the allylation products to imatinib analogues, which showed antiproliferative activity against cancer cell lines (see section 3.5). The DAAA approach also worked well for the synthesis of ketopiperazines with tertiary stereocenters (up to 99% *ee*). More recently, the same catalytic system proved to be efficient in the DAAA of 1,4-diazepan-5-ones (*ee* values up to 95%; Scheme 216b).^590^ The use of a nonpolar solvent and the presence of a *p*-anisoyl lactam protecting group proved crucial to achieve such high enantioselectivities.

**Scheme 217:**
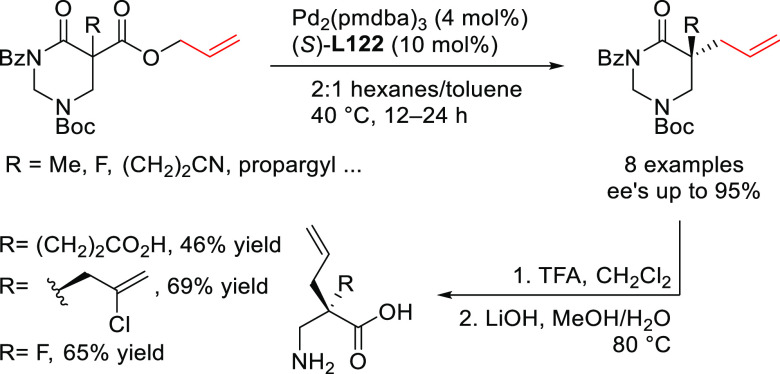
DAAA of *N*-Boc Tetrahydropyrimidin-2-ones

A limitation of this approach was the 5-step sequence required to access the required substrates. This low-yielding route also limited the substrate scope because of the unwanted side reactions. These issues were addressed by the Stoltz group in a recent publication reporting a new 3-step sequence beginning from commercially available 1-Boc-3-oxopiperazine.^591^ As part of this work, the application of an isomeric substrate class, *N*-Boc tetrahydropyrimidin-2-ones was described (Scheme 217). The desired α-quaternary products were formed in excellent yields and *ee* values of up to 95%. These products were then hydrolyzed to give valuable β^2,2^-amino acids (Scheme 217).

**Scheme 218:**
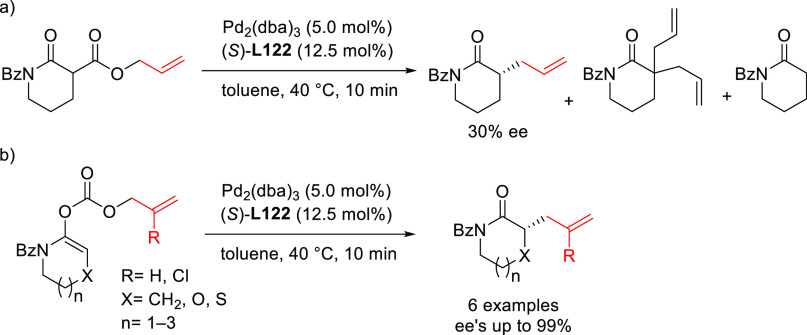
β-Amido Esters vs Enol Carbonates in the DAAA Forming Tertiary Stereocenters

Stoltz extended the DAAA scope to the enantioselective preparation of lactams containing α-allyl tertiary-substituted stereocenters (Scheme 218).^592^ β-Amido esters were first tested as catalytic substrates but led to low enantioselectivities and yields because of the unwanted synthesis of side products (diallylated and unallylated lactams). Their formation and the low *ee* of β-amido esters were proposed to be due to the scrambling of the α-proton (Scheme 218a). These problems were overcome when the substrate was changed to the related enol carbonates, which afforded the required α-allylated lactams in high yields and enantioselectivities (up to 99% *ee*; Scheme 218b).

**Scheme 219:**
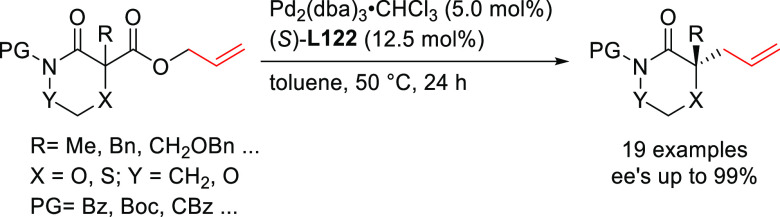
DAAA of N,S- and N,O-Heterocycles

Furthermore, Stoltz broadened the DAAA scope to N,S- and N,O-containing heterocycles (Scheme 219).^593^ Excellent enantioselectivities were observed with morpholinone and oxazolidin-4-one substrates (up to 99% *ee*) whereas analogous thiomorpholinones provided a slightly lower *ee* values (X = S; Y = C; R = Me; 86% *ee*). Hydroxamic acid derivatives (X = C, Y = O, R = Me) also furnished α-allylated products in modest to very high levels of enantioselectivity (72–93% *ee*). The products were subsequently transformed into morpholine, α-tertiary hydroxyl ester, and α-quaternary δ-lactone analogues.

**Scheme 220:**
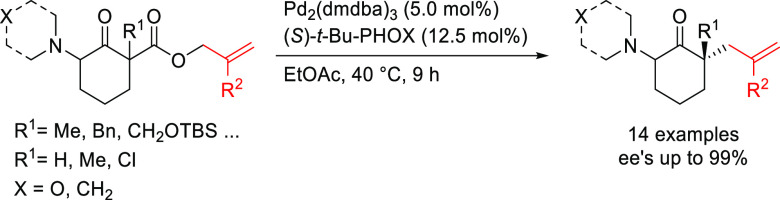
Pd-Catalyzed DAAA of α-Enaminones

Stoltz reported the Pd-catalyzed DAAA of α-enaminones (Scheme 220).^594^ The use of the Pd/(*S*)-*t*-Bu-PHOX catalyst led to the synthesis of valuable enantioenriched enaminone derivatives bearing an all-carbon quaternary stereocenter, which are competent precursors for a range of postalkylation transformations.

**Scheme 221:**
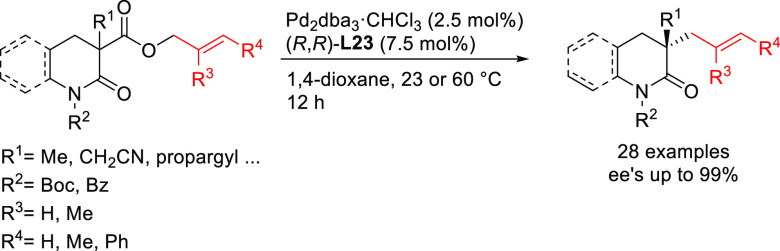
DAAA of Dihydroquinolones

Trost recently described the DAAA of dihydroquinolinones to afford α-allylated derivatives (Scheme 221).^595^ Although Stoltz had previously shown that PHOX-type ligands are effective in the DAAA of simple lactams, Trost chose to screen a variety of his own ligands as they generally show broader scope in terms of both the electrophile and nucleophile. The optimal ligand was found to be Trost ligand (*R*,*R*)-**L23**, giving high yields and enantioselectivities (up to 98% *ee*). With simple δ-valerolactams, yields of up to 99% and *ee* values of up to 93% were achieved. In some cases, increasing the reaction temperature from 23 to 60 °C improved both the yield and the enantioselectivity.

**Scheme 222:**
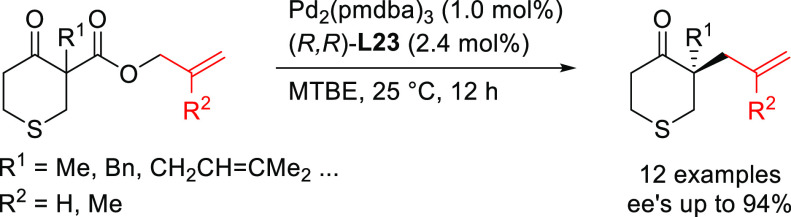
DAAA of Thiopyranone Derivatives

Thiopyranone derivatives were also investigated as substrates in DAAA reactions by Stoltz (Scheme 222).^596^ Employing traditional enolate chemistry to generate α-quaternary derivatives of 4-thiopyranones can prove difficult because of the tendency of these heterocycles to afford ring-opened sulfur alkylation products and the inherent reactivity of the β-disposed sulfur.^597^ The synthesis of α-quaternary 4-thiopyranones via DAAA was demonstrated in good to high levels of enantioselectivity (50–94% *ee*) employing Trost’s ligand (*R,R*)-**L23**. Although not reported, the key feature of this approach was the potential to prepare acyclic ketones possessing quaternary stereocenters because of the facile reductive cleavage of the C–S bond to form the acyclic product. As we will see, Stoltz ultimately did not have to resort to this two-step “work-around” to form acyclic ketones possessing quaternary stereocenters in a subsequent report in 2018 (vide infra).

**Scheme 223:**
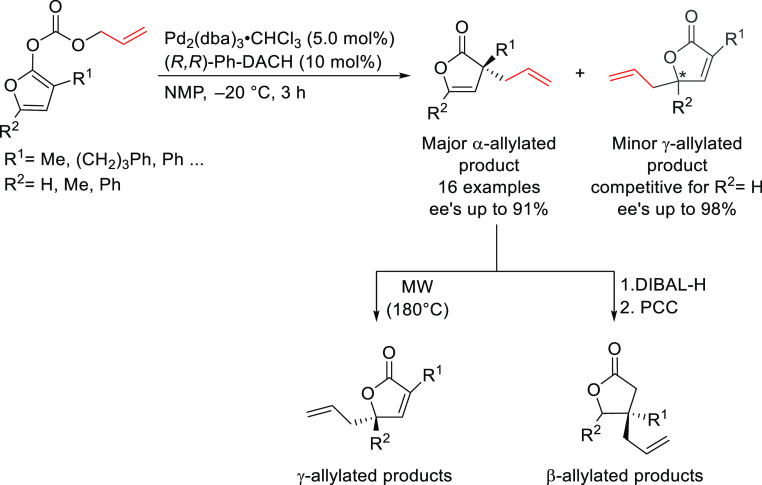
DAAA of Cyclic Dienol Carbonates

In 2013, Cossy broadened the DAAA to γ-butenolides employing cyclic dienol carbonates (Scheme 223).^598^*C*_2_-symmetric diphosphine ligands afforded the desired α-allylated products in high *ee* values, with the Trost ligand (*R,R*)-Ph-DACH giving the highest levels of asymmetric induction. During the optimization process, a significant difference in enantioselectivity between the α- and the γ-allylated products was noted, regardless of the solvent employed. For example, in toluene, the γ-allylated product was generated in 98% *ee*, whereas the α-allylated product was accessed in just 40% *ee* for the model substrate allyl (3-(3-phenylpropyl)furan-2-yl) carbonate (R^1^ = (CH_2_)_3_Ph and R^2^ = H). This finding supported the proposal that the γ-allylated product resulted from a competitive allylation rather than a [3,3]-sigmatropic rearrangement. α-Allylated products predominated in excellent yields and enantioselectivity (up to 91% *ee*) under the optimized reaction conditions which used *N*-methyl-2-pyrrolidone (NMP) as solvent. A decrease in regioselectivities (α/γ = 3/1) were obtained for substrates containing *ortho-*substituted aryl groups resulting in the formation of α-allylated products in diminished yields. α,α-Disubstituted butenolides, formed by the DAAA transformation, were subsequently converted via a microwave-assisted Cope rearrangement to allylated products possessing either a γ-quaternary or γ-tertiary stereocenter without loss of enantiopurity. Reduction by DIBAL, and then oxidation by PCC, led to β-quaternary butyrolactones, again with conservation of enantiopurity. Using the newly developed DAAA protocol, the total synthesis of (−)-nephrosteranic acid and (−)-roccellaric acid was accomplished (see section 3.5).

**Scheme 224:**
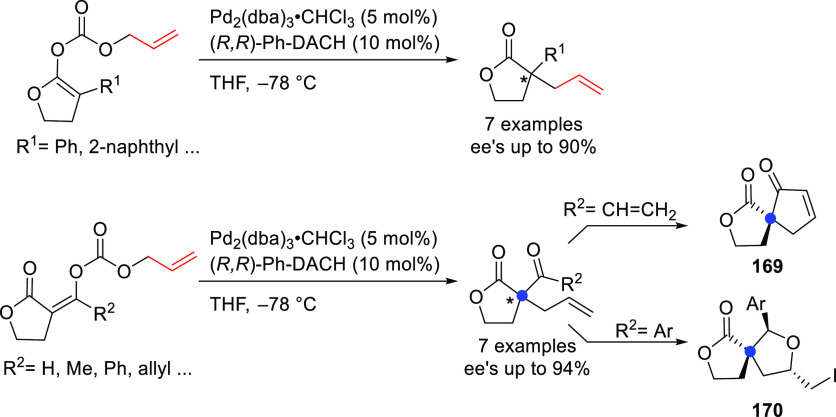
DAAA of γ-Butyrolactone-Derived Substrates

Subsequently, Cossy applied the Pd-catalyzed DAAA to enol carbonates, derived from γ-butyrolactones and α-acyl-γ-butyrolactones, to generate α,α′-disubstituted γ-butyrolactones in high yields and excellent levels of enantioselectivity (Scheme 224).^599^ The Trost ligand (*R,R*)-Ph-DACH proved to be optimal with these substrates inducing *ee* values of up to 94%. Notably, an intermolecular variant was studied in one case by generating the enolate in situ employing 3-benzoyldihydrofuran-2(3*H*)-one and a base and then reaction with allyl acetate. Yield and enantioselectivity of the allylated product were comparable to those obtained using the analogous allyl enol carbonate substrate. The synthesis of γ-butyrolactone-derived spirocycles **169** and **170** was reported by a ring-closing metathesis or a Luche reduction-iodocyclization approach, respectively.

**Scheme 225:**
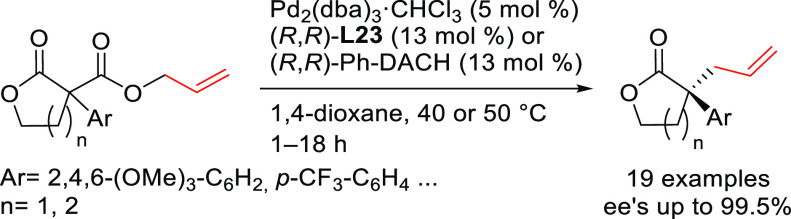
DAAA of Lactone-Derived Substrates

**Figure 25:**
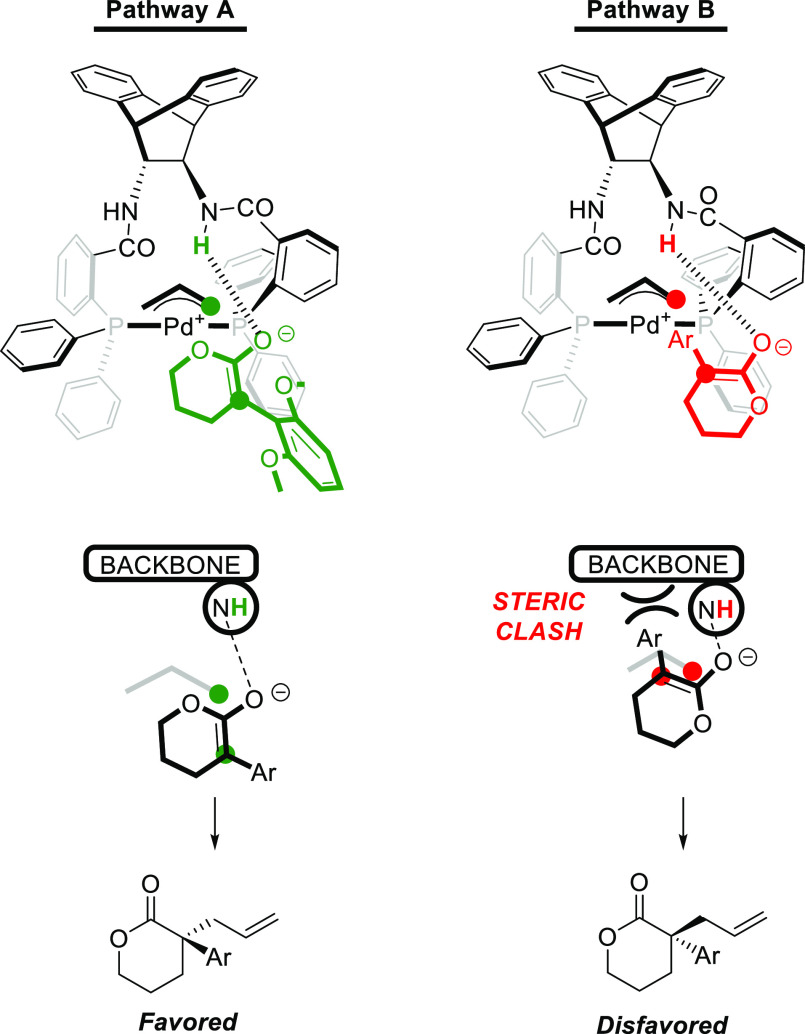
Explanation of the stereochemical outcome of the DAAA with lactone substrates.

Guiry and co-workers extended their previous work on Pd-catalyzed DAAA of sterically hindered α-aryl β-keto allyl ester substrates^570^ to include α-aryl β-oxo-allyl lactone derivatives (Scheme 225).^600^ Studies to optimize this process with dihydrocoumarin and δ-valerolactone-derived α-aryl β-oxo-allyl esters possessing a 2,4,6-trimethoxyphenyl substituent, found Trost’s ligand (*R,R*)-**L23** to be the optimal for these sterically hindered substrates. A wide range of substrates with different aryl substitutents were effectively used to give the corresponding α-allylated products. Substrates possessing sterically hindered aryl moieties, such as naphthyl or those containing di-*ortho*-substitutions afforded excellent levels of enantioselectivity (up to 99.5% *ee*). α-Allyl-α-aryl lactones of this type were not previously accessible by other methods. The authors attributed the observed excellent levels of enantioselectivity to the steric clash between the sterically hindered aryl group and the ligand scaffold. *Ortho*-substitution is proposed to prevent coplanarity and thus prohibits conjugation, resulting in an unstabilized enolate (Figure 25). A limitation of this strategy is the relatively moderate enantioselectivities obtained with substrates possessing aryl groups without *ortho*-substituents. Nevertheless, the (*S,S*)-DACH-phenyl ligand was superior for such less hindered substrates, including a substrate containing a *para-*trifluoromethylphenyl group (80% *ee*; Scheme 225).^600^

**Scheme 226:**
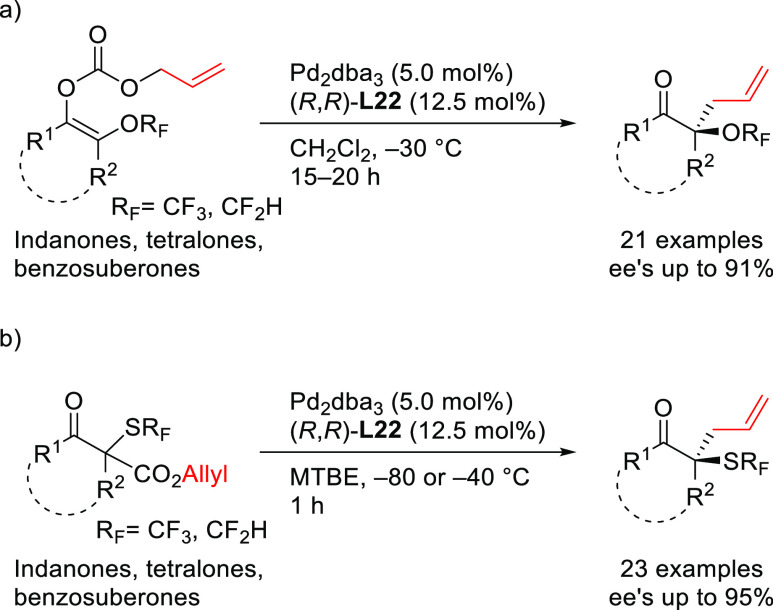
DAAA of Various Fluorine-Containing Substrates

Shibata and co-workers reported the synthesis of enantioenriched α-trifluoromethoxy ketones through DAAA using enol carbonates as substrates (Scheme 226). These are attractive targets because of the electron-withdrawing nature of the trifluoromethoxy moiety and its ability to increase the lipophilicity of compounds containing this functionality.^601^ Few examples of the enantioselective synthesis of trifluoromethoxy-containing compounds have been reported yet.^602^ After optimization, including a careful temperature study, the best reaction conditions were found to be CH_2_Cl_2_ with Trost’s ligand (*S*,*S*)-**L22** (Scheme 19) at −30 °C. The substrate scope comprised various indanones with substituents in the aromatic ring, tetralones and benzosuberones, which gave the corresponding allylated products in yields of up to 94% and *ee* values of up to 91% (Scheme 226a). Oxindoles, 4-chromanones and acyclic substrates were less suited, as the corresponding products were obtained in either low yields or with poor enantioselectivities.

Shortly after, the same group published related work on the DAAA of α-difluoromethylthio- and α-trifluoromethylthio-β-keto allyl esters (Scheme 226b).^603^ Compounds containing a trifluoromethylthio group display even higher lipophilicity than those possessing a trifluoromethoxy group. α-Difluoromethylthio- and α-trifluoromethylthio-β-keto allyl esters are prepared by electrophilic difluoromethylthiolation/trifluoromethylthiolation of the corresponding tertiary β-keto allyl esters. Again Pd/(*S*,*S*)-**L22** was found to be the optimal catalyst. When applied to a range of indanone-, tetralone-, and benzosuberone-containing substrates, the corresponding α-difluoromethylthio products were formed in yields of up to 99% and *ee* values of up to 94%. The analogous α-trifluoromethylthio products were obtained in yields of up to 96% and *ee* values of up to 95%. Again acyclic substrates proved difficult, giving only 19% yield and 33% *ee* in the case of the α-difluoromethylthio substrate. In contrast, the corresponding α-difluoromethylthio-oxindole exhibited improved reactivity (67% yield, 74% *ee*). It is noteworthy that these reactions were carried out at much lower temperatures than the typical reaction temperatures of other DAAA reactions. This may be attributed to the strong electron-withdrawing nature of the -SCF_3_ and -SCF_2_H groups, which is expected to accelerate decarboxylation and stabilize the resulting enolate. The absolute configuration of the products was determined using a combination of ECD spectra, UV spectra and computational methods.

**Scheme 227:**
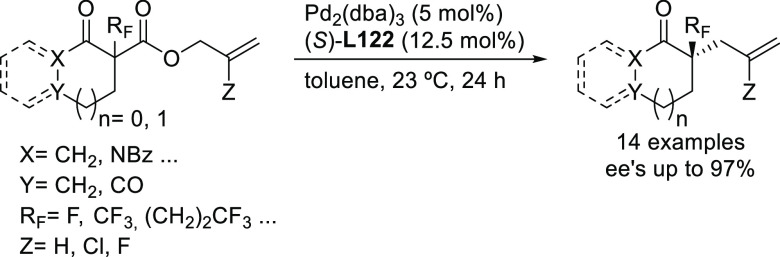
Synthesis of Chiral (Poly)fluorinated 5- and 6-Membered Ketones and Lactams

More recently, Stoltz’s group disclosed a general method for the synthesis of carbo- and heterocyclic carbonyl compounds bearing fluorinated α-substituted sterocenters via Pd-catalyzed DAAA (Scheme 227).^604^ The use of (*S*)-**L122** ligand proved to be crucial to afford 5- and 6-membered ketones and lactams bearing (poly)fluorinated tetrasubstituted chiral centers in high enantioselectivities (*ee* values up to 97%).

**Scheme 228:**
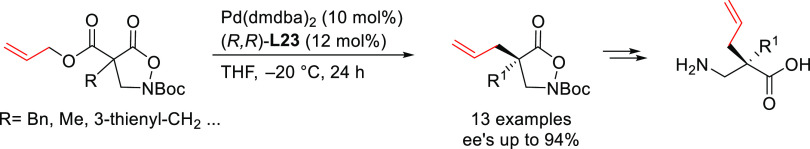
Preparation of Quaternary β^2,2^-Amino Acids Using DAAA

Shibasaki and Noda reported the preparation of 4-substituted isoxazolidin-5-ones by Pd-catalyzed DAAA (Scheme 228).^605^ Pd complexes of the Trost ligand (*R,R*)-**L23** afforded the allylated products possessing α-2-arylmethyl and α-benzyl substituents in very high yields and levels of enantioselectivity (up to 94% *ee*). They were subsequently transformed by cleaving the N–O bond into a range of β^2,2^-amino acids that were not accessible previously. In addition, the allylated products were demonstrated to be suitable substrates for a variety of transformations including an α-ketoacid-hydroxylamine ligation^606,607^ and Fmoc-based solid-phase peptide synthesis.

**Scheme 229:**
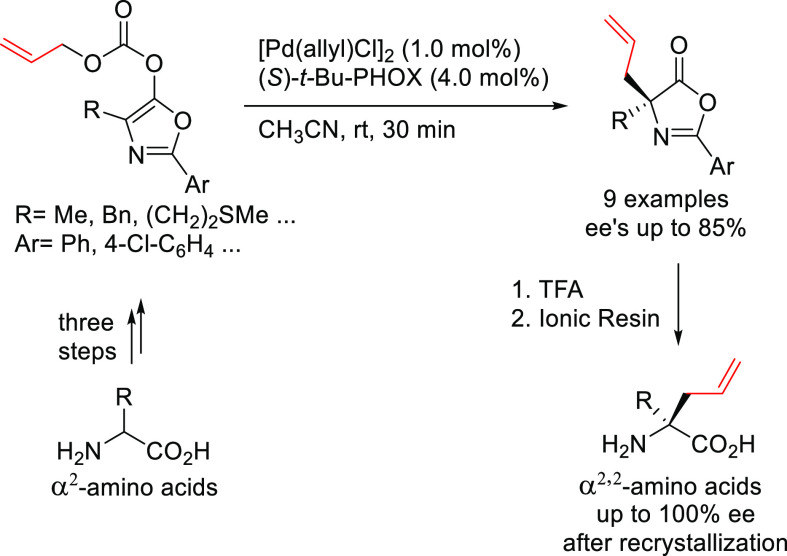
Formation of Quaternary α^2,2^-Amino Acids

Shibasaki, Noda, and later Stoltz utilized the DAAA reaction as the key enantiodetermining step in the development of quaternary β^2,2^-amino acids (Schemes 217 and 228). Colombo also applied this method to the enantioselective synthesis of quaternary α^2,2^-amino acids,^608^ using azlactone enol carbonates as substrates, which can be accessed in 33% to 89% overall yield from commercially available tertiary amino acids in three steps (Scheme 229). Among the range of ligands tested, Trost’s ligand (*R*,*R*)-Ph-DACH gave the highest enantioselectivity (70% *ee*) for the phenylglycine-derived model substrate. It was found that a too high catalyst loading was detrimental to the enantioselectivity, which was assumed to be due to oligomerization of the substrate-catalyst complex, as proposed by Lloyd-Jones and Norrby.^470^ Slow addition of the substrate as a solution via syringe pump improved the level of enantioselectivity (50 to 59% *ee*). In contrast, slow addition of the Pd-ligand complex to a solution of the substrate led to a massive drop in enantioselectivity to 4% *ee*. In general, the yields obtained were good (up to 98%), but with only moderate to high enantioselectivity (up to 85% *ee*). Following hydrolysis, the products could be recrystallized to highly enantioenriched α^2,2^-amino acids.

**Scheme 230:**
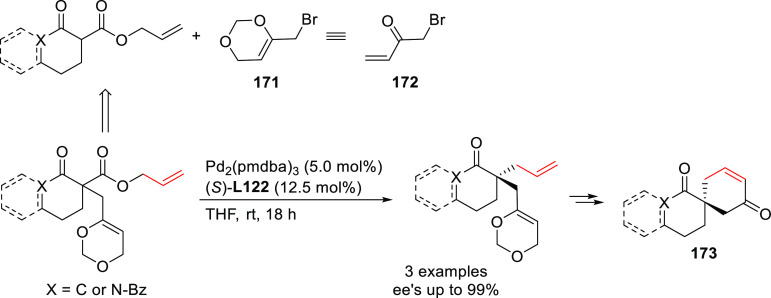
Synthesis of Spirocyclic Compounds by Stoltz Using DAAA

Stoltz and co-workers developed the DAAA of cyclic ketone substrates containing a masked methyl vinyl ketone at the α-position (Scheme 230).^609^ The dioxin unit in **171** was employed as a surrogate for bromomethyl vinyl ketone **172** to overcome problems associated with nucleophilic addition to **172**. The Pd catalyst derived from *p*-(CF_3_)_3_-*t*-Bu-PHOX ligand **L122** (Scheme 208) enabled the preparation of α-allylated products with high enantioselectivities (up to 99% *ee*). The important spirocyclic frameworks **173**, containing both an all carbon quaternary stereocenter and a 1,4-dicarbonyl unit, were subsequently synthesized by ring closing metathesis once the dioxin unit was unmasked under thermal conditions.

**Scheme 231:**
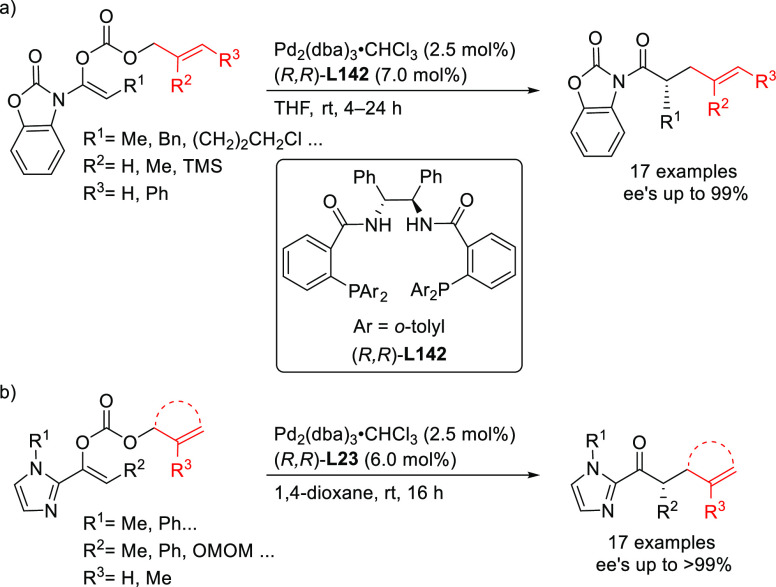
DAAA of (a) Benzoxazolinone and (b) 2-Acylimidazole Derivatives

The DAAA of benzoxazolinone-based allyl enol carbonates was reported by Trost in 2012 (Scheme 231a).^610^ A number of new stilbene diamine-derived Trost ligand complexes were evaluated for this reaction. They all showed high activity, with the catalyst derived from *ortho*-tolyl Trost ligand **L142** being optimal. In a broad substrate screen, excellent enantioselectivities (up to 99% *ee*) were observed for a wide range of enol carbonates substituted at the allylic, internal and terminal positions of the allyl unit. Importantly, the allylated *N-*acetyloxazolinones could be converted into carboxylic acid, ester, thioester and amide derivatives under mild conditions without loss of enantiopurity. The same group made use of the Pd/(*R*,*R*)-**L23** catalyst for the synthesis of highly enantioenriched 2-acylimidazoles from 2-imidazolo-substituted enol carbonates (*ee* values up to >99%; Scheme 231b).^611^

**Scheme 232:**
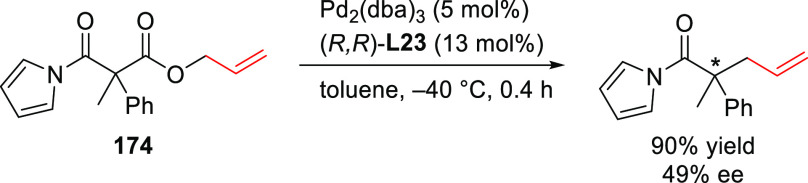
DAAA of Amide Enolates

Tunge developed the DAAA of acyclic β-ketoesters and β-ketoamides.^612^ This transformation for substrate **174** afforded excellent yields but enantioselectivities were moderate with a maximum of 49% *ee* (Scheme 232). The authors proposed that this was because of the formation of the (*E*)- and (*Z*)-enolate without bias. The transformation was also performed with a chiral auxiliary and an achiral ligand, which afforded a maximum diastereoselectivity of only 5.6/1. However, this represented the best selectivity observed for acyclic β-oxo esters at that time.

**Scheme 233:**
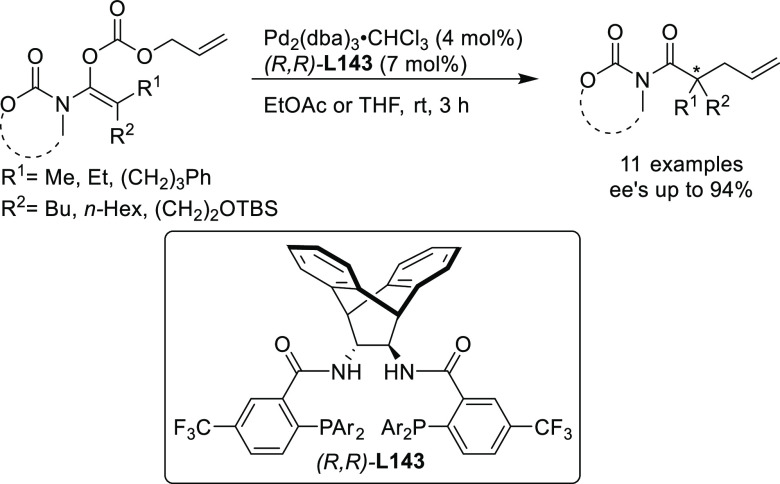
DAAA of Amide Enolates

Benzoxazolinones and a range of acyclic polysubstituted allyloxycarbonyl amide enolates were studied in a collaboration between the Stoltz and Marek groups in 2017 (Scheme 233).^613^ On the basis of their prior work on the DAAA of lactam enolates, they proposed that the stereoelectronic features of the amido group would be important for the success of the reaction. The electron-deficient *C*_*2*_*-*symmetric bisphosphine Trost ligand **L143** afforded the optimal *ee* of 94% for the model substrate studied. The enantioselectivities were generally high for the substrates investigated, showing the broad functional group tolerance of the DAAA. Only moderate yields were obtained in some cases, which was attributed to side reactions, such as enolate protonation and β-hydride elimination, or steric congestion.

**Scheme 234:**
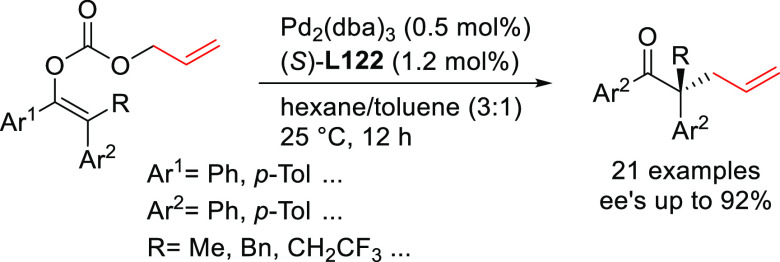
DAAA of Acyclic Enol Carbonates

While cyclic substrates have been extensively studied in Pd-catalyzed DAAA giving rise to α-quaternary stereocenters, acyclic systems, which are less rigid, have been much less explored. Stoltz and Zhang have recently described the DAAA of fully substituted acyclic enol carbonates (Scheme 234).^614^ The electron-deficient *p*-(CF_3_)_3_-*t*-Bu-PHOX ligand **L122** (see Scheme 205 for ligand structure) proved optimal, providing the linear α-quaternary ketone products with high yields and levels of enantioselectivity (up to 92% *ee*). Even though allyl enol carbonates could be generated with high *E*/*Z* selectivity by enolization of the acyclic ketones and trapping of the resultant enolates, the *E/Z* ratio surprisingly proved to be not critical as both isomers afforded the same product enantiomer with almost identical *ee* values. Racemic allyl β-ketoesters as well could be used as substrates giving the α-allylated products with the same high *ee* values as the corresponding enolate carbonates. A dynamic kinetic resolution with ligand **L122**, through equilibration between *C-*bound and *O-*bound Pd-enolates, was suggested to explain these results. Similarly, Stoltz’s group also reported the highly efficient Pd-catalyzed DAAA of protected benzoin-derived enol carbonates using Pd/(*R,R*)-**L23** catalyst (*ee* values up to 88%).^615^

**Scheme 235:**
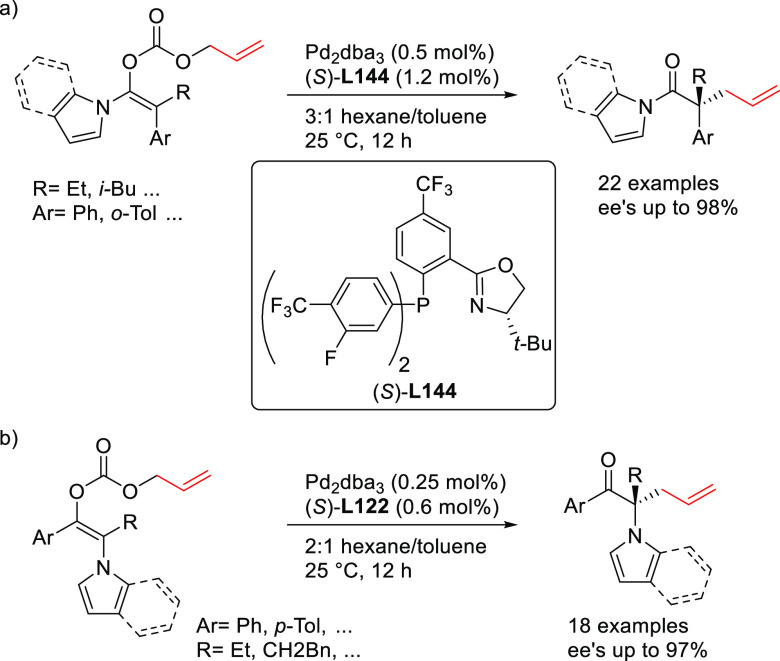
DAAA of Fully Substituted (a) *N*-Acyl Indole-Derived Enol Carbonates and (b) α-*N*-Pyrrolyl/indolyl Enol Carbonates

Stoltz and Zhang sought to expand the scope of the DAAA of acyclic enol carbonates to include ester enolates as such substrates offer a route to synthetically versatile α-quaternary carboxylic acids.^616^ Previous highly successful work by Trost’s group on ester enolate equivalents has been limited to trisubstituted enolates (Scheme 231).^610^ Several tetrasubstituted enol carbonate substrates were synthesized from esters in high *E/Z* selectivity, however these compounds were found to be extremely poor substrates. Stoltz and Zhang examined ester enolate equivalents to address this issue. A range of *N*-acyl hetereocycles was synthesized, with *N*-acyl indole found to be the optimal ester equivalent which could then be enolized with high *E/Z* selectivity. When this substrate was applied in the DAAA the product was formed in 95% yield with 90% *ee*. Substituting the (CF_3_)_3_-*t*-Bu-PHOX ligand **L122** (Scheme 205) for the novel ligand (*S*)-Ty-PHOX **L144** led to an improved yield of 99% with an *ee* of 95% (Scheme 235a). In contrast to previous work with ketone enolates, the *E/Z* ratio of the *N-*acyl enolates was found to have a significant effect on the stereochemical outcome. The use of a 21:79 *E*/*Z* mixture reduced the *ee* from 95% to 66%. The major enantiomer formed was still the same as when a 98:2 *E*/*Z* mixture was used. This indicates that there is still some degree of dynamic kinetic resolution occurring as with ketone enolates, albeit to a lesser extent. Yields and enantioselectivities for a range of α-aryl groups were generally excellent, with a *p*-tolyl group providing the product in a 99% yield with an *ee* of 98%. A range of aryl substitution patterns were well tolerated, except a strongly withdrawing *p*-CF_3_ which led to an *ee* of 72%. Also, sterically demanding *ortho*-substitution (mono *o*-Me and mono *o*-Br) required the use of the smaller *N*-acyl 3-methyl pyrrole to give the products with satisfactory *ee* values (89% and 80%, respectively). More recently, Stoltz’s group reported on the efficient synthesis of fully substituted acyclic α-*N*-pyrrolyl/indolyl ketones via Pd-catalyzed DAAA using ligand (*S*)-**L122** (Scheme 235b).^617^

**Figure 26:**
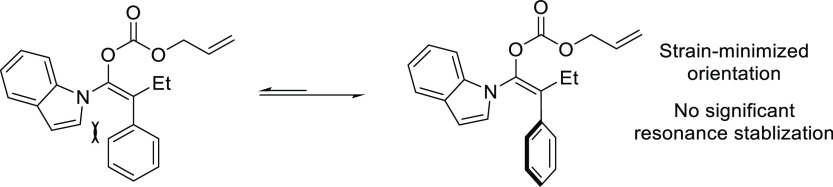
Rationalization of the unusually high enantioselectivity observed for α-aryl-containing substrates with PHOX-type ligands in this system.

The fact that a PHOX-type ligand was so successful for substrates possessing a range of α-aryl groups was surprising. α-Aryl groups stabilize enolates and stabilized enolates are known to react with lower levels of enantioselectivity when using PHOX-type ligands.^618,619^ The authors proposed an explanation for the high enantioselectivity observed in this case. They postulated that the α-aryl group rotates out of plane relative to the enolate to avoid a steric clash with the indole group, which remains in plane and in conjugation with the enolate π-system (Figure 26). A stabilizing edge-to-face interaction between the indole and aryl group may also play a role. In this orientation the α-aryl ring does not contribute significant resonance stabilization to the enolate, and therefore, high enantioselectivity can be achieved.

**Scheme 236:**
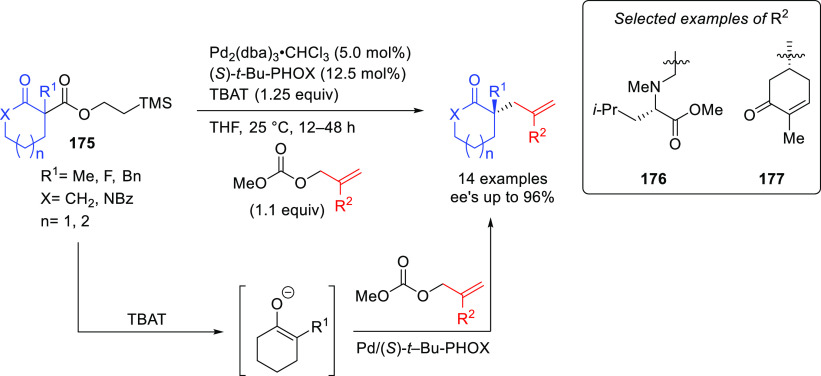
Pd-Catalyzed Enolate Alkylation Cascade

A novel method of in situ generation of the enolate was reported by Stoltz and co-workers (Scheme 236).^620,621^ They showed that a TMS-ethyl ester of type **175** can undergo desilylation affording the enolate through extrusion of ethylene and CO_2_. Initial studies had a focus on substrates containing six- and seven-membered ring lactams and ketones. A major benefit of this approach is that it leads to a wider variation of substituents on the allyl unit compared to traditional β-keto allyl esters. Notably, it enabled the preparation of DAAA products with allyl units containing sensitive functionalities and/or stereocenters (**176** and **177**) that lead to epimerization under the base-mediated reaction conditions employed for substrate synthesis.

**Scheme 237:**
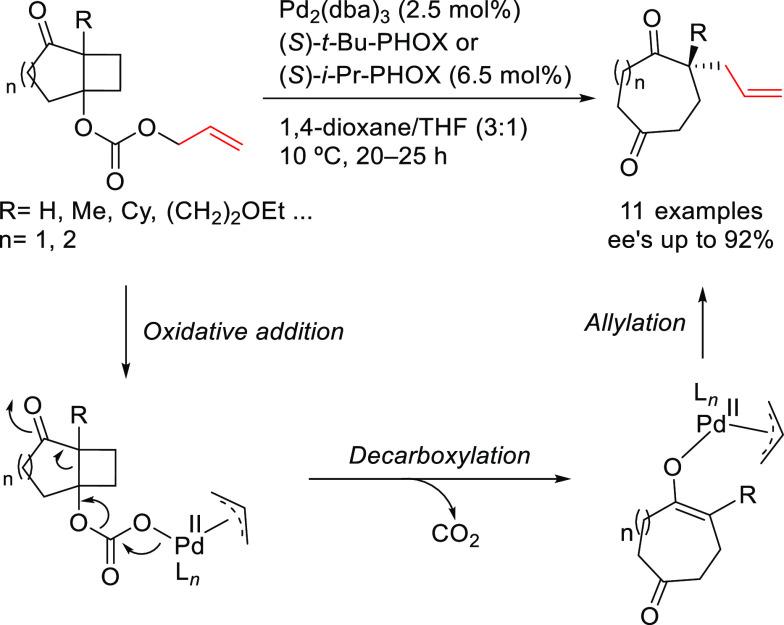
Blechert’s Asymmetric Ring-Expanding Allylation (AREA)

Schulz and Blechert developed a variation of the DAAA, which they referred to as an “asymmetric ring-expanding allylation” (AREA).^622^ By this reaction, they prepared α,α′-disubstituted cycloheptane-1,4-diones and cyclooctane-1,5-diones from allyl carbonates derived from from bicyclo[3.2.0]heptane-2-ones using Pd complexes of chiral PHOX ligands (*S*)-*t*-Bu-PHOX and (*S*)-*i-*Pr-PHOX as catalysts (Scheme 237). These strained substrates were readily synthesized by *O-*alkylation of β-diketones followed by photoinduced [2 + 2] cycloaddition. The α-quaternary cycloheptane-1,4-dione products were formed in high yields and levels of enantioselectivity, with the Pd complex of (*S*)-*t*-Bu-PHOX affording the optimal results (93% yield, 92% *ee*). For AREA reactions generating tertiary α-allyl products (R= H), the Pd complex of (*S*)-*i*-Pr-PHOX was superior to (*S*)-*t*-Bu-PHOX, providing moderate enantioselectivities in the 41–73% *ee* range. The mechanism of this reaction is proposed to proceed by an oxidative addition of Pd into the C–O bond of the substrate, followed by decarboxylation. The resulting alkoxide intermediate reacts by ring-expansion via a retro-aldol transformation to yield a Pd allyl-enolate complex, which then undergoes intramolecular allylation to yield the final product (Scheme 237).

### 3.2 Decarboxylative Allylation of Imines and Nitro Compounds

**Scheme 238:**
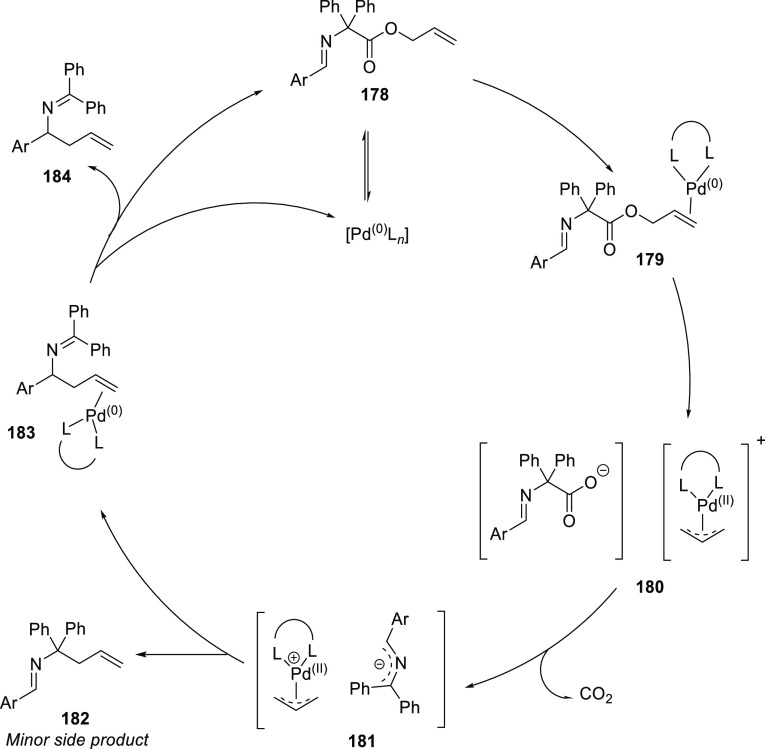
Proposed Mechanism for the Pd-Catalyzed Decarboxylative Allylation of Imines

The DAAA approach has also been extended to the enantioselective synthesis of allylated imines and nitro compounds. In 2014, Chruma prepared 2-azaallyl anions via decarboxylation and subsequent enantioselective allylation to form α-aryl homoallylic imines.^623^ Previously, Tunge and Chruma had independently developed the DAAA of α-imino allyl ester substrates affording homoallylic imine products in good yields.^624−627^ Moderate enantioselectivity of 30% *ee* could be achieved in one case with Pd/(*R*)-BINAP as catalyst.^624^ On the basis of the computational studies, Chruma and Fu proposed the DAAA of allyl α-imino esters **178** to occur by an oxidative addition, decarboxylation and reductive allylation series of transformations (Scheme 238), analogous to the Pd-catalyzed DAAA of enolates.^628^ The rate-determining step was the decarboxylation of the solvent separated ion-pair **180**. An outer sphere attack of the 2-azaallyl anions onto the Pd η^3^-allylcomplex **181** was proposed to be the regio- and enantio-differentiating step.

**Scheme 239:**
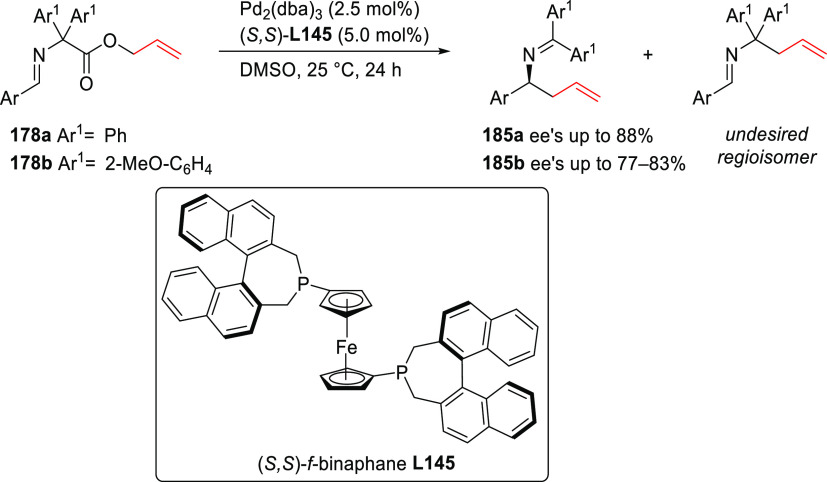
Pd-Catalyzed DAAA of Imines

For the asymmetric variant, testing of a series of chiral bi- and monodentate ligands showed that Pd complexes of the chiral ferrocenyl binaphene ligand **L145** gave the best results, affording enantioenriched α-aryl homoallylic imines **185** with moderate to high *ee* values up to 88% (Scheme 239). A trend toward lower *ee* values was observed for aryl amines with electron-donating substituents, while changing the solvent from THF to DMF or DMSO led to an overall increase in enantioselectivity. Later on, Chruma reported a positive linear Hammett correlation between the electronic parameters of the *para*-substituted benzaldimine and the regio- and enantioselectivities.^629^ Switching to the 2,2-di(2-methoxyphenyl)-glycine derived substrate **178b** led to increased yields and higher regioselectivities in favor of product **185b**, but slightly lowered (by 5–11%) enantioselectivities.

**Scheme 240:**
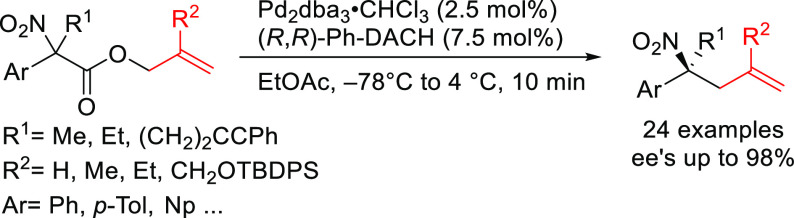
DAAA of α-Nitroesters

Until recently, examples of asymmetric *C*-allylation of secondary benzylic nitronates were scarce. The only case, in which the nucleophile was not stabilized by an ester group and the sterogenic center was formed α to the nitro group rather than in the allyl fragment, was reported by Shibasaki in 2007.^630^ However, only modest enantioselectivites were achieved.^631^ The generally unsatisfactory results obtained with this type of substrate are likely due to the propensity for *O*-allylation. More promising results were recently published by Trost and co-workers. After an initial study of the intermolecular Pd-catalyzed allylic alkylation of α-secondary benzylic nitroalkanes with allyl methyl carbonate, which afforded the desired C-allylated products, but only in moderate *ee* values,^632^ they turned their attention to the corresponding decarboxylative variant.^633^ They found that both the chiral ligand and the solvent had a significant effect on the ratio of *C*- versus *O*-allylation. The Trost ligand (*R*,*R*)-Ph-DACH and EtOAc as solvent proved to be optimal (Scheme 240). Additionally, cooling the reaction to −78 °C and allowing it to warm to 4 °C led to an improved 87% *ee* for the model substrate with a yield also of 87%. A range of aromatic groups with differing electronic properties were tolerated, as were α-ethyl nitroesters and bulky β-siloxymethallyl esters, forming α-allylated benzylic nitro compounds in up to 99% yield and up to 98% *ee*.^632^

**Figure 27:**
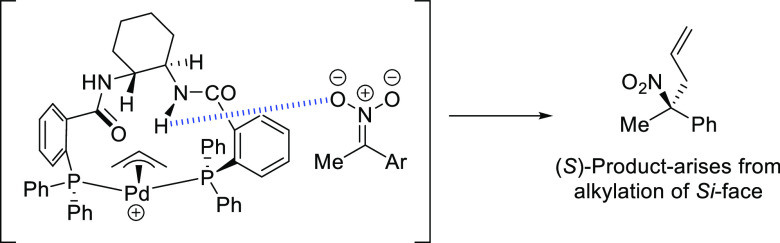
Proposed transition state for the DAAA of α-nitroesters based on the Lloyd-Jones/Norrby model.

On the basis of the findings of Lloyd-Jones and Norrby, the authors proposed a transition state in which hydrogen-bonding between one of the ligand NH groups and the nitronate accelerates the reaction and controls the approach of the nucleophile such that *C*-allylation becomes favored over *O*-allylation (Figure 27).

### 3.3 Mechanistic Aspects

**Scheme 241:**
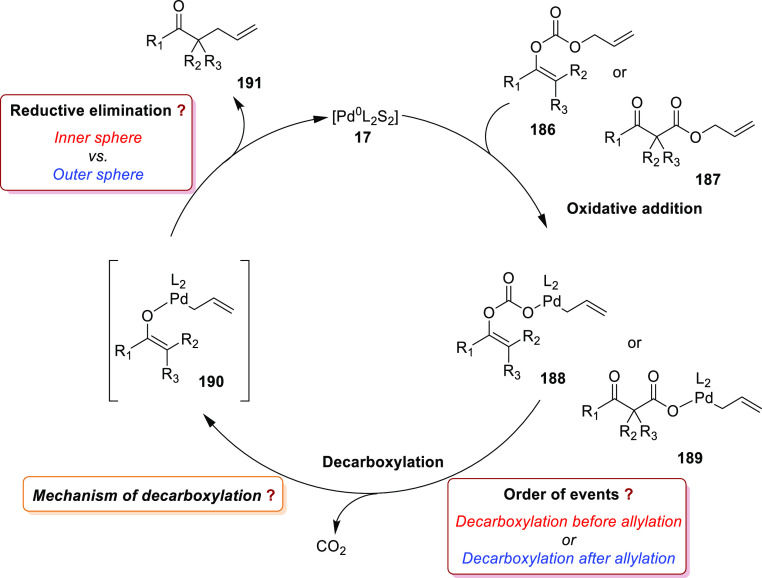
Proposed Catalytic Cycle for DAAA

The commonly accepted starting point for the DAAA mechanism is the coordination of the Pd^0^ complex **17** to the allyl fragment of either the enol carbonate **186** or the β-keto allyl ester **187** leading to oxidative addition. Subsequent loss of CO_2_ generates the Pd-enolate **190**, which undergoes reductive elimination leading to product **191** and regenerating the Pd^0^ catalyst (Scheme 241).^634^ While this is the most commonly accepted catalytic cycle for the DAAA, mechanistic studies, carried out primarily on Pd catalysts based on Trost-type or PHOX ligands, unveil a more complex and nuanced picture, in particular of the enantioselective step. The type of substrate and its substitution pattern, solvents and additives have also shown a significant impact on the mechanism. This section summarizes the mechanistic studies performed to date on the Pd-catalyzed DAAA and offer some general trends.

**Scheme 242:**
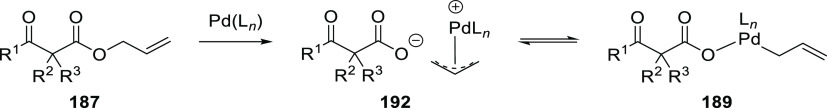
Pd-Induced Ionization

**Figure 28:**
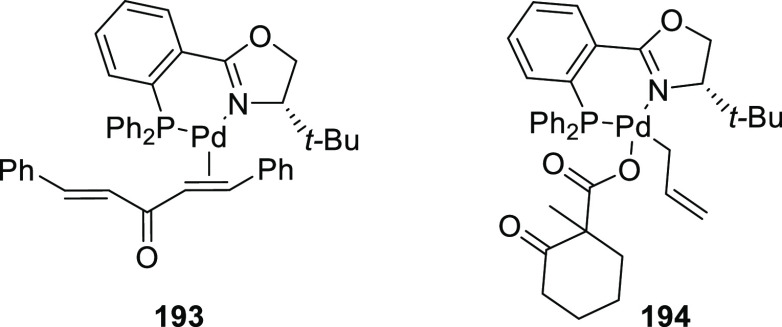
Intermediates characterized by X-ray crystallography.

Despite reports of different mechanistic pathways, there is at least general agreement on the first step of the catalytic cycle. Analogous to the Tsuji-Trost reaction of allyl acetates, the catalytic cycle begins with ionization of the carbonate or the ester-leaving group promoted by Pd. The Pd η^3^-allyl carboxylate ion pair **192** is thought to exist in equilibrium with the Pd η^1^-allyl complex **189** (Scheme 242). X-ray crystal structures of both the Pd complex **193** and the first intermediate **194** formed after oxidative addition were reported by Stoltz (Figure 28).^618,635^ The Pd η^1^-allyl-β-ketocarboxylate **194** was proposed to be the resting state using a PHOX ligand, which suggested that decarboxylation was the rate-determining step.

**Scheme 243:**
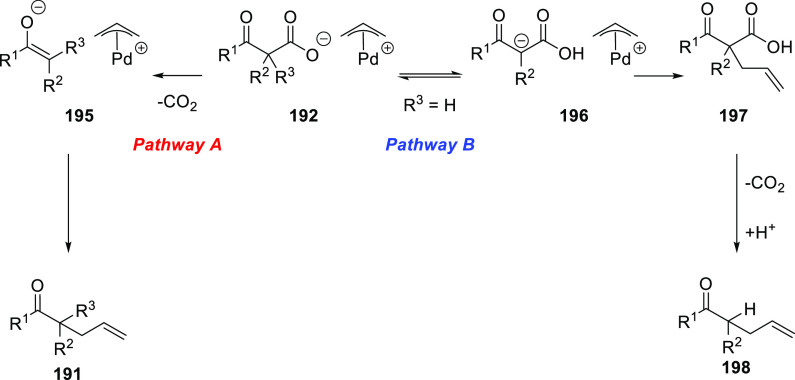
Mechanistic Pathways Proposed by Tunge Explaining the Observed Divergence of Stereoselectivity for Differently Substituted Substrates

For α,α-disubstituted esters, it can be deduced that decarboxylation forms the reactive nucleophile. However, the situation is less clear for enol carbonates and for substrates having an α-hydrogen. As it is generally accepted that decarboxylation proceeds for allyl enol carbonates more readily than for β-ketoesters, the majority of authors agree that decarboxylation happens before allylation for the former substrates without definitive supporting experimental evidence. Tunge performed a series of mechanistic investigations on α-protio substrates which showed that allylation happens before the decarboxylation step.^636^ With dihydrocoumarins as the substrates and employing an achiral catalyst, they found that substituent variation at the α-position (α-protio vs α-methyl) gave rise to divergent stereoselectivity. Therefore, they proposed that this stereodivergence results from two alternative mechanistic pathways. For substrates having an α-alkyl group, an initial decarboxylation leads to the formation of the Pd-enolate **195**, followed by allylation to form the product **191** (Scheme 243, pathway A). If the substrate contains an α-hydrogen, a proton transfer can take place to generate the stabilized carbanion **196**, which can undergo allylation and then decarboxylation of the β-ketoacid **197** to form the product **198** (Scheme 243, pathway B). They monitored the reaction by ^1^H NMR spectroscopy, which showed that a carboxylic acid appeared and then disappeared. For substrates possessing an α-hydrogen, it is possible to undergo decarboxylation, followed by allylation, and Tunge concludes that pathway **B** is supported by empirical evidence.^637^ Importantly, such divergent mechanistic pathways denote different enantioselectivity-determining steps: in pathway **A**, the configuration at the α-position is governed in the allylation step, whereas in pathway **B**, the configuration is determined in the protonation step, which proceeds post decarboxylation (Scheme 243).

**Figure 29:**
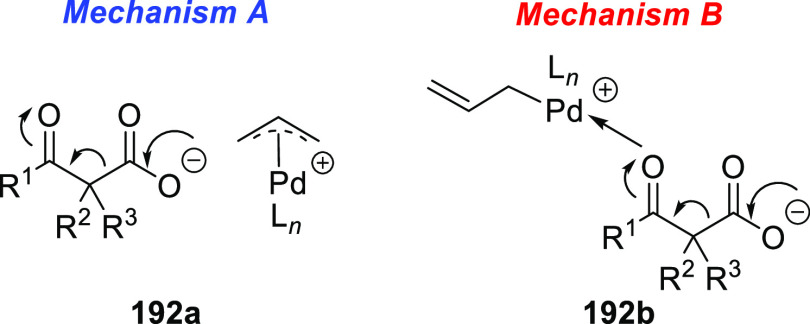
Plausible mechanistic pathways for decarboxylation.

The precise mechanism of decarboxylation, the step that leads to the formation of the nucleophile and thought to be rate-limiting, is one of the less studied aspects of the DAAA. Saegusa showed that the Pd catalyst plays an essential role in decarboxylation of sodium β-ketocarboxylate.^560^ Several decarboxylation mechanisms can be inferred taking into account the established mechanisms of decarboxylations with “soft” metals.^638^ Ionization of metal carboxylates, which has been proposed to proceed readily with Pd, is assumed to boost decarboxylation (Figure 29, pathway A).^638,639^ Coordination of Pd to the keto group also facilitates decarboxylation (Figure 29, pathway **B**).^638,640^ Tunge has outlined in considerable detail the plausible pathways for the decarboxylation step of allyl enol carbonates and β-ketocarboxylates, with a conclusion that “assessment of the actual decarboxylation mechanism will require more detailed experimentation”.^637^

**Figure 30:**
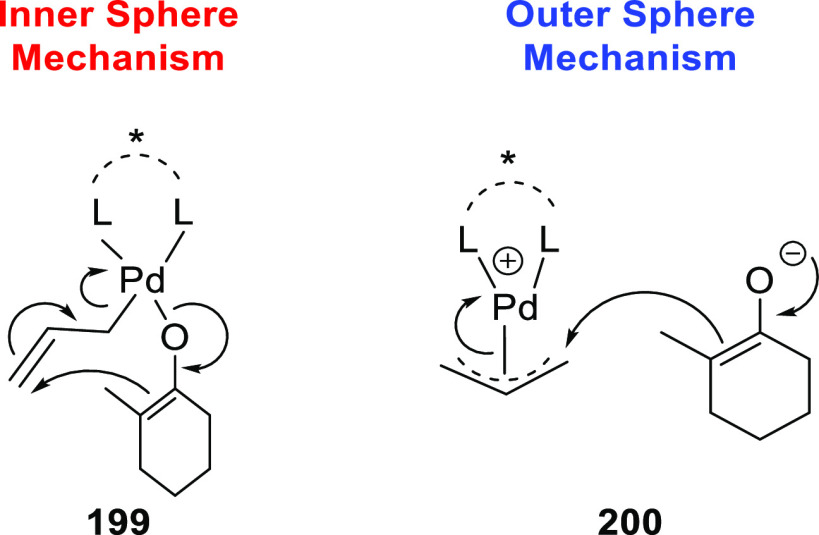
Postulated inner sphere vs outer sphere mechanisms.

The key discussion point for the reductive elimination step has been whether it occurs via an “inner-sphere” or an “outer-sphere” mechanism (Figure 30). It has been established that nonstabilized carbon nucleophiles (p*Ka* > 20) favor an “inner-sphere” pathway, while stabilized carbon nucleophiles (p*Ka* < 20) favor an “outer-sphere” pathway. Stoltz, Goddard, and co-workers performed a DFT study of a Pd-PHOX catalyzed DAAA reaction in 2007,^641^ which supported a concerted inner-sphere mechanism involving a four-coordinate Pd η^η^-allyl enolate complex **199** that directly forms the product through a seven-membered transition state (Figure 30).

**Scheme 244:**
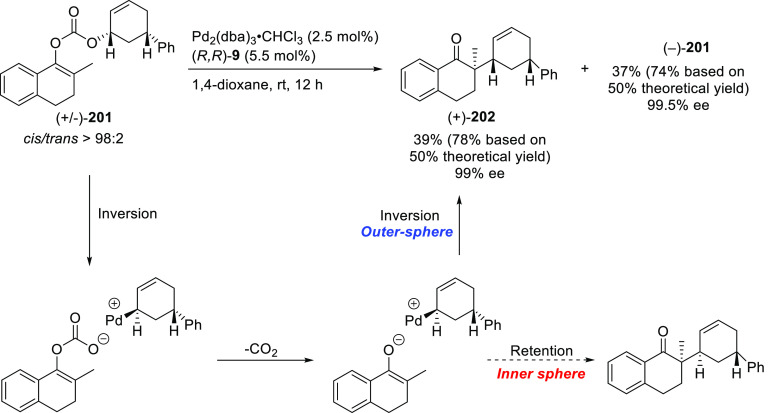
Stereochemical Probes Employed by Trost in DAAA

Trost and co-workers carried out extensive mechanistic investigations on the DAAA of enol carbonates.^634^ In the reaction of substrate **201**, they noted an initial increase in enantioselectivity with decreasing reaction temperature, followed by a decrease in *ee* below −10 °C. They also recorded significant differences between the AAA and the DAAA in a comparative study of tetralone-derived metal enolates and allyl enol carbonates. While the AAA afforded 98% *ee* the DAAA gave only 4% ee. Mechanistic investigations employing stereochemical probes were then performed to distinguish between an outer-sphere or inner-sphere process (Scheme 244). In the DAAA reaction of substrate **201**, kinetic resolution was observed. While one enantiomer showed no reaction after 12 h, the other enantiomer was transformed into a single diastereomeric allylated product **202** in 39% yield and 99% *ee*. On the basis of these findings, a double-inversion mechanism was proposed with retention of configuration at the substituted allyl group, implying an outer-sphere process. This result was inconsistent with the inner-sphere mechanism postulated by Stoltz, which may be the result of the Trost and Stoltz groups using different ligand classes.

**Scheme 245:**
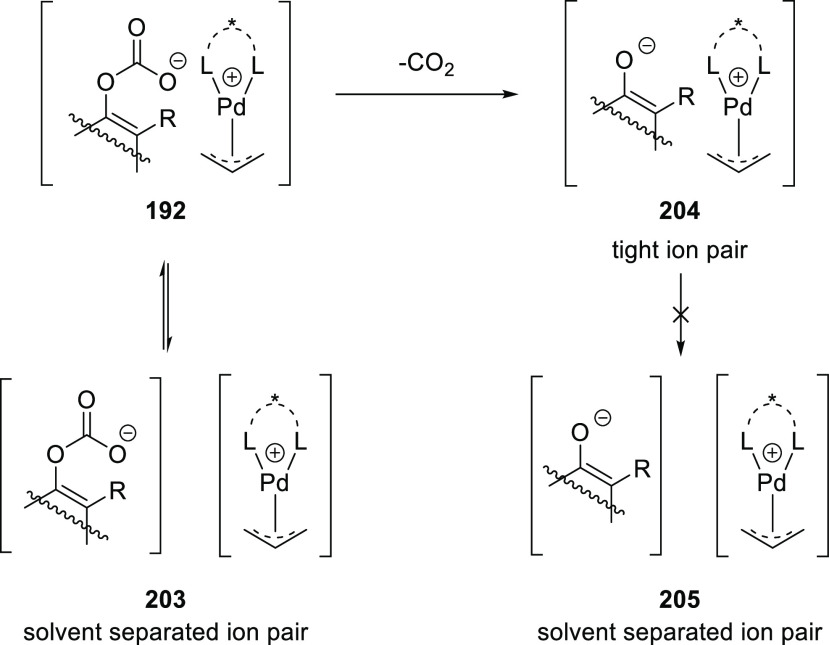
Potential Intermediates in the DAAA of Allyl Enol Carbonates

Crossover experiments revealed complete scrambling of the Pd η^3^-allyl cation between solvent-separated ion pairs. However, interpretation of crossover experiments is complex, as allyl scrambling can occur before or after decarboxylation.^366,560,561,634,642^ Trost argued that, since the enol carbonate anion is more stabilized than the enolate anion, facilitating the formation of a solvent separated Pd allyl/enol carbonate ion pair **203**, scrambling likely occurs at this stage. As a further probe, reactions were carried out in the presence of acidic additives such as malonic esters, which were thought to protonate a “naked” enolate. However, in dioxane as solvent, only a small amount of protonated enolate was formed, indicating that the Pd allyl enolate intermediate existed mainly as a tight ion pair **204** and that the reaction proceeded through this intermediate rather than a solvent separated enolate **205** (Scheme 245).^643^

**Scheme 246:**
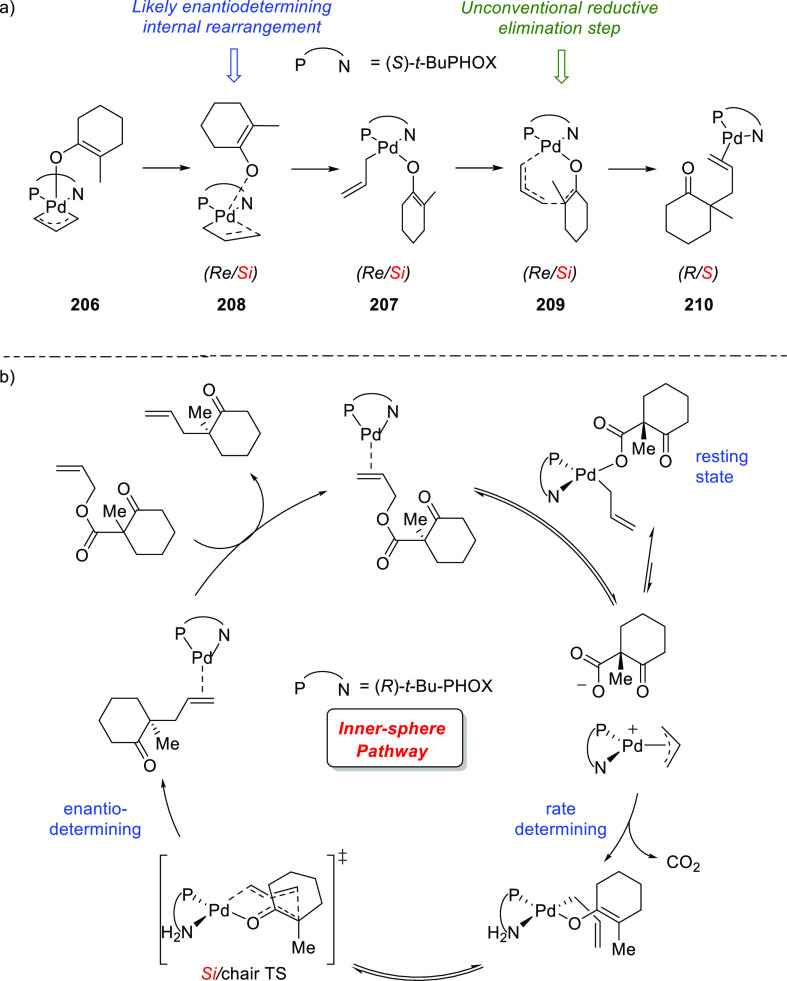
(a) Originally Proposed Possible Intermediates in the Pd/(*S*)-*t*-Bu-PHOX-Catalyzed DAAA and (b) Updated Catalytic Cycle in the Pd/(*R*)-*t*-Bu-PHOX-Catalyzed DAAA

Following their original proposal of an inner-sphere pathway (Figure 30), Stoltz and Goddard performed exhaustive DFT studies to delineate the reductive elimination step in Pd(PHOX)-catalyzed DAAA reactions.^618,644^ Three different computational arrangements using diverse levels of DFT showed a preference for the inner sphere mechanism. In addition, DFT calculations for the outer sphere mechanisms were not in agreement with the observed enantioselectivities. On the basis of these results, the pathway shown in Scheme 246a was proposed, starting from a 5-coordinate complex **206** that subsequently undergoes an internal rearrangement to the 4-coordinate Pd complex **207** via transition state **208**. According to the calculated energy profiles of the *Re-* and *Si-*pathways, the enantioselectivity was determined by this transition state. The final step was formulated to occur through reductive elimination via a seven-membered transition state **209** to yield Pd-olefin complex **210**. This year, Stoltz and Goddard reported a further detailed (and elegant) quantum mechanics investigation into the DAAA catalyzed by the Pd(PHOX) system (Scheme 246b).^645^ They presented mechanistic insights that unite all current experimental observations, including enantioinduction, reaction rate, catalyst resting state, enolate crossover experiments, water tolerance, and the effects of solvation on inner- and outer-sphere mechanisms. Starting with racemic allyl β-keto ester, oxidative addition of the Pd^0^(PHOX) proceeds through olefin coordination and electrophilic addition to Pd to yield an ion pair. This ion pair rapidly equilibrates to the previously discussed (and characterized) catalyst resting state, an off-cycle intermediate. Thereafter, decarboxylation, which is the rate limiting step, occurs to afford the key intermediate, already predisposed to undergo the enantiodetermining inner-sphere C–C bond formation via the 7-membered pericyclic transition state shown (*Si*/chair). In addition, given the experimentally observed water tolerance, an inner-sphere mechanism for C–C bond formation is generally invoked for the Pd(PHOX) system. This computational study helps to rationalize the water tolerance and the effect of solvation in this system.

**Scheme 247:**
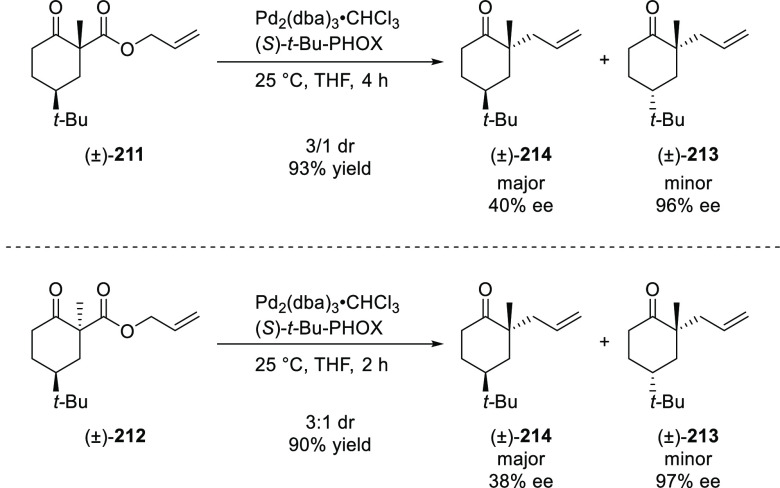
Investigating the Stereoablative Nature of the DAAA

**Figure 31:**
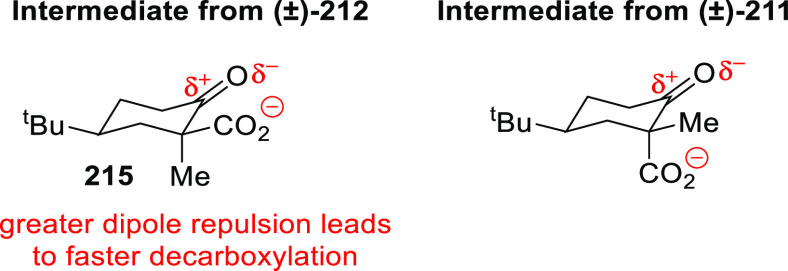
Possible reactive intermediates that control the rate.

Stoltz had come up with the term “stereoablative enantioconvergent catalysis” to describe a process, in which the chiral starting material is converted to an achiral intermediate that favors the formation of one of the product’s enantiomer under the effect of a chiral catalyst. His group investigated the Pd-catalyzed DAAA of diastereomeric β-ketoesters in 2014 to learn if the process was indeed a stereoablative transformation (Scheme 247).^646^ Two diastereomeric substrates (±)-**211** and (±)-**212** with opposite configuration at the α-position were tested in the DAAA. The comparable diastereomeric product ratios (3/1 dr) found for both substrates are highly suggestive of a stereoablative process. In addition, the reaction rate was much faster for one diastereomer (±)-**212** than the other and the minor diastereomers (±)-**213** were formed in a higher enantioselectivity than the major diastereomers (±)-**214**. It was proposed that the rate difference was attributed to the greater dipole repulsion in the intermediate in which the carbonyl group is nearer to the carboxylate group (**215**) favoring the decarboxylation (Figure 31).^647^ Higher levels of enantioselectivity were seen for the minor product (±)-**213**, and this was due to greater catalyst control vs substrate control for this enantiomer in comparison to the other three possible products.

In conclusion, as described in this section, a detailed mechanistic picture of the Pd-catalyzed DAAA of allyl enol carbonates, allyl β-ketoesters and silyl enol ethers has evolved over the years from the work of Tsuji, Trost, Stoltz, Goddard, Tunge, and others.

### 3.4 Decarboxylative Asymmetric Propargylic Alkylation

**Scheme 248:**
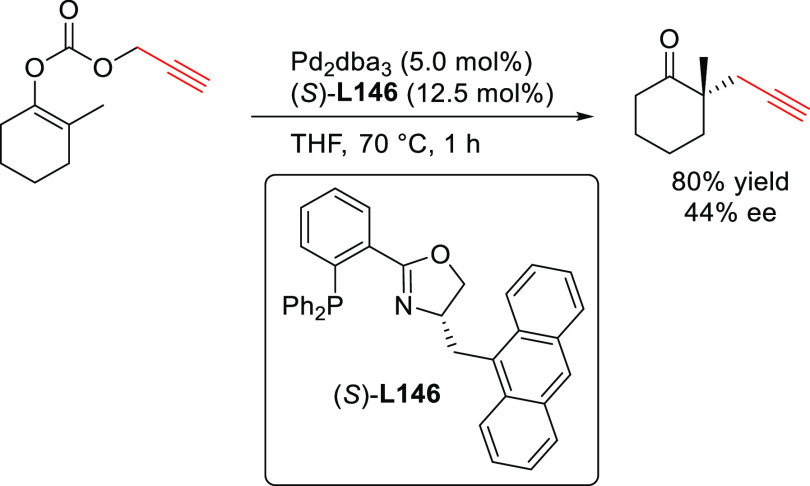
DAPA of 2-Methyl-2-(prop-2-yn-1-yl)cyclohexan-1-one Using Pd/(*S*)-**L146** Catalyst

In 2011, Stoltz and co-workers investigated propargyl enol carbonates as substrates in Pd-catalyzed decarboxylative asymmetric propargylic alkylation (DAPA) (Scheme 248).^618^ The propargylic electrophile is particularly challenging because of the myriad of products it can form under Pd-catalysis.^648^ It was found that propargylation requires considerably elevated temperatures. The best results were achieved with the Pd catalyst derived from PHOX ligand **L146**, affording 2-methyl-2-(prop-2-yn-1-yl)cyclohexan-1-one in an 80% yield but with only a modest *ee* value of 44%.

**Scheme 249:**
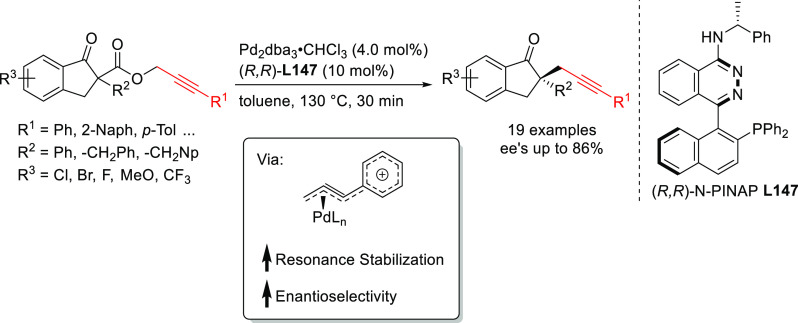
DAPA under Forcing Conditions with Good Enantioselectivity

The Guiry group recently reported the DAPA of a range of α-aryl β-keto propargyl ester indanones (Scheme 249).^649^ Initial experiments using a terminal alkyne (R^1^ = H), produced only an unwanted α-protonated product. Upon further investigation, the major proton source was found to be the terminal alkyne. Silyl or alkyl groups at the alkynyl terminus led to side reactions but with R^1^ = Ph, the substrate could be successfully converted to the desired product in 64% yield with an *ee* of 78%. A Hammett-like correlation between the *ee* values and the electronic nature of the R^1^ aryl group was observed. An increase in the resonance-donation ability of the aryl substituent led to higher *ee* values However, rather forcing conditions were required. With (*R,R*)-N-PINAP **L147** as the ligand in a sealed tube in toluene, 130 °C proved to be optimal. These reactions could also be conducted in a microwave oven with near identical results. Achieving high enantioselectivities under such severe reaction conditions is unusual for Pd-catalysis.

### 3.5 Application in Total Synthesis

The Pd-catalyzed decarboxylative asymmetric allylic alkylation (DAAA) has become an extremely useful reaction for the construction of all-carbon quaternary chiral centers next to a carbonyl group. Chiral α,α-disubstituted ketones, amides, lactones, and related compounds are found in many natural products, and therefore, it is not surprising that there are plenty of total synthesis that rely on the DAAA.

**Scheme 250:**
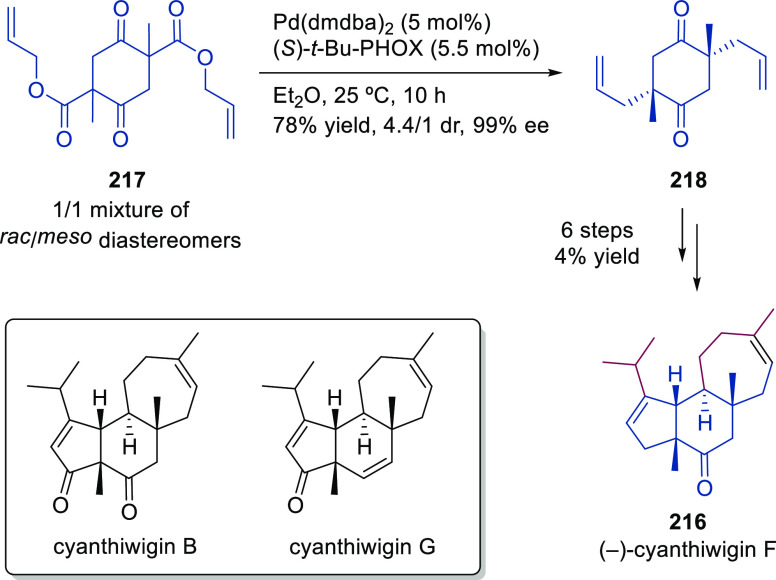
Synthesis of Cyanthiwigin F (**216**)

Stoltz and co-workers reported in 2008 the total synthesis of the marine diperteneoid (−)-cyanthiwigin F (**216**; Scheme 250).^650,651^ The key step in the synthesis is the double catalytic decarboxylative allylic alkylation of a 1:1 mixture of racemic and *meso*-diastereoisomers of bis(β-ketoester) **217** using the Pd/(*S*)-*t*-Bu-PHOX catalytic system. The reaction took place with 4.4/1 diastereoselectivity in favor of the (*R*, *R*)-**218**, which could be isolated in 78% yield with excellent enantioselectivity (99% *ee*). From this intermediate, the preparation of **216** could be achieved in six steps with 4% overall yield. The same group took later advantage of the double asymmetric alkylation of **217** to enlarge the members of the cyanthiwigin family that can be easily accessed with the preparation of cyanthiwigin B and G.^652^

In 2016, Stoltz and co-workers reported an improved synthesis of the cyanthiwigin natural products family, which relied on the reoptimization of the key double catalytic enantioselective alkylation using a protocol employing low catalyst loadings.^653^ The other improvement was the use of an anti-Markovnikov Tsuji-Wacker oxidation for the preparation of a key bicyclic aldehyde instead of the cross-metathesis/oxidation protocol used in the original strategy.

**Scheme 251:**
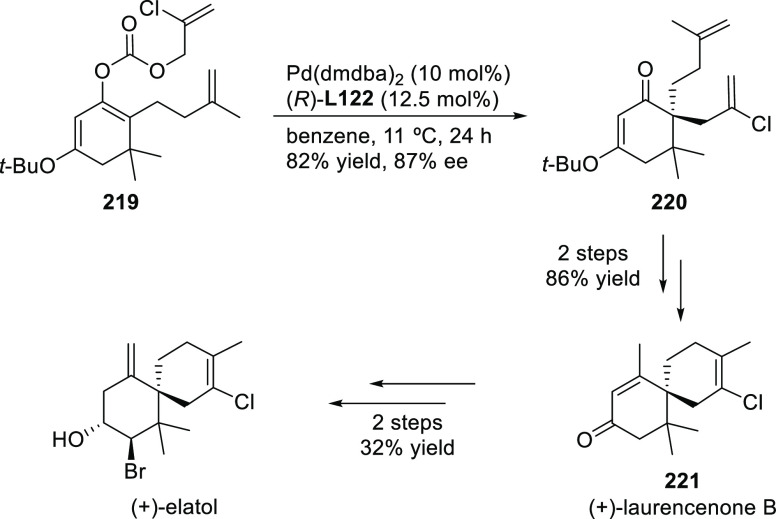
Synthesis of (+)-Laurencenone B and (+)-Elatol

DAAA has been also crucial in developing a general enantioselective synthesis of chamigrene sesquiterpenes, which possess a spiro[5.5]undecane core.^654,655^ In this context, the key step in the synthesis of (+)-elatol, one of the most studied chamigrenes, and (+)-laurencenone B, was the DAAA of enol carbonate **219** (Scheme 251). The use of Pd/(*R*)-**L122** catalyst provided diene **220** in high yield and enantioselectivity (87% *ee*). Then, a two-step sequence involving ring closing metathesis and methylation (MeLi) in the presence of CeCl_3_ afforded (+)-laurencenone B (**221**) in 86% yield. Stereoselective α-bromination and *cis*-stereoselective reduction (DIBAH) produced (+)-elatol in 32% yield.

**Scheme 252:**
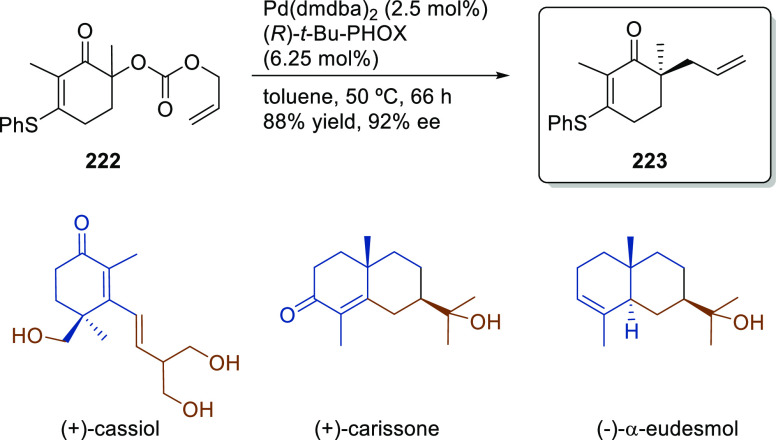
Synthesis of (+)-Cassiol, (+)-Carissone, and (−)-α-Eudesmol

The same group also made use of the Pd-catalyzed DAAA of cyclic vinylogous thioester **222** for the synthesis of (+)-cassiol and the eudesmane sesquiterpenoids (+)-carissone and (−)-α-eudesmol (Scheme 252).^566,567^ The use of Pd/(*R*)-*t*-Bu-PHOX catalyst yielded the key intermediate **223** in 92% *ee* and 88% yield. From **223**, the preparation of all three compounds could be completed uneventfully in five steps.

**Scheme 253:**
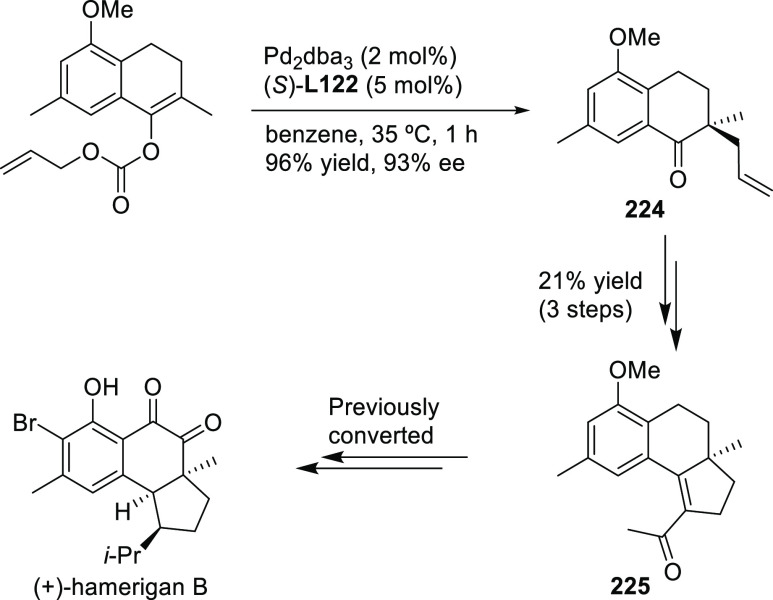
Synthesis of (+)-Hamerigan B

The Pd-catalyzed DAAA was also the critical step in the asymmetric formal synthesis of (+)-hamerigan B (Scheme 253), which has shown anticancer activity against the P-388 leukemia cell line and antiviral activity against herpes and polio viruses.^656^ The Pd/(*S*)-**L122** catalyst was used in the enantioselective DAAA step to form tetralone **224** in excellent yield (96%) and enantioselectivity (93% *ee*). Ru-catalyzed cross metathesis of intermediate **224** with methyl vinyl ketone, followed by a Cu hydride-mediated domino conjugate reduction-cyclization, yielded the late-stage intermediate **225** previously used in the preparation of (+)-hamerigan B.

**Scheme 254:**
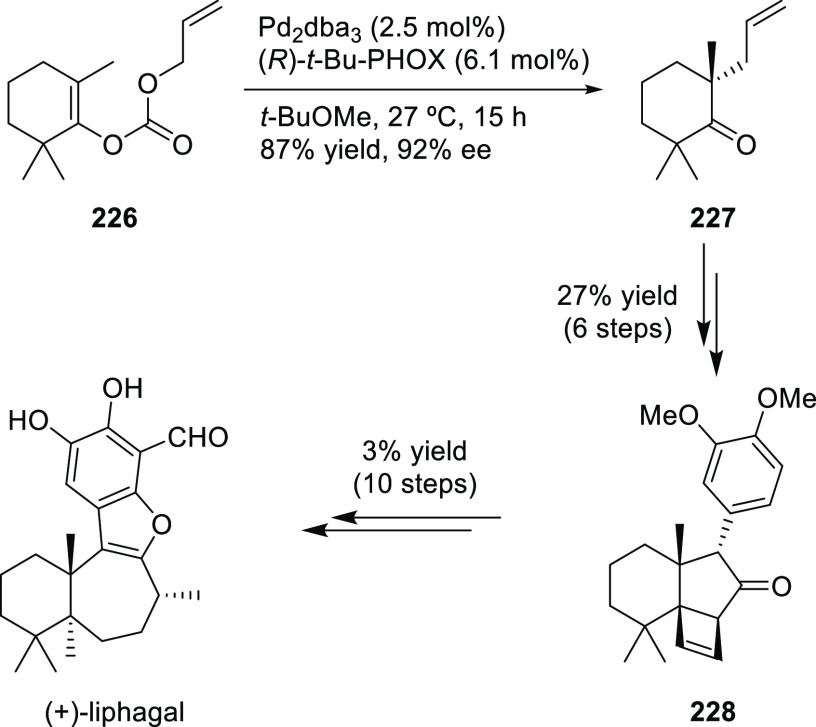
Synthesis of (+)-Liphagal

Stoltz and co-workers also developed an efficient route to optically enriched (+)-liphagal, a tetracyclic meroterpenoid natural product from the Caribbean sponge *Aka coralliphaga* (Scheme 254).^657^ Pd-catalyzed DAAA of enol carbonate **226** yielded tetrasubstituted ketone **227** in high yield (87%) and enantioselectivity (92% *ee*) using the (*R*)-*t*-Bu-PHOX ligand. Compound **227** was elaborated through a further six steps to afford tricyclic aryl ketone **228**. From this intermediate, (+)-liphagal was accessed in a 10 step sequence that involves ring expansion by selective cleavage of the strained cyclobutene, furan formation, olefin reduction, formylation and demethylation reactions.

**Scheme 255:**
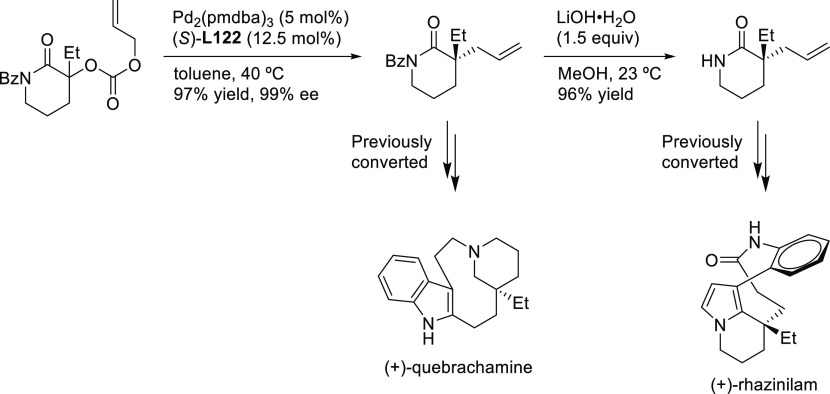
Synthesis of (+)-Quebrachamine and (+)-Rhazinilam

Soon after, the same group took advantage of their finding that Pd/(*S*)-**L122** catalyst could be efficiently used in the decarboxylative allylic alkylation of lactams to prepare synthetic intermediates for the formal total synthesis of *Aspidosperma* alkaloids (+)-quebrachamine and (+)-rhazinilam (Scheme 255).^587^

**Scheme 256:**
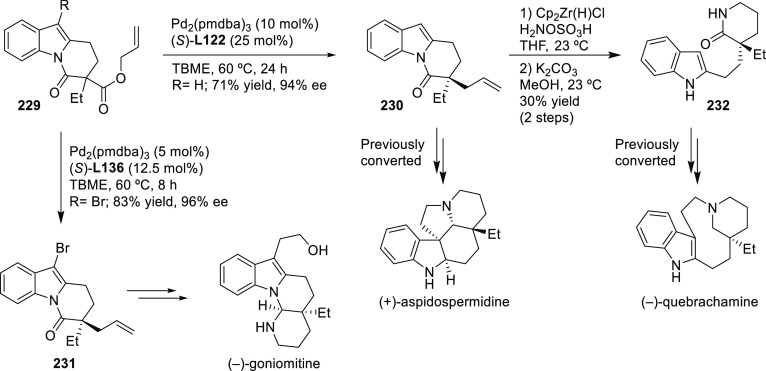
Synthesis of (−)-Quebrachamine, (+)-Aspidospermidine, and (−)-Goniomitine from Dihydropyrido[1,2-*a*]indolones **229**

In 2016, a new synthesis of (−)-quebrachamine and other monoterpene indole alkaloids, such as (+)-aspidospermidine and (−)-goniomitine, was developed by Stoltz and co-workers (Scheme 256).^658,659^ This new protocol relies on the highly efficient Pd-catalyzed DAAA of dihydropyrido[1,2-*a*]indolones. The authors identified (*S*)-**L122** as the optimal ligand for the DAAA of dihydropyrido[1,2-*a*]indolone **229**, yielding key intermediates **230** and **231** in high enantiomeric excesses (94% and 96%, respectively). α-Quaternary lactam **230** was transformed into **232** by hydroamination followed by an amide exchange. Compounds **230** and **232** are key intermediates in the formal synthesis of (+)-aspidospermidine and (−)-quebrachamine, respectively. For the total synthesis of (−)-goniomitine, intermediate **231** was subjected to a Negishi cross-coupling, followed by a formal hydroamination and subsequent reduction (28% overall yield from **229**).

**Scheme 257:**
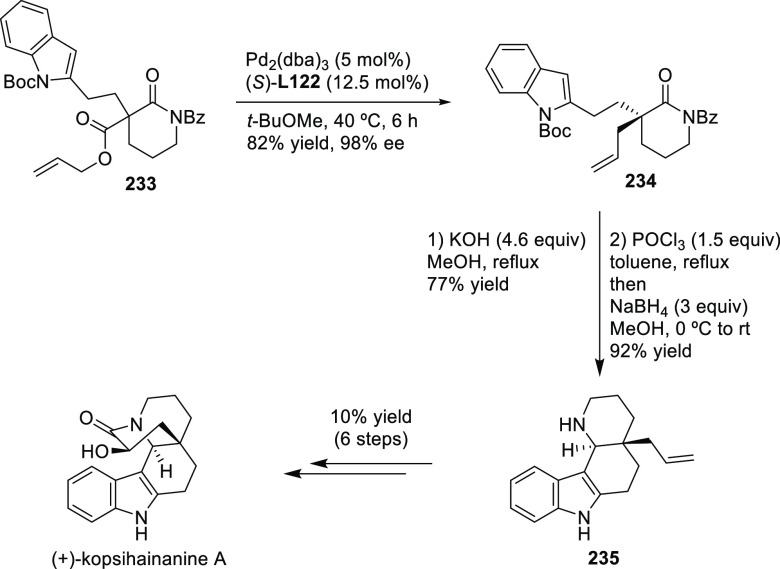
Synthesis of (+)-Kopsihainanine A

Mukai and co-workers took advantage of the Stoltz’s Pd-catalyzed DAAA of lactams to achieve the total synthesis of (+)-kopsihainanine A, a monoterpenoid indole alkaloid present in the leaves and stems of *Kopsia hainanesis* (Scheme 257).^660^ Thus, they also used Pd/(*S*)-**L122** catalyst to decarboxylatively alkylate lactam **233**, which gave access to chiral δ-lactam **234** in high yield and enantioselectivity. The Bischler–Napieralski cyclization of **234** induced by POCl_3_ followed by stereoselective reduction with NaBH_4_ afforded compound **235** with the required indoloperhydroquinoline backbone. Finally, (+)-kopsihainanine A was prepared by a consecutive oxidation of the allyl group and condensation of **235** together with the corresponding protecting/deprotecting sequences in 99% *ee* and 7% overall yield.

**Scheme 258:**
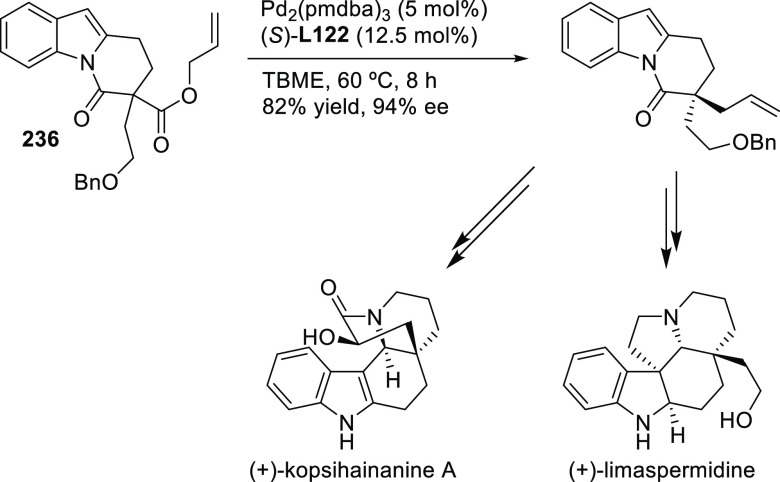
Synthesis of (+)-Limaspermidine and (+)-Kopsihainanine A

More recently, the Stoltz group coupled the Pd-catalyzed DAAA of dihydropyrido[1,2-*a*]indolone **236** with stereodivergent Pictet-Spengler and Bischler-Napieralski cyclization strategies for the synthesis of (+)-limaspermidine and (+)-kopsihainanine A (Scheme 258).^661^

**Scheme 259:**
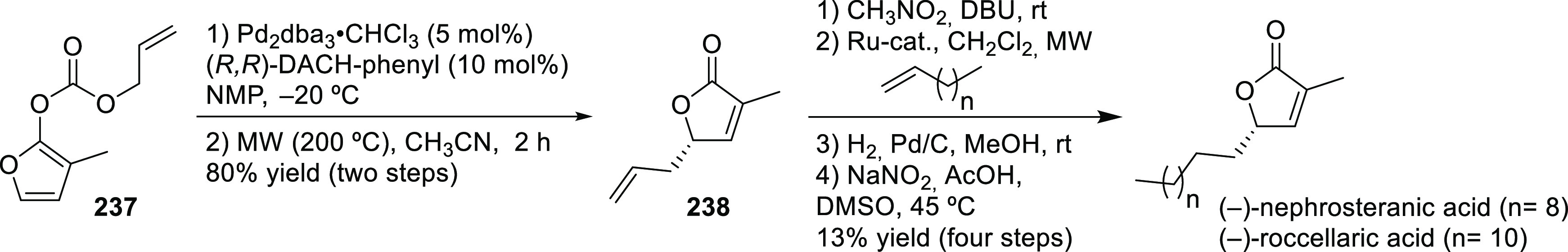
Synthesis of (−)-Nephrosteranic Acid and (−)-Roccellaric Acid

Arseniyadis, Cossy and co-workers developed the Pd-catalyzed DAAA of cyclic dienol carbonates and applied this methodology to the synthesis of (−)-nephrosteranic acid and (−)-roccellaric acid, which have anticancer and antibiotic properties (Scheme 259).^598^ Thus, the DAAA of allyl dienol carbonate **237** using Pd/(*R*,*R*)-DACH-phenyl yielded the corresponding α-quaternary butenolide, which is converted into the corresponding γ-tertiary furanone **238** by a stereoselective Cope rearrangement. This compound was then subjected to a diastereoselective 1,4-conjugate addition of nitromethane, followed by Ru-catalyzed cross-metathesis to elongate the side chain, subsequent hydrogenation over Pd/C, and a final Kornblum oxidation to yield (−)-nephrosteranic acid and (−)-roccellaric acid.

**Scheme 260:**
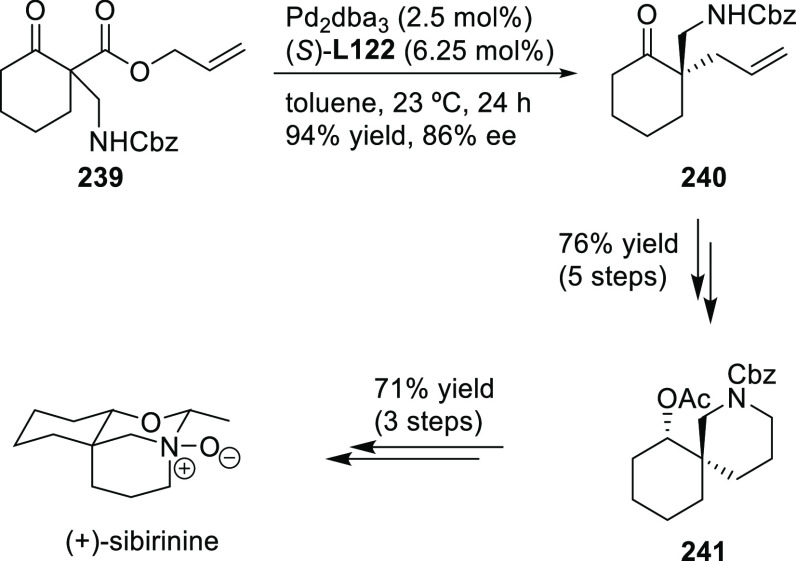
Synthesis of (+)-Sibirinine

Stoltz and co-workers studied the Pd-catalyzed DAAA of β-aminomethyl-β-keto esters to access α-quaternary Mannich-type adducts using Pd/(*S*)-**L122** catalyst. The usefulness of this procedure was demonstrated with the first total synthesis of (+)-sibirinine, a tricyclic alkaloid (Scheme 260).^662^ The asymmetric alkylation of **239** yielded β-amino ketone **240** in 94% yield and 86% *ee*. Compound **240** was then diastereoselectively reduced with DIBAL, followed by acetylation, subsequent hydroboration of the terminal alkene and a final cyclization to yield spirocycle **241**. The synthesis of (+)-sibirinine was then completed by deprotection of the acetyl and Cbz groups, followed by hemiaminal formation and subsequent oxidation in an excellent 51% overall yield from **239**.

**Scheme 261:**
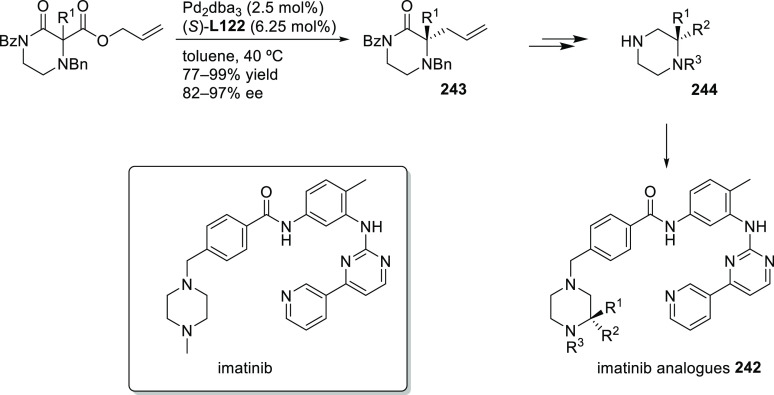
Synthesis of Imatinib Chiral Analogues **242**

Stoltz and co-workers have also prepared chiral analogues **242** of imatinib, a piperazine-containing anticancer drug (Scheme 261).^589^ The key transformation is the enantioselective synthesis of α-tertiary piperazin-2-ones (**243**) via decarboxylative asymmetric allylic alkylation, which proceeds in excellent yields and enantioselectivity using the Pd/(*S*)-**L122** catalyst system. Intermediates **243** were then easily converted into α-tertiary piperazines **244**, which were then converted to compounds **242**.

**Scheme 262:**
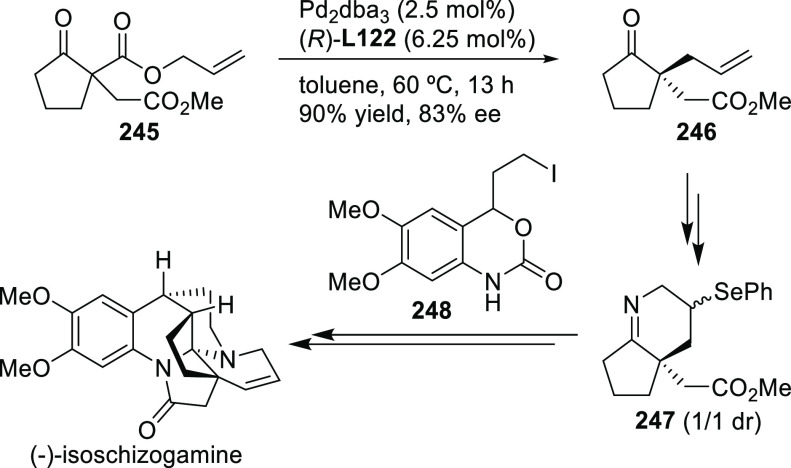
Synthesis of (−)-Isoschizogamine

Zhu and co-workers carried out the enantioselective synthesis of (−)-isoschizogamine, a complex polycyclic monoterpene indole alkaloid, employing a Pd-catalyzed DAAA of β-keto ester **245** as the strategic step determining the absolute configuration of the final compound (Scheme 262).^663^ Using Pd/(*R*)-**L122** as the catalyst, the alkylation took place to yield α-quaternary ketone **246** in high yield (90%) and enantiomeric purity (83% *ee*). Intermediate **246** was converted to bicyclic enantioenriched imine **247** by azido-phenylselenenylation of the terminal double bound followed by an intramolecular aza-Wittig reaction. *N*-Alkylation of **247** with the alkyl iodide **248** provided in a highly convergent manner an iminium precursor which was converted into the hexacyclic structure of (−)-isoschizogamine, with complete control of both relative and absolute configuration, by microwave heating in the presence of pivalic acid. A selenoxide elimination completed the synthesis of (−)-isoschizogamine (12% overall yield from **245**).

**Scheme 263:**
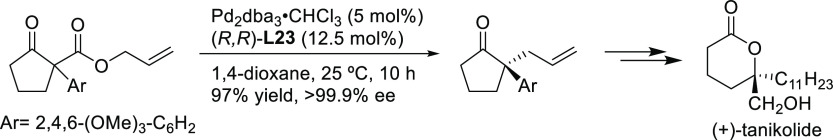
Synthesis of (+)-Tanikolide

Guiry and co-workers established the highly enantioselective Pd-catalyzed DAAA of cyclopentenone-derived α-aryl-β-keto esters using Trost’s ligand (*R*,*R*)-**L23**. They exploited this transformation for the asymmetric formal synthesis of (+)-tanikolide, a toxic and antifungal marine natural product from the algae cyanobacterium *Lyngbyamajuscula* (Scheme 263).^570^

**Scheme 264:**
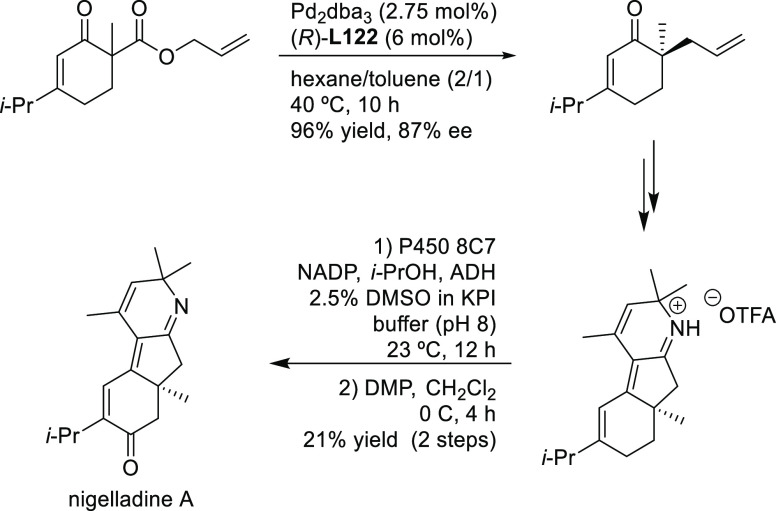
Synthesis of Nigelladine A

Arnold, Stoltz, and co-workers developed an enantioselective total synthesis of nigelladine A, a norditerpenoid alkaloid with potent protein tyrosine phosphatase 1B inhibitory activity isolated from *Nigella glandulifera* (Scheme 264).^664^ This synthesis relied upon the Pd-catalyzed DAAA for the construction of the quaternary stereogenic center in high yield and enantioselectivity, and on the late-stage chemo- and regioselective allylic C–H oxidation enabled by an engineered P450 enzyme.

**Scheme 265:**
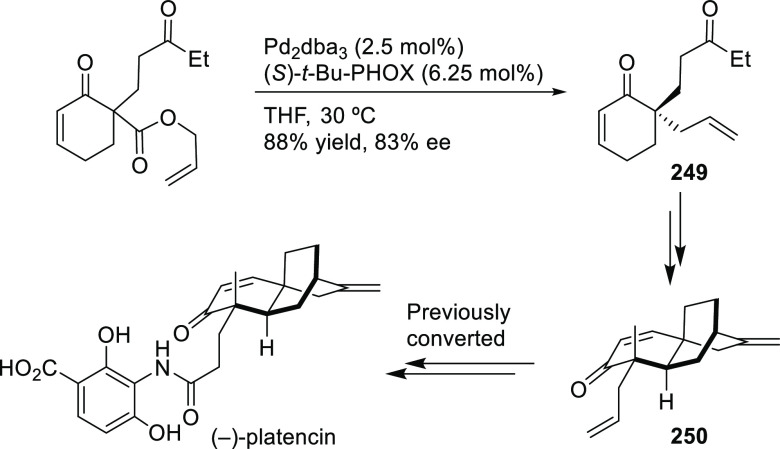
Synthesis of (−)-Platencin

The Pd-catalyzed DAAA was also used to synthesize β-keto ester **249** as the key intermediate in the enantioselective formal synthesis of the natural antibiotic (−)-platencin (Scheme 265).^665^ From chiral intermediate **249**, a radical-mediated cyclization led to the formation of the bicyclo[2.2.2]octane core that was further transformed to tricyclic intermediate **250** via a regioselective aldol cyclization. Compound **250**, which was prepared in 3.5% overall yield, had been previously converted to the target (−)-platencin.

**Scheme 266:**
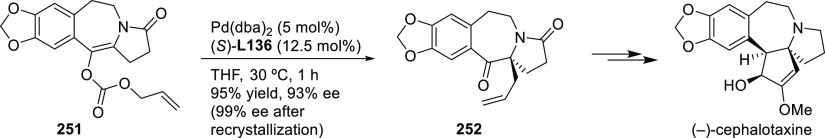
Synthesis of (−)-Cephalotaxine

Zhang and co-workers reported the asymmetric formal synthesis of (−)-cephalotaxine employing the Pd-catalyzed DAAA (Scheme 266).^666^ The use of Pd/(*S*)-**L122** catalyst enabled the enantioselective alkylation of the tetracyclic allyl enol carbonate **251** leading to intermediate **252** and affording the key aza-containing tetrasubstituted stereogenic center. From intermediate **252**, (−)-cephalotaxine could be prepared in 7 steps in 99% *ee*.

**Scheme 267:**
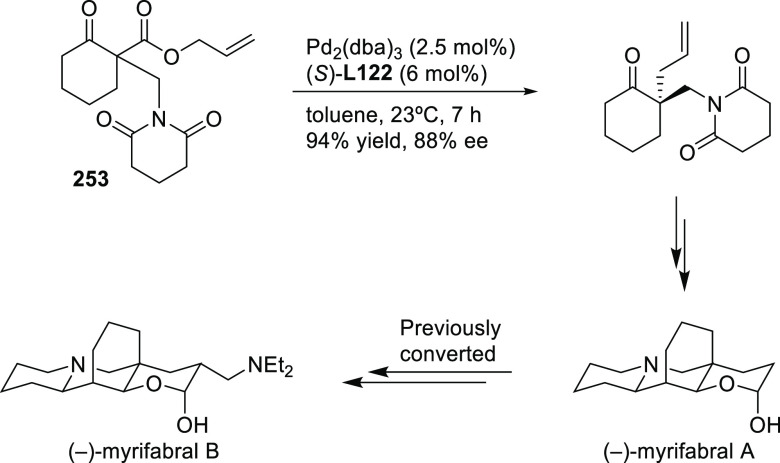
Synthesis of (−)-Myrifabral A and B

A very recent example from the Stoltz group on the use of Pd-catalyzed DAAA as a key transformation in total synthesis can be found in the asymmetric synthesis of the *Myrioneuron* alkaloids (−)-myrifabral A and B (Scheme 267).^667^ The use of the Pd/(*S*)-**L122** catalyst generated the C(10) all-carbon quaternary center (from the key compound **253**). The synthesis of myrifabral A was accomplished from **253** in 66% overall yield followed by diastereoselective *N*-acyl iminium cyclization, cross metathesis and subsequent oxidation. Myrifabral A was converted to myrifabral B using previously reported conditions.

In summary, the examples described in this section clearly demonstrate the robustness of Pd-catalyzed DAAA as an important transformation for synthetic organic chemists to generate all carbon-quaternary stereocenters which are widespread in natural products. A number of investigations have been performed by the Stoltz and Trost groups (primarily) and others using DAAA as a key step to generate a quaternary stereocenter for the synthesis of compounds of interest from nature and in medicinal chemistry research programs. Many of the researchers in this field have not just been interested in developing the DAAA as a general synthetic method based on studies of test substrates, but applications in total synthesis have been a driving force as they require the use, and study, of suitably substituted and functionalized starting materials. For the vast majority of examples cited, the ready availability of the PHOX and Trost-type ligands has allowed rapid integration of the DAAA into mainstream total synthesis planning. In addition, the allyl unit present in all DAAA products is a versatile functional handle which has been exploited exquisitely in the examples discussed. This section shows that the application of Pd-mediated DAAA in total synthesis is a thriving research area, with more examples compared to other Pd-catalyzed AAA processes, and it will be interesting to follow its future development.

## 4 Asymmetric Oxidative Allylic Substitution

### 4.1 Allylic Substitution through C–H Activation

Stoichiometric Pd-mediated allylic functionalizations of alkenes via the formation of a Pd η^3^-allyl complex through C–H bond cleavage, followed by nucleophilic attack, have been known for a long time.^668,669^ The cleavage of the allylic C–H bond has been found to be stereospecific occurring with retention of configuration.^670,671^ Catalytic allylic C–H oxidations were later reported using palladium acetate and *p*-benzoquinone (BQ) with acetate as nucleophile (eq 1).^672−675^
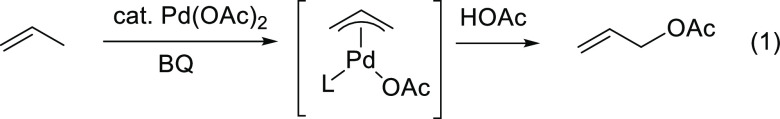
1

These catalytic allylic acetoxylations are considered to proceed via Pd η^3^-allyl complexes. In the early studies an alternative mechanism via an acetoxypalladation−β-elimination pathway (Wacker-type mechanism) was also considered, in particular for cyclic olefins and other internal olefins. Evidence for a Pd η^3^-allyl intermediate in the acetoxylation of the latter type of olefins was provided by the use of 1,2-dideuterio-cyclohexene, which ruled out the alternative Wacker-type mechanism.^676,677^ Further developments of this Pd-catalyzed allylic acetoxylation have been carried out by White and co-workers,^678,679^ and these reactions have subsequently also been applied to asymmetric versions (see below).

Although oxygen nucleophiles as carboxylate (acetate) were used early in Pd-catalyzed oxidative allylic substitutions, it took some time before these reactions were extended to nitrogen and carbon nucleophiles. Significant progress on allylic aminations of alkenes involving C–H activation were made by the groups of White^680^ and Liu.^681^

In 2008, Shi^682^ and White^683^ independently reported the use of carbon nucleophiles in the Pd-catalyzed oxidative allylic substitution. In these reactions, various stabilized carbon nucleophiles were used, such as β-dicarbonyl compounds and methyl nitroacetate. These achievements were of great importance since now oxidative allylic alkylation could be carried out in a catalytic manner through C–H activation.

All these oxidative allylic substitution reactions are thought to proceed via Pd η^3^-allyl intermediates that are formed via an initial C–H bond cleavage. Isotope effect measurements of some Pd-catalyzed allylic substitutions show that the rate-determining step is the C–H bond cleavage, and Hammett studies support a proton abstraction.^684^

### 4.2 Asymmetric Oxidative Allylic Acetoxylation and Alkoxylation

**Scheme 268:**
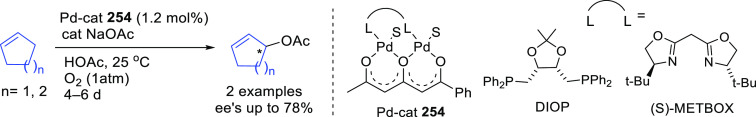
Asymmetric Allylic C–H Acetoxylation of Cyclic Olefins

The first example of asymmetric allylic C–H acetoxylation was reported by Henry and co-workers in 2002 (Scheme 268). They reported that cyclic olefins are oxidized to allylic acetates in good yields in up to 78% *ee* in acetic acid with molecular oxygen as oxidant and with the use of bidentate phosphorus or nitrogen ligands (DIOP or (*S*)-METBOX).^685^

**Scheme 269:**
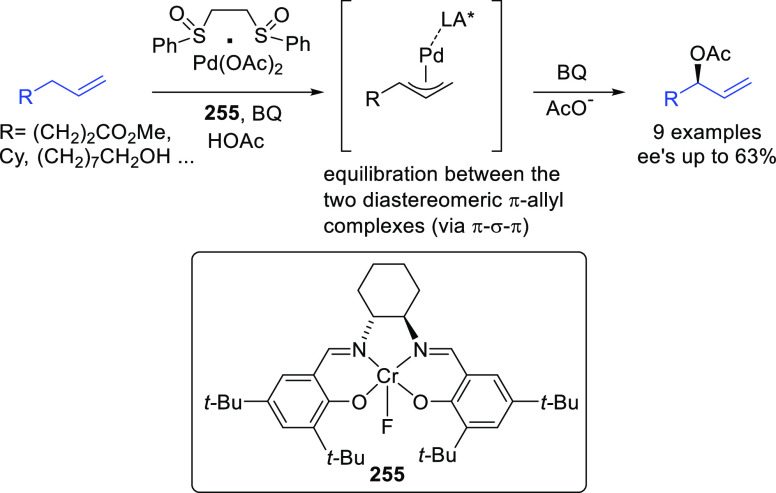
Asymmetric Allylic C–H Acetoxylation of Terminal Olefins

In 2008, Covell and White found that the combination of a bis-sulfoxide Pd acetate complex and a (salen)Cr(III) chiral Lewis acid (**255**) was efficient in the challenging asymmetric allylic C–H acetoxylation of terminal olefins.^686^ In this reaction, the nonlinear allylic acetate was obtained in good selectivity and yield in up to 63% *ee* (Scheme 269).

**Scheme 270:**
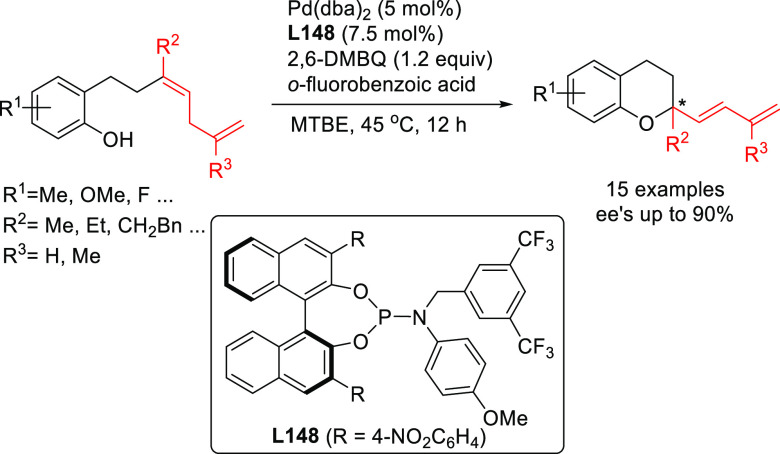
Synthesis of Chromanes via Asymmetric Allylic C–H Alkoxylation

More recently, Gong and co-workers developed an enantioselective allylic C–H alkoxylation using a Pd complex of a chiral phosphoramidite ligand **L148** in combination with *o*-fluorobenzoic acid (Scheme 270).^687^ The reaction was applied to the synthesis of chromanes and employed phenolic dienes as starting material. The allylic oxidation gives the chromanes in good to high yields and in general good *ee* values (up to 90% *ee*). Mechanistic studies ruled out the alternative Wacker oxidation pathway via oxypalladation−β-elimination. Deuteration of the bis-allylic position resulted in an isotope effect of *k*_H_/*k*_D_ = 2.5, showing that the C–H bond cleavage is the rate-limiting step of the reaction.

**Scheme 271:**
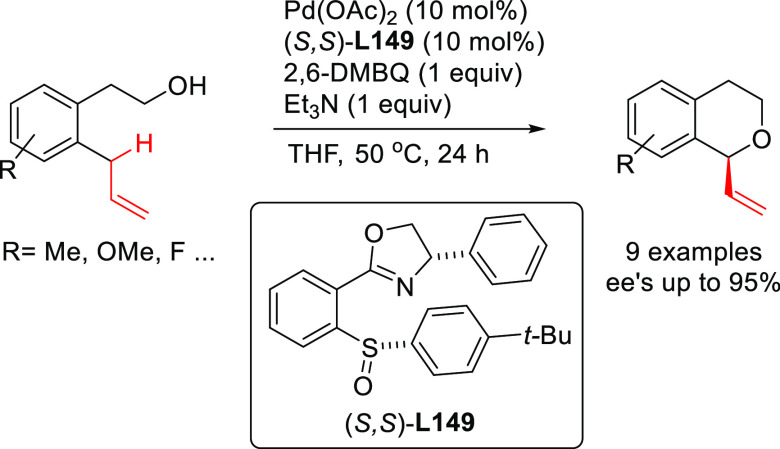
Synthesis of Isochromanes via Asymmetric Allylic C–H Alkoxylation

In a related study White subsequently showed that chiral isochromanes can be efficiently prepared via enantioselective allylic C–H oxidation (Scheme 271).^688^ In these reactions arylethyl alcohols with an allyl group in the *ortho*-position were used as starting materials. An enantioselective intramolecular allylic oxidation using a Pd-catalyzed reaction with chiral oxazoline-sulfoxide **L149** as ligand afforded isochromanes in good yield and very good enantioselectivity.

The use of a sulfoxide-oxazoline ligand (*S*,*S*)-**L149** was found to be highly efficient in promoting high levels of enantioselectivity in all substrates tested. It is interesting to note that similar chiral sulfoxide-oxazoline ligands were used by Liu and Itami in allylic C–H acetoxylations and were found to give highly regioselective and efficient reactions but with poor enantioselectivity (<5% *ee*).^689^ Surprisingly, in the allylic C–H alkoxylations in Scheme 271, these ligands gave high levels of enantioselectivity.^688^

### 4.3 Asymmetric Oxidative Allylic Amination

**Scheme 272:**
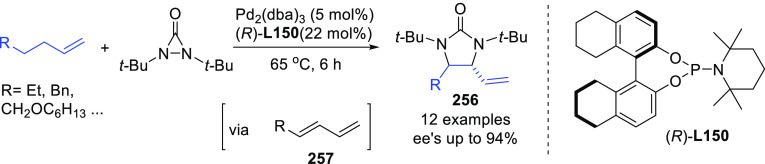
Asymmetric Allylic and Homoallylic Diamination of Terminal Olefins Using Pd/(*R*)-**L150** Catalyst

Although catalytic allylic C–H aminations were reported independently by White and Liu in 2007–2008,^680,681^ there are only limited examples of the enantioselective version of these reactions. Shi reported a Pd-catalyzed enantioselective allylic and homoallylic diamination of terminal olefins by the use of di-*tert*-butyl-diaziridinone to give products **256** (Scheme 272).^690^ Although this reaction involves an allylic C–H amination it does not proceed via the usual C–H activation to give a Pd η^3^-allyl complex followed by nucleophilic attack. The reaction is rather thought to proceed via a conjugated diene **257** that is generated in situ, followed by a Pd-catalyzed vicinal diamination.

**Scheme 273:**
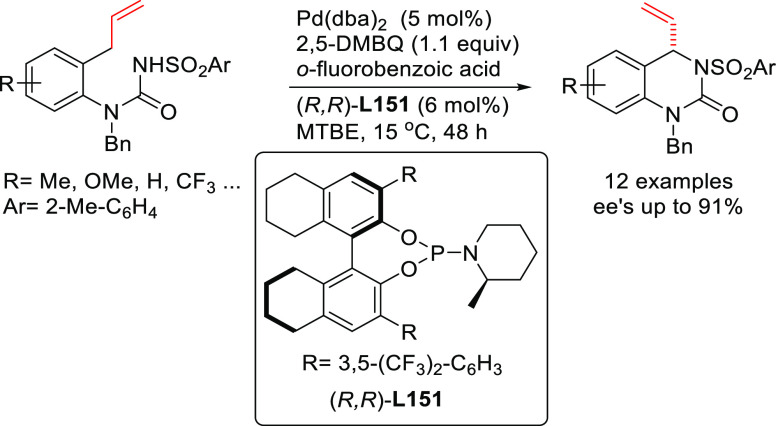
Preparation of Hydropyrimidinones via Asymmetric Allylic C–H Amination

In 2017, Gong and co-workers reported the first example of a direct enantioselective allylic C–H amination.^691^ They used chiral phosphoramidite ligand (*R,R*)-**L151** together with 2,5-dimethyl-benzoquinone (2,5-DMBQ) for the enantioselective cyclization of *N*-((2-allylphenyl)carbamoyl)sulfonamides to hydropyrimidinones in high yields and good enantioselectivity (82–91% *ee*; Scheme 273). This research group had previously demonstrated that the related phosphoramidite ligand (*S*)-**L148** was beneficial in enantioselective allylic C–H oxidation (see Scheme 270 above).^687^

**Scheme 274:**
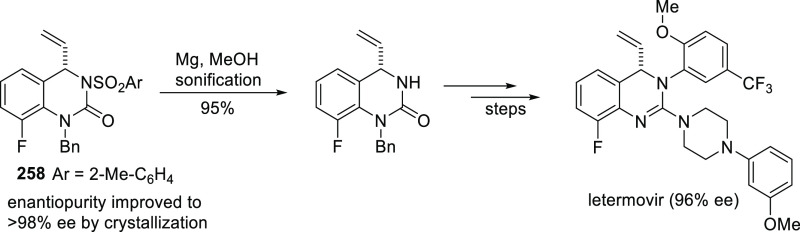
Synthesis of Letermovir

The fluoro derivative **258** was transformed into the biologically active compound letermovir, which is effective for the treatment of human cytomegalovirus (HCMV) infections (Scheme 274). At the time it was in phase III trials and it has since been approved as an antiviral drug. The synthesis begins with deprotection of the arylsulfonyl group in excellent yield followed by functionalization of the olefin and subsequent Cu-catalyzed C–N coupling. After a few more steps, the target molecule was obtained in 96% *ee*.

### 4.4 Asymmetric Oxidative Allylic Alkylation

Enantioselective allylic C–H alkylations can result in chiral products in two principally different ways: (i) the chirality is created at the nucleophilic center and (ii) the chirality is created at the allylic carbon center. Both type of reactions have been described in the literature and they are discussed in sections 4.4.1 and 4.4.2. In section 4.4.3, examples are given that proceed via C(sp^3^)–H activation in the allylic position of an allene, followed by enantioselective C–C bond formation.

#### 4.4.1 Chirality Created at Nucleophile Center

**Scheme 275:**
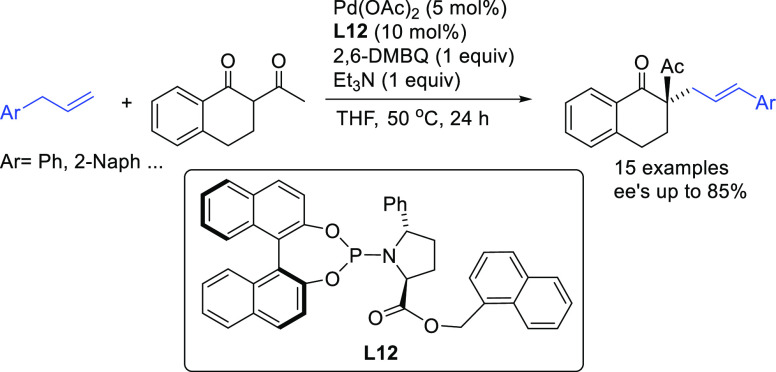
Synthesis of α-Allylated Tetralones via Asymmetric Allylic C–H Alkylation

The first example on an enantioselective allylic C–H alkylation was reported in 2013 by Trost and co-workers.^692^ In this reaction, 2-acetyl-1-tetralone was employed as nucleophile in the Pd-catalyzed reaction of allylarenes to give α-allylated tetralones (Scheme 275). It was found that phosphoramidite ligand **L12** was efficient in promoting the reaction and provided an enantioselective allylic C–H alkylation in up to 85% *ee*. The chirality is created at the nucleophilic center.

**Scheme 276:**
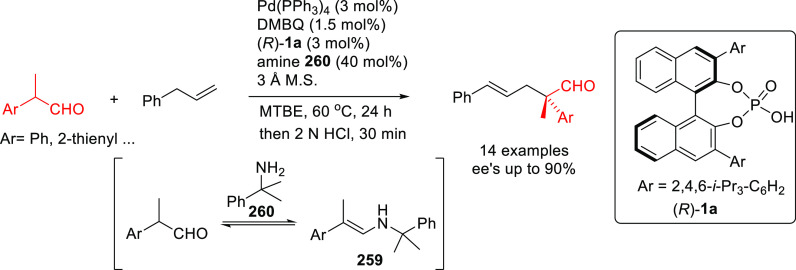
Synthesis of Quaternary α-Allylated Aldehydes through Asymmetric Allylic C–H Alkylation via Enamine

A related reaction was reported by Gong and co-workers in 2014,^693^ where 2-arylpropanals were coupled with allylbenzene via Pd-catalyzed allylic C–H activation in the presence of a chiral phosphoric acid ((*R*)-**1a**) to give quaternary α-allylated aldehydes (Scheme 276). The reaction proceeds via an enamine intermediate **259**, which is formed from reaction of the aldehyde with amine **260**. Enamine intermediates **259** attacks the Pd η^3^-allyl intermediate generated from C–H activation of allylbenzene, and after workup α-allylated aldehydes are formed. The reaction worked in an efficient manner and afforded good yields of chiral aldehydes with high levels of enantioselectivity (up to 90% *ee*). The reaction was also extended to a variety of allylarenes using 2-phenylpropanal as the coupling partner.

**Scheme 277:**
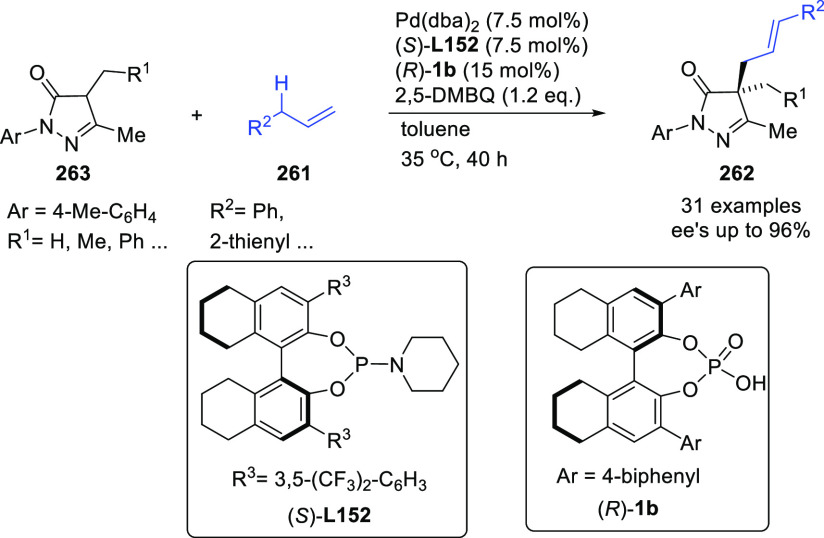
Synthesis of Quaternary α-Allylated Pyrazol-5-ones via Asymmetric Allylic C–H Alkylation

Highly enantioselective allylic C–H alkylation of terminal olefins **261** to give α-allylated pyrazol-5-ones **262** was reported by Gong in 2016.^694^ Pyrazol-5-ones **263** were employed as nucleophiles and a cooperative catalysis of Pd complexes with chiral phosphoramidite ligands and Brønsteds acids was exploited. It was found that the combination of ligand (*S*)-**L152** and phosphoric acid (*R*)-**1b** gave the best results. With this combination good yields and high levels of enantioselectivity (up to 96% *ee*) were obtained (Scheme 277). It was also demonstrated that the R^2^ group on the alkene **261** can be a vinyl group, and in this case diene products **262** are formed from diene **261** (R^2^ = vinyl) and nucleophile **263**.

**Scheme 278:**
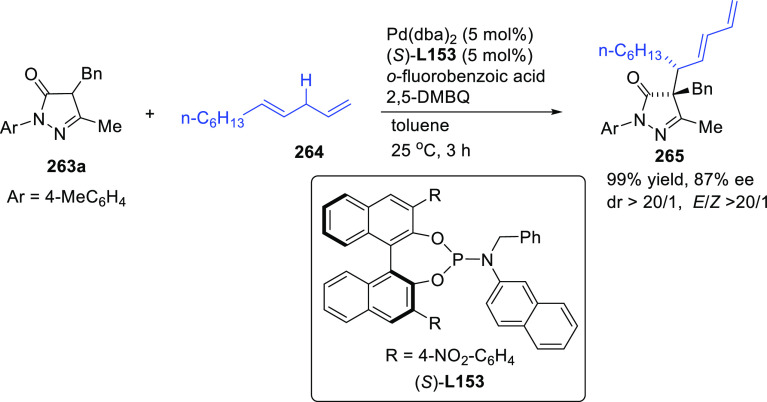
Asymmetric Allylic C–H Alkylation of Diene **264**

In the reaction of pyrazol-5-one **263a** with diene **264**, it was found that chiral ligand (*S*)-**L153**, together with *o*-fluorobenzoic acid gave a high yield and high *ee*.^694^ Substituted diene **264** afforded branched products **265**, in which chirality is created at the allylic carbon center, as well as at the nucleophilic center (Scheme 278). This is the first established example where chirality is created at the allylic carbon center in an enantioselective allylic C–H alkylation.

**Scheme 279:**
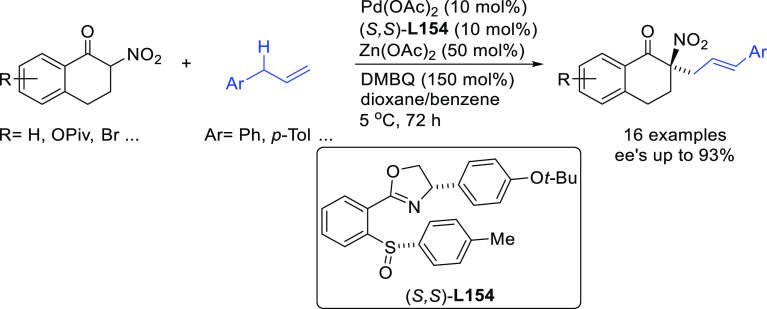
Synthesis of α-Allylated Nitrotetralones via Asymmetric Allylic C–H Alkylation

**Scheme 280:**
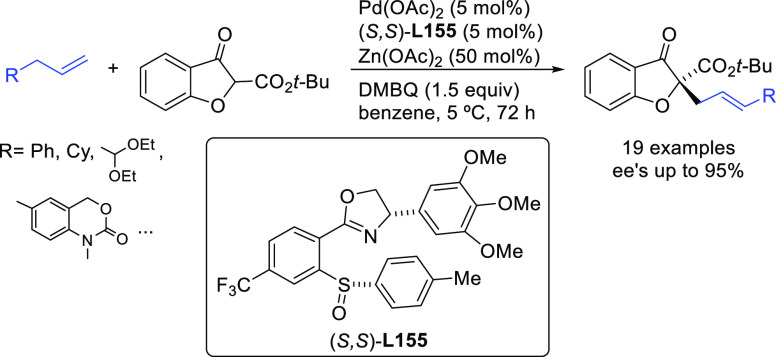
Synthesis of α-Allylated β-Ketoesters via Asymmetric Allylic C–H Alkylation

Efficient asymmetric allylic C–H alkylations of arylarenes, as well as nonactivated aliphatic olefins, were achieved by White using arylsulfoxide-oxazolidine ligands together with Pd(OAc)_2_ (Schemes 279 and 280).^695^ The oxidatively stable ArSOX scaffold was found to be the key to the success with these ligands. With nitrotetralone nucleophiles good yields of α-allylated nitrotetralones with high levels of enantioselectivity were obtained with arylarenes using ligand (*S,S*)-**L154** (*ee* values typically ≥90%; up to 93% *ee*).

The asymmetric allylic C–H alkylations were also extended to β-ketoesters. With β-ketoesters having a furan-3-one core, a range of olefins underwent the reaction with high levels of enantioselectivity (Scheme 280). With β-ketoesters excellent yields and enantioselectivity of allylated products were obtained with various olefins including nonactivated ones. ArSOX ligand (*S*,*S*)-**L155** gave the best results.

In a follow-up study, Gong and co-workers investigated monodentate phosphorus ligands in Pd-catalyzed allylic alkylation reactions of terminal alkenes using a wide range of carbon nucleophiles.^696^ Triarylphosphines and various phosphoramidite ligands were found to give highly efficient alkylation reactions with unactivated terminal olefins under mild conditions. From mechanistic studies it was found that a Pd(0) complex with coordinated monodentate phosphorus ligand, quinone, and alkene is most likely the active species. Importantly, the use of phosphoramidite ligand (*S*)-**L152** and a phosphoric acid related to (*R*)-**1b** (as in Scheme 277) now also gave good results with nonactivated alkenes (R = alkyl) with enantioselectivities up to 90% *ee*.

**Scheme 281:**
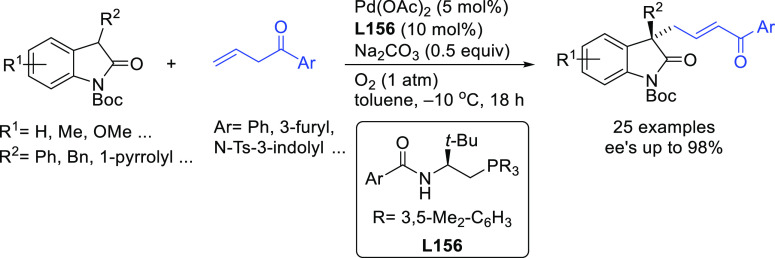
Synthesis of Quaternary α-Allylated Oxindoles via Asymmetric Allylic C–H Alkylation Using a Chiral Phosphinium-Based Phase-Transfer Catalyst

Enantioselective Pd-catalyzed allylic C–H alkylations with the use of chiral phosphinium-based phase transfer catalysts were reported by Du and Chen.^697^ In these reactions terminal alkenes with a carbonyl function in the 3-position were used as substrates and 3-substituted oxindoles were employed as nucleophiles (Scheme 281). A remarkable feature of these reactions is that molecular oxygen (O_2_) can be used as a direct oxidant and a quinone is not required in the reaction. The best results were obtained with chiral phase transfer catalyst **L156** resulting in excellent *ee* values of the quaternary α-allylated oxindoles.

#### 4.4.2 Chirality Created at the Allylic Carbon Center

**Scheme 282:**
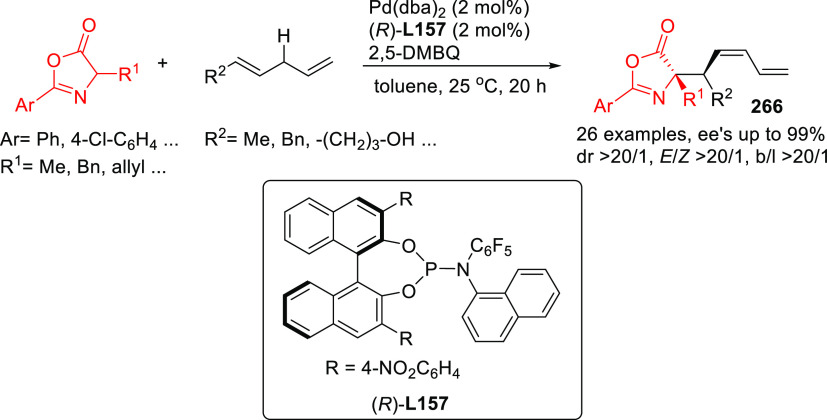
Pd-Catalyzed Asymmetric Allylic C–H Alkylation of a Range of 1,4-Dienes with Azlactones as Nucleophiles

**Figure 32:**
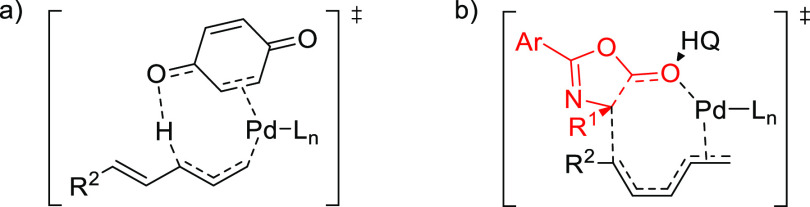
(a) Concerted proton and two-electron transfer process for the allylic C–H cleavage and (b) azlactone geometry controlled *Z*- and branch-selectivity.

Gong and co-workers had previously reported one example where chirality is created both at the nucleophilic center and at the allylic carbon center.^694^ This example involved a diene substrate (Scheme 278) and in subsequent work they have made a more extensive study of this type of reaction (Scheme 282).^698^ They found that the use of the Pd/(*R*)-**L157** catalytic system is able to promote the allylic alkylation of a broad range of 1,4-dienes with azlactones as nucleophiles. As a result a wide array of α,α-disubstituted α-amino acid surrogates **266** were formed in high yields and excellent diastereo-, *Z/E*-, regio-, and enantioselectivities (Scheme 282). This protocol have been used to synthesize lepadiformine C hydrochloride marine alkaloids. The combination of experimental studies and DFT computations suggest a novel concerted proton and two-electron transfer process for the allylic C–H cleavage (Figure 32a). DFT calculations also suggested that the *Z/E* selectivity and the regioselectivity are mainly controlled by the geometry and coordination mode of azalactones (Figure 32b).

**Scheme 283:**
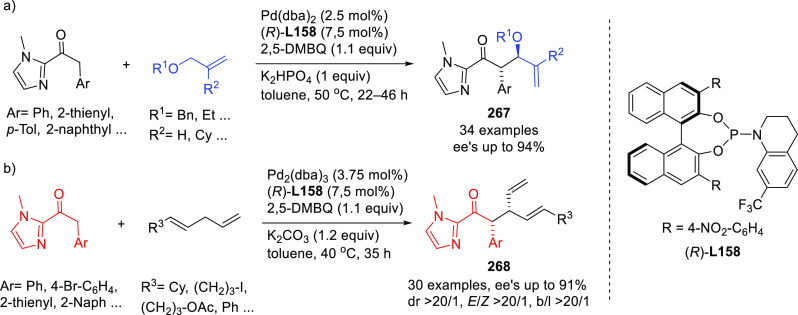
Asymmetric Allylic C–H Alkylation of 2-Acylimidazoles with (a) Allyl Ethers and (b) 1,4-Dienes

In subsequent work, Gong and co-workers developed an enantioselective Pd-catalyzed allylic C–H alkylation of allylic ethers using 2-acylimidazoles as nucleophiles (Scheme 283a).^699^ In all these reactions chirality was created at the allylic carbon as well as at the nucleophilic center in the product **267**. The resulting diastereoselectivity of the reaction was high. The Pd-catalyzed reaction of imidazoles and allylic ethers using phosphoramidite ligand (*R*)-**L158** afforded products **267** in good yields and with high levels of enantioselectivity (Scheme 283a). The diastereoselectivity (dr) and the branched/linear (b/l) ratio of the products were high (>20/1 dr and >20/1 b/l).

The reaction in Scheme 283a was also run with a wide range of allylic ethers with the aryl group of the imidazole being phenyl (Ar = Ph). These reactions were run for a slightly longer time (46 h) and gave good yields of coupled product **267** with high levels of enantioselectivity.

In a recent work, Gong and co-workers refined the regioselectivity in asymmetric allylic C–H alkylations by nucleophile coordination.^700^ Thus, as observed for azlactones (Figure 32b), DFT calculations suggest that coordination of 2-acylimidazoles-enabled inner-sphere attack mode for the enantioselective C–C bond-forming step, which is responsible for the high *E/Z*- and regioselectivities of the reaction. The authors took advantage of this feature to achieve high yields of **268** in excellent stereoselectivities in the allylic alkylation of 1,4-dienes with 2-acylimidazoles using the Pd/(*R*)-**L158** catalytic system (Scheme 283b). Interestingly, similarly high levels of regio- and stereoselectivities as well as *E*/*Z* selectivities were achieved using an allylic carbonate derivative, which indicates that both the classical allylic alkylation and the oxidative version share a similar transition state in the C–C bond formation step.

#### 4.4.3 Other Asymmetric Allylic C(sp^3^)–H C–C Bond-Forming Reactions

**Scheme 284:**
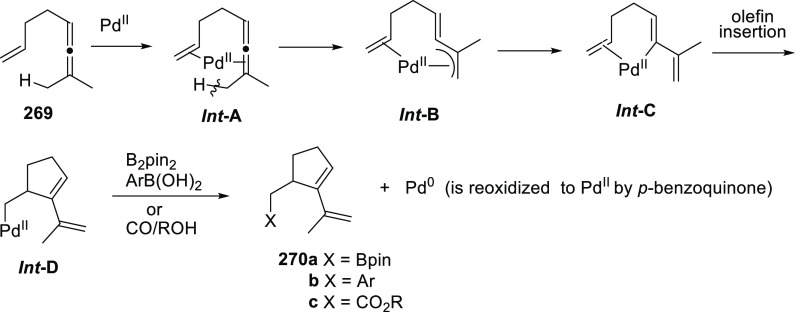
Allylic C(sp^3^)–H C–C Bond Forming Reactions of Allenes

Pd-catalyzed reactions of enallenes **269** have been found to provide cyclic products via allylic C–H bond cleavage. In these reactions a chelate Pd complex *Int***-A** is formed from which C–H activation is favored. A C–H bond cleavage in *Int***-A** would lead to a strained Pd η^3^-allyl intermediate *Int***-B** that rearranges to the more stable σ-form, dienyl-Pd complex *Int***-C** (Scheme 284). Insertion of the olefin into the dienyl-Pd bond in *Int***-C** produces organopalladium intermediate *Int***-D** that is typically quenched in situ by B_2_ pin_2_, ArB(OH)_2_, or CO/ROH to give products **270**.

**Scheme 285:**
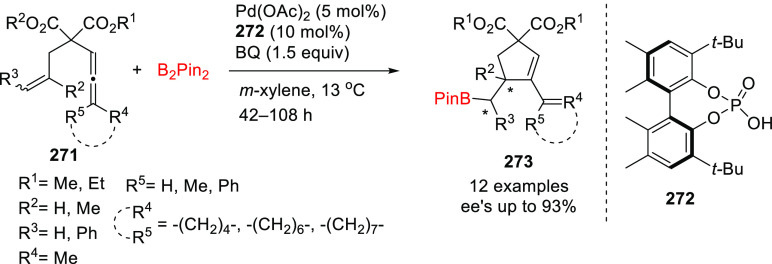
Pd(II)/Chiral Phosphate-Catalyzed Enantioselective Oxidative Carbocyclization–Borylation of Enallenes

Bäckvall’s group developed a Pd(II)/Brønsted acid-catalyzed enantioselective oxidative carbocyclization-borylation of enallenes **271**.^701^ The use of axially chiral biphenyl phosphoric acid **272** was found to be optimal to induce chirality during the migratory insertion of the alkene into the Pd–C bond. In this reaction the chiral phosphate replaces acetate on Pd. This novel synthetic procedure gives access to a range of borylated carbocycles **273** in high yields and enantioselectivities (Scheme 285).

**Scheme 286:**
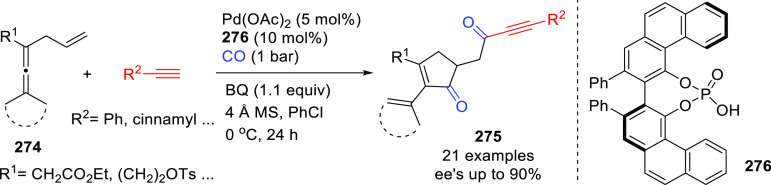
Pd(II)/Chiral Phosphate-Catalyzed Enantioselective Carbonylative Carbocyclization of Allenes with Alkynes

In a subsequent work, they developed an enantioselective Pd(II)-catalyzed carbonylative carbocyclization of allenes **274** with alkynes (Scheme 286).^702^ As a result a series of highly substituted cyclopentenones **275** with chirality at the α-position of the carbonyl group were obtained. Again the use of biaryl based phosphoric acids was key to achieve high levels of enantiocontrol. Thus, the use of sterically hindered biphenantrol-based phosphoric acid **276** induced high levels of enantioselectivity (up to 90% *ee*).

## 5 Cyclization Reactions via Pd-Catalyzed Interceptive Asymmetric Allylic Substitution

**Figure 33:**
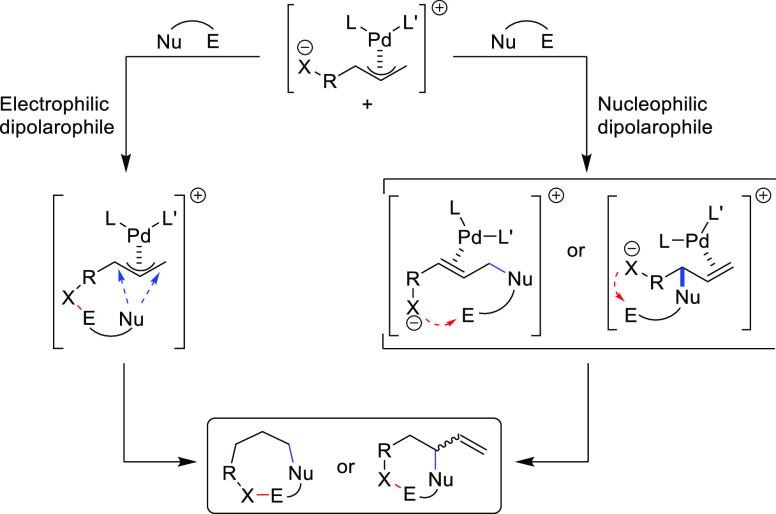
Pd-catalyzed cycloaddition reactions with an electrophilic or nucleophilic dipolarophile.

Over the past decades, catalytic cycloadditions proceeding through transition metal dipolar intermediates have become a powerful tool for synthesizing chiral carbo- and heterocyclic compounds.^637,703−705^ Among the transition metals used as catalysts for reactions of this type, Pd has played a dominant role. Such cycloadditions can proceed via reaction of the Pd-allyl dipolar species with either an electrophilic dipolarophile or a nucleophilic dipolarophile (Figure 33). While the use of electrophilic dipolarophiles has been successfully developed, the use of nucleophilic dipolarophiles has been much less explored, mainly because of the inherent selectivity in favor of linear products in the Pd-catalyzed intermolecular allylation.^706−708^ For reactions with nucleophilic dipolarophiles, Ir-catalysts, which generally favor formation of branched products, have been recently shown to represent a viable alternative.^709^

The Pd-allyl dipolar species are usually generated from vinylepoxides, vinyloxetanes, or vinylcarbonates, for which the Pd-allyl formation is favored by strain release or CO_2_ release, respectively. Vinylaziridines, vinyloxazolidinones, and vinylcyclopropanes are further cyclic substrates whose ring opening leads to Pd-allyl dipolar species. Silylated and heteroarylmethyl-substituted allylic acetates and carbonates are also prone to form trimethylenemethane-based Pd-allyl zwitterionic species able to undergo cycloadditions.

The intention of this section is to illustrate the enormous potential of enantioselective cycloaddition reactions via Pd-catalyzed allylic substitution, rather than present an exhaustive coverage of the literature.

### 5.1 [3 + 2] Cycloaddition Reactions

**Scheme 287:**
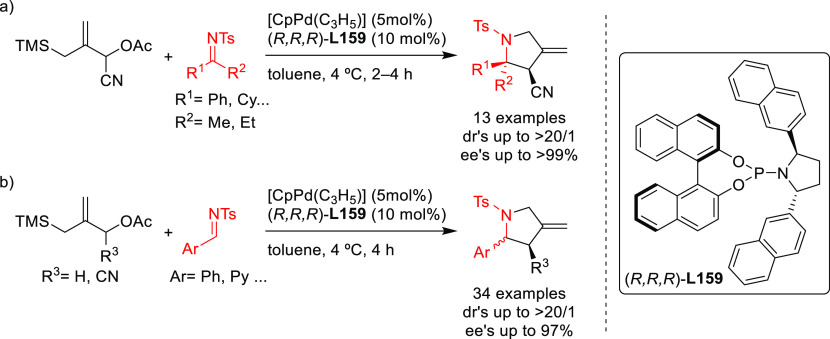
Synthesis of Pyrrolidine Cycloadducts

Pd-catalyzed [3 + 2] cycloaddition reactions represent a powerful method for the highly diastereo- and enantioselective formation of substituted five-membered rings. Among the ligands available to control these transformations, chiral monophosphoramidites have played a dominant role. For instance, Trost’s group expanded their early work on the synthesis of cyclopentanes^710^ to the construction of pyrrolidines by exploiting the [3 + 2] cycloaddition of Pd-trimethylenemethane complexes with a wide range of imines (Scheme 287).^711,712^ On the basis of this approach, the reaction of 1-cyano-2-((trimethylsilyl)methyl)allyl acetate with a range of ketimines gave access to the corresponding pyrrolidine cycloadducts containing adjacent quaternary and tertiary stereogenic centers in high yields and selectivities (dr’s up to >20/1 and *ee* values up to >99%; Scheme 287a).^711^ The same catalytic system was later used in the cycloaddition of 1-cyano-2-((trimethylsilyl)methyl)allyl acetate and 2-trimethylsilylmethyl allyl acetate with a range of aldimines (Scheme 287b).^712^

**Scheme 288:**
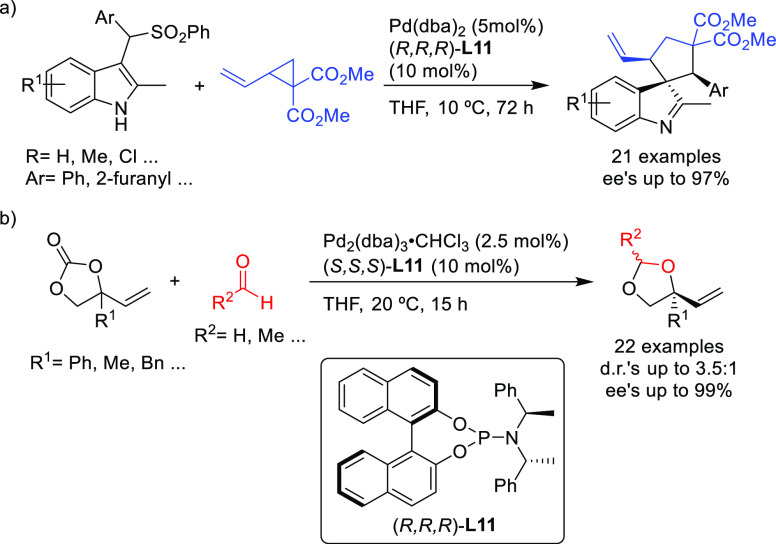
Synthesis of (a) Spirocyclopentane-1,2′-indolenines and (b) 1,3-Dioxolanes

Monophosphoramidite ligand **L11** has also been used in the Pd-catalyzed [3 + 2] cycloaddition of vinylcyclopropanes and α,β-unsaturated imines, formed in situ from aryl sulfonyl indoles. The reaction gave access to a range of spirocyclopentane-1,2′-indolenines in high enantioselectivities (up to 97% *ee*; Scheme 288a).^713^ Pd/(*S,S,S*)-**L11** was also used as catalyst for the synthesis of chiral 1,3-dioxolanes (*ee* values up to 99%; Scheme 288b).^714^

**Scheme 289:**
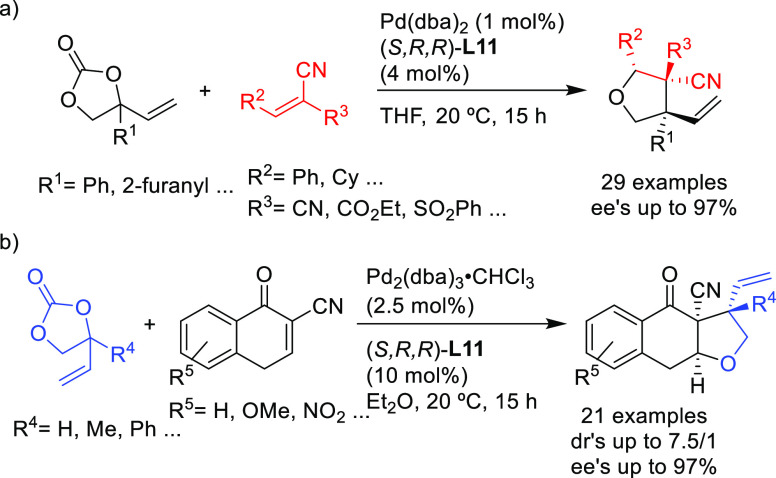
Synthesis of Highly Substituted (a) Tetrahydrofurans and (b) Furanobenzodihydropyrans Bearing Quaternary Stereocenters

Zhang’s group developed a decarboxylative cycloaddition of vinylethylene carbonates with activated olefins using the Pd/(*S,R,R*)-**L11** as catalyst (Scheme 289a). The reaction gave access to highly functionalized tetrahydrofurans bearing two adjacent quaternary stereocenters.^715^ The same catalyst was further used for the synthesis of furanobenzodihydropyrans bearing vicinal quaternary stereocenters in high yields with good to high enantio- and diastereoselectivities (Scheme 289b).^716^

**Scheme 290:**
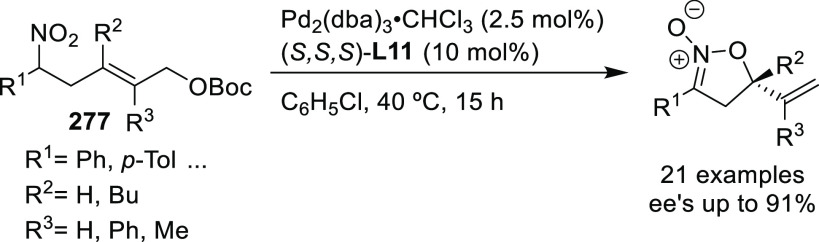
Synthesis of Isoxazoline *N*-Oxides

The same authors also developed intramolecular cycloadditions of allylic carbonates (**277**) with a nitroalkyl substituent to yield isoxazoline *N*-oxides with high *ee* values (up to 91%; Scheme 290) using Pd/(*S,S,S*)-**L11**.^717^

**Scheme 291:**
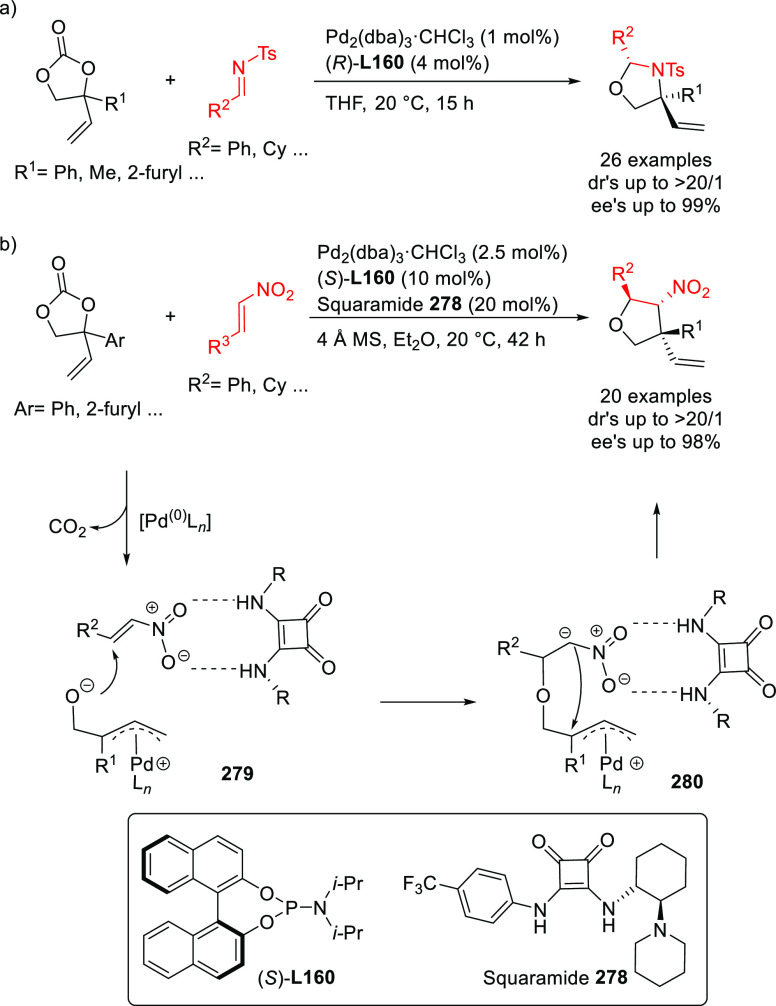
Synthesis of Chiral (a) 4-Vinyloxazolidines and (b) Tetrahydrofurans

Zhang’s group further studied the cycloadditions of vinylethylene carbonates with imines to yield substituted 4-vinyloxazolidines.^718^ A Pd complex derived from the chiral phosphoramidite **L160** proved to be an optimal catalyst leading to substituted 4-vinyloxazolidines in high yields, diastereo- and enantioselectivities (Scheme 291a). More recently, the same group extended this reaction to the use of β-nitroolefins employing a cooperative dual catalyst system comprising the squaramide **278** and Pd/(*S*)-**L160** (Scheme 291b).^719^ In this way, tetrahydrofurans containing three stereocenters were formed in good to high enantio- and diastereoselectivities via intermediates **279** and **280**.

**Scheme 292:**
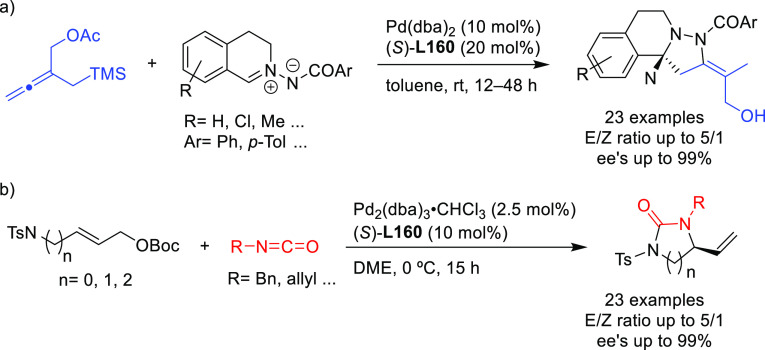
Synthesis of Chiral (a) Isoxazoline *N*-Oxides and (b) Ureas

Guo’s group developed a tandem [3 + 2] cycloaddition/allylation reaction of methylene-trimethylenemethane to yield hexahydropyrazole[5,1-*a*]isoquinoline derivatives in good-to-excellent enantioselectivities (*ee* values up to 99%) and moderate *E/Z* ratios (up to 5/1; Scheme 292a).^720^ Shortly afterward, Zhang’s group applied the same catalytic system for the synthesis of chiral ureas (imidazolidinones) through Pd-catalyzed cycloaddition of tosylamino-substituted allylic carbonates and isocyanates (Scheme 292b).^721^

**Scheme 293:**
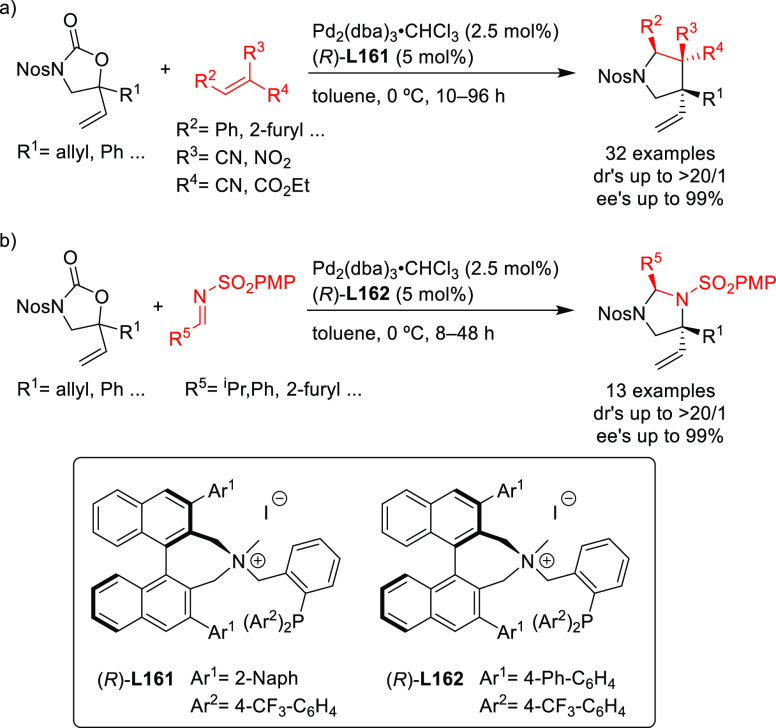
Synthesis of Highly Functionalized (a) Pyrrolidines and (b) Imidazolidines

Ooi’s group disclosed the use of chiral ammonium-phosphine hybrid ligands **L161** and **L162** in cycloadditions of 5-vinyloxazolidinones with a range of activated trisubstituted alkenes. In this manner, a variety of heavily substituted pyrrolidines was accessible with high diastero- and enantioselectivity using catalyst Pd/**L161** (Scheme 293a).^722,723^ The same group subsequently reported the reaction of 5-vinyloxazolidinones with *N*-sulfonyl imines (Scheme 293b).^724^ Switching to the chiral ammonium phosphine hybrid ligand **L162**, imidazolidines possessing α-amino quaternary stereocenters were prepared in excellent yields, diastereo- and enantioselectivities (dr’s up to >20/1 and *ee* values up to 99%).

**Scheme 294:**
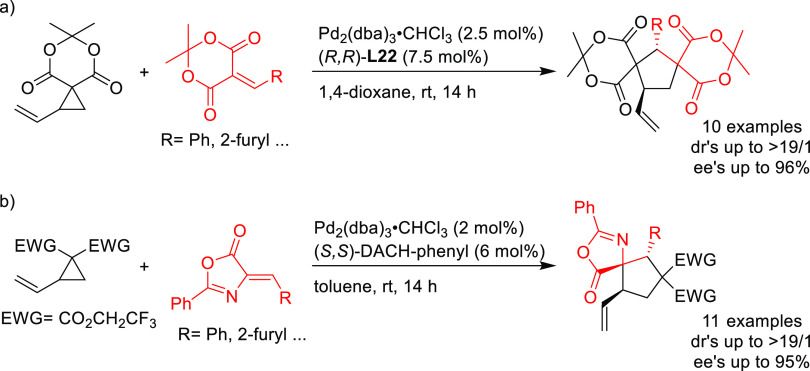
Synthesis of Highly Substituted Chiral Cyclopentanes

The Trost ligand (*R,R*)-**L22** (Scheme 19) has also been successfully used in cycloaddition reactions. Thus, the [3 + 2] cycloaddition of substituted vinylcyclopropanes with alkylidene derivatives of Meldrum’s acid gave highly substituted cyclopentanes with a spiranic structure in high selectivities (dr’s up to >19/1 and *ee* values up to 95%; Scheme 294a).^725^ The Pd/(*S,S*)-DACH-phenyl complex also efficiently catalyzed the cycloaddition of vinylcyclopropanes with azlactone alkylidenes (Scheme 294b).^725^

**Scheme 295:**
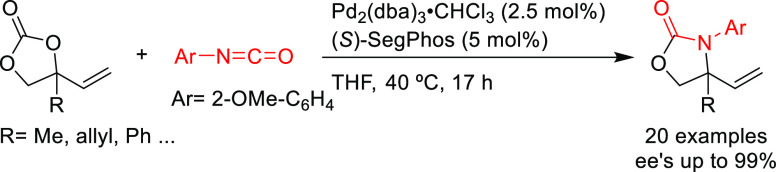
Synthesis of Chiral 4-Substituted 4-Vinyloxazolidin-2-ones

Other diphosphine ligands were also successfully used in [3 + 2] cycloadditions. Zhang’s group, for instance, used Pd/(*S*)-SegPhos as a catalyst in the cycloaddition of vinylethylene carbonates with isocyanates (Scheme 295).^726^ This protocol gave access to 4-substituted 4-vinyloxazolidin-2-ones in high yields and enantioselectivities (*ee* values up to 99%). The synthetic value of this procedure was demonstrated with the formal synthesis of the protein inhibitor MK-0731.

**Scheme 296:**
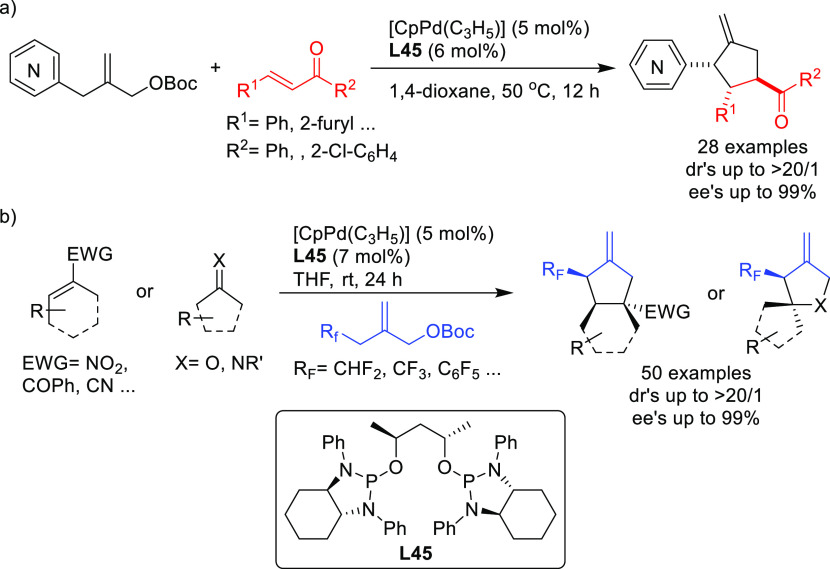
Synthesis of (a) Heteroaryl-Substituted Cyclopentanes and (b) Fluorocarbon-Substituted 5-Membered Rings

Trost’s group demonstrated that bisdiaminophosphites, like **L45** (Scheme 54), can also be successfully used in cycloadditions reactions. Thus, Pd/**L45** was used as catalyst in the cycloaddition of heteroaryl-containing allylic carbonates with linear α,β-unsaturated enones (Scheme 296a).^727^ Notably, this reaction tolerates the presence of most classes of nitrogen-containing heteroaromatic substituents, such as quinolones, pyridines, azoles, ..., giving access to various heteroaryl substituted cyclopentanes. Imines, aldehydes and nitroolefins can also be used in these cycloadditions instead of enones. More recently, the same authors extended the use of Pd/**L45** to reactions with β-fluorocarbon-containing allylic carbonates, giving access to fluorocarbon-substituted 5-membered rings (Scheme 296b).^728^

**Scheme 297:**
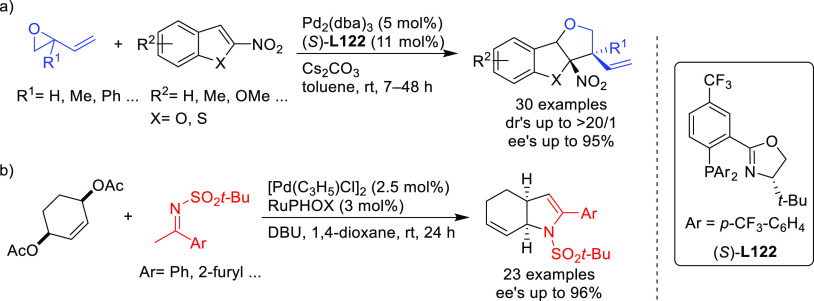
Synthesis of (a) Tetrahydrofurobenzofurans and Tetrahydrobenzothienofurans and (b) Tetrahydroindoles

PHOX-type ligands have also been applied to these reactions. Thus, You’s group reported the stereoselective formation of tetrahydrofurobenzofurans and tetrahydrobenzothienofurans through a Pd-catalyzed dearomative [3 + 2] cycloaddition of nitrobenzofurans using PHOX type ligand (*S*)-**L122** (Scheme 297a).^729^ More recently, Shen, Liu, and co-workers used Pd/RuPHOX as a catalyst to develop an alternative approach to the synthesis of tetrahydroindoles (Scheme 297b).^730^

### 5.2 [4+*n*] Cycloaddition Reactions

**Scheme 298:**
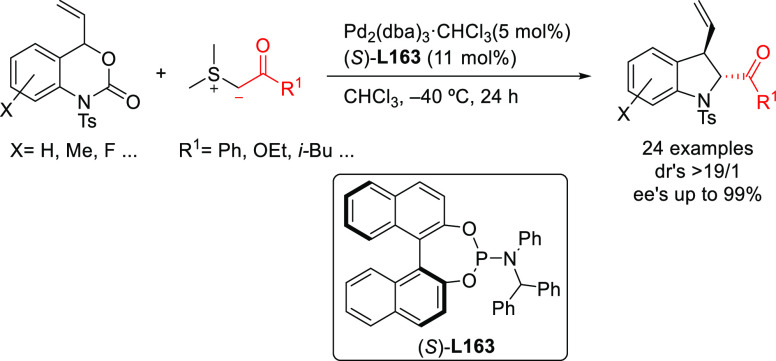
Synthesis of 3-Vinyl Indolines via [4+1] Cycloaddition

Other types of cycloadditions involving dipolar Pd η^3^-allyl intermediates have also been disclosed. For instance, Xiao reported a remarkable [4+1] cycloaddition with benzoxazinanones and sulfur ylides, resulting in the formation of synthetically useful 3-vinyl indolines using Pd/(*S*)-**L163** as catalyst (Scheme 298).^706^ The authors suggested that the electrostatic interaction between the sulfamide anion and sulfonium ion was critical to afford the branched regioselectivity and excellent levels of enantioselectivity (dr’s up to >19/1 and *ee* values up to 99%) observed. Furthermore, the authors proposed that sulfur ylides act as nucleophilic dipolarophiles, implying that the allylic alkylation step takes place before cyclization.

**Scheme 299:**
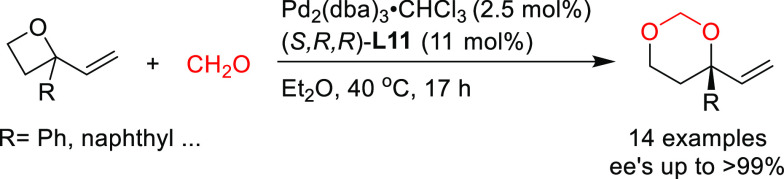
Synthesis of Enantiopure 1,3-Dioxanes

Pd-catalyzed [4+2] cycloadditions have also been extensively studied. For instance, the enantioselective [4+2] cycloaddition of vinyloxetanes with formaldehyde has proved to be an efficient method for the formation of enantiopure 1,3-dioxanes with a quaternary stereocenter (Scheme 299).^731^

**Scheme 300:**
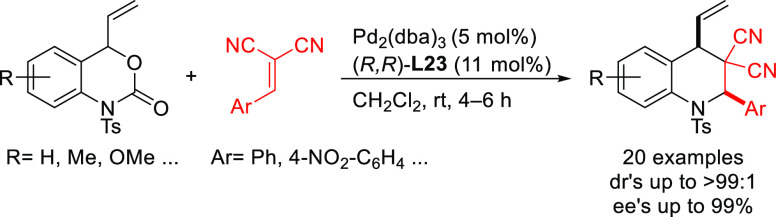
Synthesis of Chiral Hydroquinolines

In 2008, Tunge’s group reported the [4+2] cycloaddition of alkylidene derivatives of malononitrile with tosylated vinyl carbamates (Scheme 300).^732^ Using the Pd complex of Trost’s ligand (*R,R*)-**L23** (Scheme 26), hydroquinolines were obtained with high levels of diastereo- and enantioselectivities (dr’s up to >99:1 and *ee* values up to 99%).

**Scheme 301:**
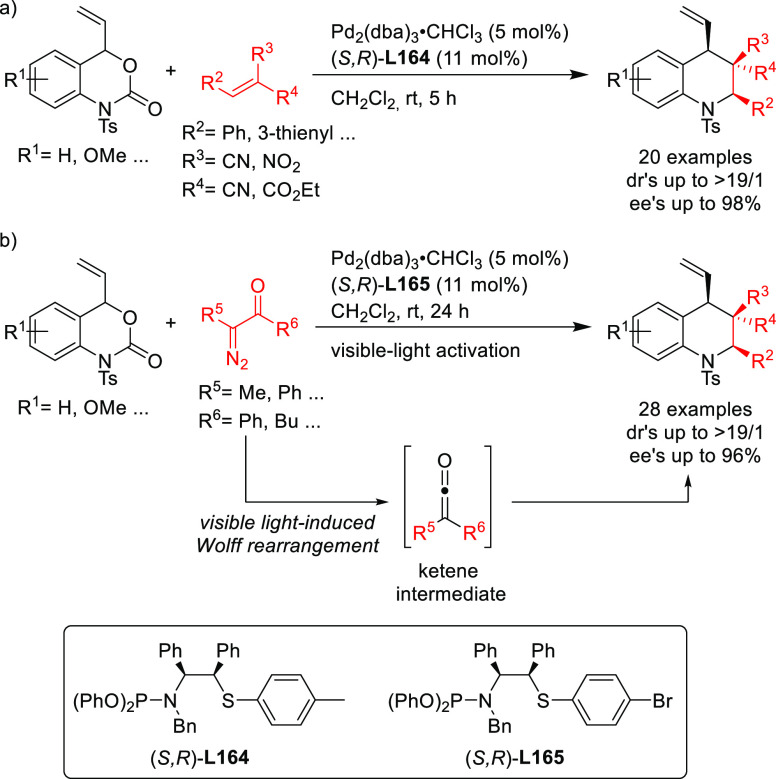
Synthesis of (a) Tetrahydroquinolines and (b) Quinolinones

Xiao and Alper employed a new hybrid *P,S* ligand **L164**, that combined a chiral β-amino sulfide and a simple diphenyl phosphite, in decarboxylative [4+2] cycloadditions (Scheme 301a).^733^ Again, Pd-polarized aza-*o*-xylylenes were intercepted by a variety of electron-deficient olefins to form highly functionalized tetrahydroquinolines bearing three contiguous stereocenters (dr’s typically >19/1 and *ee* values up to 98% *ee*). Xiao also recently reported the decarboxylative [4+2] cycloaddition of ketene intermediates with tosylated vinyl carbamates (Scheme 301b).^734^ Interception of the ketenes, generated in situ by a photolytic Wolff rearrangement of α-diazoketones, afforded chiral quinolinones in excellent yields with very high levels of stereoselectivities.

**Scheme 302:**
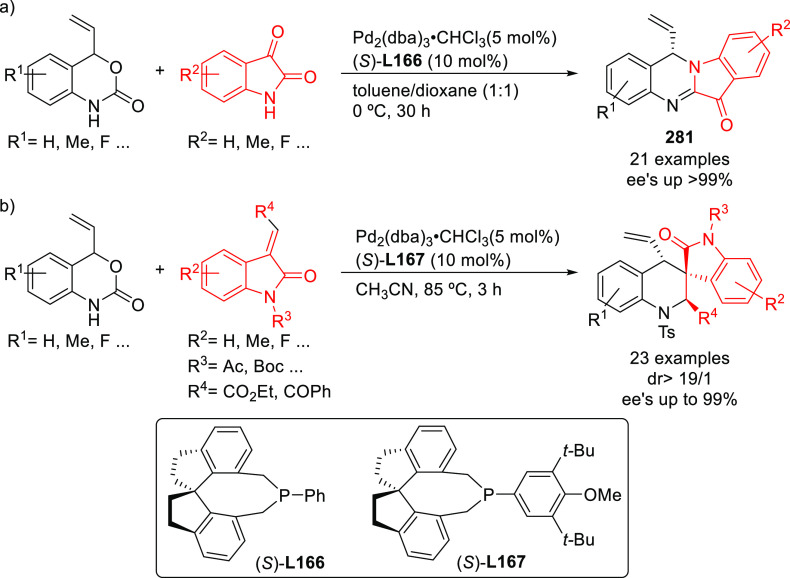
Synthesis of (a) Compounds **281** with Trypanthrin Skeleton and (b) Tetrahydroquinoline-Based 3,3-Spirooxindoles

The group of Mei and Shi reported the asymmetric formation of the trypanthrin skeleton **281** employing a decarboxylative [4+2] cyclization approach (Scheme 302a).^735^ As previously observed, the vinyl carbamates underwent Pd-catalyzed decarboxylation to form the Pd-polarized aza-*o*-xylylenes, which were subsequently intercepted by isatins through attack of the amide nitrogen atom at the internal allylic C atom. Subsequent intramolecular condensation of the resulting branched intermediate then led to the desired scaffold **281**. The Pd complex of the chiral spiro-phosphine ligand **L166** proved to be an efficient catalyst reacting with high chemo- and enantioselectivity (*ee* values up to >99%) with a wide range of substrates. The same group subsequently developed a further [4+2] cyclization strategy by intercepting the zwitterionic Pd-polarized aza-*o*-xylylene intermediate with methyleneindolinones through a reversible Michael-addition step (Scheme 302b).^736^ The subsequent intramolecular Pd-catalyzed asymmetric allylic alkylation produced the desired chiral tetrahydroquinoline-based 3,3-spirooxindole framework with excellent levels of diastereo- and enantioselectivity (dr’s > 191 and *ee* values up to 99%) using Pd/**L167** as catalyst.

**Scheme 303:**
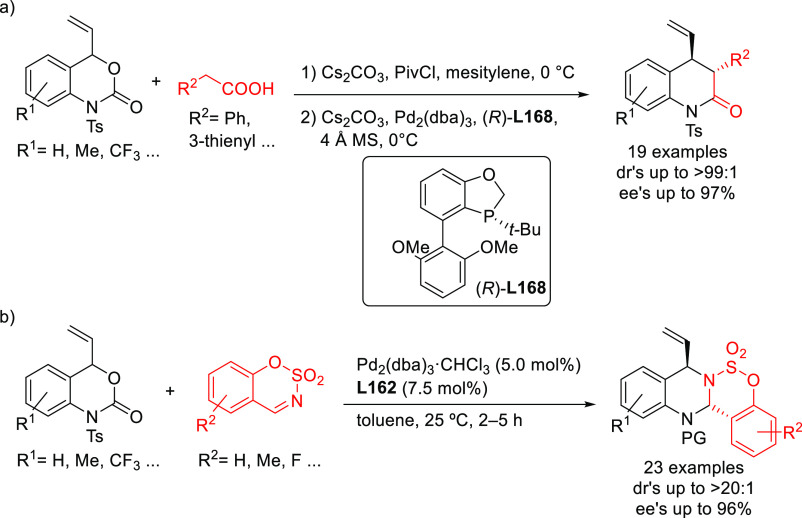
Synthesis of (a) 3,4-Dihydroquinolin-2-ones and (b) Tetrahydroquinazolines

In 2017, Deng and co-workers detailed their work on trapping the Pd-polarized aza-*o*-xylylenes with carboxylic acids to form 3,4-dihydroquinolin-2-one scaffolds possessing two adjacent tertiary stereocenters with high diastereo- and enantioselectivities (Scheme 303a).^737^ The carboxylic acid first reacts with pivaloyl chloride to form the corresponding mixed anhydride, which then intercepts the Pd η^3^-allyl intermediate. The *P*-chiral monophosphorus ligand (*R*)-**L168** induces the enantioselectivity in the final allylic alkylation step. In 2018, Guo reported the first Pd-catalyzed [4+2] cycloaddition of vinyl carbamates with sulfamate-derived cyclic imines (Scheme 303b),^738^ giving rise to tetrahydroquinazolines containing several functionalized rings in high yields with good to excellent levels of diastereo- and enantioselectivity (dr’s up to >20/1 and *ee* values up to 96%).

**Scheme 304:**
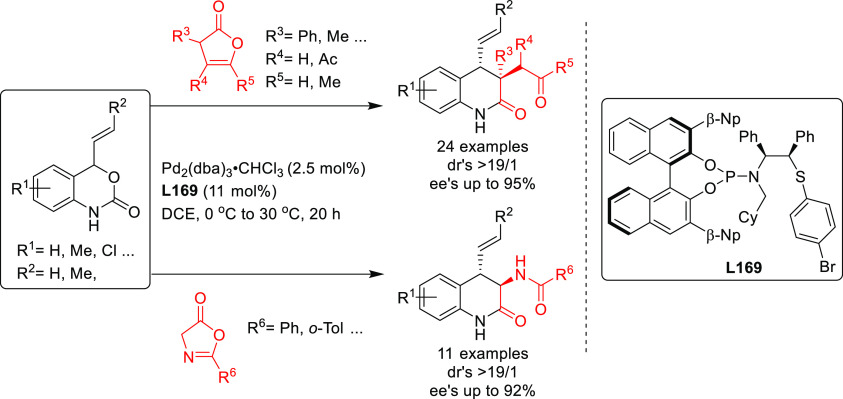
Synthesis of Chiral Dihydroquinol-2-ones

Another notable example of a [4+2] cycloaddition can be found in the synthesis of dihydroquinol-2-ones through reaction of vinyl carbamates with deconjugated butenolides and azlactones as nucleophilic dipolarophiles (Scheme 304).^739^ The success of this transformation was attributed to the use of chiral phosphoramidite-thioether ligand **L169** and control of the regioselectivity by a hydrogen bonding interaction.

**Scheme 305:**
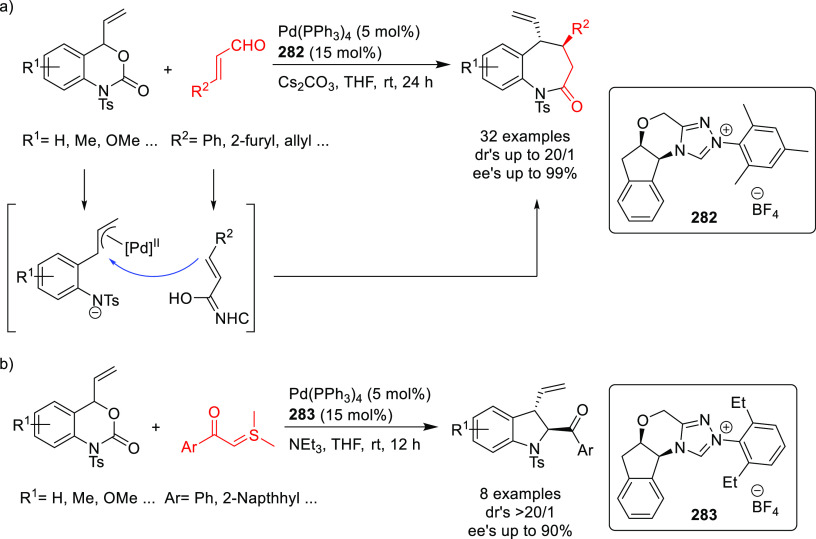
Synthesis of (a) Chiral Benzazepine Derivatives and (b) Indolines via Cooperative NHC Organocatalysis/Pd Catalysis

Glorius’s group reported the merger of N-heterocyclic carbene organocatalysis and Pd-catalysis for the [4+3] cycloaddition of enals and vinyl benzoxazinanones (Scheme 305a).^708^ This cooperative catalytic process yielded benzazepine derivatives in high yields and selectivities. The nucleophilic reactivity of the enal dipolarophiles is induced by formation of the NHC-homonenolate, which first attacks the electrophilic allyl-palladium intermediate. In a mechanistic investigation, a near first order dependence on Pd-catalyst and NHC was found.^707^ In that study, the crucial role of the phosphine ligand and a nonlinear effect of the chiral NHC organocatalyst (**282**) were established, prompting a search for the existence of a mixed Pd complex containing both the phosphine ligand and the chiral NHC. ESI-MS and X-ray investigations did indeed indicate the formation of a catalytically active [Pd(η^3^-allyl)(NHC)(PPh_3_)] complex. Furthermore, this method was extended to include a [4+1] cycloaddition, in which the [Pd(η^3^-allyl)(NHC **283**)(PPh_3_)] intermediate was postulated to be involved in the enantiodetermining step (Scheme 305b). This [4+1] cycloaddition with sulfur ylides led to indolines in high yields with excellent levels of enantio- and diastereoselectivity (dr >20:1 and *ee* values up 90%).

**Scheme 306:**
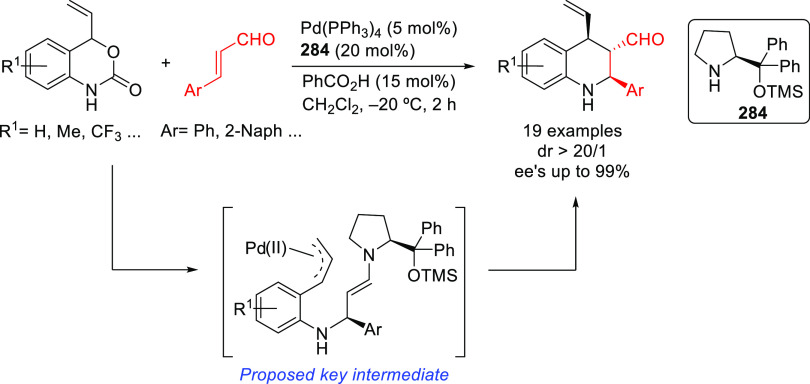
Synthesis of Vinyl Tetrahydroquinolines via [4+2] Cycloaddition Employing Cooperative Pd and Organocatalysis

Jørgensen’s group reported a complementary [4+2] cycloaddition employing cooperative Pd and organocatalysis (Scheme 306).^740^ Vinyl benzoxazinanones undergo a Pd-catalyzed decarboxylation generating a Pd-polarized aza-*o*-xylylene, which is intercepted by the iminium-ion formed from the α,β-unsaturated aldehydes and the amine cocatalyst **284**. A series of highly substituted vinyl tetrahydroquinolines were prepared in good yields with excellent levels of enantio- and diastereoselectivity (>98% *ee* and >20/1 dr).

**Scheme 307:**
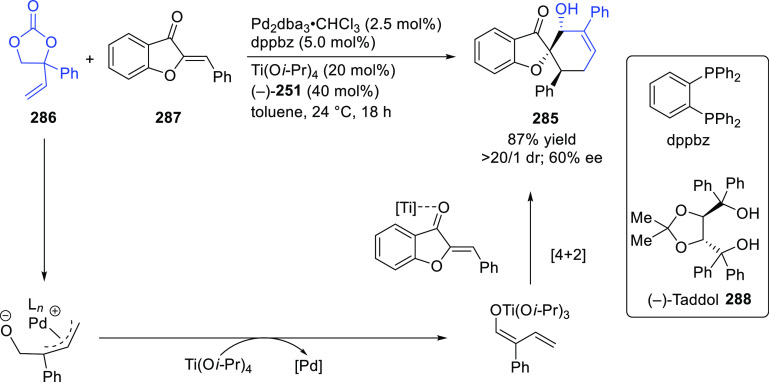
Synthesis of Spirocyclic Compound **285** from Vinylethylene Carbonate **286** and Aurone **287** via Formal [4+2] Cycloaddition

A remarkable variant of the cycloaddition of vinylethylene carbonates can be found in the work of Zhao’s group using a cooperative Pd/Lewis acid catalyst system. This strategy enabled the synthesis of spirocyclic compound **285** via [4+2] cycloaddition of vinylethylene carbonate **286** and aurone **287** (Scheme 307).^741^ The umpolung reactivity of vinylethylene carbonate results from a switch from the Pd η^3^-allyl alkoxide intermediate to a titanium dienolate, which then reacts with aurone **287** in a vinylogous Michael addition followed by aldol ring closure to form the spirocyclic compound. The use of a chiral phosphine ligand led to racemic product, which indicates that the enantiodetermining step is the vinylogous Michael addition mediated by a chiral Ti-TADDOL complex. The use of (−)-TADDOL **288** as chiral ligand afforded enantioenriched product **285** with modest 60% *ee* in 87% yield.

**Scheme 308:**
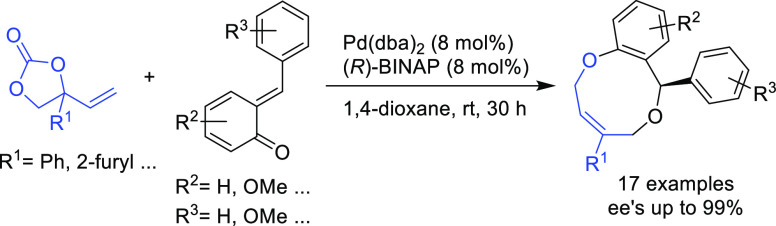
Synthesis of Enantiopure Benzo-1,6-dioxonanes

Fan’s group has recently developed a catalytic [4+5] cycloaddition of vinylethylene carbonates with *ortho*-quinone methides. By this method, a range of chiral benzo-1,6-dioxonanes was synthesized using Pd/(*R*)-BINAP as catalyst (Scheme 308).^742^

### 5.3 [5+*n*] Cycloaddition Reactions

**Scheme 309:**
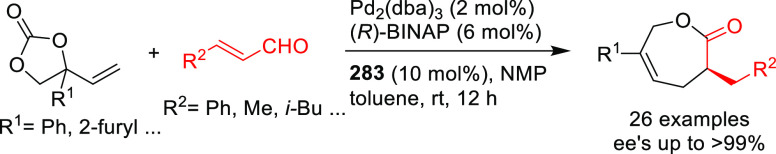
Synthesis of Chiral 7-Membered Ring Lactones

Another notable example of cooperative organocatalysis and Pd catalysis is the highly enantioselective [5+2] cycloaddition of vinylethylene carbonates with β-substituted α,β-unsaturated aldehydes. The reaction, which is one of the few examples of an inverse-electron-demand cycloaddition involving nucleophilic dipolarophiles, is enabled by a cooperative N-heterocyclic carbene (NHC)/Pd catalyst system. The use of chiral NHC **283** in combination with Pd/(*R*)-BINAP gave access to chiral 7-membered ring lactones (Scheme 309).^743^

**Scheme 310:**
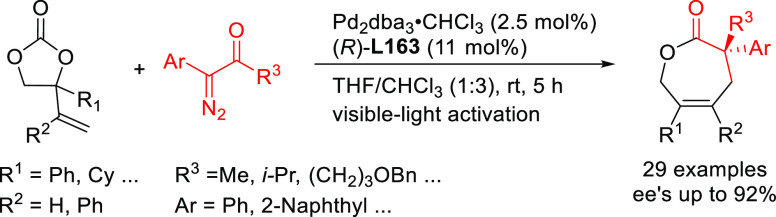
Synthesis of Chiral 7-Membered Ring Lactones

The combination of visible-light photoactivation with Pd catalysis, previously used in the synthesis of quinolinones (Scheme 301b), was also applied in the synthesis of seven-membered lactones via a [5+2] cycloaddition of vinylethylene carbonates and α-diazoketones (Scheme 310).^744^ The optimal chiral ligand was found to be phosphoramidite (*R*)-**L163**, affording the desired lactones in excellent yields and high *ee* values (up to 92%).

**Scheme 311:**
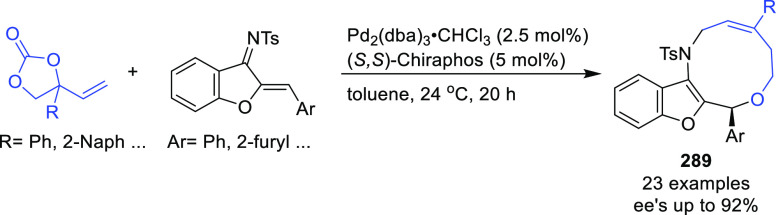
Synthesis of Benzofuran-Fused Nine-Membered Rings **289**

In 2017, Zhao reported a [5+4] cycloaddition between N-tosyl azadienes and the zwitterionic Pd η^3^-allyl intermediates generated from vinylethylene carbonates, affording benzofuran-fused nine-membered rings of type **289** (*ee* values up to 92%; Scheme 311).^745^ The high regioselectivity observed in these reactions was attributed to the presence of the sterically hindered tosyl group that favors a [5+4] cycloaddition over a [4+3] cycloaddition.^745,746^

### 5.4 [6+*n*] Cycloaddition Reactions

**Scheme 312:**
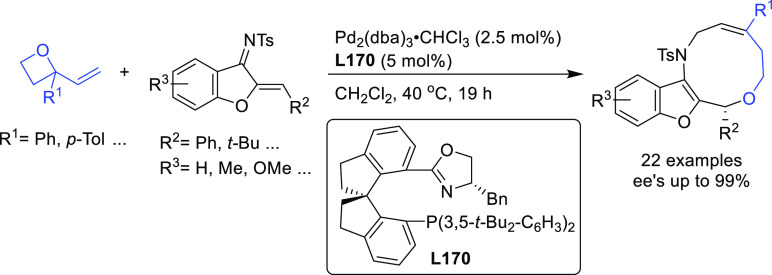
Synthesis of Chiral 10-Membered Heterocycles

Zhao’s group developed a new method to synthesize 10-membered heterocycles via Pd-catalyzed [6+4] cycloaddition. Using the spirocyclic phosphine-oxazoline ligand **L170** allowed efficient control of the reaction of vinyloxetanes with azadienes to yield a range of such heterocycles with excellent enantioselectivities (*ee* values up to 99%; Scheme 312).^747^ The reaction also performed well with vinyl epoxides, leading to the corresponding 9-membered ring compounds through a formal [6+3] cycloaddition.

## 6 Conclusions

This Review compiles the evolution, mechanistic understanding, and more recent advances in enantioselective Pd-catalyzed allylic substitution and decarboxylative and oxidative allylic substitutions. We also collect representative examples of cyclization reactions via Pd-catalyzed interceptive asymmetric allylic substitution. In the case of Pd-catalyzed allylic substitution, stabilized carbon nucleophiles, such as carbanions derived from 1,3-dicarbonyl compounds, maintain its prominent position. Apart from malonates and related stabilized C-nucleophiles including various functionalized malonates, β-diketones, 2-cyanoacetates, pyrroles, nitromethane, etc., N- and O-nucleophiles, and to a lesser, extent P- and S-nucleophiles have increasingly been used. Further improvements in ligand design and modular synthetic approaches have ended up with more finely tuned structures which provide a higher substrate and nucleophile scope. In this optimization process, mechanistic studies (by NMR and DFT) have played a key role. Among the reactions studied the alkylation of *rac*-1,3-diphenylallyl acetate using malonates and, especially, dimethyl malonate, as nucleophiles continued to serve as a benchmark reaction to evaluate the potential of new ligands in asymmetric catalysis. Remarkable efforts and progress have also been made to enlarge the scope of substrates (e.g., cyclic, 1,3-disubstituted with nonidentical substituents at the allylic termini and monosubstituted) and nucleophiles, thereby increasing the possibilities for applications to the synthesis of more complex organic molecules. Ligand design covered a wide array of structures ranging from monodentate P-donor ligands to homo- and heterodonor bidentate ligands. More than one hundred of new ligand families have been developed and applied with success. Although bidentate ligands continue to maintain a privileged position, some monodentate ligands such as the Taddol-based phosphoramidites and binaphthol-based phosphoramidites (the so-called Feringa type ligands) have provided outstanding results on more challenging and synthetically interesting substrates or nucleophiles. An important part of the research has also been directed to reduce the substrate dependency. Thus, some P–P′, P–N, and P–S ligand families use the same ligand to successfully alkylate disubstituted a broad range of hindered and unhindered substrates and even monosubstituted substrates. However, from a synthetic point of view, many recent studies were also devoted to synthetically more valuable and more challenging substrates and/or nucleophiles using well-established ligand scaffolds or slight modifications of them (e.g., Trost’s and PHOX type ligands). In this respect, some noteworthy studies have also been published on the use of well-known diphosphines, such as BINAP-type, BIPHEP, and SegPhos. Notable advances have emerged on the use of less stabilized enolates, such as ketones, lactams, etc., as well as enamines and nonstabilized C-nucleophiles (e.g., organozinc compounds). The number of applications of Pd-catalyzed asymmetric allylic substitution in the total synthesis of chiral compounds has increased steadily over the past decade. Most of the progress has been done with carbon nucleophiles while the variety of allylic substrates is still limited. Thus, heavily substituted acyclic allylic systems, as well as cyclic allylic systems in general, have been barely used as substrates in total synthesis, despite the availability of promising efficient chiral catalysts. This limitation is less pronounced with nitrogen nucleophiles that are fundamental for the preparation of chiral enantioenriched allylamines. In this case, cyclic allylic substrates have found ample application, but the scope of transformations based on acyclic substrates remains narrow. Oxygen nucleophiles, in spite of the promising results obtained with them, have only found minor application. The current level of development of allylic alkylation reactions will lead to a more intense use in total synthesis in the future with the incorporation of new allylic substrates and nucleophiles. This will advance faster as more chiral ligands become commercially available. The progress in the development of dual Pd/organocatalyst systems will also open up new possibilities for applying asymmetric allylic substitution in the synthesis of complex molecules. The increasing availability of high-throughput experimentation (HTE) methods will allow the fast screening of ligands, metals, and reaction conditions and help in overcoming the thought restrictions that have prevented until now a wider use of asymmetric allylic alkylation in total synthesis.

After the initial examples of decarboxylative catalysis in the 1980s, the development of new strategies such as decarboxylative allylations, protonations, and interceptive catalysis have significantly expanded the scope and synthetic utility of this transformation. The mild reaction conditions typically used in decarboxylative couplings compared to standard allylation conditions have enabled researchers to develop highly enantioselective variants of this transformation employing a variety of chiral ligands or chiral reagents. A combination of experimental and computational studies has greatly increased our mechanistic understanding of this transformation. Ultimately, this has shown that a broad range of factors that effect enantioselectivity, such as catalyst control, catalyst aggregation, and solvent, need to be carefully considered when designing an optimal catalytic system for different classes of substrate. The PHOX type P,N ligands and the Trost diphosphine ligands are complementary and optimal for most substrate classes tested to date, with the latter class being particularly effective for hindered substrates, such as those containing an α-aryl substituent. Many recent publications have highlighted the emergence of interceptive decarboxylative asymmetric catalysis, which generates a variety of zwitterionic complexes by decarboxylative palladium catalysis. These advances have allowed for the synthesis of a series of useful structural motifs possessing tertiary and quaternary stereocenters with excellent levels of enantio- and diastereoselectivity. Such advances have been translated to the preparation of compounds of use in medicinal chemistry and natural product synthesis. The application of Pd-mediated DAAA in total synthesis is a thriving research area, with more examples than other Pd-catalyzed AAA processes, and it will be of interest to follow the literature to determine whether its rapid uptake by the total synthesis community continues to grow in the future.

Pd-catalyzed oxidative allylic substitution where a nucleophile is replacing a hydrogen has recently led to important advances in synthetic organic chemistry. In particular, significant progress has been made with enantioselective versions of these reactions during the past decade. Enantioselective reactions involving carbon nucleophiles, as well as O- and N-nucleophiles have been developed. Various chiral ligands have been designed that can tolerate the oxidative conditions employed. In other cases, a chiral Brønsted acid such as a chiral phosphoric acid has been used. In almost all of the cases a benzoquinone has been used as the oxidant. The enantioselective allylic substitution is an important advance that now allows more simple starting materials that do not have to be prefunctionalized.
